# Proton and Metal Dication Affinities of Tetracyclic Imidazo[4,5-*b*]Pyridine-Based Molecules: Insights from Mass Spectrometry and DFT Analysis

**DOI:** 10.3390/molecules30132684

**Published:** 2025-06-21

**Authors:** Lucija Vrban, Ingrid Ana Martinac, Marijana Hranjec, Marijana Pocrnić, Nives Galić, Renata Kobetić, Robert Vianello

**Affiliations:** 1 Laboratory for the Computational Design and Synthesis of Functional Materials, Division of Organic Chemistry and Biochemistry, Ruđer Bošković Institute, 10000 Zagreb, Croatia; lucija.vrban@irb.hr; 2 Department of Chemistry, Faculty of Science, University of Zagreb, 10000 Zagreb, Croatia; ingridanamartinac12@gmail.com (I.A.M.); mpocrnic@chem.pmf.hr (M.P.); ngalic@chem.pmf.hr (N.G.); 3 Department of Organic Chemistry, Faculty of Chemical Engineering and Technology, University of Zagreb, 10000 Zagreb, Croatia; mhranjec@fkit.unizg.hr; 4 Laboratory for Biomolecular Interactions and Spectroscopy, Division of Organic Chemistry and Biochemistry, Ruđer Bošković Institute, 10000 Zagreb, Croatia

**Keywords:** imidazo[4,5-*b*]pyridine, metal dication affinity, proton affinity, mass spectrometry, DFT calculations, coordination chemistry, tetracyclic molecules, regioisomers

## Abstract

The imidazo[4,5-*b*]pyridine scaffold, a versatile heterocyclic system, is renowned for its biological and chemical significance, yet its coordination chemistry with biologically relevant metal dications remains underexplored. This study investigates the proton and metal dication affinities of twelve tetracyclic organic molecules based on the imidazo[4,5-*b*]pyridine core, focusing on their interactions with Ca(II), Mg(II), Zn(II), and Cu(II). Employing a dual approach of electrospray ionization mass spectrometry (ESI-MS) and density functional theory (DFT) calculations, we characterized the formation, stability, and structural features of metal–ligand complexes. ESI-MS revealed distinct binding behaviors, with Cu(II) and Zn(II) forming stable mono- and dinuclear complexes, often accompanied by reduction processes (e.g., Cu(II) to Cu(I)), while Ca(II) and Mg(II) exhibited lower affinities. DFT analysis elucidated the electronic structures and thermodynamic stabilities, highlighting the imidazole nitrogen as the primary binding site and the influence of regioisomeric variations on affinity. Substituent effects were found to modulate binding strength, with electron-donating groups enhancing basicity and metal coordination. These findings provide a comprehensive understanding of the coordination chemistry of imidazo[4,5-*b*]pyridine derivatives, offering insights into their potential applications in metalloenzyme modulation, metal-ion sensing, and therapeutic chelation.

## 1. Introduction

The imidazo[4,5-*b*]pyridine scaffold, a fused heterocyclic system of imidazole and pyridine rings, has emerged as a privileged structure in chemical and biological research. Its appeal lies in its structural versatility and electronic properties, which enable it to participate in a variety of intermolecular interactions, including hydrogen bonding, π–π stacking, and coordination with metal ions. These attributes make its derivatives promising candidates for drug discovery, with demonstrated antimicrobial, anticancer, antiviral, and enzyme inhibitory activities [1,2,3,4,5]. The presence of nitrogen atoms in the heterocyclic core enhances its capacity to act as a ligand, a feature that has been exploited in the design of biologically active compounds targeting metalloenzymes and metal-dependent pathways [6]. Despite their well-explored pharmacological potential, the coordination chemistry of these derivatives with metal ions—particularly biologically relevant divalent cations—remains underexplored.

Divalent metal cations, such as Ca(II), Mg(II), Zn(II), and Cu(II), are ubiquitous in biological systems and play critical roles in maintaining cellular homeostasis and facilitating biochemical processes. Calcium and magnesium ions, for instance, are critical for signal transduction and structural stabilization of biomolecules, respectively, while zinc and copper ions serve as essential cofactors in enzymatic catalysis and redox reactions [7,8]. This study focuses on these four metal ions due to their high biological relevance, as they are among the most prevalent divalent cations in metalloenzymes and cellular pathways [9]. Their interactions with organic ligands are governed by factors such as ligand denticity, electronic properties, and steric effects, making the study of metal-binding affinities a key step in understanding their biological roles and potential applications. Such interactions could not only elucidate the mechanistic basis of their biological activities but also unlock new applications in areas such as metal-ion sensing, catalysis, and the development of chelating agents for therapeutic or industrial purposes [10].

Historically, the affinity of heterocyclic compounds for metal ions has been investigated using a variety of experimental and theoretical approaches. Mass spectrometry stands out as a powerful technique for detecting metal–ligand complexes and quantifying binding strengths, owing to its sensitivity and ability to analyze gas-phase interactions [11]. Complementarily, DFT computational analysis provides detailed insights into the electronic structures, coordination geometries, and thermodynamic parameters of these complexes, offering a molecular-level understanding that enhances experimental findings [12]. In the context of imidazo[4,5-*b*]pyridine derivatives, the synergy of these methods is particularly valuable, given the scaffold’s structural complexity and the nuanced effects of substituents on its metal-binding behavior.

**Scheme 1:**
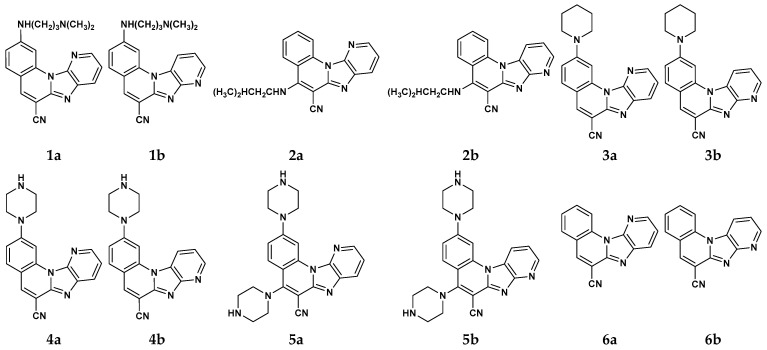
Tetracyclic molecules investigated in this work.

In this study, we investigate the metal cation affinities of twelve organic molecules built upon the imidazo[4,5-*b*]pyridine core fused in a tetracyclic structural framework (Scheme 1), with a focus on their interactions with Ca(II), Mg(II), Zn(II), and Cu(II). In addition, these will be evaluated and interpreted relative to their affinities for the smallest cation—the proton (H^+^)—providing an excellent reference system deprived of any steric hindrance. These molecules, previously characterized for their diverse biological activities [13,14], were subjected to mass spectrometry measurements to evaluate complex formation and relative binding strengths, alongside DFT calculations to probe the energetic and structural aspects of these interactions. By systematically varying substituents on the imidazo[4,5-*b*]pyridine framework, we aimed to delineate how structural modifications influence metal-binding affinity and selectivity, a critical consideration for tailoring these compounds to specific applications. This dual experimental–computational approach not only provides a robust dataset on their coordination properties but also establishes a methodological framework for future studies of heterocyclic ligands.

The motivation for this work stems from the dual potential of imidazo[4,5-*b*]pyridine derivatives as both biologically active agents and metal-coordinating platforms. While their pharmacological properties have been well documented, their capacity to engage with metal dications offers a new dimension for exploration, potentially bridging their biological roles with applications in coordination chemistry. For instance, high-affinity ligands for Zn(II) or Cu(II) could find use in modulating metalloenzyme activity or mitigating metal-ion toxicity, whereas selective binders of Ca(II) or Mg(II) might serve as tools for studying cellular signaling. Herein, we present the comprehensive experimental and computational analysis of how the imidazo[4,5-*b*]pyridine scaffold interacts with biologically relevant metal dications. This study lays the groundwork for expanding the functional repertoire of these molecules, highlighting their promise beyond traditional therapeutic contexts.

## 2. Results and Discussion

The molecules studied in this work include variously substituted tetracyclic organic compounds based on an imidazo[4,5-b]pyridine core fused to two phenyl rings. Their synthesis and evaluation of several biological properties were reported earlier [13,14], while here, we investigate their metal-binding properties using MS measurements and DFT calculations. Substituents, including nitrogen-bound and open-chain or cyclic moieties, were used to mono- or di-substitute the parent skeletons **6a** and **6b**. All compounds studied feature a strong electron-withdrawing 6-CN group, which counterbalances the electron-donating effects of the N-substituents, thereby tuning the binding affinities for metal cations. We also highlight a key structural feature: all compounds form pairs of regioisomers based on the position of the pyridine nitrogen (N8 or N11, denoted as ***X*a** and ***X*b** throughout the text). As discussed below, this subtle difference significantly alters ligand–metal cation interactions, offering a tool to tailor binding affinities for applications such as catalysis or metal ion sensing.

### 2.1. Mass Spectrometry

Electrospray ionization mass spectrometry (ESI-MS) is a powerful technique for characterizing metal coordination complexes. Yet, assigning the ESI mass spectra of such compounds can be challenging [15], as the considerations involved differ significantly from those for purely organic samples. Metal complexes may lose or gain labile ligands, become oxidized or reduced, or react with oxygen or moisture, depending on the specific ESI conditions. Notably, the detection of fragile structures bound by weaker intermolecular forces is often limited in ESI-MS due to relatively harsh instrument conditions, such as high voltages or desolvation temperatures. In this work, ESI-MS analysis focuses on regioisomers **1** and **2** and analyzes their Cu(II) and Zn(II) binding affinities in detail, while computational DFT results provide a broader overview.

**Figure 1:**
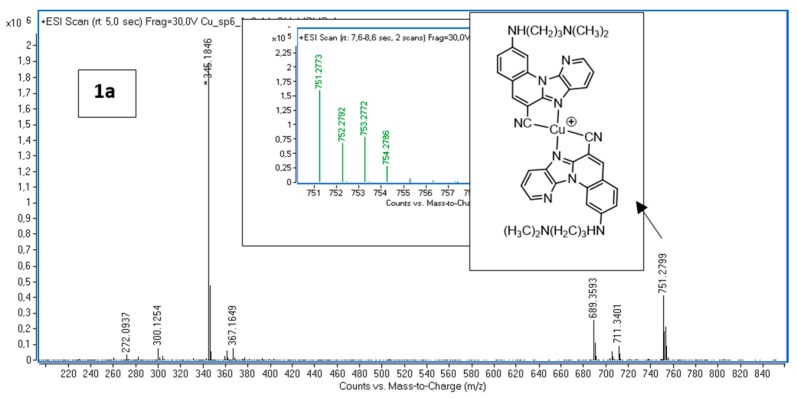
Solution TIC spectrum with **1a** and Cu(II) acetate in MeOH in a 3:1 ratio. Identical results were obtained for other ratios, indicating that the structure with *m*/*z* 751 is the most favorable.

Compound **1a** forms a single complex ion with Cu(II) at *m*/*z* 751.2799, identified as [**1a**_2_Cu]^+^ (Figure 1). MS/MS experiments confirmed the complex’s composition, with its proposed structure shown in the inset of Figure 1, displaying the ESI+ mode full-scan spectrum. Isotopic distribution analysis verified a singly charged ion, suggesting Cu(II) reduction to Cu(I) in the gas phase, likely due to electron transfer within charged methanol droplets in the ESI environment. Analogous Cu(II) reduction has been noted in prior studies, [16,17,18] attributed to inner-sphere (ligand-to-metal electron transfer within the coordination sphere) and outer-sphere (electron capture from the ESI environment, often via corona discharge or solvent effects) mechanisms. The copper ion’s complexation with polydentate ligands forms stable complexes, facilitating the transfer of multiply charged ions into the gas phase, stabilized by ligand interactions.

While the TIC spectrum predominantly shows the [**1a**_2_Cu]^+^ complex at *m*/*z* 751.2799, the formation of a [**1a**Cu]^+^ species cannot be entirely excluded. No signals corresponding to a 1:1 complex were observed in the *m*/*z* 100–3000 range, suggesting that such a species, if formed, may be labile or present below the detection limit. Future studies with varied ligand-to-metal ratios or softer ionization techniques could further probe this possibility.

**Figure 2:**
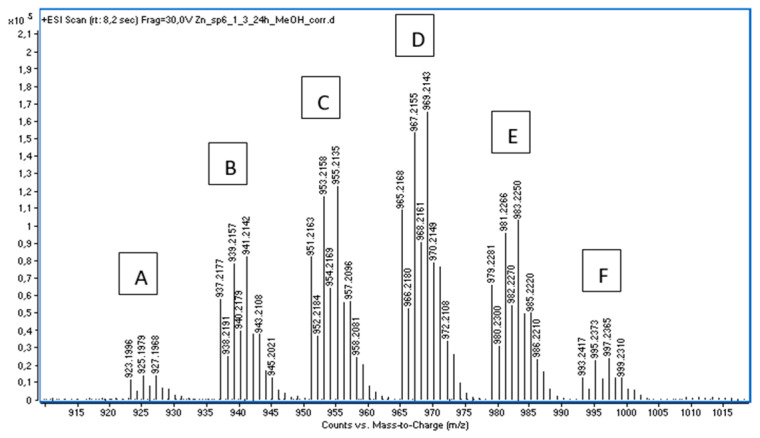
Part of the solution TIC spectrum of **1a** and Zn(II) acetate in methanol, with signals indicating dinuclear complex ions A–F. The detailed signal assignation is presented in Table 1.

The interaction of **1b** with Cu yielded an identical TIC spectrum as that for its regioisomeric analog **1a**, analogously assigned as [**1b**_2_Cu]^+^ (Figure S1). The *m*/*z* 751 signals for [**1a**_2_Cu]^+^ and [**1b**_2_Cu]^+^ show nearly identical isotopic envelopes and MS/MS fragmentation patterns, suggesting minimal regioisomeric effects on the gas-phase ion structure. Still, DFT calculations (see later) indicate a slightly higher Cu(II) affinity for **1b** (~2–3 kcal mol^−1^), which may not be discernible in ESI-MS due to Cu(II) reduction to Cu(I). Higher-resolution MS or ion mobility studies could further probe subtle differences in complex stability or conformation. In contrast, both regioisomers form a series of complex ions with Zn(II), having combinations of OH^−^, acetate, and formate as counter ions. TIC spectra show that **1a** mainly forms dinuclear complex ions, with counterions as links between metals. Thus, for **1a**, signals at *m*/*z* 923, 937, 951, 965, 979, and 993 were observed. The signal at *m*/*z* 951, assigned as [2(**1a**) + 2 Zn^2+^ + 2(HCOO^−^) + (CH_3_COO^−^)]^+^, had the highest abundance (Figure 2, Table 1). Compound **1b** is dominated by mononuclear complex ions detected as signals *m*/*z* 783, 797, and 813. Three signals of lower abundance for dinuclear complex ions with Zn(II) ion (*m/z* 909, 923, and 939) were also observed (Table S1 and Figure S2).

Several types of mononuclear complex ions are formed between Cu(II) acetate and **2a** in methanol. The signals at *m*/*z* 693, 738, and 796 are assigned as the mononuclear complex ions (Figure S4), with the major signal (*m*/*z* 693) assigned as [2(**2a**) + Cu]^+^ (Figure S3). In addition, a dinuclear complex ion was also observed at *m*/*z* 863. Thus, **2a** forms mono- and dinuclear complex ions with Cu(II) acetate, which distinguishes **2a** from **2b** but also from **1a** and **1b**. The reduction of Cu(II) to Cu(I) within the charge droplet is also present, since all observed complex ions involve a Cu(I) ion.

**Figure 3:**
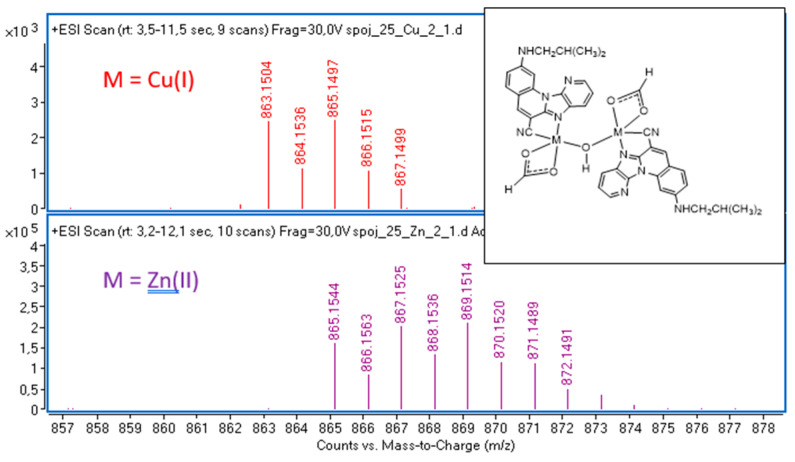
Part of the solution TIC spectra of **2a** with Cu(I) or Zn(II) acetate in methanol showing a more detailed isotopic distribution of dinuclear complexes. Full TIC spectra are shown in Figure S4.

The interaction of **2a** with Zn(II) offered mono- and dinuclear complex ions, similar to the structures observed with Cu(II) (Figure S4). For example, a signal at *m*/*z* 865 was observed analogously to the signal at *m*/*z* 863, which was assigned as a complex ion of Cu(II) and **2a**. Consequently, the regioisomer **2a** formed two identical dinuclear complex ions with both metal ions Cu(I) and Zn(II), as shown in Figure 3.

**Figure 4:**
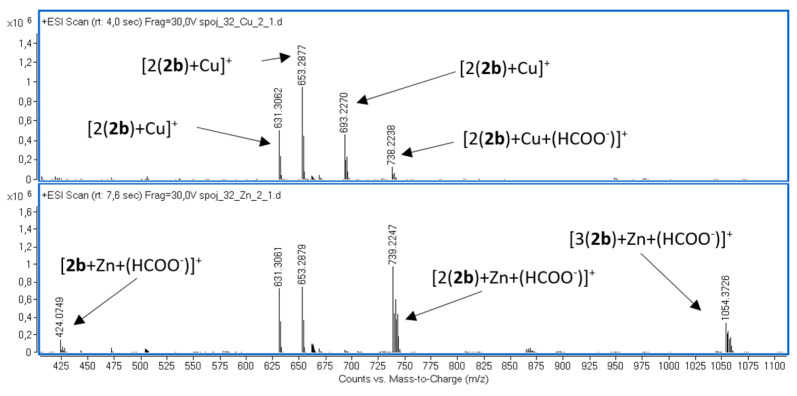
The TIC spectra of a solution of **2b** with Cu(II) or Zn(II) acetate in methanol that shows the signals corresponding to the mononuclear complex ions.

**Figure 5:**
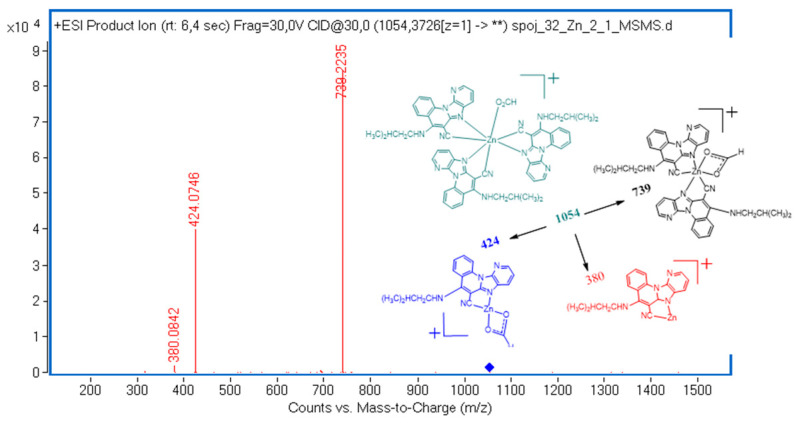
Fragmentation pathways for the dissociation of the mixed ligand complex ion (*m*/*z* 1054) assigned as [3(**2b**) + Zn + (HCOO^−^)]^+^. The scheme is based on the CID (MS2, scan 3726, CID at 30 eV) targeting the ^64^Zn isotopic peak. The chemical structure elucidates the likely final product ions generated during the MS/MS experiment.

Compound **2b** solely formed mononuclear complexes with Cu(II) and Zn(II) (Figure 4). Notably, a signal at *m*/*z* 1054 was detected, corresponding to a Zn(II) complex with three **2b** ligands, despite the observed Zn:**2b** interaction ratio of 1:2. In contrast, complex ions with two ligands and varying counterion combinations were observed for molecules **1a**, **1b**, and **2a** but not for **2b**. The structures of these complex ions were proposed based on MS/MS experimental results, such as those for the *m*/*z* 1054 signal (Figure 5). The exclusive formation of mononuclear complexes by **2b**, including the [Zn(**2b**)_3_]^2+^ species, contrasts with **2a**’s ability to form both mono- and dinuclear complexes with Zn(II) and Cu(II). This is likely due to reduced steric hindrance in **2b**, where the pyridine nitrogen orientation facilitates higher coordination numbers, and its higher basicity enhances charge delocalization. In **2a**, steric constraints from the N8 pyridine nitrogen favor dinuclear complexes stabilized by counterion bridges, preventing the formation of a [Zn(**2a**)_3_]^2+^ complex.

### 2.2. Computational DFT Analysis

The purpose of the computational analysis was to provide insights into the electronic structures, coordination geometries, and thermodynamic stabilities of the metal–ligand complexes investigated experimentally. These results complement experimental measurements and allow us to systematically assess how structural variations within the tetracyclic framework influence metal-binding behavior, providing a comprehensive dataset that complements their previously documented biological profiles.

#### 2.2.1. Gas-Phase Basicities and Affinities for a Proton

Before we engage into the discussion for the computed 1:1 and 2:1 metal–ligand complexes, we will initiate the computational analysis by inspecting the matching affinities towards the smallest cation—a proton (H^+^)—to probe the intrinsic nucleophilicities of various nitrogen positions within the studies systems. The computed gas-phase basicities are presented in Table 2, corresponding to the most favorable processes, while a complete analysis for all nitrogen sites is given in the Supporting Information (Table S2). 

The most basic site in all investigated ligands is the unsaturated imidazole nitrogen, which is consistent with the higher basicity of imidazole (p*K*_a_ ~7.0) compared to pyridine (p*K*_a_ ~5.2) [19]. This trend is observed across all derivatives. The next most basic sites are the pyridine nitrogen and aliphatic amine groups, which have similar basicities (Table S2). These are followed by the aniline and nitrile nitrogen atoms, which exhibit comparable basicities. The annulated imidazole nitrogen displays the weakest basicity, with its nucleophilicity significantly reduced due to steric hindrance and ring strain.

Importantly, the basicity of regioisomers ***X*b** is consistently higher than that of ***X*a**, due to the more favorable orientation of its pyridine nitrogen, which enhances the stabilization of the cation coordination at the imidazole nitrogen. In ***X*a**, where the pyridine nitrogen is oriented opposite to the imidazole nitrogen, the cation’s interaction with the latter is hindered by nearby pyridine C–H groups. In contrast, this hindrance is absent in ***X*b**, resulting in higher basicity. This effect, quantified as 2–3 kcal mol^−1^ in favor of ***X*b**, is significant and becomes more pronounced with larger metal dications, as discussed below.

The least basic systems are the parent compounds **6a** and **6b**, with computed gas-phase basicities of 219.7 and 222.3 kcal mol^−1^, respectively. These values are close to the experimental gas-phase basicity of benzimidazole (220.0 kcal mol^−1^) [20], indicating that the basicity remains largely unchanged despite the extended aromatic π-system in the tetracyclic structures of **6a** and **6b**. As expected, this π-system extension could enhance basicity through increased cationic resonance stabilization in the conjugate acid. However, this effect is counteracted by the strong electron-withdrawing 6-CN group near the protonation site, which reduces electron density and lowers basicity. Thus, the basicity of tetracyclic **6a** and **6b** remains comparable to that of bicyclic benzimidazole. Their further substitution increases basicity, primarily due to the electron-donating effects of nitrogen-containing substituents, which stabilize the positive charge in protonated forms via resonance. The weakest basicity enhancement occurs when these substituent are introduced on the phenyl ring farthest from the protonation site, as in **1**, **3**, and **4**. There, 2-substituents contribute 9–10 kcal mol^−1^ to the basicity, increasing in the order –NH(CH_2_)_3_N(CH₃)₂ < piperazine < piperidine, consistent with their electron-donating abilities, as measured using Hammett–Taft substituent constants [21]. In contrast, substitution on the phenyl ring closer to the protonation site, as in **2**, results in a greater effect, making this system 4–5 kcal mol^−1^ more basic than **1**. Consequently, the disubstituted compound **5** exhibits a synergistic effect, making it the most basic of all investigated systems. With gas-phase basicity values of approximately 237 kcal mol^−1^ for **5a** and 240 kcal mol^−1^ for **5b**, both regioisomers qualify as neutral organic superbases [22,23].

#### 2.2.2. Metal Dication Affinities

The gas-phase basicities serve as a reference for interpreting the computed metal cation affinities (Table 2). Notably, all 1:1 affinities for Mg(II), and especially Ca(II), are significantly lower (by 50–100 kcal mol^−1^) than those for the proton, suggesting low thermodynamic favorability for their complex formation. In contrast, affinities for Zn(II) substantially exceed those for the proton, with this trend being particularly pronounced for Cu(II), where some affinities (e.g., for disubstituted ligands) are up to 100 kcal mol^−1^ higher than proton affinities. These trends can be rationalized using Hard–Soft Acid–Base (HSAB) theory [24,25], where “hard” acids prefer “hard” bases (e.g., O-donor ligands), and “soft” acids prefer “soft” bases (e.g., S-donor ligands), with N-donors being intermediate. Ca(II) and Mg(II), as hard acids, exhibit strong affinities for O-donor ligands due to their high electronegativity, resulting in low affinities for nitrogen-rich ligands, making 1:1 complex formation unlikely. In contrast, Zn(II), a borderline acid, shows versatility, with good affinity for O-donor, N-donor, and even S-donor ligands (e.g., cysteine in biological contexts). This flexibility arises from its filled d^10^ orbitals, which lack ligand field stabilization effects, making Zn(II) more attractive for the investigated ligands compared to Mg(II) and Ca(II). Cu(II), with its d^9^ configuration, displays a strong affinity for N-donor ligands due to enhanced d-orbital overlap, evident in its highly exergonic affinities, often exceeding 300 kcal mol^−1^. Similar to gas-phase basicities, ***X*b** regioisomers exhibit stronger binding at the imidazole nitrogen than their ***X*a** analogs, for the same reasons discussed for proton affinities (e.g., favorable pyridine nitrogen orientation in ***X*b**).

Additionally, Table 2 shows that metal dication affinities increase with N-substitution, following the order –NH(CH_2_)_3_N(CH_3_)_2_ < piperazine < piperidine and monosubstituted < disubstituted. The only exception are Cu(II) affinities, which follow an inverse trend, piperidine < piperazine < –NH(CH_2_)_3_N(CH_3_)_2_, likely due to metal’s unique d^9^ electronic configuration. This leads to the Jahn–Teller distortion in Cu(II) complexes, typically an elongation along the axial ligands, which weakens axial bonds and strengthens equatorial ones. In the context of investigated ligands, the primary binding site is the imidazole nitrogen, with secondary interactions from nitrile/pyridine nitrogens, a situation identical for all ligands and metals. The Jahn–Teller distortion results in a tetragonal geometry, with the imidazole nitrogen occupying an equatorial position (stronger binding) and other nitrogens in axial positions (weaker binding). This distortion enhances the covalent character of equatorial bonds, increasing Cu(II)’s affinity for ligands that optimize these interactions. The latter is most successfully achieved by investigated derivatives containing the –NH(CH_2_)_3_N(CH_3_)_2_ substituent due to its much higher flexibility over the other two more rigid substituents. This explains why Cu(II) affinities deviate from the trend observed for Zn(II) (d^10^, no Jahn–Teller effect) or Mg(II)/Ca(II) (no d-orbital contributions) and suggests that they are less dependent on intrinsic ligand nucleophilicity (proton affinity) and more influenced by covalent and geometric factors.

**Figure 6:**
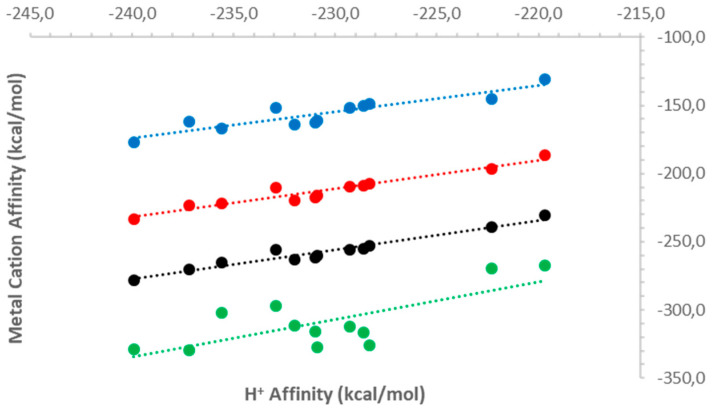
Correlation between proton and metal cation affinities in the gas phase (in kcal mol^−1^). Correlation coefficients (R^2^) are 0.83 (for Mg^2+^, in blue), 0.93 (for Ca^2+^, in red), 0.95 (for Zn^2+^, in black), and 0.54 (for Cu^2+^, in green).

To further illustrate this behavior, it is insightful to correlate the metal cation affinities of each ligand with their gas-phase basicities. The results in Figure 6 reveal a strong correlation, indicating that inherent ligand properties dominate binding, with steric and electronic effects playing a lesser role. This is particularly evident for Mg(II), Ca(II), and Zn(II), where gas-phase basicities and metal cation affinities show strong correlations, with R^2^ values of 0.83, 0.93, and 0.95, respectively. In contrast, for Cu(II) ions, the correlation is significantly weaker (R^2^ = 0.54), suggesting that gas-phase basicities poorly predict Cu(II) binding affinities. A similar conclusion was reached by Bickelhaupt et al. [26], who highlighted the challenges of expecting a simple linear correlation between proton and metal cation affinities. They argued that divalent transition metal cations, such as Cu(II), exhibit more complex interactions, involving both electrostatic and covalent contributions due to d-orbital effects, compared to main group metals like Mg(II) and Ca(II). In our case, the enhanced covalent character of Cu(II) complexes is evident in the computed NBO occupancies of the ligand’s nitrogen lone pairs involved in metal binding. For **6a**, the occupancy of imidazole and nitrile nitrogens in **6a**-Cu^2+^ is 0.94 and 0.97, respectively, indicating significant electron donation to the metal and strong covalent bonding. In contrast, analogous values for the imidazole nitrogen in Ca(II), Mg(II), and Zn(II) complexes with **6a** are 1.91, 1.89, and 1.79, and for the nitrile nitrogen, are 1.95, 1.94, and 1.89, respectively, indicating predominantly electrostatic interactions with minimal electron sharing. Similarly, Xu et al. [27] investigated proton and Cu(II) binding in humic acids, revealing varied affinities across functional groups. These studies highlight the difficulty of directly correlating gas-phase basicities with Cu(II) binding across nitrogen-rich ligands.

**Figure 7:**
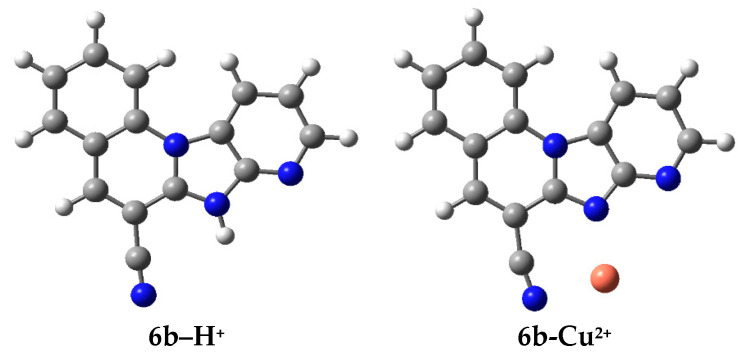
Computed geometries for 1:1 complexes of system **6b** with H^+^ and Cu^2+^ cations obtained with the M06–2X/Def2TZVPP DFT approach in the gas phase.

Regarding the geometry of 1:1 complexes, each cation most favorably binds to the unsaturated imidazole nitrogen, as consistently observed across all studied complexes, regardless of the cation or ligand. Typical geometries are presented in Figure 7, using **6b** as a reference with H^+^ and Cu^2+^ as representative cations. The proton, a small cation, binds locally to the imidazole nitrogen with minimal participation from other neighboring nitrogen sites, as evidenced by the N(imidazole)–H bond distance of 1.01 Å, compared to significantly larger non-bonded distances of 3.05 Å for N(nitrile)···H and 2.75 Å for N(pyridine)···H. In contrast, the Cu(II) ion, with a larger ionic radius, forms coordination bonds with both imidazole and nitrile nitrogens, as seen in **6b**-Cu^2+^, with bond distances of 2.11 Å for N(imidazole)–Cu(II) and 2.20 Å for N(nitrile)–Cu(II), and a non-bonded distance of 3.56 Å for N(pyridine)···Cu(II), indicating cooperative coordination primarily involving the imidazole and nitrile nitrogens.

Lastly, besides the most favorable binding at the unsaturated imidazole nitrogen for H^+^ and cooperative coordination with the nitrile nitrogen for metal dications, the investigated nitrogen-rich ligands contain other nitrogen sites capable of binding cations (Table S2). However, the annelated imidazole nitrogen is a significantly weaker binding site for protons, with Gibbs free energies of binding typically 20–30 kcal mol^−1^ lower than those for the non-annelated imidazole nitrogen. For metal dications, cations typically shift to more nucleophilic sites during geometry optimization. In contrast, the binding behavior of the pyridine nitrogen depends heavily on the regioisomer. The orientation of the pyridine nitrogen relative to the imidazole nitrogen is crucial for its nucleophilicity. In ***X*a** regioisomers, cation binding to the pyridine nitrogen is largely unfavorable, with affinities typically 10–20 kcal mol^−1^ lower than those for the unsaturated imidazole nitrogen. In ***X*b** regioisomers, however, the Gibbs free energies of binding for pyridine and imidazole nitrogens are comparable, although imidazole binding is typically 2–3 kcal mol^−1^ more favorable. This makes pyridine binding only marginally favorable. A notable exception is the parent compound **6b**, where protonation of the imidazole and pyridine nitrogens has nearly identical Gibbs free energies, with imidazole favored by only 0.1 kcal mol^−1^. This aligns with experimental uncertainties about the precise protonation site in imidazo[4,5-*b*]pyridines, where aqueous-phase measurements and DFT calculations support the higher basicity of the imidazole site [28].

Given the highly exergonic binding computed for Zn(II) and Cu(II) in their 1:1 complexes, we expanded our DFT analysis to include 2:1 (ligand/metal) complexes to further explore these trends (Table 3). Notably, all simulated 2:1 complexes are significantly more stable (by 20–50 kcal mol^−1^) than their 1:1 counterparts. This indicates their high thermodynamic favorability and supports their potential formation, particularly for Zn(II) and Cu(II), consistent with the experiments. As observed for 1:1 complexes, all ***X*b** regioisomers are more stable than their ***X*a** analogs due to the favorable orientation between the unsaturated imidazole and pyridine nitrogens, as already discussed. These stability differences are smallest for H^+^ at around 5 kcal mol^−1^, increase to 12–15 kcal mol^−1^ for Zn(II), and are largest for Cu(II), reaching up to 30 kcal mol^−1^ in favor of ***X*b** regioisomers.

**Figure 8:**
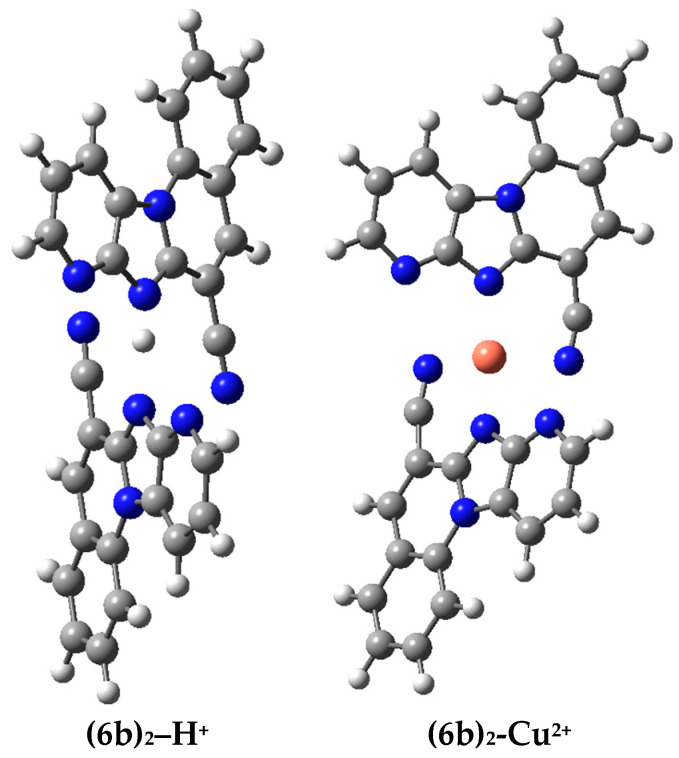
Computed geometries for 2:1 ligand/metal complexes for **6b** with H^+^ and Cu^2+^ cations, obtained using the M06–2X/Def2TZVPP DFT approach in the gas phase.

For proton 2:1 complexes, all exhibit asymmetric coordination, with H^+^ covalently bonded to one imidazole nitrogen and hydrogen bonded to another (Figure 8). For **6b**, these distances are N1(imidazole)–H = 1.08 Å and N2(imidazole)···H = 1.62 Å. In contrast, Cu(II) 2:1 complexes display a symmetric coordination environment, with each ligand contributing three nitrogen atoms (imidazole, nitrile, and pyridine) at identical N···Cu(II) distances of 1.95 Å, 2.25 Å, and 3.27 Å, respectively.

## 3. Materials and Methods

### 3.1. Mass Spectrometry Measurements

The solvents used for the spectroscopic measurements were HPLC or spectroscopic grade (Sigma-Aldrich Chemie GmbH, Steinheim, Germany) and were employed without further purification. The detailed study of the Cu (II) and Zn (II) ions with **1a**, **1b**, **2a**, and **2b** was studied by ESI-MS/MS. The spectra were obtained at the same conditions in positive ion (ESI+) mode. Certain fragmentations occurred at higher collision energies. The fragmentation pathways for all analyzed compounds were proposed based on MS/MS spectra of protonated molecular ions [M + H]^+^ and sodium adducts. The structures of complex ions were proposed based on the selection of signals of the appropriate isotopic distribution and the analysis of their MS/MS spectra. The latter were acquired on an Agilent 6550 Series Accurate-Mass-Quadrupole Time-of-Flight (Q-TOF) spectrometer equipped with an electrospray ionization interface operated in the ESI+ mode (Agilent Technologies, Palo Alto, CA, USA). The samples were prepared in methanol to a concentration of about 0.05 mg mL^−1^. The infusion into the mass spectrometer was performed via the Agilent 1290 Infinity II UHPLC system at a flow rate of 0.4 mL min^−1^ with a mobile phase consisting of 0.1% formic acid in water (A) and in acetonitrile (B) in the ratio A:B 50:50, optimized for solubility and ionization efficiency of metal–ligand complexes [29]. Nitrogen was used as an auxiliary (35 psi) and sheath gas (350 °C and 11 L min^−1^). The other parameters included capillary voltage = (VCap) 3000 V and 4000 V; nozzle voltage = 0 V; and a drying gas temperature of 200 °C. These parameters were selected based on their established efficacy in analyzing metal–ligand complexes with heterocyclic ligands [30]. The full mass spectra were acquired over the mass range *m*/*z* 100–3000. For data analysis, Mass Hunter Qualitative Analysis Navigator B 08.00 was used. A parent ion window of typically 2 amu (i.e., parent mass ± 1 amu) was chosen to perform further MS/MS experiments.

### 3.2. Computational Analysis

All geometries were optimized using the M06-2X/Def2TZVPP method in the gas phase, which combines a reliable DFT functional with a highly flexible triple-zeta basis set, both recommended for accurate geometries and energies involving these types of molecules [31]. Subsequent frequency analysis provided thermal corrections, ensuring that all reported computational data correspond to Gibbs free energies at 298.15 K and 1 atm, employing an experimental gas-phase Gibbs free energy of −6.28 kcal mol^−1^ for the proton [32]. This computational setup was chosen based on its success in modeling various organometallic complexes [33,34], and accurately reproducing thermodynamic and kinetic parameters of organic [35] and enzymatic [36] reactions. All cation affinities were computed as the difference in Gibbs free energy between the proton or metal adduct and its individual components. To address the moderate flexibility of the investigated systems, we evaluated multiple conformations for each case and report results corresponding to the most stable structures. All calculations were performed using the Gaussian 16 program package [37].

## 4. Conclusions

This study delivers a comprehensive analysis of the proton and metal dication affinities of twelve tetracyclic imidazo[4,5-*b*]pyridine-based organic molecules, significantly advancing the understanding of their coordination chemistry with biologically relevant metal ions, including Ca(II), Mg(II), Zn(II), and Cu(II). Through electrospray ionization mass spectrometry, we observed distinct coordination profiles, with Zn(II) and Cu(II) forming stable mononuclear and dinuclear complexes, often involving counterions such as acetate, formate, or hydroxide, which facilitated complex stability. A notable finding was the consistent reduction of Cu(II) to Cu(I) in the gas phase during ESI-MS experiments, a phenomenon attributed to the stabilization of charged complexes within methanol-based charged droplets, highlighting the intricate dynamics of metal–ligand interactions under these conditions. In contrast, Ca(II) and Mg(II) exhibited significantly lower binding affinities, aligning with Hard–Soft Acid–Base theory, which predicts weaker interactions between hard acids (Ca(II) and Mg(II)) and nitrogen-rich ligands compared to borderline (Zn(II)) or softer (Cu(II)) acids.

Complementing these experimental insights, DFT calculations provided a detailed molecular-level understanding of the electronic structures, coordination geometries, and thermodynamic stabilities of the complexes. The unsaturated imidazole nitrogen emerged as the most favorable binding site across all ligands and cations, driven by its higher basicity compared to pyridine or other nitrogen sites. Regioisomeric differences played a critical role, with ***X*b** derivatives consistently demonstrating higher binding affinities over their ***X*a** counterparts due to the favorable orientation of the pyridine nitrogen, which minimizes steric hindrance and enhances electronic stabilization. This effect was particularly pronounced for larger cations like Cu(II), where affinity differences between regioisomers reached up to 30 kcal mol^−1^ in 2:1 complexes. Substituent effects further modulated binding strength, with electron-donating groups such as piperidine and piperazine increasing basicity and metal coordination strength, particularly in disubstituted systems like **5a** and **5b**, which exhibited superbase-like properties in the gas phase.

The correlation analysis between proton and metal dication affinities revealed high linearity for Mg(II), Ca(II), and Zn(II) (R^2^ values of 0.83, 0.93, and 0.95, respectively), indicating that intrinsic ligand nucleophilicity largely governs these interactions. Yet, the lower correlation for Cu(II) (R^2^ = 0.54) underscores the complex interplay of electrostatic and covalent contributions, due to Cu(II)’s d^9^ configuration and stronger d-orbital overlap with nitrogen ligands. The 2:1 ligand–metal complexes were found to be significantly more stable than their 1:1 counterparts, particularly for Zn(II) and Cu(II), with computed Gibbs free energies suggesting high feasibility of formation, corroborating the prevalence of dinuclear complexes in ESI-MS spectra. Structural analysis of these 2:1 complexes revealed symmetrical coordination for Cu(II), involving both imidazole and nitrile nitrogens, while proton complexes exhibited asymmetrical hydrogen-bonding interactions.

These findings not only deepen the understanding of imidazo[4,5-*b*]pyridine derivatives as versatile ligands but also expand their functional repertoire beyond their well-documented pharmacological applications. The high affinity and selectivity for Zn(II) and Cu(II) position these molecules as promising candidates for modulating metalloenzyme activity, mitigating metal-ion toxicity, or designing selective chelators for therapeutic or industrial purposes. Conversely, the weaker interactions with Ca(II) and Mg(II) suggest potential applications in studying cellular signaling pathways where selective binding is required. The integrated experimental–computational approach established in this study provides a robust methodological framework for future investigations into heterocyclic ligand–metal interactions, paving the way for tailored ligand design in coordination chemistry, metal-ion sensing, catalysis, and beyond.

## Figures and Tables

**Table 1:** The assignation of singly charged signals in the TIC spectrum of **1a** and Zn(II) acetate in MeOH (Figure 2).

Sign	*m*/*z*	Assignation
A	923.1996	2(**1a**) + 2 Zn^2+^ + 2(HCOO^−^) + (OH^−^)
B	937.2177	2(**1a**) + 2 Zn^2+^ + (HCOO^−^) + (CH_3_COO^−^) + (OH^−^)
C	951.2163	2(**1a**) + 2 Zn^2+^ + 3(HCOO^−^)
D	956.2168	2(**1a**) + 2 Zn^2+^ + 2(HCOO^−^) + (CH_3_COO^−^)
E	979.2281	2(**1a**) + 2 Zn^2+^ + (HCOO^−^) + 2(CH_3_COO^−^)
F	993.2417	2(**1a**) + 2 Zn^2+^ + 3(CH_3_COO^−^)

**Table 2:** Computed Gibbs free energies for the selected cation affinities corresponding to the 1:1 complexes in the gas phase (in kcal mol^−1^). The values pertain to the most favorable imidazole nitrogen binding in all cases and are obtained using the M06–2X/Def2TZVPP DFT approach.

Ligand	H^+^	Ca^2+^	Mg^2+^	Zn^2+^	Cu^2+^	Ligand	H^+^	Ca^2+^	Mg^2+^	Zn^2+^	Cu^2+^
**1a**	−228.3	−148.7	−207.1	−253.0	−326.1	**1b**	−230.9	−161.4	−216.3	−260.0	−327.4
**2a**	−232.9	−151.4	−210.1	−256.0	−296.9	**2b**	−235.6	−166.8	−221.5	−265.1	−301.9
**3a**	−229.3	−151.4	−209.9	−256.2	−312.0	**3b**	−232.0	−164.2	−219.5	−263.4	−311.8
**4a**	−228.6	−150.2	−209.1	−255.3	−316.6	**4b**	−231.0	−162.4	−217.7	−261.7	−316.2
**5a**	−237.2	−162.1	−223.3	−270.0	−329.8	**5b**	−239.9	−176.7	−233.4	−277.9	−329.1
**6a**	−219.7	−130.7	−186.5	−230.8	−267.3	**6b**	−222.3	−145.1	−196.9	−239.0	−269.5

**Table 3:** Computed Gibbs free energies (in kcal mol^−1^) for the cation affinities of 2:1 ligand/metal complexes in the gas phase, obtained using the M06–2X/Def2TZVPP DFT approach. The values correspond to the most favorable imidazole nitrogen binding in all cases. Values in square brackets represent the increase in affinity relative to the matching 1:1 ligand/metal complex.

System	H^+^	Zn^2+^	Cu^2+^	System	H^+^	Zn^2+^	Cu^2+^
**1a**	−245.6 [−17.3]	−362.0 [−109.0]	−376.4 [−49.6]	**1b**	−250.6 [−19.7]	−374.5 [−114.6]	−381.3 [−53.9]
**2a**	−249.2 [−16.3]	−369.3 [−113.3]	−363.7 [−66.8]	**2b**	−254.1 [−18.6]	−384.3 [−119.2]	−392.8 [−92.4]
**3a**	−247.1 [−17.4]	−366.4 [−109.8]	−375.1 [−62.8]	**3b**	−252.2 [−20.2]	−378.0 [−114.5]	−378.7 [−67.0]
**4a**	−245.1 [−16.5]	−364.9 [−109.6]	−372.8 [−56.2]	**4b**	−249.8 [−18.4]	−376.4 [−114.3]	−376.9 [−61.2]
**5a**	−252.8 [−15.3]	−383.1 [−112.9]	−385.8 [−55.6]	**5b**	−258.7 [−18.3]	−396.0 [−117.7]	−389.9 [−60.3]
**6a**	−236.4 [−16.6]	−336.5 [−105.7]	−330.1 [−62.7]	**6b**	−241.8 [−19.5]	−351.1 [−112.1]	−360.1 [−90.6]

## Data Availability

Data is contained within the article or Supplementary Material.

## Supplementary Materials

The following supporting information can be downloaded at: https://www.mdpi.com/article/10.3390/molecules30132684/s1, Figure S1. Comparison of segments of TIC spectra obtained as a result of the interaction of **1a** and **1b** with Cu(II); Figure S2. The comparison of observed signals for **1a** and **1b** with Zn(II); Table S1. The list and assignation of the A–E signals observed in the TIC spectrum of the solution of **1b** with Zn(II); Figure S3. TIC spectrum of the MeOH solution of Cu(II) acetate and **2a**, revealing that only one type of mononuclear complex ion is formed; Figure S4. Part of the TIC spectrum of the MeOH solutions of Cu(II) acetate or Zn(II) acetate and **2a**; Table S2. Computed Gibbs free energies for the selected 1:1 metal–ligand cation affinities in the gas phase obtained via the M06–2X/Def2TZVPP approach; Cartesian coordinates and total Gibbs free energies for all complexes discussed in the text.

## References

[1] Jarmoni K., Misbahi K., Ferrières V. (2023). Imidazo[4,5-*b*]Pyridines: From Kinase Inhibitors to more Diversified Biological Properties. Curr. Med. Chem..

[2] Peytam F., Emamgholipour Z., Mousavi A., Moradi M., Foroumadi R., Firoozpour L., Divsalar F., Safavi M., Foroumadi A. (2023). Imidazopyridine-based kinase inhibitors as potential anticancer agents: A review. Bioorg. Chem..

[3] Krause M., Foks H., Gobis K. (2017). Pharmacological Potential and Synthetic Approaches of Imidazo[4,5-*b*]pyridine and Imidazo[4,5-*c*]pyridine Derivatives. Molecules.

[4] Marinescu M., Popa C.-V. (2022). Pyridine Compounds with Antimicrobial and Antiviral Activities. Int. J. Mol. Sci..

[5] Shelke R.N., Pansare D.N., Pawar C.D., Deshmukh A.K., Pawar R.P., Bembalkar S.R. (2017). Synthesis of 3*H*-imidazo[4,5-*b*]pyridine with evaluation of their anticancer and antimicrobial activity. Eur. J. Chem..

[6] Devi N., Singh D., Rawal R.K., Bariwal J., Singh V. (2016). Medicinal Attributes of Imidazo[1,2-*a*]pyridine Derivatives: An Update. Curr. Top. Med. Chem..

[7] Andreini C., Bertini I., Cavallaro G., Holliday G.L., Thornton J.M. (2008). Metal ions in biological catalysis: From enzyme databases to general principles. J. Biol. Inorg. Chem..

[8] Maret W., Sigel A., Sigel H., Sigel R. (2013). Zinc and Human Disease. Interrelations Between Essential Metal Ions and Human Diseases.

[9] Helmann J.D. (2025). Metals in Motion: Understanding Labile Metal Pools in Bacteria. Biochemistry.

[10] Chaudhran P.A., Sharma A. (2024). Progress in the Development of Imidazopyridine-Based Fluorescent Probes for Diverse Applications. Crit. Rev. Anal. Chem..

[11] O’HAir R.A.J. (2006). The 3D quadrupole ion trap mass spectrometer as a complete chemical laboratory for fundamental gas-phase studies of metal mediated chemistry. Chem. Commun..

[12] Neese F. (2012). The ORCA program system. WIREs Comput. Mol. Sci..

[13] Lončar B., Perin N., Mioč M., Boček I., Grgić L., Kralj M., Tomić S., Stojković M.R., Hranjec M. (2021). Novel amino substituted tetracyclic imidazo[4,5-*b*]pyridine derivatives: Design, synthesis, antiproliferative activity and DNA/RNA binding study. Eur. J. Med. Chem..

[14] Perin N., Lončar B., Kadić M., Kralj M., Starčević K., Carvalho R.A., Jarak I., Hranjec M. (2024). Design, Synthesis, Antitumor Activity and NMR-Based Metabolomics of Novel Amino Substituted Tetracyclic Imidazo[4,5-*b*]Pyridine Derivatives. ChemMedChem.

[15] McIndoe J.S., Vikse K.L. (2019). Assigning the ESI mass spectra of organometallic and coordination compounds. J. Mass Spectrom..

[16] Gianelli L., Amendola V., Fabbrizzi L., Pallavicini P., Mellerio G.G. (2001). Investigation of reduction of Cu(II) complexes in positive-ion mode electrospray mass spectrometry. Rapid Commun. Mass Spectrom..

[17] Lavanant H., Virelizier H., Hoppilliard Y. (1998). Reduction of copper(II) complexes by electron capture in an electrospray ionization source. J. Am. Soc. Mass Spectrom..

[18] Frański R., Klonowska-Wieszczycka K., Borowiak-Resterna A., Olszanowski A. (2006). Influence of solvent and counter ion on copper complexes with N-alkyl-pyridine-2-carboxamides as studied by electrospray ionization mass spectrometry. Eur. J. Mass Spectrom..

[19] Tshepelevitsh S., Kütt A., Lõkov M., Kaljurand I., Saame J., Heering A., Plieger P.G., Vianello R., Leito I. (2019). On the Basicity of Organic Bases in Different Media. Eur. J. Org. Chem..

[20] Hunter E.P.L., Lias S.G. (1998). Evaluated Gas Phase Basicities and Proton Affinities of Molecules: An Update. J. Phys. Chem. Ref. Data.

[21] Hansch C., Leo A., Taft R.W. (1991). A survey of Hammett substituent constants and resonance and field parameters. Chem. Rev..

[22] Maksić Z.B., Kovačević B., Vianello R. (2012). Advances in Determining the Absolute Proton Affinities of Neutral Organic Molecules in the Gas Phase and Their Interpretation: A Theoretical Account. Chem. Rev..

[23] Schwamm R.J., Vianello R., Maršavelski A., Garcia M.A., Claramunt R.M., Alkorta I., Saame J., Leito I., Fitchett C.M., Edwards A.J. (2016). ^15^N NMR Spectroscopy, X-ray and Neutron Diffraction, Quantum-Chemical Calculations, and UV/vis-Spectrophotometric Titrations as Complementary Techniques for the Analysis of Pyridine-Supported Bicyclic Guanidine Superbases. J. Org. Chem..

[24] Pearson R.G. (1963). Hard and Soft Acids and Bases. J. Am. Chem. Soc..

[25] Chakraborty D., Chattaraj P.K. (2021). Conceptual density functional theory based electronic structure principles. Chem. Sci..

[26] Boughlala Z., Guerra C.F., Bickelhaupt F.M. (2016). Alkali Metal Cation versus Proton and Methyl Cation Affinities: Structure and Bonding Mechanism. ChemistryOpen.

[27] Xu J., Koopal L.K., Fang L., Xiong J., Tan W. (2018). Proton and Copper Binding to Humic Acids Analyzed by XAFS Spectroscopy and Isothermal Titration Calorimetry. Environ. Sci. Technol..

[28] Boček I., Hranjec M., Vianello R. (2022). Imidazo[4,5-*b*]pyridine derived iminocoumarins as potential pH probes: Synthesis, spectroscopic and computational studies of their protonation equilibria. J. Mol. Liq..

[29] Cole R.B. (2010). Electrospray and MALDI Mass Spectrometry: Fundamentals, Instrumentation, Practicalities, and Biological Applications.

[30] Di Marco V.B., Bombi G.G. (2006). Electrospray Mass Spectrometry (ESI-MS) in the Study of Metal-Ligand Solution Equilibria. Mass Spectrom. Rev..

[31] Bursch M., Mewes J., Hansen A., Grimme S. (2022). Best-Practice DFT Protocols for Basic Molecular Computational Chemistry. Angew. Chem. Int. Ed..

[32] Linstrom P.J., Mallard W.G. (2013). NIST Chemistry WebBook, NIST Standard Reference Database Number 69.

[33] Glušič M., Stare J., Grdadolnik J., Vianello R. (2013). Binding of cadmium dication to glutathione facilitates cysteine –SH deprotonation: A computational DFT study. J. Inorg. Biochem..

[34] Pantalon Juraj N., Tandarić T., Tadić V., Perić B., Moreth D., Schatzschneider U., Brozović A., Vianello R., Kirin S.I. (2022). Tuning the coordination properties of chiral pseudopeptide bis(2-picolyl)amine and iminodiacetamide ligands in Zn(II) and Cu(II) complexes. Dalton Trans..

[35] Alizadeh A., Bagherinejad A., Kayanian J., Vianello R. (2022). An experimental and computational study of new spiro-barbituric acid pyrazoline scaffolds: Restricted rotation vs. annular tautomerism. New J. Chem..

[36] Vrban L., Vianello R. (2024). Prominent neuroprotective potential of indole-2-*N*-methylpropargylamine: High affinity and irreversible inhibition efficiency towards monoamine oxidase B revealed by computational scaffold analysis. Pharmaceuticals.

[37] Frisch M.J., Trucks G.W., Schlegel H.B., Scuseria G.E., Robb M.A., Cheeseman J.R., Scalmani G., Barone V., Petersson G.A., Nakatsuji H. (2016). Gaussian 16, Revision C.01.

