# Supplementary material for: Proton and Metal Dication Affinities of Tetracyclic Imidazo[4,5-b]Pyridine-Based Molecules: Insights from Mass Spectrometry and DFT Analysis
Source: Molecules. 2025 Jun 21;30(13):2684. doi: 10.3390/molecules30132684 (PMC12250928; doi:10.3390/molecules30132684)
Supplement: Supplementary file 1 [file molecules-30-02684-s001.zip › molecules-3680055-supplementary.pdf]

Proton and Metal Dication Affinities of Tetracyclic Imidazo[4,5-*b*]Pyridine-Based Molecules: Insights from Mass Spectrometry and DFT Analysis

Lucija Vrban,[1] Ingrid Ana Martinac,[2] Marijana Hranjec,[3] Marijana Pocrnić,[2] Nives Galić,[2] Renata Kobetić,[4,*] and Robert Vianello[1,*]

[1]Laboratory for the Computational Design and Synthesis of Functional Materials, Division of Organic Chemistry and Biochemistry, Ruđer Bošković Institute, Zagreb, Croatia

[2]Department of Chemistry, Faculty of Science, University of Zagreb, Zagreb, Croatia

[3]Department of Organic Chemistry, Faculty of Chemical Engineering and Technology, University of Zagreb, Zagreb, Croatia

[4]Laboratory for Biomolecular Interactions and Spectroscopy, Division of Organic Chemistry and Biochemistry, Ruđer Bošković Institute, Zagreb, Croatia

[*]Corresponding authors: Renata Kobetić (renata.kobetic@irb.hr); Robert Vianello (robert.vianello@irb.hr)

SUPPORTING INFORMATION

CONTENT	PAGE
**Figure S1.** Comparison of segments of TIC spectra obtained as a result of the interaction of **1a** and **1b** with Cu(II).	S2
**Figure S2.** The comparison of observed signals for **1a** and **1b** with Zn(II). Part of the TIC spectrum of a solution of **1a** and **1b** with Zn(II) acetate in methanol that shows the signals corresponding to the dinuclear complex ions. The detailed signal assignation is presented in Tables 1 and S1.	S2
**Table S1.** The list and assignation of the A–E signals (Figure S2) observed in the TIC spectrum of the solution of **1b** with Zn(II) acetate in MeOH.	S3
**Figure S3.** TIC spectrum of the MeOH solution of Cu(II) acetate and **2a** revealing that only one type of mononuclear complex ion is formed.	S3
**Figure S4.** Part of the TIC spectrum of the MeOH solutions of Cu(II) acetate or Zn(II) acetate and **2a**.	S4
**Table S2.** Computed Gibbs free energies for the selected 1:1 metal-ligand cation affinities in the gas phase obtained with the M06–2X/Def2TZVPP approach (in kcal mol^{-1}). Values shaded in gray mark the most favorable attachment site, corresponding to the imidazole nitrogen (N1) in all instances. In certain cases, the investigated cation migrated to another position on the molecule during geometry optimization, which is also indicated.	S5–S8
**Cartesian coordinates and total Gibbs Free Energies** for all complexes discussed in the text	S9–S198

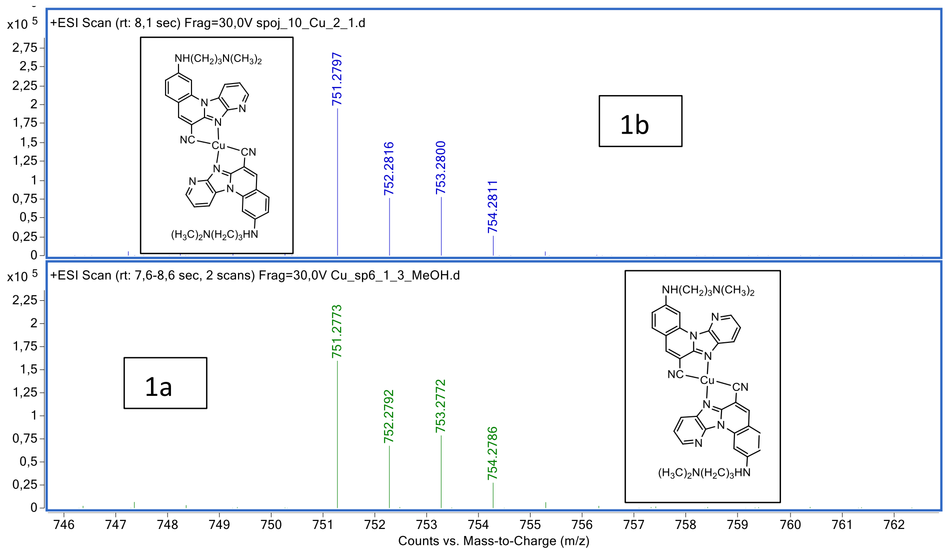

Figure S1. Comparison of segments of TIC spectra obtained as a result of the interaction of **1a** and **1b** with Cu(II).

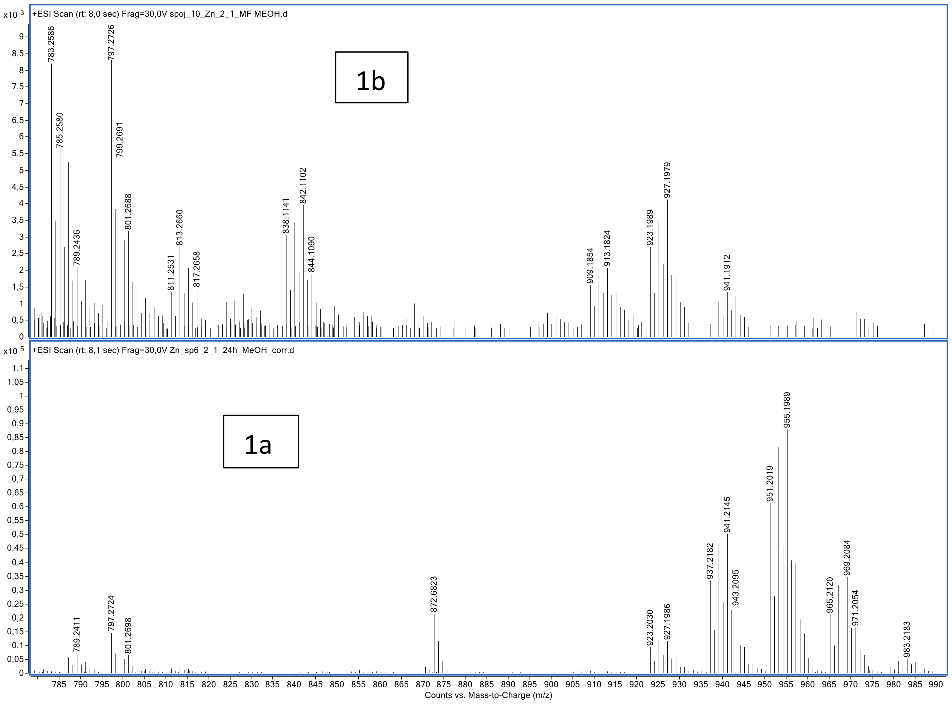

Figure S2. The comparison of observed signals for **1a** and **1b** with Zn(II). Part of the TIC spectrum of a solution of **1a** and **1b** with Zn(II) acetate in methanol that shows the signals corresponding to the dinuclear complex ions. The detailed signal assignation is presented in Tables 1 and S1.

Table S1. The list and assignation of the A–E signals (Figure S2) observed in the TIC spectrum of the solution of **1b** with Zn(II) acetate in MeOH.

Sign	m/z	Assignation
**A**	783.2586	(**1b-H**) + Zn^{2+} + (CH_3OH)
**B**	797.2726	(**1b-H**) + Zn^{2+} + ($HCOO^-$)
**C**	813.2660	(**1b-H**) + Zn^{2+} + 2 (CH_3OH)
**D**	909.1854	2 (**1b**) + 2 Zn^{2+} + (CH_3COO^-) + 2 (OH^-)
**E**	923.1989	2 (**1a**) + 2 Zn^{2+} + 2 ($HCOO^-$) + (OH^-)

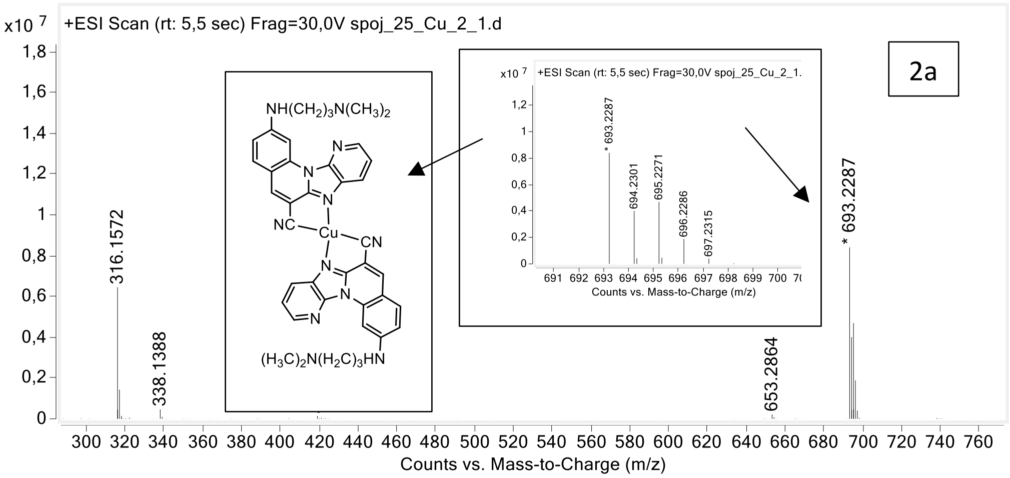

Figure S3. TIC spectrum of the MeOH solution of Cu(II) acetate and **2a** revealing that only one type of mononuclear complex ion is formed.

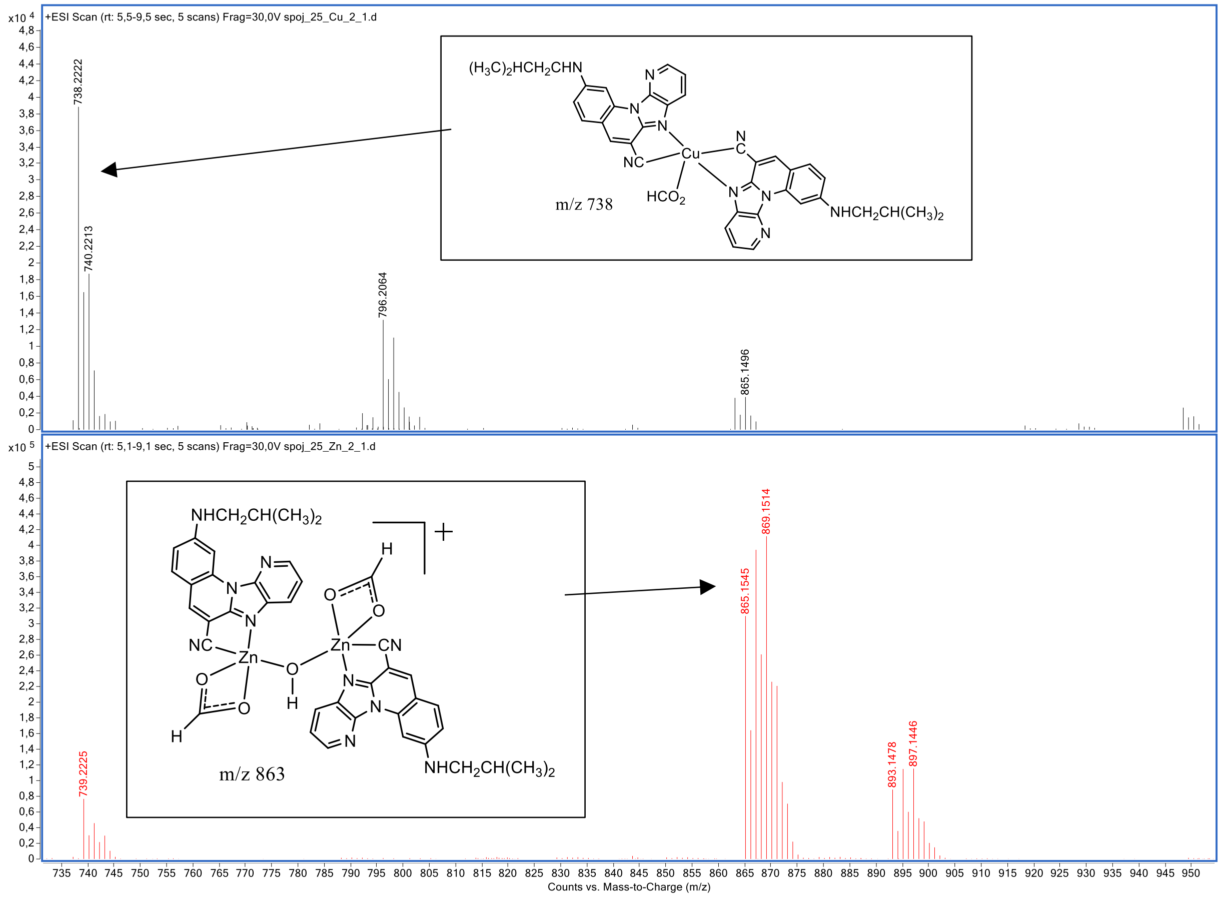

Figure S4. Part of the TIC spectrum of the MeOH solutions of Cu(II) acetate or Zn(II) acetate and **2a**.

Table S2. Computed Gibbs free energies for the selected 1:1 metal-ligand cation affinities in the gas phase obtained with the M06–2X/Def2TZVPP approach (in kcal mol^{-1}). Values shaded in gray mark the most favorable attachment site, corresponding to the imidazole nitrogen (N1) in all instances. In certain cases, the investigated cation migrated to another position on the molecule during geometry optimization, which is also indicated.

Molecule	Site	H^+	Ca^{2+}	Mg^{2+}	Zn^{2+}	Cu^{2+}
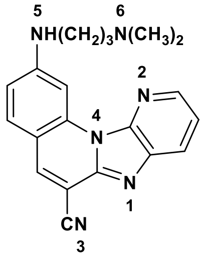	N1	−228.3	−148.7	−207.1	−253.0	−326.1
	N2	−210.3	−146.0[1]	−167.6	−200.0	−293.8
	N3	−204.4	moved to N1	moved to N1	−223.2	−311.3
	N4	−171.0	moved to N1	moved to N2	−203.7	moved to N2
	N5	−204.7	−146.0[1]	moved to N2	−223.8	−294.6
**1a**	N6	−213.2	−146.0[1]	−195.9	−213.8	−296.1

[1]Cation positioned between N6, N5, and N2.

Molecule	Site	H^+	Ca^{2+}	Mg^{2+}	Zn^{2+}	Cu^{2+}
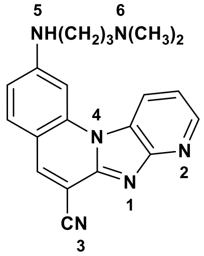	N1	−230.9	−161.4	−216.3	−260.0	−327.4
	N2	−228.7	moved to N1	−204.1	−244.2	−320.2
	N3	−203.9	moved to N1	moved to N1	moved to N1	−309.1
	N4	−170.0	moved to N1	−132.7	−195.5	moved to N1
	N5	−199.0	−122.4[1]	−139.6	−212.7	−259.0
**1b**	N6	−210.6	−122.4[1]	−135.7	−207.3	−289.9

[1]Cation positioned between N6 and N5.

Molecule	Site	H^+	Ca^{2+}	Mg^{2+}	Zn^{2+}	Cu^{2+}
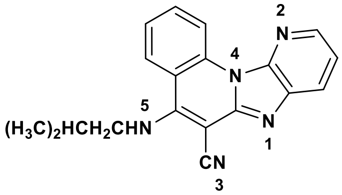	N1	−232.9	−151.4	−210.1	−256.0	−296.9
	N2	−213.6	−102.1	−159.4	−214.9	−277.2
	N3	−205.8	moved to N1	moved to N1	moved to N1	−292.5
	N4	−187.7	moved to N1	moved to N1	−202.2	moved to N2
**2a**	N5	−203.7	−118.9[1]	−179.3[1]	−226.4[1]	−277.1[1]

[1]Cation positioned between N5 and N3.

Molecule	Site	H^+	Ca^{2+}	Mg^{2+}	Zn^{2+}	Cu^{2+}
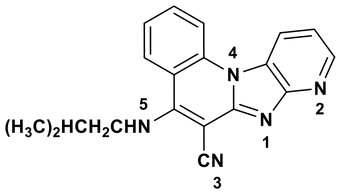	N1	−235.6	−166.8	−221.5	−265.1	−301.9
	N2	−232.6	moved to N1	−210.8	−249.7	−296.1
	N3	−205.6	moved to N1	moved to N1	moved to N1	−291.6
	N4	−176.1	moved to N1	moved to N1	−196.4	moved to N1
**2b**	N5	−202.5	−117.7[1]	−177.9[1]	−224.9	−274.4[1]

[1]Cation positioned between N5 and N3.

Table S2. Continued

Molecule	Site	H^+	Ca^{2+}	Mg^{2+}	Zn^{2+}	Cu^{2+}
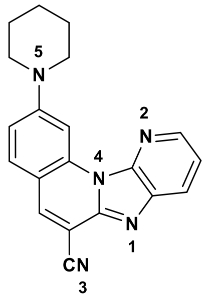	**N1**	−229.3	−151.4	−209.9	−256.2	−312.0
	**N2**	−210.3	−118.1(1)	−175.3(1)	−234.1	−282.6
	**N3**	−213.9	moved to N1	−166.2	−227.8	−304.8
	**N4**	−171.7	moved to N1	moved to N2	−206.4	−278.2
**3a**	**N5**	−205.9	−118.1(1)	−175.3(1)	−229.1(1)	−269.4

(1)Cation positioned between N5 and N2.

Molecule	Site	H^+	Ca^{2+}	Mg^{2+}	Zn^{2+}	Cu^{2+}
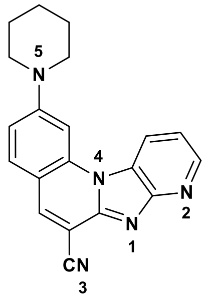	**N1**	−232.0	−164.2	−219.5	−263.4	−311.8
	**N2**	−229.4	moved to N1	−206.6	−245.5	−307.9
	**N3**	−205.6	moved to N1	moved to N1	−224.5	moved to N1
	**N4**	−171.0	moved to N1	−138.8	−198.3	moved to N1
**3b**	**N5**	−209.2	−87.7	−137.6	−188.1	−260.9

Molecule	Site	H^+	Ca^{2+}	Mg^{2+}	Zn^{2+}	Cu^{2+}
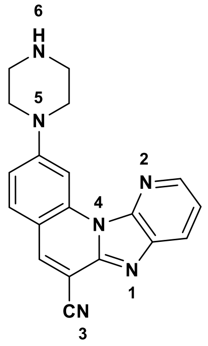	**N1**	−228.6	−150.2	−209.1	−255.3	−316.6
	**N2**	−210.3	−118.1(1)	−177.0	−233.1	−282.5
	**N3**	−205.3	moved to N1	moved to N1	−226.6	−306.9
	**N4**	−171.2	moved to N1	moved to N2	−205.7	−278.5
	**N5**	−212.7	−118.1(1)	−176.5(1)	−232.0(1)	−269.9
**4a**	**N6**	−210.5	−131.8(2)	−141.8	−210.1	−286.4

(1)Cation positioned between N5 and N2. (2)Cation positioned between N6, N5, and N2.

Table S2. Continued

Molecule	Site	H^+	Ca^{2+}	Mg^{2+}	Zn^{2+}	Cu^{2+}
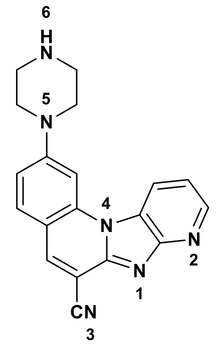	**N1**	−231.0	−162.4	−217.7	−261.7	−316.2
	**N2**	−228.7	moved to N1	−205.1	−244.6	−310.8
	**N3**	−204.5	moved to N1	moved to N1	−223.5	−299.8
	**N4**	−188.0	moved to N1	−138.0	−196.7	moved to N1
	**N5**	−207.3	−88.0	−140.2	−190.6	−264.6
**4b**	**N6**	−206.3	−105.6	−133.2	−201.9	−278.5

Molecule	Site	H^+	Ca^{2+}	Mg^{2+}	Zn^{2+}	Cu^{2+}
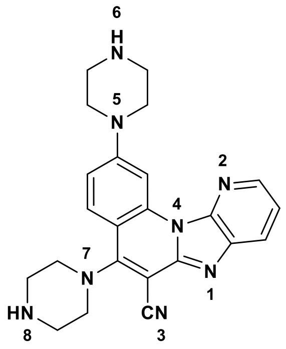	**N1**	−237.2	−162.1	−223.3	−270.0	−329.8
	**N2**	−217.0	−130.8	−191.3[1]	−246.9[1]	−302.4
	**N3**	−211.7	−123.5	moved to N1	moved to N1	−314.7
	**N4**	−182.8	moved to N1	−153.0	−221.5	moved to N2
	**N5**	−215.9	−130.6[1]	−191.3[1]	−246.9[1]	−296.5
	**N6**	−213.3	−141.3[2]	−152.0	−224.8	−306.1
	**N7**	−208.1	−135.8[3]	−200.6[3]	−253.2[3]	−316.1[3]
**5a**	**N8**	−220.1	−132.2	−157.9	−229.2	−307.5

[1]Cation positioned between N5 and N2. [2]Cation positioned between N6, N5, and N2. [3]Cation positioned between N7 and N3.

Molecule	Site	H^+	Ca^{2+}	Mg^{2+}	Zn^{2+}	Cu^{2+}
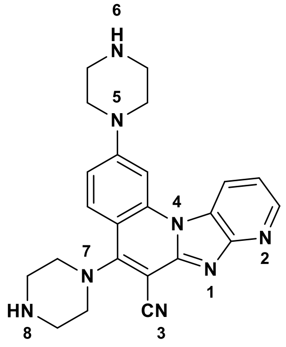	**N1**	−239.9	−176.7	−233.9	−277.9	−329.1
	**N2**	−182.6	moved to N1	−221.2	−260.7	−322.7
	**N3**	−219.2	−122.7	moved to N1	moved to N1	moved to N1
	**N4**	−236.1	moved to N1	−155.7	−215.6	moved to N1
	**N5**	−210.4	−101.5	−156.2	−209.1	−290.1
	**N6**	−208.6	−112.6	−145.3	−218.6	−299.9
	**N7**	−210.5	−152.7[1]	−198.3[1]	−250.9[1]	−307.9[1]
**5b**	**N8**	−205.8	−128.2[2]	−154.6	−224.4	−302.4

[1]Cation positioned between N7 and N3. [2]Cation positioned between N8 and N7.

Table S2. Continued

Molecule	Site	H^+	Ca^{2+}	Mg^{2+}	Zn^{2+}	Cu^{2+}
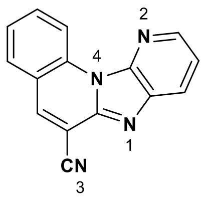	**N1**	–219.7	–130.7	–186.5	–230.8	–267.3
	**N2**	–205.2	–90.1	–146.5	–201.2	–243.5
	**N3**	–193.0	moved to N1	moved to N1	moved to N1	moved to N1
**6a**	**N4**	–163.5	moved to N1	moved to N1	moved to N1	moved to N1

Molecule	Site	H^+	Ca^{2+}	Mg^{2+}	Zn^{2+}	Cu^{2+}
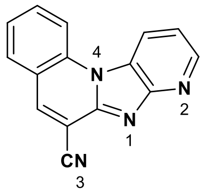	**N1**	–222.3	–145.1	–196.9	–239.0	–269.5
	**N2**	–222.2	moved to N1	–189.0	–226.9	–264.2
	**N3**	–192.6	moved to N1	moved to N1	moved to N1	moved to N1
**6b**	**N4**	–162.2	moved to N1	–102.3	moved to N1	moved to N1

Ligand	Description	Total Gibbs Free energy (M06-2X/Def2TZVPP)
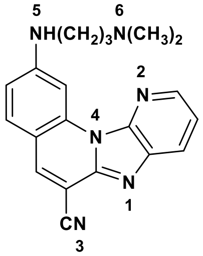<br>**1a**	H$^+$ bound to N1	-1102,697761 a.u.

CARTESIAN COORDINATES

Center Number	Atomic Number	Atomic Type	Coordinates (Angstroms) X	Y	Z
1	7	0	2.492680	0.109001	-0.005204
2	6	0	1.280381	-0.594084	-0.000635
3	6	0	3.692743	-0.524011	0.016595
4	7	0	4.681354	0.387068	0.005545
5	6	0	4.130401	1.654343	-0.024701
6	6	0	2.743759	1.496786	-0.031729
7	6	0	3.802116	-1.923515	0.045212
8	7	0	1.860833	2.467090	-0.058289
9	6	0	4.667475	2.929533	-0.047246
10	6	0	2.376041	3.694412	-0.080038
11	6	0	3.747776	3.963502	-0.075557
12	1	0	1.664349	4.508507	-0.102277
13	1	0	4.083951	4.989601	-0.094538
14	1	0	5.733180	3.108886	-0.043142
15	6	0	1.381695	-2.015096	0.027535
16	6	0	2.629065	-2.646582	0.049807
17	1	0	2.674582	-3.727752	0.071078
18	6	0	0.066383	0.048176	-0.022285
19	6	0	0.173260	-2.752227	0.031026
20	6	0	-1.037893	-2.136540	0.008980
21	6	0	-1.119960	-0.713353	-0.017238
22	1	0	0.231046	-3.832648	0.051173
23	1	0	-1.942747	-2.724726	0.010633
24	7	0	-2.307348	-0.097968	-0.036069
25	1	0	0.019325	1.125154	-0.043236
26	1	0	-2.313946	0.909316	-0.068222
27	6	0	-3.602909	-0.762608	-0.049301
28	6	0	-4.717287	0.269064	-0.052577
29	1	0	-3.677082	-1.398657	-0.935848
30	1	0	-3.690084	-1.405497	0.831124
31	6	0	-6.085215	-0.401178	-0.038350
32	1	0	-4.598222	0.915265	0.819610
33	1	0	-4.644716	0.893986	-0.946006
34	7	0	-7.168336	0.530956	-0.276864
35	1	0	-6.115647	-1.152333	-0.831651
36	1	0	-6.220009	-0.939549	0.918125
37	6	0	-8.416512	-0.184411	-0.480825
38	6	0	-7.309046	1.494785	0.799870
39	1	0	-9.210467	0.524740	-0.708605
40	1	0	-8.317738	-0.869806	-1.321782
41	1	0	-8.715427	-0.763189	0.407289
42	1	0	-8.157853	2.144035	0.592801
43	1	0	-7.478047	1.007361	1.774102
44	1	0	-6.423908	2.124084	0.875711
45	6	0	5.113486	-2.479177	0.066358
46	7	0	6.204243	-2.836709	0.081865
47	1	0	5.660916	0.140248	0.017354

Ligand	Description	Total Gibbs Free energy (M06-2X/Def2TZVPP)
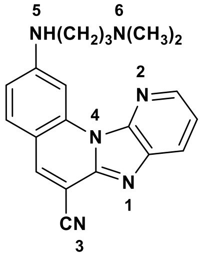 1a	Ca^{2+} bound to N1	-1779,47150340 a.u.

CARTESIAN COORDINATES

Center Number	Atomic Number	Atomic Type	Coordinates (Angstroms) X	Y	Z
1	6	0	1.668471	3.709318	-0.084155
2	7	0	1.168951	2.475903	-0.060956
3	6	0	2.063186	1.516173	-0.036662
4	6	0	3.452067	1.667240	-0.032995
5	6	0	3.960698	2.957815	-0.057270
6	6	0	3.038468	3.989375	-0.083196
7	7	0	1.826968	0.130321	-0.008768
8	6	0	3.046672	-0.467423	0.010221
9	7	0	4.067072	0.412384	-0.003714
10	6	0	3.134280	-1.885347	0.041349
11	6	0	1.962712	-2.642646	0.049021
12	6	0	0.730848	-2.022379	0.027913
13	6	0	0.619483	-0.588227	-0.001059
14	6	0	-0.481505	-2.773445	0.033690
15	6	0	-1.692122	-2.172095	0.013469
16	6	0	-1.782649	-0.741900	-0.013119
17	6	0	-0.591739	0.036639	-0.020342
18	7	0	-7.813079	0.534592	-0.267687
19	6	0	-6.745645	-0.415789	-0.043609
20	6	0	-5.371161	0.244381	-0.043839
21	6	0	-4.267145	-0.797766	-0.039065
22	7	0	-2.959493	-0.139244	-0.031189
23	6	0	4.460801	-2.316800	0.060425
24	1	0	0.949308	4.516867	-0.104489
25	1	0	5.022912	3.172596	-0.056285
26	1	0	3.366900	5.018087	-0.102846
27	1	0	2.019935	-3.723182	0.071657
28	1	0	-0.413075	-3.853378	0.054335
29	1	0	-2.594623	-2.763840	0.016929
30	1	0	-0.653081	1.113456	-0.041860
31	6	0	-9.065393	-0.158146	-0.528970
32	6	0	-7.968571	1.458113	0.843101
33	1	0	-6.780337	-1.150489	-0.852046
34	1	0	-6.885775	-0.971794	0.901958
35	1	0	-5.289297	0.872193	-0.934437
36	1	0	-5.254236	0.884824	0.832893
37	1	0	-4.348302	-1.435487	0.844419
38	1	0	-4.337880	-1.433010	-0.925201
39	7	0	5.619898	-2.379026	0.070328
40	1	0	-2.971330	0.870682	-0.056700
41	1	0	-8.955600	-0.808000	-1.396160
42	1	0	-9.844874	0.570097	-0.744291
43	1	0	-9.390471	-0.768550	0.327324
44	1	0	-7.079508	2.075186	0.964230
45	1	0	-8.804527	2.124841	0.641183
46	1	0	-8.166686	0.935600	1.792757
47	20	0	6.300390	-0.203688	0.015554

Ligand	Description	Total Gibbs Free energy (M06-2X/Def2TZVPP)
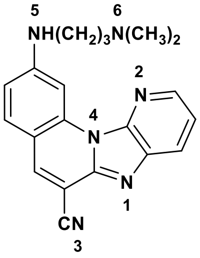 **1a**	Cu^{2+} bound to N1	-2742,2893098 a.u.

CARTESIAN COORDINATES

Center Number	Atomic Number	Atomic Type	Coordinates (Angstroms) X	Y	Z
1	6	0	1.449118	3.774727	-0.067710
2	7	0	0.936188	2.548271	-0.049767
3	6	0	1.831617	1.582673	-0.029978
4	6	0	3.223484	1.732527	-0.026714
5	6	0	3.743299	3.020601	-0.045737
6	6	0	2.822930	4.050151	-0.066439
7	7	0	1.599355	0.200816	-0.008130
8	6	0	2.835982	-0.386014	0.006330
9	7	0	3.828149	0.490171	-0.003951
10	6	0	2.938832	-1.815647	0.029599
11	6	0	1.801920	-2.566976	0.037006
12	6	0	0.529661	-1.947200	0.022930
13	6	0	0.412077	-0.533750	-0.000126
14	6	0	-0.656461	-2.702474	0.028397
15	6	0	-1.886168	-2.104384	0.013824
16	6	0	-1.985092	-0.694518	-0.002821
17	6	0	-0.822750	0.084858	-0.012929
18	7	0	-8.134738	0.268707	0.002973
19	6	0	-6.933700	-0.538487	-0.102155
20	6	0	-5.617628	0.212202	0.002754
21	6	0	-4.457994	-0.775073	-0.098919
22	7	0	-3.201239	-0.073835	0.005325
23	6	0	4.278032	-2.298609	0.042502
24	1	0	0.737356	4.589253	-0.084031
25	1	0	4.808784	3.206530	-0.044389
26	1	0	3.151702	5.078838	-0.081936
27	1	0	1.863332	-3.647275	0.054145
28	1	0	-0.583308	-3.782065	0.040894
29	1	0	-2.773964	-2.718384	0.010805
30	1	0	-0.879758	1.162909	-0.025644
31	6	0	-9.409240	-0.338733	-0.296131
32	6	0	-8.110732	1.622473	0.496629
33	1	0	-7.010627	-1.087216	-1.046513
34	1	0	-7.018443	-1.297515	0.690387
35	1	0	-5.543411	0.944170	-0.805092
36	1	0	-5.549779	0.743954	0.953297
37	1	0	-4.535895	-1.503558	0.713402
38	1	0	-4.525321	-1.329625	-1.042948
39	7	0	5.420232	-2.466204	0.049655
40	1	0	-3.202311	0.919659	-0.157050
41	1	0	-9.282815	-1.388904	-0.538451
42	1	0	-9.857942	0.194845	-1.140455
43	1	0	-10.072158	-0.214492	0.563956
44	1	0	-7.366026	2.202960	-0.048227
45	1	0	-9.095574	2.067064	0.394312
46	1	0	-7.820085	1.606504	1.553324
47	29	0	5.706810	-0.303214	0.016745

Ligand	Description	Total Gibbs Free energy (M06-2X/Def2TZVPP)
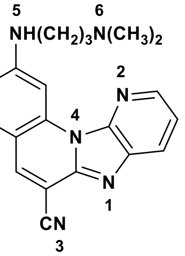 **1a**	Mg^{2+} bound to N1	-1301,8763518 a.u.

CARTESIAN COORDINATES

Center Number	Atomic Number	Atomic Type	Coordinates (Angstroms) X	Y	Z
1	6	0	1.954378	3.695949	-0.081835
2	7	0	1.456189	2.461658	-0.057571
3	6	0	2.351631	1.504434	-0.034090
4	6	0	3.737421	1.661535	-0.032139
5	6	0	4.249645	2.949144	-0.057552
6	6	0	3.324327	3.977945	-0.082770
7	7	0	2.113408	0.114660	-0.006050
8	6	0	3.325425	-0.488101	0.011013
9	7	0	4.345635	0.401688	-0.003503
10	6	0	3.413185	-1.908113	0.040168
11	6	0	2.233999	-2.664558	0.049503
12	6	0	1.010381	-2.039409	0.031476
13	6	0	0.902436	-0.601093	0.003014
14	6	0	-0.207339	-2.788055	0.039433
15	6	0	-1.414133	-2.183566	0.021184
16	6	0	-1.500030	-0.750798	-0.006044
17	6	0	-0.304471	0.026375	-0.014536
18	7	0	-7.521393	0.552242	-0.273690
19	6	0	-6.460670	-0.403201	-0.042679
20	6	0	-5.082727	0.250659	-0.042637
21	6	0	-3.985095	-0.797953	-0.030659
22	7	0	-2.671754	-0.146160	-0.023589
23	6	0	4.744056	-2.312368	0.053571
24	1	0	1.233991	4.502409	-0.101685
25	1	0	5.312023	3.158754	-0.058260
26	1	0	3.651409	5.007105	-0.103505
27	1	0	2.289093	-3.745110	0.070823
28	1	0	-0.141343	-3.868109	0.060268
29	1	0	-2.319120	-2.771575	0.026633
30	1	0	-0.363788	1.103417	-0.035936
31	6	0	-8.776351	-0.133941	-0.541362
32	6	0	-7.677834	1.478652	0.834801
33	1	0	-6.496918	-1.141037	-0.848226
34	1	0	-6.605325	-0.954553	0.904936
35	1	0	-4.995936	0.873520	-0.936231
36	1	0	-4.964903	0.894357	0.831610
37	1	0	-4.069198	-1.430680	0.855910
38	1	0	-4.055225	-1.436138	-0.914455
39	7	0	5.908608	-2.282954	0.058292
40	1	0	-2.680700	0.864514	-0.048633
41	1	0	-8.665336	-0.785322	-1.407217
42	1	0	-9.550114	0.598654	-0.762095
43	1	0	-9.109803	-0.741153	0.313805
44	1	0	-6.786128	2.091222	0.959870
45	1	0	-8.508698	2.149761	0.626849
46	1	0	-7.884423	0.959008	1.784088
47	12	0	6.188848	-0.323183	0.018090

Ligand	Description	Total Gibbs Free energy (M06-2X/Def2TZVPP)
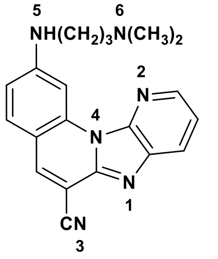 **1a**	Zn^{2+} bound to N1	-2881,1240340 a.u.

CARTESIAN COORDINATES

Center Number	Atomic Number	Atomic Type	Coordinates (Angstroms) X	Y	Z
1	6	0	1.394425	3.740750	-0.083128
2	7	0	0.913681	2.499269	-0.058965
3	6	0	1.820996	1.554575	-0.036128
4	6	0	3.203093	1.737188	-0.034947
5	6	0	3.701559	3.028747	-0.060011
6	6	0	2.760458	4.042981	-0.084497
7	7	0	1.599936	0.159239	-0.008250
8	6	0	2.815658	-0.433210	0.008219
9	7	0	3.815703	0.482962	-0.006936
10	6	0	2.924108	-1.852227	0.037109
11	6	0	1.747397	-2.619513	0.047074
12	6	0	0.519948	-2.007520	0.029750
13	6	0	0.395528	-0.569022	0.001411
14	6	0	-0.691094	-2.770049	0.038422
15	6	0	-1.903410	-2.178875	0.020983
16	6	0	-2.004918	-0.745949	-0.006153
17	6	0	-0.816764	0.045172	-0.015374
18	7	0	-8.034523	0.511473	-0.270975
19	6	0	-6.968925	-0.438863	-0.042571
20	6	0	-5.595071	0.223948	-0.041077
21	6	0	-4.490252	-0.817080	-0.028888
22	7	0	-3.181248	-0.154335	-0.022982
23	6	0	4.256301	-2.254168	0.050089
24	1	0	0.662387	4.536644	-0.102514
25	1	0	4.761939	3.246427	-0.060785
26	1	0	3.072231	5.076911	-0.104999
27	1	0	1.813886	-3.699396	0.068392
28	1	0	-0.613020	-3.849296	0.059172
29	1	0	-2.802362	-2.776192	0.027012
30	1	0	-0.888546	1.121445	-0.036681
31	6	0	-9.284928	-0.180177	-0.546769
32	6	0	-8.199750	1.430610	0.842451
33	1	0	-7.000642	-1.174496	-0.850311
34	1	0	-7.109916	-0.993786	0.903487
35	1	0	-5.512042	0.847694	-0.934422
36	1	0	-5.482361	0.867957	0.833632
37	1	0	-4.567886	-1.449578	0.858327
38	1	0	-4.554693	-1.455992	-0.912483
39	7	0	5.424704	-2.217590	0.053580
40	1	0	-3.200503	0.856578	-0.047418
41	1	0	-9.167656	-0.825485	-1.416316
42	1	0	-10.061886	0.549599	-0.765453
43	1	0	-9.617698	-0.794276	0.303630
44	1	0	-7.311798	2.047212	0.974466
45	1	0	-9.033210	2.098691	0.635284
46	1	0	-8.407205	0.904382	1.787833
47	30	0	5.591568	-0.257536	0.013535

Ligand	Description	Total Gibbs Free energy (M06-2X/Def2TZVPP)
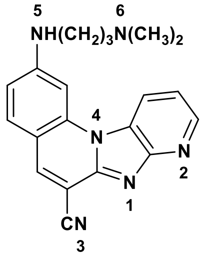 **1b**	H^+ bound to N1	-1102.692083 a.u.

CARTESIAN COORDINATES

Center Number	Atomic Number	Atomic Type	Coordinates (Angstroms) X	Y	Z
1	7	0	2.469897	0.108329	-0.007823
2	6	0	1.253878	-0.589707	-0.008652
3	6	0	3.661560	-0.553013	0.026611
4	7	0	4.665256	0.331286	0.026600
5	6	0	4.143267	1.609793	-0.012587
6	6	0	2.748591	1.490873	-0.036910
7	6	0	3.750751	-1.955628	0.052539
8	6	0	2.007916	2.665017	-0.088698
9	7	0	4.847168	2.715379	-0.027545
10	6	0	2.731544	3.845803	-0.105245
11	6	0	4.127844	3.831047	-0.072664
12	1	0	2.212726	4.791887	-0.145391
13	1	0	4.684370	4.758806	-0.085462
14	6	0	1.335288	-2.010071	0.010473
15	6	0	2.573594	-2.662826	0.040335
16	1	0	2.602293	-3.744525	0.056189
17	6	0	0.034950	0.049195	-0.024317
18	6	0	0.122307	-2.738551	0.003590
19	6	0	-1.086862	-2.119867	-0.016645
20	6	0	-1.159773	-0.697919	-0.027563
21	1	0	0.176004	-3.819310	0.015413
22	1	0	-1.994407	-2.703506	-0.022793
23	7	0	-2.344771	-0.075490	-0.038571
24	1	0	-0.037803	1.121122	-0.028769
25	1	0	-2.353023	0.931619	-0.060876
26	6	0	-3.643286	-0.736329	-0.057655
27	6	0	-4.753675	0.299519	-0.045143
28	1	0	-3.721145	-1.360226	-0.952339
29	1	0	-3.730309	-1.389718	0.814829
30	6	0	-6.124455	-0.365444	-0.041126
31	1	0	-4.632459	0.932049	0.836782
32	1	0	-4.679487	0.937323	-0.929400
33	1	0	0.933669	2.703346	-0.119467
34	7	0	-7.203082	0.575817	-0.262317
35	1	0	-6.158416	-1.102119	-0.847687
36	1	0	-6.260394	-0.920154	0.905726
37	6	0	-8.453768	-0.129879	-0.485684
38	6	0	-7.343441	1.516467	0.834932
39	1	0	-9.243972	0.587560	-0.699908
40	1	0	-8.355353	-0.796659	-1.341470
41	1	0	-8.757611	-0.726818	0.388483
42	1	0	-8.188717	2.173736	0.639263
43	1	0	-7.517825	1.008626	1.797626
44	1	0	-6.456046	2.140414	0.927592
45	6	0	5.052359	-2.535144	0.085832
46	7	0	6.131007	-2.926099	0.111961
47	1	0	5.648049	0.091988	0.046974

Ligand	Description	Total Gibbs Free energy (M06-2X/Def2TZVPP)
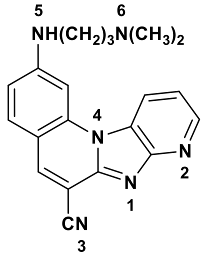 **1b**	Ca^{2+} bound to N1	-1779,4820325 a.u.

CARTESIAN COORDINATES

Center Number	Atomic Number	Atomic Type	Coordinates (Angstroms) X	Y	Z
1	6	0	2.222131	3.808349	-0.086568
2	6	0	1.426522	2.669954	-0.064290
3	6	0	2.101322	1.457335	-0.034825
4	6	0	3.500630	1.496264	-0.030635
5	7	0	4.274325	2.564926	-0.051817
6	6	0	3.617005	3.724454	-0.079840
7	7	0	1.780000	0.081747	-0.005817
8	6	0	2.967397	-0.588788	0.013215
9	7	0	4.019131	0.224967	-0.000873
10	6	0	3.039372	-2.001907	0.043794
11	6	0	1.855080	-2.722164	0.052203
12	6	0	0.639868	-2.050742	0.032103
13	6	0	0.561358	-0.614359	0.003452
14	6	0	-0.589270	-2.769859	0.038750
15	6	0	-1.789197	-2.144995	0.019693
16	6	0	-1.853579	-0.716065	-0.006399
17	6	0	-0.644022	0.028610	-0.014182
18	7	0	-7.866807	0.641434	-0.273302
19	6	0	-6.812008	-0.322870	-0.049243
20	6	0	-5.429503	0.320703	-0.041736
21	6	0	-4.338353	-0.734807	-0.033153
22	7	0	-3.022355	-0.092328	-0.023250
23	6	0	4.395220	-2.383154	0.058321
24	1	0	1.758355	4.783274	-0.109823
25	1	0	4.216366	4.624537	-0.097704
26	1	0	1.867491	-3.803999	0.074134
27	1	0	-0.545416	-3.851099	0.059217
28	1	0	-2.702281	-2.719953	0.024055
29	1	0	-0.704136	1.103504	-0.035078
30	6	0	-9.124985	-0.034662	-0.550643
31	6	0	-8.020924	1.556514	0.844724
32	1	0	-6.852069	-1.053434	-0.861108
33	1	0	-6.962330	-0.881364	0.893191
34	1	0	-5.336454	0.948369	-0.931429
35	1	0	-5.309488	0.958694	0.836453
36	1	0	-4.430003	-1.370271	0.850911
37	1	0	-4.415553	-1.369927	-0.918836
38	1	0	0.353772	2.769712	-0.070707
39	7	0	5.551064	-2.320687	0.062428
40	1	0	-3.024950	0.916838	-0.049071
41	1	0	-9.015915	-0.677441	-1.423150
42	1	0	-9.894418	0.704463	-0.764918
43	1	0	-9.463500	-0.649275	0.297305
44	1	0	-7.125415	2.161365	0.979689
45	1	0	-8.846699	2.235453	0.641701
46	1	0	-8.234027	1.027703	1.787558
47	20	0	6.258739	-0.071221	0.008879

Ligand	Description	Total Gibbs Free energy (M06-2X/Def2TZVPP)
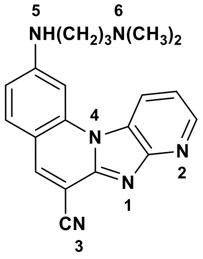 **1b**	Cu^{2+} bound to N1	-2742,2816341 a.u.

CARTESIAN COORDINATES

Center Number	Atomic Number	Atomic Type	Coordinates (Angstroms) X	Y	Z
1	6	0	1.854122	3.925625	-0.069984
2	6	0	1.121371	2.754041	-0.051918
3	6	0	1.856315	1.573468	-0.030403
4	6	0	3.259784	1.661802	-0.027629
5	7	0	3.964411	2.778137	-0.044827
6	6	0	3.255414	3.895351	-0.066050
7	7	0	1.572227	0.199713	-0.007569
8	6	0	2.792953	-0.433372	0.007762
9	7	0	3.809851	0.400016	-0.003594
10	6	0	2.870608	-1.863549	0.033578
11	6	0	1.723919	-2.591180	0.042955
12	6	0	0.465859	-1.936614	0.027093
13	6	0	0.375241	-0.520973	0.000272
14	6	0	-0.729544	-2.674751	0.033970
15	6	0	-1.954226	-2.066678	0.016242
16	6	0	-2.036682	-0.658278	-0.005152
17	6	0	-0.860991	0.100445	-0.015888
18	7	0	-8.187644	0.306213	0.004047
19	6	0	-6.982610	-0.493209	-0.115194
20	6	0	-5.669066	0.261210	0.001944
21	6	0	-4.507191	-0.721923	-0.116131
22	7	0	-3.248751	-0.023031	0.004116
23	6	0	4.209677	-2.352491	0.046836
24	1	0	1.347454	4.879113	-0.087099
25	1	0	3.815515	4.821467	-0.080557
26	1	0	1.757418	-3.672415	0.062583
27	1	0	-0.667435	-3.755015	0.049031
28	1	0	-2.847249	-2.672391	0.012538
29	1	0	-0.937207	1.173650	-0.027844
30	6	0	-9.458893	-0.301286	-0.308807
31	6	0	-8.171221	1.648708	0.527959
32	1	0	-7.057276	-1.026496	-1.068394
33	1	0	-7.063483	-1.265494	0.664855
34	1	0	-5.599044	1.006754	-0.793901
35	1	0	-5.602296	0.777337	0.961008
36	1	0	-4.583871	-1.461132	0.685734
37	1	0	-4.573711	-1.263071	-1.068047
38	1	0	0.045763	2.800255	-0.054215
39	7	0	5.352584	-2.503724	0.053462
40	1	0	-3.245670	0.960328	-0.211128
41	1	0	-9.326864	-1.344508	-0.576751
42	1	0	-9.911711	0.250462	-1.139156
43	1	0	-10.121621	-0.201786	0.554764
44	1	0	-7.429260	2.245232	-0.003122
45	1	0	-9.158283	2.090364	0.434579
46	1	0	-7.881708	1.610497	1.584429
47	29	0	5.714233	-0.320431	0.016294

Ligand	Description	Total Gibbs Free energy (M06-2X/Def2TZVPP)
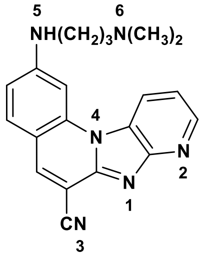 **1b**	Mg^{2+} bound to N1	-1301,8812006 a.u.

CARTESIAN COORDINATES

Center Number	Atomic Number	Atomic Type	Coordinates (Angstroms) X	Y	Z
1	6	0	2.419964	3.834494	-0.089987
2	6	0	1.658516	2.675745	-0.068417
3	6	0	2.367524	1.481359	-0.036176
4	6	0	3.763078	1.557476	-0.029696
5	7	0	4.501961	2.644047	-0.049849
6	6	0	3.817270	3.784969	-0.079889
7	7	0	2.070130	0.098616	-0.007547
8	6	0	3.262980	-0.555707	0.012772
9	7	0	4.303453	0.285089	0.000841
10	6	0	3.338765	-1.972844	0.041748
11	6	0	2.153672	-2.705006	0.048538
12	6	0	0.940757	-2.046045	0.028929
13	6	0	0.854814	-0.606736	0.001524
14	6	0	-0.285881	-2.776578	0.035619
15	6	0	-1.488079	-2.161423	0.018213
16	6	0	-1.560215	-0.730488	-0.006390
17	6	0	-0.353272	0.025013	-0.014434
18	7	0	-7.571232	0.621979	-0.271922
19	6	0	-6.519690	-0.344420	-0.044599
20	6	0	-5.136174	0.297833	-0.039432
21	6	0	-4.046147	-0.758753	-0.029290
22	7	0	-2.728582	-0.114596	-0.021573
23	6	0	4.686096	-2.350976	0.056428
24	1	0	1.929526	4.796328	-0.115578
25	1	0	4.396366	4.698388	-0.097138
26	1	0	2.178985	-3.786603	0.069273
27	1	0	-0.232278	-3.857386	0.055145
28	1	0	-2.398265	-2.741099	0.022954
29	1	0	-0.425689	1.098404	-0.033544
30	6	0	-8.830791	-0.050813	-0.552672
31	6	0	-7.725893	1.538981	0.844718
32	1	0	-6.560921	-1.077277	-0.854368
33	1	0	-6.670335	-0.899759	0.899640
34	1	0	-5.043478	0.923039	-0.930906
35	1	0	-5.015310	0.937594	0.837392
36	1	0	-4.134646	-1.392037	0.856414
37	1	0	-4.121158	-1.394755	-0.914233
38	1	0	0.584132	2.746751	-0.078021
39	7	0	5.844482	-2.261001	0.060649
40	1	0	-2.735866	0.895369	-0.044945
41	1	0	-8.720983	-0.694440	-1.424428
42	1	0	-9.596973	0.690686	-0.770017
43	1	0	-9.173846	-0.663411	0.294723
44	1	0	-6.829368	2.142079	0.981312
45	1	0	-8.549176	2.219840	0.638383
46	1	0	-7.943091	1.012095	1.787553
47	12	0	6.184761	-0.282435	0.021764

Ligand	Description	Total Gibbs Free energy (M06-2X/Def2TZVPP)
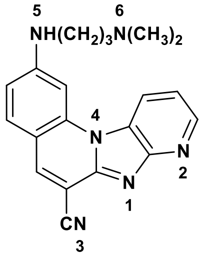 **1b**	Zn^{2+} bound to N1	-2881,1253091 a.u.

CARTESIAN COORDINATES

Center Number	Atomic Number	Atomic Type	Coordinates (Angstroms) X	Y	Z
1	6	0	1.838400	3.892096	-0.094173
2	6	0	1.103029	2.717462	-0.072239
3	6	0	1.836775	1.537440	-0.039341
4	6	0	3.229666	1.646781	-0.033105
5	7	0	3.943769	2.745542	-0.053569
6	6	0	3.236727	3.871889	-0.083871
7	7	0	1.561464	0.148140	-0.010480
8	6	0	2.760700	-0.492500	0.009715
9	7	0	3.781919	0.377772	-0.002452
10	6	0	2.852259	-1.909804	0.038097
11	6	0	1.669696	-2.653058	0.043822
12	6	0	0.452850	-2.009069	0.024606
13	6	0	0.352021	-0.569519	-0.001286
14	6	0	-0.766532	-2.754746	0.030749
15	6	0	-1.974429	-2.153477	0.014552
16	6	0	-2.061735	-0.722198	-0.007926
17	6	0	-0.862090	0.047969	-0.015583
18	7	0	-8.084332	0.573774	-0.265840
19	6	0	-7.024678	-0.385197	-0.046191
20	6	0	-5.647040	0.270017	-0.037517
21	6	0	-4.547605	-0.776703	-0.029089
22	7	0	-3.235087	-0.119875	-0.021578
23	6	0	4.194744	-2.299846	0.053287
24	1	0	1.326857	4.842923	-0.120373
25	1	0	3.798190	4.796305	-0.101416
26	1	0	1.708218	-3.734277	0.063712
27	1	0	-0.699783	-3.834832	0.048961
28	1	0	-2.878580	-2.742590	0.018874
29	1	0	-0.949015	1.119914	-0.032843
30	6	0	-9.336881	-0.107093	-0.559331
31	6	0	-8.251618	1.476206	0.861003
32	1	0	-7.059583	-1.112266	-0.861445
33	1	0	-7.169408	-0.948912	0.893974
34	1	0	-5.559388	0.898327	-0.927307
35	1	0	-5.533289	0.908426	0.841238
36	1	0	-4.628459	-1.411482	0.856161
37	1	0	-4.615979	-1.412099	-0.914906
38	1	0	0.027725	2.766449	-0.082381
39	7	0	5.357747	-2.225957	0.058314
40	1	0	-3.253596	0.890361	-0.042708
41	1	0	-9.218270	-0.738828	-1.438573
42	1	0	-10.108853	0.630179	-0.770280
43	1	0	-9.677272	-0.732910	0.279335
44	1	0	-7.360592	2.084649	1.009470
45	1	0	-9.079029	2.153164	0.658551
46	1	0	-8.469500	0.936325	1.796201
47	30	0	5.593190	-0.253817	0.018597

Ligand	Description	Total Gibbs Free energy (M06-2X/Def2TZVPP)
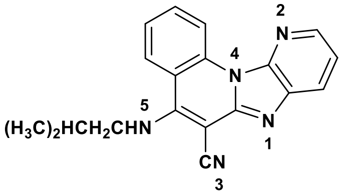 2a	H^+ bound to N1	-1008.105577 a.u.

CARTESIAN COORDINATES

Center Number	Atomic Number	Atomic Type	Coordinates (Angstroms) X	Y	Z
1	7	0	-1.588918	0.179171	-0.016199
2	6	0	-0.951891	1.426487	-0.056093
3	6	0	-0.870404	-0.964035	-0.016429
4	7	0	-1.704602	-2.018221	0.027531
5	6	0	-3.011915	-1.561853	0.057087
6	6	0	-2.957950	-0.167888	0.030256
7	6	0	0.530051	-0.999276	-0.061942
8	7	0	-3.989321	0.639363	0.045348
9	6	0	-4.241584	-2.190184	0.103727
10	6	0	-5.177203	0.035948	0.090326
11	6	0	-5.343458	-1.349268	0.120115
12	1	0	-6.039851	0.688096	0.103114
13	1	0	-6.341003	-1.761223	0.155920
14	1	0	-4.341238	-3.266086	0.125932
15	6	0	0.454307	1.456904	-0.100681
16	6	0	1.234685	0.213802	-0.111712
17	6	0	1.068342	-2.317203	-0.065646
18	7	0	1.340305	-3.433712	-0.062521
19	7	0	2.554084	0.279555	-0.166400
20	6	0	3.516348	-0.824004	-0.178785
21	1	0	2.966311	1.195164	-0.225397
22	6	0	-1.703453	2.601077	-0.047977
23	6	0	1.071575	2.716252	-0.128571
24	6	0	0.335082	3.877729	-0.120102
25	6	0	-1.056365	3.816802	-0.081397
26	1	0	2.147506	2.810817	-0.153462
27	1	0	0.838512	4.833344	-0.141956
28	1	0	-1.638738	4.727331	-0.075017
29	1	0	-2.778920	2.541692	-0.014726
30	6	0	4.941026	-0.280224	-0.189250
31	1	0	3.366636	-1.442681	0.708804
32	1	0	3.340963	-1.443596	-1.059598
33	6	0	5.913518	-1.441228	-0.366230
34	1	0	5.036240	0.385344	-1.054420
35	6	0	5.253649	0.500573	1.084528
36	1	0	6.939004	-1.076981	-0.394961
37	1	0	5.830683	-2.139950	0.468307
38	1	0	5.721952	-1.988608	-1.288714
39	1	0	6.269711	0.890436	1.050566
40	1	0	4.585847	1.349585	1.244408
41	1	0	5.173457	-0.151848	1.956119
42	1	0	-1.384063	-2.976403	0.033426

Ligand	Description	Total Gibbs Free energy (M06-2X/Def2TZVPP)
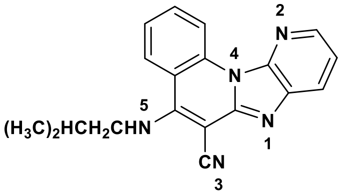 2a	Ca^{2+} bound to N1	-1684,8615911 a.u.

CARTESIAN COORDINATES

Center Number	Atomic Number	Atomic Type	Coordinates (Angstroms) X	Y	Z
1	6	0	4.443041	2.327923	0.216558
2	7	0	3.111622	2.330469	0.200066
3	6	0	2.554153	1.149456	0.098178
4	6	0	3.213048	-0.078504	0.008438
5	6	0	4.599150	-0.070552	0.024880
6	6	0	5.214054	1.165643	0.131490
7	7	0	1.177752	0.845338	0.061772
8	6	0	1.081041	-0.494467	-0.043522
9	7	0	2.276475	-1.116196	-0.079470
10	6	0	-0.196577	-1.117544	-0.049979
11	6	0	-1.391760	-0.363751	0.097541
12	6	0	-1.244799	1.083580	0.001609
13	6	0	0.041488	1.675273	0.034069
14	6	0	-2.340742	1.934491	-0.213857
15	6	0	-2.184731	3.299738	-0.291515
16	6	0	-0.918246	3.860204	-0.156962
17	6	0	0.196038	3.055637	-0.014571
18	6	0	-0.066460	-2.502034	-0.059420
19	1	0	4.921310	3.294306	0.300964
20	1	0	6.291138	1.242895	0.149486
21	7	0	-2.528652	-1.006249	0.306104
22	1	0	-3.317833	1.514468	-0.374251
23	1	0	-3.044535	3.929923	-0.467446
24	1	0	1.184431	3.482151	0.038957
25	1	0	-0.792324	4.933202	-0.198921
26	7	0	0.316149	-3.598743	-0.087384
27	6	0	-3.824972	-0.602586	0.887777
28	1	0	-2.465808	-2.014489	0.242534
29	6	0	-5.005374	-0.947978	-0.019224
30	6	0	-6.299973	-0.727444	0.757613
31	6	0	-5.002904	-0.162166	-1.325029
32	1	0	-4.924348	-2.014718	-0.255834
33	1	0	-4.049234	-0.231040	-1.852565
34	1	0	-5.774800	-0.542397	-1.991899
35	1	0	-5.229563	0.891163	-1.144744
36	1	0	-6.337714	-1.332923	1.662861
37	1	0	-7.158803	-0.988805	0.142255
38	1	0	-6.403620	0.321203	1.043114
39	1	0	-3.912065	-1.165116	1.819751
40	1	0	-3.797357	0.447607	1.156598
41	20	0	2.597469	-3.394293	-0.176993
42	1	0	5.195473	-0.973407	-0.043058

Ligand	Description	Total Gibbs Free energy (M06-2X/Def2TZVPP)
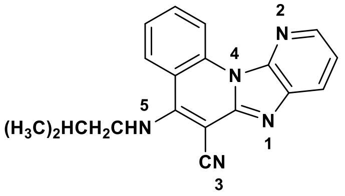 2a	Cu^{2+} bound to N1	-2647,6329937 a.u.

CARTESIAN COORDINATES

Center Number	Atomic Number	Atomic Type	Coordinates (Angstroms) X	Y	Z
1	6	0	4.299312	2.527309	0.199343
2	7	0	2.973585	2.539860	0.187787
3	6	0	2.418866	1.352438	0.101024
4	6	0	3.112049	0.097059	0.019607
5	6	0	4.520774	0.114836	0.033728
6	6	0	5.100251	1.347161	0.124511
7	7	0	1.077510	1.040463	0.072273
8	6	0	1.001151	-0.322539	-0.019053
9	7	0	2.230831	-0.910739	-0.054339
10	6	0	-0.219525	-0.971699	-0.017872
11	6	0	-1.445652	-0.230599	0.150075
12	6	0	-1.349444	1.216974	0.021826
13	6	0	-0.081067	1.848261	0.032762
14	6	0	-2.472647	2.031164	-0.188356
15	6	0	-2.356029	3.401243	-0.288949
16	6	0	-1.106343	3.999115	-0.184067
17	6	0	0.035355	3.227493	-0.042294
18	6	0	-0.130914	-2.388473	-0.058374
19	1	0	4.784490	3.492715	0.271809
20	1	0	6.175438	1.455518	0.141956
21	7	0	-2.522756	-0.923805	0.436622
22	1	0	-3.440340	1.582585	-0.328439
23	1	0	-3.236604	4.003717	-0.459443
24	1	0	1.009418	3.687786	-0.007848
25	1	0	-1.011338	5.074382	-0.246213
26	7	0	0.123097	-3.513584	-0.101284
27	6	0	-3.865305	-0.570907	0.936182
28	1	0	-2.404644	-1.932191	0.412437
29	6	0	-4.973035	-1.002728	-0.031614
30	6	0	-6.317601	-0.830729	0.669293
31	6	0	-4.933255	-0.246931	-1.354342
32	1	0	-4.822632	-2.068908	-0.231794
33	1	0	-3.948688	-0.273088	-1.827271
34	1	0	-5.640653	-0.687948	-2.054370
35	1	0	-5.231266	0.794876	-1.217184
36	1	0	-6.376206	-1.417982	1.585064
37	1	0	-7.123649	-1.151645	0.012147
38	1	0	-6.491408	0.217240	0.920438
39	1	0	-3.968477	-1.128031	1.869665
40	1	0	-3.902246	0.484431	1.181786
41	29	0	2.273448	-2.984612	-0.151946
42	1	0	5.101309	-0.795771	-0.024286

Ligand	Description	Total Gibbs Free energy (M06-2X/Def2TZVPP)
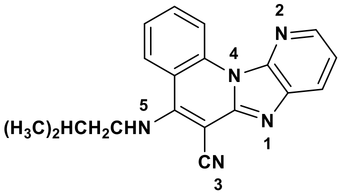 2a	Mg^{2+} bound to N1	-1207,2659903 a.u.

CARTESIAN COORDINATES

Center Number	Atomic Number	Atomic Type	Coordinates (Angstroms) X	Y	Z
1	6	0	4.725485	1.785439	0.209300
2	7	0	3.398186	1.888518	0.199921
3	6	0	2.753707	0.754191	0.091014
4	6	0	3.321031	-0.514718	-0.011630
5	6	0	4.702423	-0.617806	-0.001891
6	6	0	5.406965	0.568412	0.111129
7	7	0	1.353613	0.557351	0.060616
8	6	0	1.147404	-0.764327	-0.053901
9	7	0	2.301171	-1.471929	-0.101658
10	6	0	-0.178867	-1.286483	-0.062480
11	6	0	-1.311832	-0.436058	0.106480
12	6	0	-1.045623	0.993036	0.011883
13	6	0	0.285815	1.477664	0.040368
14	6	0	-2.069153	1.932292	-0.193792
15	6	0	-1.801289	3.280401	-0.267588
16	6	0	-0.492612	3.734067	-0.137449
17	6	0	0.553131	2.839787	-0.003710
18	6	0	-0.133285	-2.671784	-0.104685
19	1	0	5.275489	2.712425	0.299185
20	1	0	6.486921	0.564338	0.124828
21	7	0	-2.486957	-0.987682	0.337572
22	1	0	-3.078337	1.595315	-0.350823
23	1	0	-2.607205	3.979906	-0.436642
24	1	0	1.573124	3.184266	0.047078
25	1	0	-0.278779	4.793234	-0.175912
26	7	0	0.252221	-3.770082	-0.165225
27	6	0	-3.756943	-0.479592	0.898700
28	1	0	-2.505745	-1.998393	0.281078
29	6	0	-4.946736	-0.732819	-0.027054
30	6	0	-6.230930	-0.408230	0.730754
31	6	0	-4.862480	0.046805	-1.333827
32	1	0	-4.947072	-1.803402	-0.260110
33	1	0	-3.908802	-0.095681	-1.846814
34	1	0	-5.649892	-0.276279	-2.012489
35	1	0	-5.012294	1.114782	-1.159884
36	1	0	-6.330048	-1.006356	1.636174
37	1	0	-7.097994	-0.603220	0.102519
38	1	0	-6.256144	0.646210	1.012416
39	1	0	-3.899733	-1.034837	1.827943
40	1	0	-3.647955	0.564854	1.168647
41	12	0	2.211478	-3.440567	-0.208910
42	1	0	5.221827	-1.565246	-0.078578

Ligand	Description	Total Gibbs Free energy (M06-2X/Def2TZVPP)
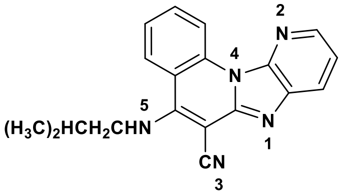 **2a**	Zn^{2+} bound to N1	-2786,5132958 a.u.

CARTESIAN COORDINATES

Center Number	Atomic Number	Atomic Type	X	Y	Z
1	6	0	4.278862	2.569872	0.220965
2	7	0	2.948298	2.525636	0.204241
3	6	0	2.432346	1.327503	0.104864
4	6	0	3.141785	0.131546	0.018871
5	6	0	4.525079	0.176162	0.035990
6	6	0	5.091947	1.434465	0.139451
7	7	0	1.059488	0.978604	0.070276
8	6	0	0.992387	-0.357056	-0.030456
9	7	0	2.226328	-0.921699	-0.066160
10	6	0	-0.269163	-1.022140	-0.038690
11	6	0	-1.486825	-0.289592	0.126060
12	6	0	-1.372707	1.157238	0.009070
13	6	0	-0.100420	1.781040	0.032852
14	6	0	-2.489655	1.979190	-0.212595
15	6	0	-2.365674	3.346799	-0.307275
16	6	0	-1.112913	3.938581	-0.182311
17	6	0	0.021694	3.162566	-0.032578
18	6	0	-0.091033	-2.397696	-0.075413
19	1	0	4.722344	3.552918	0.303379
20	1	0	6.165606	1.550495	0.158669
21	7	0	-2.593564	-0.958812	0.373677
22	1	0	-3.456748	1.534489	-0.366450
23	1	0	-3.240518	3.954395	-0.488736
24	1	0	0.998779	3.614830	0.014599
25	1	0	-1.012315	5.013728	-0.237302
26	7	0	0.403366	-3.455719	-0.131187
27	6	0	-3.915774	-0.579084	0.917613
28	1	0	-2.506523	-1.967075	0.335550
29	6	0	-5.063650	-0.975096	-0.011433
30	6	0	-6.381222	-0.773838	0.731823
31	6	0	-5.050958	-0.216027	-1.332964
32	1	0	-4.948647	-2.044007	-0.222574
33	1	0	-4.083633	-0.266526	-1.837865
34	1	0	-5.794006	-0.633918	-2.009859
35	1	0	-5.314798	0.833141	-1.181726
36	1	0	-6.424148	-1.361852	1.648154
37	1	0	-7.217149	-1.071836	0.101879
38	1	0	-6.520310	0.277195	0.992072
39	1	0	-4.004351	-1.131297	1.855311
40	1	0	-3.920603	0.475450	1.169620
41	30	0	2.280172	-2.838662	-0.134878
42	1	0	5.139818	-0.712935	-0.027366

Ligand	Description	Total Gibbs Free energy (M06-2X/Def2TZVPP)
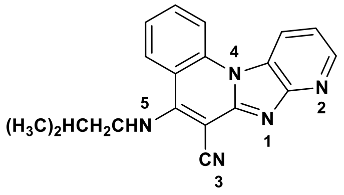 **2b**	H^+ bound to N1	-1008.099706 a.u.

CARTESIAN COORDINATES

Center Number	Atomic Number	Atomic Type	Coordinates (Angstroms) X	Y	Z
1	7	0	1.593886	0.237286	-0.034485
2	6	0	0.928921	1.468272	-0.098068
3	6	0	0.886196	-0.917024	-0.116665
4	7	0	1.727845	-1.957878	-0.093463
5	6	0	3.025870	-1.490018	0.030733
6	6	0	2.962571	-0.093055	0.081466
7	6	0	-0.513127	-0.971785	-0.191336
8	6	0	4.153468	0.595139	0.261644
9	7	0	4.096267	-2.239076	0.105790
10	6	0	5.302076	-0.178745	0.341980
11	6	0	5.236000	-1.568627	0.253986
12	1	0	6.260150	0.299541	0.480574
13	1	0	6.136704	-2.164739	0.314546
14	6	0	-0.478328	1.478821	-0.142929
15	6	0	-1.241345	0.225016	-0.161608
16	6	0	-1.034316	-2.294195	-0.280009
17	7	0	-1.307075	-3.407764	-0.352862
18	7	0	-2.562952	0.270133	-0.159308
19	6	0	-3.509064	-0.848440	-0.155859
20	1	0	-2.993359	1.178907	-0.144031
21	6	0	1.646482	2.661923	-0.137961
22	6	0	-1.117174	2.726414	-0.196388
23	6	0	-0.407223	3.903894	-0.213005
24	6	0	0.984211	3.868130	-0.191904
25	1	0	-2.194112	2.797807	-0.238809
26	1	0	-0.929221	4.848551	-0.256231
27	1	0	1.553267	4.786332	-0.226226
28	1	0	2.720468	2.650149	-0.151320
29	6	0	-4.937866	-0.328207	-0.039678
30	1	0	-3.389175	-1.424587	-1.074221
31	1	0	-3.287177	-1.503521	0.689469
32	1	0	4.228931	1.664471	0.356511
33	6	0	-5.904441	-1.496547	-0.200627
34	6	0	-5.168997	0.388852	1.287840
35	1	0	-5.104236	0.373871	-0.864353
36	1	0	-6.934285	-1.149158	-0.138612
37	1	0	-5.772418	-1.997959	-1.158944
38	1	0	-5.751518	-2.231042	0.592234
39	1	0	-6.190322	0.761941	1.344464
40	1	0	-5.017095	-0.300752	2.120387
41	1	0	-4.503734	1.241531	1.438980
42	1	0	1.447842	-2.929161	-0.133791

Ligand	Description	Total Gibbs Free energy (M06-2X/Def2TZVPP)
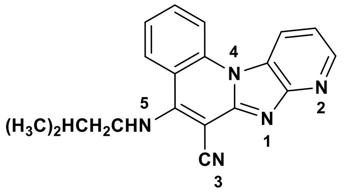 **2b**	Ca^{2+} bound to N1	-1684,8125158 a.u.

CARTESIAN COORDINATES

Center Number	Atomic Number	Atomic Type	Coordinates (Angstroms) X	Y	Z
1	6	0	-1.080767	3.318437	-0.034834
2	6	0	-0.517795	2.048173	-0.047435
3	6	0	0.885281	1.883563	-0.091768
4	6	0	1.673486	3.042392	-0.120528
5	6	0	1.112879	4.299375	-0.108171
6	6	0	-0.271548	4.435663	-0.065467
7	7	0	-1.337982	0.906739	-0.015382
8	6	0	-0.775089	-0.325283	-0.025909
9	6	0	0.625441	-0.548391	-0.069299
10	6	0	1.501849	0.550512	-0.105592
11	7	0	-1.675859	-1.300095	0.006912
12	6	0	-2.896592	-0.675755	0.040798
13	6	0	-2.740122	0.713562	0.028639
14	7	0	-4.019957	-1.366666	0.078316
15	6	0	-5.125559	-0.618661	0.108263
16	6	0	-5.093604	0.777206	0.100238
17	6	0	-3.895228	1.482178	0.059882
18	6	0	0.833664	-1.941210	-0.070287
19	7	0	2.807430	0.404694	-0.149384
20	1	0	-6.027271	1.319233	0.126210
21	1	0	-6.070740	-1.142873	0.139932
22	1	0	2.751774	2.979005	-0.152945
23	1	0	1.746905	5.173630	-0.131199
24	1	0	-2.149710	3.437624	-0.000834
25	1	0	-3.913217	2.559817	0.054631
26	1	0	-0.720585	5.418914	-0.055573
27	7	0	0.610772	-3.078342	-0.058428
28	1	0	3.383386	1.231478	-0.177493
29	6	0	3.525289	-0.868344	-0.172633
30	1	0	3.208893	-1.432247	-1.055265
31	1	0	3.260113	-1.439679	0.723931
32	6	0	5.034800	-0.645268	-0.205089
33	1	0	5.255718	-0.026406	-1.081047
34	6	0	5.520775	0.065667	1.054837
35	6	0	5.731062	-1.990976	-0.376636
36	1	0	5.519117	-2.644506	0.471861
37	1	0	6.809419	-1.853550	-0.425584
38	1	0	5.415497	-2.496021	-1.289446
39	1	0	5.058224	1.043150	1.207055
40	1	0	6.596072	0.226214	1.003893
41	1	0	5.315024	-0.541747	1.938279
42	20	0	-1.720939	-3.539670	0.013765

Ligand	Description	Total Gibbs Free energy (M06-2X/Def2TZVPP)
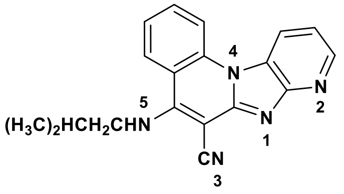<br>2b	Cu^{2+} bound to N1	-2647,5973695 a.u.

CARTESIAN COORDINATES

Center Number	Atomic Number	Atomic Type	Coordinates (Angstroms) X	Y	Z
1	6	0	-1.081341	3.430989	-0.183688
2	6	0	-0.523276	2.163374	-0.103062
3	6	0	0.876263	1.994465	-0.135614
4	6	0	1.673000	3.143374	-0.225212
5	6	0	1.117798	4.403032	-0.278592
6	6	0	-0.264955	4.543927	-0.265162
7	7	0	-1.342091	1.014697	-0.015177
8	6	0	-0.791932	-0.245850	-0.082513
9	6	0	0.579106	-0.459396	-0.142259
10	6	0	1.473622	0.657266	-0.116287
11	7	0	-1.735068	-1.214264	-0.043529
12	6	0	-2.905024	-0.582881	0.072239
13	6	0	-2.701727	0.843218	0.110909
14	7	0	-4.086177	-1.203184	0.156059
15	6	0	-5.115559	-0.413028	0.299153
16	6	0	-5.019866	1.018453	0.383093
17	6	0	-3.813081	1.669214	0.293133
18	6	0	0.912158	-1.843117	-0.184463
19	7	0	2.768923	0.497247	-0.082037
20	1	0	-5.929119	1.585438	0.527657
21	1	0	-6.091044	-0.879923	0.365816
22	1	0	2.750695	3.073835	-0.274492
23	1	0	1.755539	5.272221	-0.348310
24	1	0	-2.147882	3.563482	-0.210338
25	1	0	-3.770650	2.741119	0.382609
26	1	0	-0.712007	5.526126	-0.329948
27	7	0	0.912953	-2.995200	-0.213432
28	1	0	3.335739	1.334143	-0.033872
29	6	0	3.553869	-0.749749	-0.092707
30	1	0	3.304360	-1.306405	-0.998007
31	1	0	3.277030	-1.344042	0.782355
32	6	0	5.046734	-0.432884	-0.053905
33	1	0	5.270074	0.206423	-0.914816
34	6	0	5.430552	0.289521	1.234480
35	6	0	5.824384	-1.736473	-0.205601
36	1	0	5.613584	-2.409763	0.627217
37	1	0	6.893670	-1.535490	-0.206145
38	1	0	5.578305	-2.248618	-1.135392
39	1	0	4.908790	1.239564	1.378402
40	1	0	6.495380	0.514187	1.233476
41	1	0	5.222153	-0.339415	2.101877
42	29	0	-1.276163	-3.236161	-0.120860

Ligand	Description	Total Gibbs Free energy (M06-2X/Def2TZVPP)
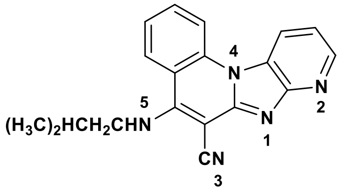 **2b**	Mg^{2+} bound to N1	-1207,1777831 a.u.

CARTESIAN COORDINATES

Center Number	Atomic Number	Atomic Type	Coordinates (Angstroms) X	Y	Z
1	6	0	-1.436795	3.013776	-0.035985
2	6	0	-0.767401	1.796641	-0.048119
3	6	0	0.644249	1.758813	-0.093273
4	6	0	1.331324	2.980618	-0.125470
5	6	0	0.664119	4.183796	-0.113783
6	6	0	-0.726913	4.197039	-0.068594
7	7	0	-1.488536	0.586925	-0.015042
8	6	0	-0.834577	-0.594385	-0.025203
9	6	0	0.587464	-0.686294	-0.069007
10	6	0	1.369662	0.487557	-0.104815
11	7	0	-1.669905	-1.637155	0.009646
12	6	0	-2.944089	-1.101545	0.044555
13	6	0	-2.874258	0.292426	0.030325
14	7	0	-4.022583	-1.851206	0.084163
15	6	0	-5.169450	-1.175518	0.113548
16	6	0	-5.226742	0.221212	0.102934
17	6	0	-4.074488	0.992731	0.060724
18	6	0	0.934996	-2.040513	-0.068754
19	7	0	2.680152	0.443094	-0.145810
20	1	0	-6.191342	0.706373	0.128166
21	1	0	-6.078496	-1.760521	0.146735
22	1	0	2.411107	3.008966	-0.161026
23	1	0	1.218818	5.110353	-0.139688
24	1	0	-2.510732	3.044579	-0.000916
25	1	0	-4.157786	2.066495	0.053423
26	1	0	-1.260806	5.136985	-0.058716
27	7	0	0.812680	-3.197104	-0.055240
28	1	0	3.190140	1.313210	-0.168185
29	6	0	3.497455	-0.769279	-0.168086
30	1	0	3.225487	-1.356682	-1.050848
31	1	0	3.281807	-1.356167	0.732441
32	6	0	4.985611	-0.429082	-0.205057
33	1	0	5.155070	0.198745	-1.085957
34	6	0	5.415060	0.326153	1.049538
35	6	0	5.783157	-1.718419	-0.368986
36	1	0	5.626010	-2.379725	0.485342
37	1	0	6.847162	-1.497230	-0.422935
38	1	0	5.505910	-2.253366	-1.277206
39	1	0	4.880056	1.267097	1.195356
40	1	0	6.474700	0.568552	0.995422
41	1	0	5.258030	-0.288577	1.937870
42	12	0	-1.167341	-3.529610	0.008979

Ligand	Description	Total Gibbs Free energy (M06-2X/Def2TZVPP)
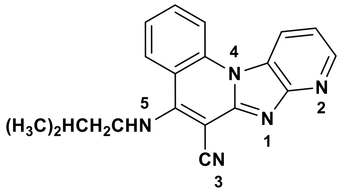 **2b**	Zn^{2+} bound to N1	-2786,4135653 a.u.

CARTESIAN COORDINATES

Center Number	Atomic Number	Atomic Type	Coordinates (Angstroms) X	Y	Z
1	6	0	-1.115459	3.400540	-0.038177
2	6	0	-0.528781	2.141512	-0.048947
3	6	0	0.876983	2.011167	-0.092667
4	6	0	1.644875	3.184128	-0.123623
5	6	0	1.059344	4.428791	-0.112753
6	6	0	-0.327899	4.533853	-0.070008
7	7	0	-1.329297	0.980928	-0.015962
8	6	0	-0.757791	-0.240950	-0.025845
9	6	0	0.656754	-0.427520	-0.069266
10	6	0	1.514737	0.696143	-0.104429
11	7	0	-1.680076	-1.214118	0.009348
12	6	0	-2.917358	-0.594550	0.044055
13	6	0	-2.734724	0.788695	0.029345
14	7	0	-4.048757	-1.254999	0.083890
15	6	0	-5.138703	-0.493034	0.113319
16	6	0	-5.086452	0.904500	0.102726
17	6	0	-3.877321	1.581230	0.060269
18	6	0	0.943010	-1.794784	-0.070006
19	7	0	2.818020	0.562104	-0.145232
20	1	0	-6.009425	1.464912	0.128417
21	1	0	-6.090060	-1.006554	0.146731
22	1	0	2.724174	3.140483	-0.157358
23	1	0	1.674274	5.316602	-0.137355
24	1	0	-2.184250	3.505258	-0.005082
25	1	0	-3.876724	2.657891	0.053360
26	1	0	-0.798560	5.507080	-0.061354
27	7	0	0.774689	-2.948894	-0.057638
28	1	0	3.385448	1.396359	-0.167062
29	6	0	3.555930	-0.700966	-0.167421
30	1	0	3.247602	-1.269097	-1.051016
31	1	0	3.304993	-1.271391	0.734671
32	6	0	5.063440	-0.456739	-0.205248
33	1	0	5.271700	0.157540	-1.087388
34	6	0	5.539968	0.271143	1.048453
35	6	0	5.775871	-1.795183	-0.367859
36	1	0	5.577410	-2.443963	0.487448
37	1	0	6.851781	-1.642527	-0.422633
38	1	0	5.464686	-2.312312	-1.275381
39	1	0	5.066772	1.244961	1.193218
40	1	0	6.612901	0.445094	0.993601
41	1	0	5.344437	-0.331112	1.937661
42	30	0	-1.206258	-3.068741	0.005239

Ligand	Description	Total Gibbs Free energy (M06-2X/Def2TZVPP)
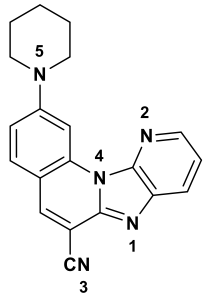<br>3a	H^+ bound to N1	-1046.191269 a.u.

CARTESIAN COORDINATES

Center Number	Atomic Number	Atomic Type	Coordinates (Angstroms) X	Y	Z
1	7	0	-1.499031	0.022700	0.070689
2	6	0	-0.310426	-0.709948	0.193893
3	6	0	-2.703323	-0.583007	-0.095285
4	7	0	-3.667486	0.351651	-0.179759
5	6	0	-3.096382	1.604640	-0.067064
6	6	0	-1.722271	1.415652	0.092249
7	6	0	-2.838884	-1.976934	-0.157545
8	7	0	-0.829297	2.367509	0.233000
9	6	0	-3.606662	2.890519	-0.083348
10	6	0	-1.319507	3.605574	0.217893
11	6	0	-2.675756	3.904399	0.065058
12	1	0	-0.598546	4.403500	0.333002
13	1	0	-2.991638	4.937089	0.063631
14	1	0	-4.661458	3.092758	-0.203476
15	6	0	-0.439978	-2.124320	0.127108
16	6	0	-1.685901	-2.728143	-0.044362
17	1	0	-1.751356	-3.807460	-0.091016
18	6	0	0.903699	-0.087224	0.358197
19	6	0	0.756824	-2.875229	0.236043
20	6	0	1.964150	-2.280869	0.400656
21	6	0	2.087467	-0.853695	0.485104
22	1	0	0.692265	-3.954412	0.188311
23	1	0	2.834015	-2.909227	0.487148
24	7	0	3.278043	-0.266424	0.694372
25	1	0	0.927290	0.985489	0.369422
26	6	0	-4.150829	-2.501900	-0.334040
27	7	0	-5.240258	-2.834595	-0.477453
28	6	0	4.562951	-0.932241	0.462483
29	6	0	5.091117	-0.544139	-0.918496
30	1	0	5.249019	-0.589864	1.239683
31	1	0	4.473978	-2.005945	0.568261
32	6	0	5.210408	0.974147	-1.046190
33	1	0	6.058018	-1.020417	-1.081175
34	1	0	4.404295	-0.932872	-1.675523
35	6	0	3.906018	1.671385	-0.658911
36	1	0	6.008370	1.325020	-0.385383
37	1	0	5.499470	1.245294	-2.060803
38	6	0	3.448109	1.187413	0.714656
39	1	0	4.038367	2.753201	-0.640760
40	1	0	3.124820	1.448086	-1.391121
41	1	0	4.218079	1.403464	1.458455
42	1	0	2.540731	1.675963	1.052254
43	1	0	-4.644455	0.127973	-0.305452

Ligand	Description	Total Gibbs Free energy (M06-2X/Def2TZVPP)
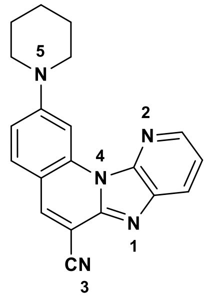 3a	Ca^{2+} bound to N1	-1722,9140034 a.u.

CARTESIAN COORDINATES

Center Number	Atomic Number	Atomic Type	Coordinates (Angstroms) X	Y	Z
1	6	0	0.790802	3.642981	0.248344
2	7	0	0.290253	2.409105	0.243604
3	6	0	1.179401	1.449232	0.137867
4	6	0	2.564518	1.603865	0.031832
5	6	0	3.073152	2.894053	0.037609
6	6	0	2.155791	3.924809	0.148980
7	7	0	0.943813	0.063346	0.107010
8	6	0	2.160313	-0.531617	-0.012585
9	7	0	3.177523	0.352026	-0.062798
10	6	0	2.249599	-1.947096	-0.069858
11	6	0	1.078575	-2.709681	0.002376
12	6	0	-0.143794	-2.092241	0.127905
13	6	0	-0.259941	-0.661097	0.184683
14	6	0	-1.362317	-2.831200	0.202289
15	6	0	-2.561252	-2.227249	0.332083
16	6	0	-2.672225	-0.788988	0.407092
17	6	0	-1.461149	-0.031444	0.307924
18	7	0	-3.846779	-0.194035	0.562688
19	6	0	3.569865	-2.372942	-0.194502
20	7	0	4.725802	-2.434192	-0.293759
21	1	0	0.075208	4.449216	0.335374
22	1	0	4.132436	3.109395	-0.039810
23	1	0	2.485026	4.953365	0.160068
24	1	0	1.135738	-3.789516	-0.039729
25	1	0	-1.309486	-3.910659	0.145028
26	1	0	-3.442531	-2.843980	0.364121
27	1	0	-1.469579	1.040932	0.341570
28	6	0	-5.136805	-0.900164	0.615314
29	6	0	-5.889942	-0.686115	-0.695183
30	6	0	-6.095298	0.805041	-0.950084
31	6	0	-4.775330	1.566373	-0.856778
32	6	0	-4.062378	1.257022	0.459687
33	1	0	-5.695592	-0.462376	1.444719
34	1	0	-4.997926	-1.947992	0.845570
35	1	0	-6.846292	-1.205240	-0.640983
36	1	0	-5.318317	-1.138027	-1.510259
37	1	0	-6.790995	1.202009	-0.205911
38	1	0	-6.552969	0.962777	-1.925350
39	1	0	-4.943726	2.641165	-0.919772
40	1	0	-4.117570	1.289271	-1.684930
41	1	0	-4.688967	1.544951	1.305845
42	1	0	-3.129608	1.798752	0.548487
43	20	0	5.401568	-0.262453	-0.260561

Ligand	Description	Total Gibbs Free energy (M06-2X/Def2TZVPP)
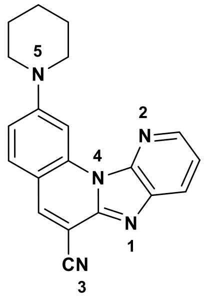 3a	Cu^{2+} bound to N1	-2685,7586946 a.u.

CARTESIAN COORDINATES

Center Number	Atomic Number	Atomic Type	Coordinates (Angstroms) X	Y	Z
1	6	0	-0.394478	3.656121	-0.244215
2	7	0	0.063093	2.408548	-0.252362
3	6	0	-0.874418	1.488178	-0.146870
4	6	0	-2.253922	1.699404	-0.031485
5	6	0	-2.716168	3.013777	-0.023915
6	6	0	-1.754410	3.994877	-0.133182
7	7	0	-0.702645	0.101125	-0.127662
8	6	0	-1.959286	-0.430144	-0.006436
9	7	0	-2.907407	0.485919	0.054542
10	6	0	-2.116363	-1.856436	0.036273
11	6	0	-1.027111	-2.666825	-0.044503
12	6	0	0.271119	-2.097856	-0.169868
13	6	0	0.441661	-0.689761	-0.216687
14	6	0	1.423409	-2.912602	-0.253960
15	6	0	2.668322	-2.377057	-0.368255
16	6	0	2.840057	-0.956315	-0.418315
17	6	0	1.692513	-0.124362	-0.340654
18	7	0	4.065750	-0.406399	-0.545133
19	6	0	-3.476954	-2.275631	0.163896
20	7	0	-4.620025	-2.375144	0.265281
21	1	0	0.347731	4.438480	-0.330296
22	1	0	-3.767792	3.251742	0.061880
23	1	0	-2.033517	5.038379	-0.135614
24	1	0	-1.136683	-3.742663	-0.013340
25	1	0	1.301676	-3.987438	-0.237720
26	1	0	3.510312	-3.041317	-0.465572
27	1	0	1.763659	0.950427	-0.336065
28	6	0	5.317649	-1.115629	-0.261724
29	6	0	5.898734	-0.556445	1.051240
30	6	0	6.108937	0.949414	0.962955
31	6	0	4.829816	1.655050	0.528755
32	6	0	4.296119	1.024844	-0.765597
33	1	0	5.996728	-0.917113	-1.091854
34	1	0	5.155638	-2.181847	-0.186916
35	1	0	6.836010	-1.078696	1.239560
36	1	0	5.215292	-0.805321	1.866329
37	1	0	6.904685	1.166971	0.246518
38	1	0	6.438689	1.332946	1.927762
39	1	0	4.061285	1.580554	1.302294
40	1	0	5.005005	2.713095	0.338537
41	1	0	3.398824	1.505478	-1.134584
42	1	0	5.056719	1.090270	-1.546027
43	29	0	-4.849551	-0.184410	0.235438

Ligand	Description	Total Gibbs Free energy (M06-2X/Def2TZVPP)
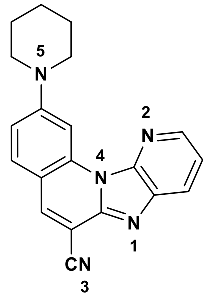 **3a**	Mg^{2+} bound to N1	-1245,3128689 a.u.

CARTESIAN COORDINATES

Center Number	Atomic Number	Atomic Type	Coordinates (Angstroms) X	Y	Z
1	6	0	1.080971	3.624707	0.227263
2	7	0	0.573883	2.393188	0.222645
3	6	0	1.457748	1.430263	0.112058
4	6	0	2.840240	1.582573	0.001326
5	6	0	3.360637	2.866503	0.006435
6	6	0	2.447244	3.900131	0.122955
7	7	0	1.211439	0.042133	0.080415
8	6	0	2.415947	-0.565225	-0.043985
9	7	0	3.438288	0.322116	-0.096711
10	6	0	2.496721	-1.983260	-0.103129
11	6	0	1.313627	-2.737993	-0.027303
12	6	0	0.103643	-2.108283	0.102828
13	6	0	-0.000220	-0.672393	0.162153
14	6	0	-1.124329	-2.836939	0.182111
15	6	0	-2.314979	-2.222663	0.319711
16	6	0	-2.412566	-0.781227	0.397355
17	6	0	-1.192267	-0.032971	0.291962
18	7	0	-3.577537	-0.177275	0.558940
19	6	0	3.818622	-2.389477	-0.230359
20	7	0	4.980311	-2.366295	-0.328002
21	1	0	0.369642	4.434250	0.318323
22	1	0	4.421084	3.070058	-0.075004
23	1	0	2.781652	4.927046	0.134328
24	1	0	1.361834	-3.818061	-0.070531
25	1	0	-1.081258	-3.916607	0.121498
26	1	0	-3.202319	-2.830578	0.353678
27	1	0	-1.191277	1.039391	0.329442
28	6	0	-4.873449	-0.872888	0.644703
29	6	0	-5.658735	-0.651466	-0.645440
30	6	0	-5.860010	0.841115	-0.892869
31	6	0	-4.531806	1.590753	-0.835320
32	6	0	-3.784234	1.277006	0.461118
33	1	0	-5.404594	-0.428743	1.488641
34	1	0	-4.737262	-1.921604	0.871803
35	1	0	-6.616539	-1.164265	-0.564569
36	1	0	-5.113200	-1.107343	-1.475956
37	1	0	-6.532358	1.242728	-0.130110
38	1	0	-6.342042	1.003359	-1.855476
39	1	0	-4.691873	2.667105	-0.891930
40	1	0	-3.899433	1.309497	-1.681539
41	1	0	-4.383488	1.569478	1.325129
42	1	0	-2.844774	1.810762	0.520454
43	12	0	5.268724	-0.411095	-0.275052

Ligand	Description	Total Gibbs Free energy (M06-2X/Def2TZVPP)
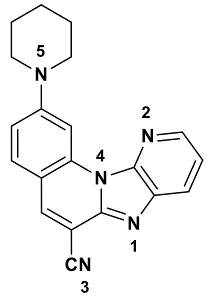<br>**3a**	Zn^{2+} bound to N1	-2824,6210275 a.u.

CARTESIAN COORDINATES

Center Number	Atomic Number	Atomic Type	Coordinates (Angstroms) X	Y	Z
1	6	0	0.536580	3.677318	0.243393
2	7	0	0.060276	2.433307	0.237493
3	6	0	0.967331	1.492181	0.137961
4	6	0	2.345195	1.684706	0.040037
5	6	0	2.837930	2.978034	0.045939
6	6	0	1.896672	3.987469	0.151081
7	7	0	0.753527	0.095975	0.107963
8	6	0	1.969395	-0.488220	-0.003298
9	7	0	2.962210	0.436180	-0.048120
10	6	0	2.086947	-1.904340	-0.057018
11	6	0	0.914260	-2.682755	0.010792
12	6	0	-0.307570	-2.078963	0.127073
13	6	0	-0.444308	-0.644127	0.181354
14	6	0	-1.521399	-2.834770	0.196246
15	6	0	-2.725653	-2.246578	0.320225
16	6	0	-2.855921	-0.806337	0.394216
17	6	0	-1.649913	-0.030908	0.298253
18	7	0	-4.033459	-0.227988	0.543812
19	6	0	3.415636	-2.293936	-0.170594
20	7	0	4.581746	-2.251054	-0.258163
21	1	0	-0.195792	4.468833	0.325715
22	1	0	3.894777	3.200850	-0.026440
23	1	0	2.204511	5.022670	0.162802
24	1	0	0.986195	-3.761603	-0.028582
25	1	0	-1.453678	-3.913365	0.139649
26	1	0	-3.599578	-2.873865	0.348592
27	1	0	-1.673579	1.041310	0.330728
28	6	0	-5.317260	-0.949592	0.610936
29	6	0	-6.095316	-0.727243	-0.683656
30	6	0	-6.323249	0.763601	-0.916677
31	6	0	-5.010092	1.537357	-0.839920
32	6	0	-4.268521	1.223630	0.459911
33	1	0	-5.863025	-0.525606	1.455777
34	1	0	-5.163168	-1.998348	0.825270
35	1	0	-7.043592	-1.259258	-0.615941
36	1	0	-5.534599	-1.163843	-1.514365
37	1	0	-7.009560	1.144372	-0.155729
38	1	0	-6.800016	0.926793	-1.881699
39	1	0	-5.189664	2.611117	-0.885460
40	1	0	-4.365194	1.277870	-1.683626
41	1	0	-4.882174	1.492593	1.321385
42	1	0	-3.340675	1.775208	0.535872
43	30	0	4.735141	-0.293661	-0.200412

Ligand	Description	Total Gibbs Free energy (M06-2X/Def2TZVPP)
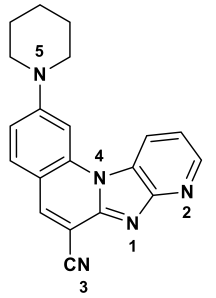 3b	H^+ bound to N1	-1046.184791 a.u.

CARTESIAN COORDINATES

Center Number	Atomic Number	Atomic Type	Coordinates (Angstroms) X	Y	Z
1	7	0	-1.462409	0.012701	0.084029
2	6	0	-0.272108	-0.715009	0.217154
3	6	0	-2.655642	-0.618351	-0.112363
4	7	0	-3.630305	0.292487	-0.224783
5	6	0	-3.088435	1.555689	-0.089742
6	6	0	-1.711483	1.400851	0.113443
7	6	0	-2.772725	-2.016469	-0.164354
8	6	0	-0.960876	2.553795	0.307735
9	7	0	-3.763433	2.678314	-0.135850
10	6	0	-1.654615	3.752084	0.263323
11	6	0	-3.032636	3.773835	0.038407
12	1	0	-1.126705	4.682942	0.406998
13	1	0	-3.565512	4.714814	0.002699
14	6	0	-0.384611	-2.129015	0.177099
15	6	0	-1.621481	-2.753072	-0.008393
16	1	0	-1.672910	-3.833622	-0.038243
17	6	0	0.950271	-0.094454	0.357843
18	6	0	0.813602	-2.873290	0.308910
19	6	0	2.020263	-2.275238	0.461886
20	6	0	2.137856	-0.848424	0.501452
21	1	0	0.748850	-3.953363	0.290829
22	1	0	2.891459	-2.896896	0.579972
23	7	0	3.330320	-0.252333	0.689578
24	1	0	1.008696	0.972246	0.307892
25	1	0	0.098565	2.563586	0.496001
26	6	0	-4.072038	-2.564877	-0.368320
27	7	0	-5.146914	-2.930888	-0.535232
28	6	0	4.610870	-0.893518	0.373432
29	6	0	5.075062	-0.438307	-1.010672
30	1	0	5.327694	-0.584538	1.136667
31	1	0	4.531601	-1.972007	0.425177
32	6	0	5.165591	1.085297	-1.086605
33	1	0	6.041768	-0.890887	-1.231484
34	1	0	4.364367	-0.809526	-1.754319
35	6	0	3.865719	1.745188	-0.623733
36	1	0	5.982135	1.427374	-0.444301
37	1	0	5.410047	1.397895	-2.100882
38	6	0	3.481433	1.200228	0.749324
39	1	0	3.981066	2.828025	-0.573048
40	1	0	3.061506	1.530869	-1.333992
41	1	0	4.284621	1.403138	1.461166
42	1	0	2.586105	1.657424	1.160170
43	1	0	-4.608261	0.078902	-0.370472

Ligand	Description	Total Gibbs Free energy (M06-2X/Def2TZVPP)
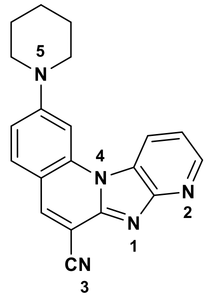 3b	Ca^{2+} bound to N1	-1722,9772714 a.u.

CARTESIAN COORDINATES

Center Number	Atomic Number	Atomic Type	Coordinates (Angstroms) X	Y	Z
1	6	0	-1.341907	3.729490	-0.248953
2	6	0	-0.543001	2.592762	-0.264515
3	6	0	-1.207145	1.379449	-0.149393
4	6	0	-2.602189	1.416317	-0.030417
5	7	0	-3.378388	2.483049	-0.013885
6	6	0	-2.730336	3.643099	-0.124589
7	7	0	-0.879333	0.005648	-0.115066
8	6	0	-2.059268	-0.667253	0.016310
9	7	0	-3.111986	0.145470	0.071157
10	6	0	-2.123774	-2.077969	0.080308
11	6	0	-0.936275	-2.796374	0.004658
12	6	0	0.265034	-2.121597	-0.130880
13	6	0	0.339067	-0.688436	-0.196596
14	6	0	1.503670	-2.822174	-0.204212
15	6	0	2.687838	-2.188601	-0.338830
16	6	0	2.762780	-0.751913	-0.426427
17	6	0	1.530805	-0.033938	-0.327384
18	6	0	-3.470487	-2.462026	0.211207
19	7	0	-4.623539	-2.406993	0.306858
20	1	0	-0.885254	4.704217	-0.334877
21	1	0	-3.333051	4.541064	-0.114944
22	1	0	-0.941806	-3.877413	0.050799
23	1	0	1.481065	-3.902660	-0.145925
24	1	0	3.582635	-2.784510	-0.379897
25	1	0	1.534594	1.036650	-0.350493
26	1	0	0.525246	2.695799	-0.362710
27	7	0	3.924197	-0.128697	-0.596136
28	6	0	5.238042	-0.790135	-0.539320
29	6	0	5.902598	-0.474719	0.799386
30	6	0	6.034824	1.034702	0.990822
31	6	0	4.696856	1.740782	0.776611
32	6	0	4.091345	1.329327	-0.564362
33	1	0	5.829771	-0.382950	-1.361007
34	1	0	5.148594	-1.854475	-0.709760
35	1	0	6.880517	-0.954103	0.829756
36	1	0	5.301231	-0.909535	1.602337
37	1	0	6.761079	1.423941	0.272257
38	1	0	6.423430	1.256718	1.983328
39	1	0	4.825390	2.822863	0.793334
40	1	0	3.997474	1.479310	1.575713
41	1	0	4.771806	1.586550	-1.378086
42	1	0	3.155006	1.834279	-0.769041
43	20	0	-5.340263	-0.167493	0.261409

Ligand	Description	Total Gibbs Free energy (M06-2X/Def2TZVPP)
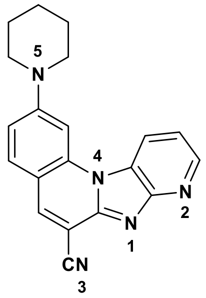 **3b**	Cu^{2+} bound to N1	-2685,7476673 a.u.

CARTESIAN COORDINATES

Center Number	Atomic Number	Atomic Type	Coordinates (Angstroms) X	Y	Z
1	6	0	-1.108569	3.872559	-0.248219
2	6	0	-0.342888	2.725608	-0.255498
3	6	0	-1.044315	1.524856	-0.155649
4	6	0	-2.446440	1.578139	-0.055842
5	7	0	-3.183490	2.675291	-0.048227
6	6	0	-2.511537	3.807230	-0.144481
7	7	0	-0.726230	0.160731	-0.121767
8	6	0	-1.929012	-0.499796	-0.004930
9	7	0	-2.961660	0.306532	0.036174
10	6	0	-1.970077	-1.931005	0.062340
11	6	0	-0.818302	-2.647497	0.008528
12	6	0	0.422386	-1.964674	-0.116866
13	6	0	0.479054	-0.542221	-0.178379
14	6	0	1.631451	-2.692193	-0.192500
15	6	0	2.833348	-2.073732	-0.336181
16	6	0	2.899690	-0.646868	-0.388910
17	6	0	1.692887	0.097053	-0.303324
18	6	0	-3.301414	-2.439388	0.182815
19	7	0	-4.438761	-2.588463	0.272999
20	1	0	-0.632548	4.839312	-0.323135
21	1	0	-3.094282	4.719401	-0.140677
22	1	0	-0.830936	-3.727987	0.055410
23	1	0	1.588427	-3.771186	-0.126440
24	1	0	3.731878	-2.668501	-0.354747
25	1	0	1.716491	1.164702	-0.401604
26	1	0	0.728648	2.806272	-0.336227
27	7	0	4.087680	-0.021148	-0.521779
28	6	0	5.332152	-0.718371	-0.866666
29	6	0	6.237242	-0.795621	0.370162
30	6	0	6.516286	0.607307	0.894971
31	6	0	5.218571	1.369528	1.128012
32	6	0	4.334785	1.370562	-0.134642
33	1	0	5.812903	-0.107143	-1.633588
34	1	0	5.115453	-1.684954	-1.303531
35	1	0	7.156864	-1.302706	0.081403
36	1	0	5.748729	-1.406766	1.133223
37	1	0	7.082556	0.553525	1.823955
38	1	0	7.136464	1.148315	0.176265
39	1	0	5.410359	2.409275	1.390927
40	1	0	4.650620	0.922573	1.947202
41	1	0	4.854135	1.854942	-0.962573
42	1	0	3.405368	1.890969	0.057443
43	29	0	-4.888817	-0.392396	0.207118

Ligand	Description	Total Gibbs Free energy (M06-2X/Def2TZVPP)
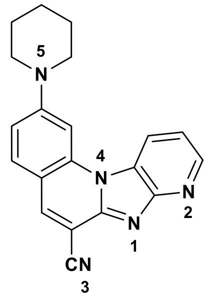 3b	Mg^{2+} bound to N1	-1245.2484733 a.u.

CARTESIAN COORDINATES

Center Number	Atomic Number	Atomic Type	Coordinates (Angstroms) X	Y	Z
1	6	0	-1.510294	3.749399	-0.220376
2	6	0	-0.748323	2.590485	-0.241434
3	6	0	-1.448731	1.396404	-0.123609
4	6	0	-2.839200	1.473701	0.002416
5	7	0	-3.578074	2.560109	0.024756
6	6	0	-2.900583	3.700437	-0.087632
7	7	0	-1.148261	0.014486	-0.093826
8	6	0	-2.334770	-0.639461	0.041362
9	7	0	-3.373627	0.202889	0.103807
10	6	0	-2.407283	-2.054337	0.100859
11	6	0	-1.221196	-2.787852	0.019111
12	6	0	-0.020645	-2.128336	-0.118154
13	6	0	0.064788	-0.692311	-0.182266
14	6	0	1.213478	-2.843181	-0.194903
15	6	0	2.401662	-2.222338	-0.329883
16	6	0	2.488801	-0.783883	-0.417344
17	6	0	1.260478	-0.052330	-0.315870
18	6	0	-3.745905	-2.431278	0.236582
19	7	0	-4.901112	-2.345353	0.338104
20	1	0	-1.025176	4.710219	-0.308174
21	1	0	-3.480732	4.613211	-0.072594
22	1	0	-1.242313	-3.868661	0.064333
23	1	0	1.178336	-3.923464	-0.139864
24	1	0	3.290573	-2.826446	-0.377197
25	1	0	1.279646	1.017364	-0.333317
26	1	0	0.321254	2.662763	-0.345950
27	7	0	3.651081	-0.171202	-0.586913
28	6	0	4.964341	-0.837322	-0.523185
29	6	0	5.644811	-0.477536	0.796626
30	6	0	5.786340	1.036038	0.937253
31	6	0	4.448329	1.739344	0.717628
32	6	0	3.826217	1.287917	-0.602595
33	1	0	5.546856	-0.462448	-1.366347
34	1	0	4.868069	-1.906749	-0.650509
35	1	0	6.620458	-0.961240	0.829750
36	1	0	5.051912	-0.883717	1.620448
37	1	0	6.505077	1.399287	0.197977
38	1	0	6.187824	1.287638	1.917404
39	1	0	4.579695	2.820905	0.696141
40	1	0	3.757953	1.507227	1.533397
41	1	0	4.500382	1.512319	-1.431076
42	1	0	2.890707	1.790362	-0.816275
43	12	0	-5.242872	-0.371369	0.292737

Ligand	Description	Total Gibbs Free energy (M06-2X/Def2TZVPP)
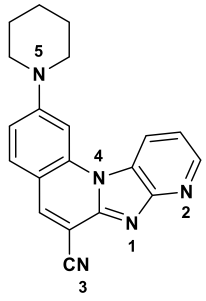<br>**3b**	Zn^{2+} bound to N1	-2824,6217221 a.u.

CARTESIAN COORDINATES

Center Number	Atomic Number	Atomic Type	Coordinates (Angstroms) X	Y	Z
1	6	0	-0.943866	3.819013	-0.253882
2	6	0	-0.221221	2.635943	-0.270939
3	6	0	-0.960227	1.464336	-0.156345
4	6	0	-2.347435	1.590540	-0.039607
5	7	0	-3.049246	2.697135	-0.021215
6	6	0	-2.335998	3.815038	-0.128540
7	7	0	-0.698017	0.072697	-0.126984
8	6	0	-1.899229	-0.554144	-0.002494
9	7	0	-2.909033	0.329228	0.054615
10	6	0	-2.004729	-1.968322	0.049605
11	6	0	-0.829694	-2.726676	-0.029896
12	6	0	0.383471	-2.095594	-0.154106
13	6	0	0.501537	-0.660196	-0.207246
14	6	0	1.602431	-2.839491	-0.224663
15	6	0	2.805016	-2.245709	-0.342098
16	6	0	2.925817	-0.807766	-0.415890
17	6	0	1.712017	-0.047846	-0.321904
18	6	0	-3.343963	-2.341674	0.175005
19	7	0	-4.503365	-2.257964	0.271144
20	1	0	-0.426579	4.763173	-0.339543
21	1	0	-2.887984	4.745204	-0.115976
22	1	0	-0.877279	-3.806875	0.008826
23	1	0	1.540756	-3.919003	-0.179113
24	1	0	3.680401	-2.869489	-0.386381
25	1	0	1.758707	1.020713	-0.327200
26	1	0	0.850182	2.674090	-0.371673
27	7	0	4.101800	-0.220755	-0.565937
28	6	0	5.400364	-0.916126	-0.494939
29	6	0	6.080566	-0.571220	0.829263
30	6	0	6.254850	0.938554	0.972236
31	6	0	4.933737	1.670800	0.745711
32	6	0	4.310076	1.234786	-0.578866
33	1	0	5.995238	-0.554000	-1.334942
34	1	0	5.281063	-1.983183	-0.621760
35	1	0	7.044850	-1.076832	0.866770
36	1	0	5.474504	-0.965223	1.649368
37	1	0	6.985816	1.286851	0.237839
38	1	0	6.655683	1.180057	1.955148
39	1	0	5.088216	2.749279	0.725401
40	1	0	4.233615	1.453339	1.557168
41	1	0	4.994575	1.443518	-1.402874
42	1	0	3.387592	1.758194	-0.798455
43	30	0	-4.717182	-0.286923	0.223120

Ligand	Description	Total Gibbs Free energy (M06-2X/Def2TZVPP)
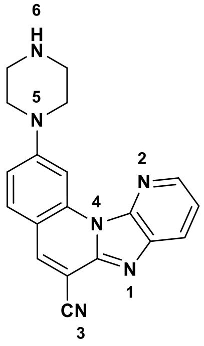<br>**4a**	H^+ bound to N1	-1062.235739 a.u.

CARTESIAN COORDINATES

Center Number	Atomic Number	Atomic Type	Coordinates (Angstroms) X	Y	Z
1	7	0	-1.597001	0.042683	0.014516
2	6	0	-0.409889	-0.701991	0.023397
3	6	0	-2.817279	-0.550551	-0.033804
4	7	0	-3.775976	0.393012	-0.031565
5	6	0	-3.184369	1.640619	0.017328
6	6	0	-1.803459	1.438240	0.046150
7	6	0	-2.971716	-1.944143	-0.079942
8	7	0	-0.891456	2.381247	0.092756
9	6	0	-3.681117	2.931970	0.039684
10	6	0	-1.368223	3.624072	0.114080
11	6	0	-2.730215	3.936447	0.089527
12	1	0	-0.631705	4.415083	0.153472
13	1	0	-3.034253	4.972490	0.110097
14	1	0	-4.740485	3.144727	0.019565
15	6	0	-0.558407	-2.114089	-0.015614
16	6	0	-1.822301	-2.706445	-0.068916
17	1	0	-1.902504	-3.785369	-0.098784
18	6	0	0.822068	-0.091766	0.084499
19	6	0	0.633856	-2.878319	0.011999
20	6	0	1.856993	-2.295441	0.072062
21	6	0	2.002319	-0.869665	0.097560
22	1	0	0.554154	-3.956888	-0.024590
23	1	0	2.725253	-2.932074	0.065669
24	7	0	3.219572	-0.288766	0.115330
25	1	0	0.857753	0.978386	0.157647
26	6	0	-4.299869	-2.456174	-0.131266
27	7	0	-5.401111	-2.778062	-0.170603
28	6	0	4.415857	-0.972494	0.623305
29	6	0	5.660747	-0.545571	-0.133765
30	1	0	4.534747	-0.704543	1.678000
31	1	0	4.298056	-2.046158	0.555709
32	7	0	5.786833	0.895530	-0.038259
33	1	0	6.525477	-1.020835	0.326912
34	1	0	5.585546	-0.897146	-1.173537
35	6	0	4.660041	1.536364	-0.684357
36	1	0	6.662638	1.214845	-0.428263
37	6	0	3.374718	1.165947	0.041739
38	1	0	4.777904	2.618176	-0.640815
39	1	0	4.557930	1.244486	-1.740380
40	1	0	3.386164	1.583660	1.053833
41	1	0	2.541453	1.590688	-0.509067
42	1	0	-4.762686	0.178894	-0.062699

Ligand	Description	Total Gibbs Free energy (M06-2X/Def2TZVPP)
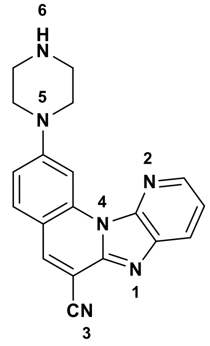 **4a**	Ca^{2+} bound to N1	-1738,9820478 a.u.

CARTESIAN COORDINATES

Center Number	Atomic Number	Atomic Type	Coordinates (Angstroms) X	Y	Z
1	6	0	0.799102	3.643199	0.218818
2	7	0	0.303069	2.407592	0.207122
3	6	0	1.197289	1.450710	0.118069
4	6	0	2.583405	1.610043	0.035475
5	6	0	3.087387	2.902050	0.048454
6	6	0	2.164679	3.929682	0.142644
7	7	0	0.967074	0.063936	0.084941
8	6	0	2.187462	-0.526787	-0.014598
9	7	0	3.202287	0.360283	-0.047805
10	6	0	2.282301	-1.941931	-0.072634
11	6	0	1.113276	-2.708498	-0.018937
12	6	0	-0.113291	-2.094984	0.086394
13	6	0	-0.235284	-0.664573	0.138969
14	6	0	-1.329576	-2.837835	0.152905
15	6	0	-2.532227	-2.237712	0.266307
16	6	0	-2.651187	-0.799396	0.318863
17	6	0	-1.440575	-0.039006	0.244396
18	7	0	-3.832185	-0.205679	0.435659
19	6	0	3.605978	-2.363258	-0.177358
20	7	0	4.763492	-2.420120	-0.257965
21	1	0	0.079367	4.447061	0.292663
22	1	0	4.147085	3.121045	-0.010963
23	1	0	2.490045	4.959413	0.158189
24	1	0	1.174901	-3.788228	-0.057451
25	1	0	-1.272415	-3.917433	0.103060
26	1	0	-3.410365	-2.859013	0.290997
27	1	0	-1.450402	1.032383	0.300696
28	6	0	-4.054401	1.243225	0.315071
29	6	0	-5.009952	1.510927	-0.845256
30	7	0	-6.251332	0.808071	-0.604087
31	6	0	-6.049971	-0.624068	-0.526080
32	6	0	-5.109885	-0.907648	0.638421
33	1	0	-4.504340	1.589437	1.246792
34	1	0	-3.123076	1.770069	0.156043
35	1	0	-5.200669	2.581873	-0.895010
36	1	0	-4.513922	1.205908	-1.779563
37	1	0	-6.960955	1.053458	-1.280059
38	1	0	-7.001620	-1.117073	-0.334906
39	1	0	-5.617462	-1.052275	-1.443198
40	1	0	-5.547336	-0.505413	1.553873
41	1	0	-4.957486	-1.968991	0.775069
42	20	0	5.431732	-0.245782	-0.211041

Ligand	Description	Total Gibbs Free energy (M06-2X/Def2TZVPP)
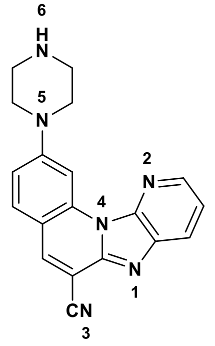 4a	Cu^{2+} bound to N1	-2701,7646690 a.u.

CARTESIAN COORDINATES

Center Number	Atomic Number	Atomic Type	Coordinates (Angstroms) X	Y	Z
1	6	0	1.645310	2.614651	-1.000417
2	7	0	0.816265	1.609181	-0.731550
3	6	0	1.390407	0.436474	-0.535553
4	6	0	2.783895	0.160055	-0.561274
5	6	0	3.646328	1.209152	-0.901447
6	6	0	3.045912	2.469254	-1.115434
7	7	0	0.794815	-0.784460	-0.277233
8	6	0	1.852181	-1.685659	-0.144535
9	7	0	3.033952	-1.147415	-0.305127
10	6	0	1.562191	-3.062355	0.142200
11	6	0	0.270430	-3.457634	0.240006
12	6	0	-0.804879	-2.530666	0.057139
13	6	0	-0.548238	-1.170303	-0.211418
14	6	0	-2.142166	-2.953108	0.109190
15	6	0	-3.175375	-2.079427	-0.095773
16	6	0	-2.909036	-0.717703	-0.359121
17	6	0	-1.582167	-0.269150	-0.417125
18	7	0	-3.964956	0.154149	-0.598944
19	6	0	2.660320	-3.960357	0.311660
20	7	0	3.535482	-4.685848	0.457777
21	1	0	1.194274	3.582626	-1.174546
22	1	0	4.700526	1.029517	-1.084042
23	1	0	3.633097	3.303622	-1.480343
24	1	0	0.037976	-4.493201	0.450360
25	1	0	-2.351868	-3.999089	0.288332
26	1	0	-4.183897	-2.461035	-0.113416
27	1	0	-1.334475	0.761198	-0.609233
28	6	0	-5.236294	-0.011533	0.041634
29	6	0	-5.232639	0.939541	1.323237
30	7	0	-4.935887	2.267885	0.939174
31	6	0	-3.750547	2.456280	0.186739
32	6	0	-3.764146	1.489925	-1.073456
33	1	0	-6.035349	0.304984	-0.625335
34	1	0	-5.406158	-1.026531	0.378192
35	1	0	-6.205004	0.874642	1.804524
36	1	0	-4.462284	0.548239	1.991766
37	1	0	-5.722133	2.854955	0.698182
38	1	0	-2.874532	2.188177	0.783029
39	1	0	-3.662782	3.484221	-0.154996
40	1	0	-2.846339	1.584166	-1.642669
41	1	0	-4.606680	1.772435	-1.702006
42	29	0	3.686173	2.363173	1.028483

Ligand	Description	Total Gibbs Free energy (M06-2X/Def2TZVPP)
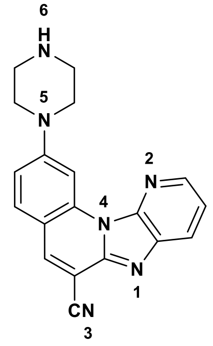<br>**4a**	Mg^{2+} bound to N1	-1261,3096341 a.u.

CARTESIAN COORDINATES

Center Number	Atomic Number	Atomic Type	Coordinates (Angstroms) X	Y	Z
1	6	0	1.369102	3.735989	0.578976
2	7	0	0.905638	2.491270	0.703001
3	6	0	1.721857	1.573571	0.238985
4	6	0	2.977787	1.761569	-0.342230
5	6	0	3.452963	3.066238	-0.456764
6	6	0	2.614761	4.056672	0.017028
7	7	0	1.510404	0.168853	0.262316
8	6	0	2.692319	-0.352359	-0.415431
9	7	0	3.553120	0.553081	-0.701087
10	6	0	2.784337	-1.800646	-0.497151
11	6	0	1.657101	-2.559866	-0.204472
12	6	0	0.416533	-1.972748	0.217892
13	6	0	0.258893	-0.536022	0.219937
14	6	0	-0.792556	-2.731285	0.340205
15	6	0	-2.001703	-2.137539	0.419962
16	6	0	-2.154336	-0.697711	0.370287
17	6	0	-0.942735	0.083287	0.312351
18	7	0	-3.339912	-0.121430	0.394451
19	6	0	4.005102	-2.394450	-0.938259
20	7	0	4.987555	-2.890109	-1.259887
21	1	0	0.723206	4.522499	0.945872
22	1	0	4.418647	3.278977	-0.893930
23	1	0	2.908132	5.095068	-0.038545
24	1	0	1.718346	-3.639095	-0.291328
25	1	0	-0.728998	-3.812146	0.318887
26	1	0	-2.869074	-2.772430	0.472482
27	1	0	-0.955673	1.156420	0.385823
28	6	0	-3.577413	1.314424	0.152576
29	6	0	-4.451373	1.472215	-1.091818
30	7	0	-5.691520	0.762362	-0.882173
31	6	0	-5.476646	-0.654387	-0.690496
32	6	0	-4.621221	-0.835924	0.558914
33	1	0	-4.101725	1.710886	1.022469
34	1	0	-2.645399	1.847929	0.022774
35	1	0	-4.653351	2.532713	-1.233005
36	1	0	-3.883213	1.111626	-1.963072
37	1	0	-6.370558	0.954318	-1.605314
38	1	0	-6.430643	-1.153612	-0.530514
39	1	0	-4.971953	-1.139606	-1.539878
40	1	0	-5.125937	-0.372052	1.407409
41	1	0	-4.461749	-1.880750	0.785552
42	12	0	2.029694	-1.377924	1.697919

Ligand	Description	Total Gibbs Free energy (M06-2X/Def2TZVPP)
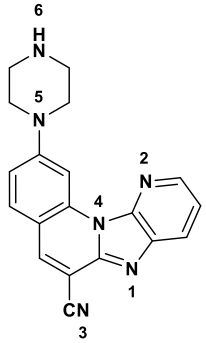 4a	Zn^{2+} bound to N1	-2840,6650979 a.u.

CARTESIAN COORDINATES

Center Number	Atomic Number	Atomic Type	Coordinates (Angstroms) X	Y	Z
1	6	0	0.701109	3.510283	-0.055785
2	7	0	0.150591	2.299942	-0.039967
3	6	0	1.019589	1.304037	-0.029622
4	6	0	2.424012	1.412264	-0.032295
5	6	0	2.971522	2.690119	-0.049373
6	6	0	2.080311	3.747314	-0.061228
7	7	0	0.764052	-0.061391	-0.011221
8	6	0	2.019593	-0.666524	-0.001912
9	7	0	3.016318	0.175962	-0.014956
10	6	0	2.080953	-2.102207	0.024790
11	6	0	0.927681	-2.816655	0.040136
12	6	0	-0.345889	-2.176734	0.026521
13	6	0	-0.435892	-0.769914	0.001052
14	6	0	-1.547310	-2.895280	0.041112
15	6	0	-2.767127	-2.267726	0.028459
16	6	0	-2.852557	-0.855620	-0.014014
17	6	0	-1.659426	-0.122428	-0.019747
18	7	0	-4.072438	-0.205894	-0.073796
19	6	0	3.358949	-2.742486	0.037010
20	7	0	4.365945	-3.292453	0.048129
21	1	0	0.011184	4.344728	-0.064752
22	1	0	4.042749	2.835717	-0.052602
23	1	0	2.435963	4.767583	-0.074709
24	1	0	0.967903	-3.897907	0.061636
25	1	0	-1.503205	-3.976482	0.078683
26	1	0	-3.658326	-2.871605	0.075293
27	1	0	-1.656705	0.951207	-0.073180
28	6	0	-4.223938	1.072933	0.621697
29	6	0	-5.419331	1.843261	0.093830
30	7	0	-6.609465	1.019591	0.221609
31	6	0	-6.467640	-0.176486	-0.588759
32	6	0	-5.293218	-0.999796	-0.086149
33	1	0	-4.362578	0.890909	1.695331
34	1	0	-3.331363	1.675085	0.492083
35	1	0	-5.540996	2.751272	0.683675
36	1	0	-5.220134	2.130736	-0.949367
37	1	0	-7.434962	1.537253	-0.046933
38	1	0	-7.373900	-0.776847	-0.513656
39	1	0	-6.295362	0.057780	-1.650090
40	1	0	-5.520059	-1.385587	0.917266
41	1	0	-5.159392	-1.842098	-0.761076
42	30	0	4.895527	0.377594	-0.019350

Ligand	Description	Total Gibbs Free energy (M06-2X/Def2TZVPP)
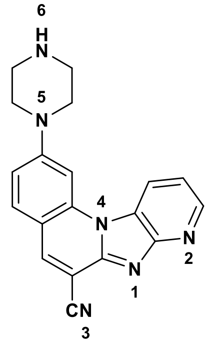 4b	H^+ bound to N1	-1062.229635 a.u.

CARTESIAN COORDINATES

Center Number	Atomic Number	Atomic Type	Coordinates (Angstroms) X	Y	Z
1	7	0	1.571256	0.036608	-0.003360
2	6	0	0.381522	-0.703182	0.013093
3	6	0	2.785551	-0.583100	-0.000351
4	7	0	3.757801	0.335858	-0.039776
5	6	0	3.190368	1.595139	-0.055702
6	6	0	1.800485	1.428176	-0.026037
7	6	0	2.921257	-1.980739	0.053065
8	6	0	1.018708	2.576743	0.000476
9	7	0	3.854433	2.725099	-0.080821
10	6	0	1.700248	3.782168	-0.025240
11	6	0	3.095969	3.815341	-0.070213
12	1	0	1.148938	4.710369	-0.007258
13	1	0	3.619184	4.762126	-0.093067
14	6	0	0.509883	-2.113225	0.077745
15	6	0	1.768144	-2.726356	0.100855
16	1	0	1.833119	-3.805608	0.146656
17	6	0	-0.855966	-0.096590	-0.056835
18	6	0	-0.687159	-2.869512	0.089451
19	6	0	-1.908898	-2.283310	0.034099
20	6	0	-2.041887	-0.860838	-0.033818
21	1	0	-0.610867	-3.947134	0.151591
22	1	0	-2.782905	-2.911109	0.074031
23	7	0	-3.258464	-0.270306	-0.056946
24	1	0	-0.917959	0.962350	-0.194838
25	1	0	-0.056290	2.577522	0.049669
26	6	0	4.241663	-2.517402	0.059942
27	7	0	5.332588	-2.873536	0.061991
28	6	0	-4.435655	-0.947118	-0.621259
29	6	0	-5.718572	-0.469321	0.033106
30	1	0	-4.478008	-0.719506	-1.691298
31	1	0	-4.345277	-2.019533	-0.503398
32	7	0	-5.799436	0.971370	-0.109150
33	1	0	-6.559848	-0.936538	-0.476305
34	1	0	-5.728085	-0.792016	1.084618
35	6	0	-4.713005	1.598991	0.612558
36	1	0	-6.695345	1.322799	0.198993
37	6	0	-3.384794	1.186289	-0.005724
38	1	0	-4.801143	2.682256	0.543016
39	1	0	-4.699511	1.323278	1.677600
40	1	0	-3.299661	1.604245	-1.015415
41	1	0	-2.589331	1.586463	0.618650
42	1	0	4.748565	0.131469	-0.042083

Ligand	Description	Total Gibbs Free energy (M06-2X/Def2TZVPP)
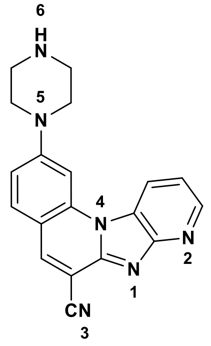 4b	Ca^{2+} bound to N1	-1738,9304492 a.u.

CARTESIAN COORDINATES

Center Number	Atomic Number	Atomic Type	Coordinates (Angstroms) X	Y	Z
1	6	0	1.405899	3.739901	0.216216
2	6	0	0.601502	2.607067	0.206023
3	6	0	1.263584	1.390567	0.114110
4	6	0	2.661912	1.420785	0.041989
5	7	0	3.443387	2.483788	0.050970
6	6	0	2.797438	3.646898	0.138881
7	7	0	0.930703	0.018221	0.068598
8	6	0	2.111151	-0.659944	-0.024890
9	7	0	3.168841	0.147590	-0.043264
10	6	0	2.170903	-2.070934	-0.092439
11	6	0	0.979368	-2.784071	-0.049700
12	6	0	-0.223176	-2.103781	0.049744
13	6	0	-0.292913	-0.670115	0.102074
14	6	0	-1.464135	-2.800920	0.113379
15	6	0	-2.648590	-2.162307	0.222118
16	6	0	-2.721278	-0.724481	0.259855
17	6	0	-1.486113	-0.010813	0.195475
18	6	0	3.519615	-2.460744	-0.183790
19	7	0	4.675101	-2.409871	-0.244264
20	1	0	0.951276	4.716847	0.286327
21	1	0	3.404317	4.542052	0.149166
22	1	0	0.981903	-3.865425	-0.088414
23	1	0	-1.443736	-3.881767	0.061378
24	1	0	-3.548996	-2.751953	0.235047
25	1	0	-1.487160	1.057482	0.277593
26	1	0	-0.468859	2.715106	0.269558
27	7	0	-3.888955	-0.092707	0.350135
28	6	0	-4.066198	1.349916	0.141071
29	6	0	-5.163354	1.587632	-0.894640
30	7	0	-6.386104	0.959342	-0.447268
31	6	0	-6.226070	-0.475459	-0.326520
32	6	0	-5.158111	-0.739992	0.723406
33	1	0	-4.348539	1.807764	1.091421
34	1	0	-3.146766	1.796745	-0.217793
35	1	0	-5.316829	2.661554	-0.990242
36	1	0	-4.810694	1.203153	-1.863916
37	1	0	-7.170315	1.203964	-1.035510
38	1	0	-5.930514	-0.958493	-1.269968
39	1	0	-7.161185	-0.921432	0.007754
40	1	0	-5.017919	-1.799177	0.890107
41	1	0	-5.473215	-0.286782	1.666132
42	20	0	5.400891	-0.172971	-0.166358

Ligand	Description	Total Gibbs Free energy (M06-2X/Def2TZVPP)
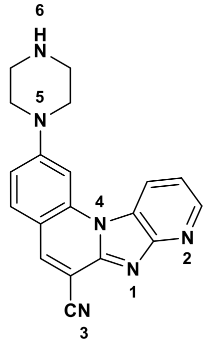 **4b**	Cu^{2+} bound to N1	-2701,7188549 a.u.

CARTESIAN COORDINATES

Center Number	Atomic Number	Atomic Type	Coordinates (Angstroms) X	Y	Z
1	6	0	-2.715151	3.756372	0.055088
2	6	0	-1.912618	2.623807	-0.025475
3	6	0	-2.587237	1.423496	0.026482
4	6	0	-4.027217	1.421498	0.130551
5	7	0	-4.786005	2.535834	0.215398
6	6	0	-4.139947	3.661770	0.181250
7	7	0	-2.236555	0.066373	-0.032889
8	6	0	-3.439447	-0.615875	0.012442
9	7	0	-4.510543	0.191846	0.117613
10	6	0	-3.474699	-2.015013	-0.063723
11	6	0	-2.279749	-2.691147	-0.191446
12	6	0	-1.051688	-1.998465	-0.221073
13	6	0	-1.016000	-0.586421	-0.120899
14	6	0	0.169471	-2.699827	-0.334210
15	6	0	1.359512	-2.035239	-0.335280
16	6	0	1.387999	-0.631023	-0.214209
17	6	0	0.210338	0.087089	-0.109057
18	6	0	-4.727368	-2.698383	-0.018198
19	7	0	-5.711694	-3.284307	0.016512
20	1	0	-2.262964	4.737957	0.019516
21	1	0	-4.722704	4.572839	0.248656
22	1	0	-2.284440	-3.771410	-0.264795
23	1	0	0.143696	-3.778818	-0.409029
24	1	0	2.285965	-2.590686	-0.413706
25	1	0	0.235292	1.156227	-0.015728
26	1	0	-0.847053	2.735580	-0.134454
27	7	0	2.676540	0.003682	-0.198793
28	6	0	2.691180	1.433715	0.197198
29	6	0	4.137239	1.892460	0.332724
30	7	0	4.819954	1.707674	-0.910650
31	6	0	4.823761	0.344683	-1.350383
32	6	0	3.395113	-0.149710	-1.508440
33	1	0	2.200674	2.042663	-0.567303
34	1	0	2.167429	1.538558	1.147093
35	1	0	4.170341	2.936001	0.639359
36	1	0	4.615479	1.314196	1.172100
37	1	0	5.719076	2.164416	-0.963976
38	1	0	5.352345	-0.332335	-0.623229
39	1	0	5.358191	0.245264	-2.293108
40	1	0	3.379863	-1.196701	-1.801295
41	1	0	2.875160	0.439363	-2.267447
42	29	0	4.104511	-0.824260	1.152309

Ligand	Description	Total Gibbs Free energy (M06-2X/Def2TZVPP)
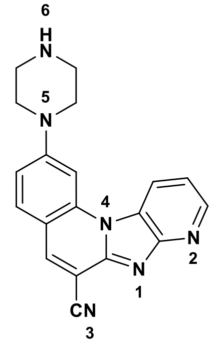 **4b**	Mg^{2+} bound to N1	-1261,2973510 a.u.

CARTESIAN COORDINATES

Center Number	Atomic Number	Atomic Type	Coordinates (Angstroms) X	Y	Z
1	6	0	1.605473	3.757780	0.222425
2	6	0	0.828158	2.608959	0.218911
3	6	0	1.515616	1.406469	0.109687
4	6	0	2.909588	1.466091	0.018182
5	7	0	3.662963	2.542733	0.020185
6	6	0	2.997874	3.691230	0.122412
7	7	0	1.198057	0.028661	0.065462
8	6	0	2.378997	-0.639948	-0.043617
9	7	0	3.429855	0.188845	-0.076236
10	6	0	2.434655	-2.055532	-0.108499
11	6	0	1.239042	-2.774421	-0.047411
12	6	0	0.043500	-2.099864	0.062976
13	6	0	-0.026162	-0.662516	0.110981
14	6	0	-1.198153	-2.801036	0.140175
15	6	0	-2.380841	-2.165445	0.256174
16	6	0	-2.454954	-0.724833	0.287401
17	6	0	-1.217759	-0.007812	0.208400
18	6	0	3.771402	-2.449241	-0.214514
19	7	0	4.929542	-2.377646	-0.288618
20	1	0	1.131026	4.724345	0.305418
21	1	0	3.590085	4.596349	0.126900
22	1	0	1.248460	-3.855785	-0.082515
23	1	0	-1.174506	-3.881974	0.092201
24	1	0	-3.279982	-2.756441	0.280304
25	1	0	-1.223395	1.060208	0.279771
26	1	0	-0.242283	2.694645	0.302045
27	7	0	-3.618365	-0.095671	0.387072
28	6	0	-3.806631	1.346888	0.173959
29	6	0	-4.866406	1.567848	-0.904502
30	7	0	-6.096733	0.927565	-0.498810
31	6	0	-5.928298	-0.504009	-0.360585
32	6	0	-4.894772	-0.749628	0.728844
33	1	0	-4.136339	1.792382	1.114176
34	1	0	-2.880904	1.811515	-0.141941
35	1	0	-5.027880	2.639658	-1.009597
36	1	0	-4.472671	1.184436	-1.858308
37	1	0	-6.864395	1.162610	-1.112267
38	1	0	-5.595721	-0.993492	-1.288348
39	1	0	-6.869902	-0.956280	-0.054300
40	1	0	-4.752234	-1.804902	0.915713
41	1	0	-5.240993	-0.284320	1.654001
42	12	0	5.296035	-0.408353	-0.222920

Ligand	Description	Total Gibbs Free energy (M06-2X/Def2TZVPP)
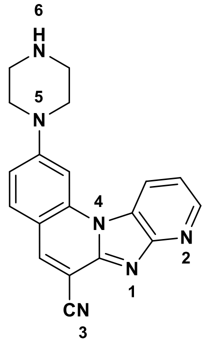 **4b**	Zn^{2+} bound to N1	-2840,6654363 a.u.

CARTESIAN COORDINATES

Center Number	Atomic Number	Atomic Type	Coordinates (Angstroms) X	Y	Z
1	6	0	1.040199	3.832987	0.267194
2	6	0	0.300080	2.660726	0.261792
3	6	0	1.023365	1.479677	0.142872
4	6	0	2.413829	1.586420	0.048788
5	7	0	3.132145	2.682541	0.052936
6	6	0	2.433852	3.809746	0.161035
7	7	0	0.741448	0.092270	0.096956
8	6	0	1.935317	-0.551044	-0.012524
9	7	0	2.958489	0.317746	-0.046957
10	6	0	2.021401	-1.966530	-0.070309
11	6	0	0.836169	-2.708710	0.001873
12	6	0	-0.370301	-2.060690	0.108108
13	6	0	-0.470130	-0.623442	0.141373
14	6	0	-1.597775	-2.788719	0.190154
15	6	0	-2.793416	-2.177888	0.293516
16	6	0	-2.898669	-0.737658	0.303375
17	6	0	-1.675029	0.005710	0.221886
18	6	0	3.357276	-2.358370	-0.176663
19	7	0	4.518934	-2.290241	-0.254841
20	1	0	0.535734	4.783629	0.357633
21	1	0	2.999579	4.731688	0.166747
22	1	0	0.870178	-3.789826	-0.023999
23	1	0	-1.549790	-3.869340	0.155552
24	1	0	-3.679722	-2.787693	0.322675
25	1	0	-1.706345	1.073887	0.274131
26	1	0	-0.771493	2.713470	0.353697
27	7	0	-4.074136	-0.133052	0.386868
28	6	0	-4.294266	1.302573	0.152535
29	6	0	-5.332877	1.482063	-0.954351
30	7	0	-6.555683	0.816706	-0.568048
31	6	0	-6.356419	-0.607846	-0.404225
32	6	0	-5.342589	-0.812102	0.712450
33	1	0	-4.660022	1.747014	1.079553
34	1	0	-3.373783	1.789380	-0.144102
35	1	0	-5.518025	2.548007	-1.078075
36	1	0	-4.906562	1.096014	-1.892971
37	1	0	-7.315911	1.025241	-1.200090
38	1	0	-5.990976	-1.103426	-1.316284
39	1	0	-7.293467	-1.078430	-0.111930
40	1	0	-5.179607	-1.860244	0.920980
41	1	0	-5.719046	-0.338886	1.621401
42	30	0	4.761026	-0.322790	-0.182886

Ligand	Description	Total Gibbs Free energy (M06-2X/Def2TZVPP)
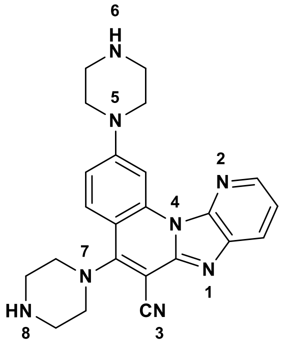 5a	H^+ bound to N1	-1328.851143 a.u.

CARTESIAN COORDINATES

Center Number	Atomic Number	Atomic Type	Coordinates (Angstroms) X	Y	Z
1	7	0	0.222526	1.531791	0.047856
2	6	0	-0.313236	0.236098	0.081448
3	6	0	1.547223	1.735334	0.242095
4	7	0	1.804129	3.059068	0.194196
5	6	0	0.624945	3.744314	-0.036742
6	6	0	-0.391684	2.790303	-0.126053
7	6	0	2.473326	0.702986	0.393743
8	7	0	-1.660042	3.044796	-0.337442
9	6	0	0.313524	5.081606	-0.180489
10	6	0	-1.962748	4.336858	-0.476595
11	6	0	-1.025314	5.365758	-0.406581
12	1	0	-3.005475	4.562624	-0.653611
13	1	0	-1.351026	6.387904	-0.529859
14	1	0	1.062042	5.858889	-0.121154
15	6	0	0.590201	-0.837412	0.244974
16	6	0	2.018491	-0.635737	0.317382
17	7	0	2.871470	-1.674649	0.319915
18	6	0	-1.681090	0.057102	-0.003572
19	6	0	-0.007696	-2.097849	0.465506
20	6	0	-1.354513	-2.293806	0.411712
21	6	0	-2.248139	-1.221299	0.134923
22	1	0	0.617615	-2.934246	0.735666
23	1	0	-1.734651	-3.276471	0.636834
24	7	0	-3.591693	-1.415331	0.072914
25	1	0	-2.289095	0.923238	-0.183521
26	6	0	3.826326	1.135569	0.382491
27	7	0	4.876404	1.603554	0.359067
28	6	0	-4.146262	-2.701527	-0.364797
29	6	0	-5.540469	-2.920389	0.192627
30	1	0	-4.197571	-2.705547	-1.459225
31	1	0	-3.502394	-3.514012	-0.050029
32	7	0	-6.379641	-1.806515	-0.205289
33	1	0	-5.938665	-3.845568	-0.221561
34	1	0	-5.474810	-3.030205	1.285386
35	6	0	-5.882477	-0.580042	0.385381
36	1	0	-7.344770	-1.967186	0.047504
37	6	0	-4.490048	-0.282565	-0.148587
38	1	0	-6.537715	0.247910	0.118325
39	1	0	-5.828870	-0.635408	1.483004
40	1	0	-4.543788	-0.052216	-1.218939
41	1	0	-4.105688	0.586182	0.379103
42	6	0	2.697478	-2.868748	-0.521056
43	6	0	3.899396	-3.003169	-1.450645
44	1	0	2.629096	-3.756786	0.111869
45	1	0	1.792334	-2.768073	-1.111019
46	7	0	5.113757	-3.082561	-0.663997

47	1	0	3.781407	-3.916492	-2.032844
48	1	0	3.895891	-2.149405	-2.145954
49	6	0	5.329852	-1.854867	0.083887
50	1	0	5.911191	-3.303419	-1.243780
51	6	0	4.156122	-1.678561	1.033425
52	1	0	6.245213	-1.939091	0.667860
53	1	0	5.414250	-0.970358	-0.561489
54	1	0	4.125718	-2.541104	1.704535
55	1	0	4.249039	-0.786237	1.642556
56	1	0	2.733110	3.444266	0.282960

Ligand	Description	Total Gibbs Free energy (M06-2X/Def2TZVPP)
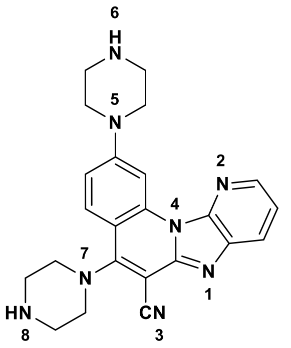 **5a**	Ca^{2+} bound to N1	-2005,5989526 a.u.

CARTESIAN COORDINATES

Center	Atomic	Atomic	Coordinates (Angstroms)		
Number	Number	Type	X	Y	Z
1	6	0	-1.328559	4.393214	0.675576
2	7	0	-1.323052	3.067558	0.538186
3	6	0	-0.153474	2.539027	0.266254
4	6	0	1.051463	3.230043	0.111163
5	6	0	1.032901	4.607770	0.252810
6	6	0	-0.190818	5.189851	0.542322
7	7	0	0.160148	1.181552	0.074052
8	6	0	1.485737	1.116449	-0.184601
9	7	0	2.081608	2.329741	-0.169556
10	6	0	2.130134	-0.135399	-0.383705
11	6	0	1.385218	-1.357563	-0.278069
12	6	0	-0.028030	-1.224263	-0.144666
13	6	0	-0.659096	0.032705	0.069411
14	6	0	-0.917470	-2.307507	-0.358259
15	6	0	-2.264425	-2.196492	-0.232779
16	6	0	-2.877148	-0.957990	0.144331
17	6	0	-2.013377	0.169002	0.220412
18	7	0	-4.188354	-0.856520	0.375382
19	6	0	3.501395	0.047844	-0.456954
20	7	0	4.583360	0.479208	-0.508216
21	1	0	-2.284574	4.845423	0.901850
22	1	0	1.918750	5.223479	0.142916
23	1	0	-0.275481	6.259462	0.664753
24	1	0	-0.517561	-3.255180	-0.685057
25	1	0	-2.864637	-3.061047	-0.458208
26	1	0	-2.398451	1.156099	0.386272
27	6	0	-5.137161	-1.972928	0.297084
28	6	0	-6.133217	-1.721747	-0.828252
29	7	0	-6.815627	-0.470424	-0.560971
30	6	0	-5.896045	0.648340	-0.506210
31	6	0	-4.896887	0.404059	0.620157
32	1	0	-4.623891	-2.916135	0.166526
33	1	0	-5.674904	-2.008263	1.246406
34	1	0	-5.589748	-1.720932	-1.785768
35	1	0	-6.859116	-2.533108	-0.848058
36	1	0	-7.565498	-0.306111	-1.217675
37	1	0	-6.450288	1.561816	-0.295401
38	1	0	-5.335807	0.792588	-1.443185
39	1	0	-5.436449	0.302678	1.563564
40	1	0	-4.207412	1.233775	0.708205
41	7	0	1.999754	-2.547166	-0.300852
42	6	0	3.272597	-2.791511	-0.988806
43	6	0	4.370996	-3.179231	-0.010280
44	7	0	3.917227	-4.354998	0.700862
45	6	0	2.714327	-4.083826	1.459093
46	6	0	1.579619	-3.704708	0.511554

47	1	0	3.535614	-1.938332	-1.608091
48	1	0	3.102712	-3.635492	-1.661555
49	1	0	4.584259	-2.329767	0.659537
50	1	0	5.278486	-3.411282	-0.565891
51	1	0	4.643767	-4.756953	1.276121
52	1	0	2.417624	-4.978144	2.005105
53	1	0	2.844042	-3.266878	2.186403
54	1	0	1.350462	-4.544827	-0.146902
55	1	0	0.701906	-3.439988	1.090484
56	20	0	4.331434	2.722682	-0.405504

Ligand	Description	Total Gibbs Free energy (M06-2X/Def2TZVPP)
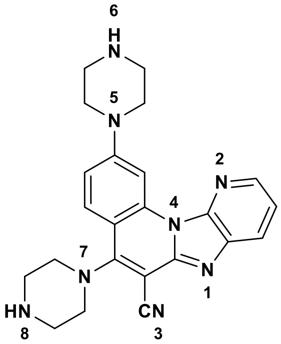 5a	Cu^{2+} bound to N1	-2968,3966235 a.u.

CARTESIAN COORDINATES

Center Number	Atomic Number	Atomic Type	Coordinates (Angstroms) X	Y	Z
1	6	0	-1.064300	4.500193	0.825001
2	7	0	-1.200024	3.179482	0.697342
3	6	0	-0.090190	2.546328	0.385593
4	6	0	1.168834	3.114003	0.172892
5	6	0	1.294701	4.488310	0.308558
6	6	0	0.143383	5.179702	0.643720
7	7	0	0.088132	1.166366	0.194023
8	6	0	1.405186	0.986019	-0.131012
9	7	0	2.083155	2.123431	-0.147859
10	6	0	1.921744	-0.332379	-0.342596
11	6	0	1.104398	-1.458809	-0.199847
12	6	0	-0.331528	-1.194460	-0.009607
13	6	0	-0.810785	0.109379	0.220732
14	6	0	-1.297158	-2.199993	-0.177163
15	6	0	-2.637796	-1.967357	-0.000228
16	6	0	-3.085651	-0.677911	0.344910
17	6	0	-2.162542	0.359592	0.435710
18	7	0	-4.438184	-0.421023	0.588696
19	6	0	3.328843	-0.327635	-0.508049
20	7	0	4.456924	-0.103568	-0.628606
21	1	0	-1.959163	5.048698	1.085859
22	1	0	2.239359	4.994071	0.160416
23	1	0	0.165666	6.252397	0.767459
24	1	0	-0.984604	-3.184606	-0.487724
25	1	0	-3.327107	-2.781466	-0.161139
26	1	0	-2.445321	1.365552	0.699463
27	6	0	-5.396898	-1.474445	0.685730
28	6	0	-6.055864	-1.668916	-0.755697
29	7	0	-6.572934	-0.426489	-1.189791
30	6	0	-5.680929	0.667175	-1.199497
31	6	0	-5.029226	0.836115	0.255101
32	1	0	-4.950744	-2.401686	1.026840
33	1	0	-6.188845	-1.180101	1.371545
34	1	0	-5.262429	-2.007611	-1.425321
35	1	0	-6.836268	-2.422304	-0.691302
36	1	0	-7.539434	-0.229543	-0.968983
37	1	0	-6.188028	1.591499	-1.462744
38	1	0	-4.849811	0.486016	-1.884400
39	1	0	-5.821443	1.080209	0.959413
40	1	0	-4.304844	1.640782	0.205509
41	7	0	1.549779	-2.721072	-0.277252
42	6	0	2.748635	-3.113173	-1.034513
43	6	0	3.874892	-3.572276	-0.119305
44	7	0	3.360490	-4.662979	0.684904
45	6	0	2.261202	-4.227798	1.517082
46	6	0	1.095546	-3.793266	0.631621

47	1	0	3.045005	-2.309934	-1.702239
48	1	0	2.447047	-3.962157	-1.651986
49	1	0	4.231423	-2.726906	0.487107
50	1	0	4.704003	-3.923572	-0.731417
51	1	0	4.087790	-5.110157	1.225045
52	1	0	1.923921	-5.053853	2.141862
53	1	0	2.527169	-3.386348	2.176504
54	1	0	0.751400	-4.642507	0.037886
55	1	0	0.289955	-3.426063	1.259064
56	29	0	4.094032	2.023454	-0.468335

Ligand	Description	Total Gibbs Free energy (M06-2X/Def2TZVPP)
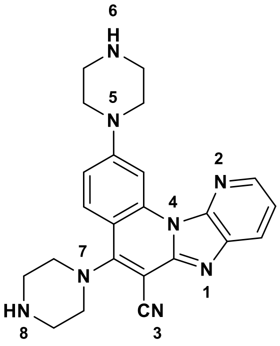 **5a**	Mg^{2+} bound to N1	-1527,9282352 a.u.

CARTESIAN COORDINATES

Center Number	Atomic Number	Atomic Type	Coordinates (Angstroms) X	Y	Z
1	6	0	-1.634855	4.344957	0.636304
2	7	0	-1.483453	3.027998	0.501817
3	6	0	-0.263284	2.632084	0.229001
4	6	0	0.852686	3.453936	0.072310
5	6	0	0.688982	4.821159	0.211779
6	6	0	-0.591562	5.262750	0.500744
7	7	0	0.196547	1.312218	0.040350
8	6	0	1.516532	1.383949	-0.220372
9	7	0	1.971491	2.663968	-0.206926
10	6	0	2.290716	0.205390	-0.425537
11	6	0	1.677136	-1.092811	-0.307403
12	6	0	0.261675	-1.105157	-0.171713
13	6	0	-0.498339	0.080900	0.039508
14	6	0	-0.510950	-2.277546	-0.378540
15	6	0	-1.860652	-2.307692	-0.249528
16	6	0	-2.599473	-1.138247	0.127148
17	6	0	-1.857053	0.075446	0.194496
18	7	0	-3.910464	-1.173400	0.363882
19	6	0	3.624545	0.546837	-0.532552
20	7	0	4.619394	1.158445	-0.610141
21	1	0	-2.634765	4.689304	0.862777
22	1	0	1.505894	5.523995	0.101873
23	1	0	-0.793712	6.316618	0.622244
24	1	0	-0.014136	-3.178552	-0.704757
25	1	0	-2.367697	-3.230661	-0.471858
26	1	0	-2.343758	1.016972	0.357634
27	6	0	-4.735027	-2.387688	0.314916
28	6	0	-5.794298	-2.247348	-0.771317
29	7	0	-6.598612	-1.078818	-0.473899
30	6	0	-5.805180	0.132912	-0.456374
31	6	0	-4.743298	0.005325	0.631266
32	1	0	-4.127180	-3.268689	0.159713
33	1	0	-5.229213	-2.481432	1.283779
34	1	0	-5.291446	-2.188409	-1.748851
35	1	0	-6.426020	-3.134154	-0.765176
36	1	0	-7.389912	-0.999866	-1.096951
37	1	0	-6.445898	0.982320	-0.224122
38	1	0	-5.300798	0.332810	-1.414497
39	1	0	-5.231992	-0.147854	1.595186
40	1	0	-4.145244	0.905525	0.688923
41	7	0	2.420929	-2.203289	-0.320655
42	6	0	3.726569	-2.297704	-0.984312
43	6	0	4.845266	-2.532698	0.019814
44	7	0	4.527743	-3.749273	0.733884
45	6	0	3.285691	-3.627561	1.466388
46	6	0	2.129911	-3.399010	0.495528

47	1	0	3.892762	-1.429327	-1.617459
48	1	0	3.674654	-3.164746	-1.646776
49	1	0	4.935053	-1.656623	0.684662
50	1	0	5.786551	-2.651574	-0.514785
51	1	0	5.287862	-4.057196	1.323628
52	1	0	3.092313	-4.549077	2.013421
53	1	0	3.297021	-2.797151	2.189890
54	1	0	2.016472	-4.263611	-0.160865
55	1	0	1.216786	-3.239487	1.057622
56	12	0	3.899746	2.981068	-0.451897

Ligand	Description	Total Gibbs Free energy (M06-2X/Def2TZVPP)
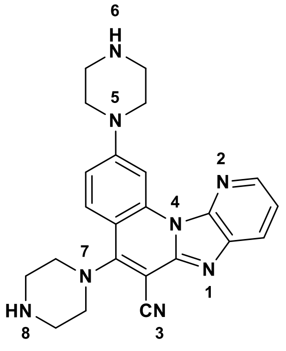 **5a**	Zn^{2+} bound to N1	-3107,2902382 a.u.

CARTESIAN COORDINATES

Center Number	Atomic Number	Atomic Type	Coordinates (Angstroms) X	Y	Z
1	6	0	1.291315	-4.366495	0.717481
2	7	0	1.331328	-3.043583	0.563374
3	6	0	0.180454	-2.478153	0.291316
4	6	0	-1.040392	-3.137091	0.154440
5	6	0	-1.080939	-4.510094	0.314033
6	6	0	0.124967	-5.126400	0.602479
7	7	0	-0.083543	-1.105453	0.086141
8	6	0	-1.400113	-0.981265	-0.166930
9	7	0	-2.023870	-2.190654	-0.130600
10	6	0	-1.997460	0.294440	-0.385162
11	6	0	-1.193429	1.489845	-0.278642
12	6	0	0.207170	1.293519	-0.151707
13	6	0	0.786989	0.009905	0.065339
14	6	0	1.142296	2.339211	-0.372546
15	6	0	2.482115	2.171336	-0.253447
16	6	0	3.044766	0.907392	0.125374
17	6	0	2.132127	-0.184377	0.208555
18	7	0	4.347589	0.751281	0.349901
19	6	0	-3.368698	0.168401	-0.495758
20	7	0	-4.449979	-0.284467	-0.573567
21	1	0	2.232670	-4.848379	0.944051
22	1	0	-1.995703	-5.081689	0.219824
23	1	0	0.174018	-6.196418	0.740193
24	1	0	0.780088	3.300774	-0.703189
25	1	0	3.118367	3.007152	-0.488306
26	1	0	2.477169	-1.185253	0.379021
27	6	0	5.339262	1.833714	0.292953
28	6	0	6.343058	1.553284	-0.818605
29	7	0	6.974563	0.277168	-0.548000
30	6	0	6.014726	-0.806951	-0.519762
31	6	0	5.006365	-0.538221	0.593293
32	1	0	4.862426	2.795693	0.162177
33	1	0	5.861961	1.840634	1.251111
34	1	0	5.816119	1.578943	-1.784884
35	1	0	7.096959	2.338810	-0.819898
36	1	0	7.733417	0.090316	-1.188169
37	1	0	6.530340	-1.742397	-0.307720
38	1	0	5.465261	-0.922839	-1.466927
39	1	0	5.532067	-0.465342	1.546876
40	1	0	4.285666	-1.343061	0.658269
41	7	0	-1.767657	2.695876	-0.293826
42	6	0	-3.050218	2.977408	-0.950035
43	6	0	-4.116028	3.375232	0.060241
44	7	0	-3.619592	4.534651	0.766124
45	6	0	-2.403672	4.237313	1.491767
46	6	0	-1.299561	3.840453	0.514905

47	1	0	-3.345014	2.141739	-1.581481
48	1	0	-2.876621	3.825215	-1.616712
49	1	0	-4.327459	2.523738	0.729865
50	1	0	-5.034001	3.627223	-0.468648
51	1	0	-4.322726	4.955592	1.356776
52	1	0	-2.075231	5.123222	2.033156
53	1	0	-2.530242	3.420165	2.219330
54	1	0	-1.066629	4.676337	-0.146988
55	1	0	-0.415117	3.551256	1.071177
56	30	0	-3.927008	-2.154320	-0.350441

Ligand	Description	Total Gibbs Free energy (M06-2X/Def2TZVPP)
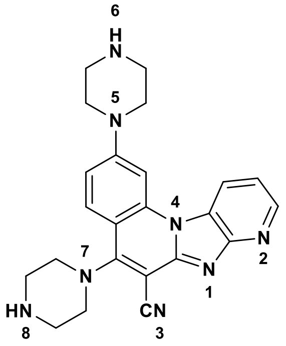<br>**5b**	H^+ bound to N1	-1328.845138 a.u.

CARTESIAN COORDINATES

Center Number	Atomic Number	Atomic Type	Coordinates (Angstroms) X	Y	Z
1	7	0	-0.231627	1.510938	-0.040003
2	6	0	0.325869	0.226064	-0.068899
3	6	0	-1.549525	1.680797	-0.325931
4	7	0	-1.832843	2.993958	-0.328110
5	6	0	-0.690619	3.707666	-0.008903
6	6	0	0.339177	2.779285	0.187501
7	6	0	-2.450541	0.625326	-0.491272
8	6	0	1.572963	3.265883	0.590421
9	7	0	-0.615907	5.008830	0.109366
10	6	0	1.676142	4.644493	0.719898
11	6	0	0.582049	5.468636	0.465431
12	1	0	2.611694	5.085241	1.030222
13	1	0	0.670797	6.542261	0.564362
14	6	0	-0.552207	-0.868794	-0.197035
15	6	0	-1.985375	-0.696767	-0.315578
16	7	0	-2.813157	-1.753687	-0.286056
17	6	0	1.700616	0.067056	-0.031730
18	6	0	0.064489	-2.125925	-0.378034
19	6	0	1.414827	-2.299853	-0.338704
20	6	0	2.293735	-1.200461	-0.142110
21	1	0	-0.551083	-2.980968	-0.609512
22	1	0	1.808982	-3.284159	-0.530188
23	7	0	3.645288	-1.366695	-0.132356
24	1	0	2.317102	0.937304	0.056058
25	1	0	2.419455	2.642903	0.826403
26	6	0	-3.809711	1.032474	-0.575308
27	7	0	-4.871787	1.468766	-0.629869
28	6	0	4.230647	-2.607053	0.394724
29	6	0	5.629444	-2.833128	-0.146591
30	1	0	4.279770	-2.535149	1.486882
31	1	0	3.605181	-3.453121	0.135379
32	7	0	6.440694	-1.671967	0.165317
33	1	0	6.049247	-3.714331	0.336192
34	1	0	5.568344	-3.026427	-1.227757
35	6	0	5.918230	-0.510158	-0.523987
36	1	0	7.411085	-1.828900	-0.069068
37	6	0	4.519031	-0.201728	-0.013800
38	1	0	6.554435	0.351724	-0.327592
39	1	0	5.865215	-0.656793	-1.613057
40	1	0	4.570431	0.126844	1.032078
41	1	0	4.115646	0.605101	-0.622226
42	6	0	-2.637745	-2.890083	0.631800
43	6	0	-3.853604	-2.981208	1.548887
44	1	0	-2.548838	-3.814459	0.056208
45	1	0	-1.743430	-2.741394	1.228283
46	7	0	-5.052741	-3.127413	0.749010

47	1	0	-3.733909	-3.853798	2.190205
48	1	0	-3.872293	-2.084854	2.188154
49	6	0	-5.271699	-1.954947	-0.082133
50	1	0	-5.857593	-3.322113	1.327919
51	6	0	-4.083632	-1.825573	-1.021437
52	1	0	-6.175522	-2.090172	-0.674303
53	1	0	-5.378750	-1.030699	0.501171
54	1	0	-4.027079	-2.731854	-1.630030
55	1	0	-4.177098	-0.979024	-1.692245
56	1	0	-2.752594	3.385721	-0.477883

Ligand	Description	Total Gibbs Free energy (M06-2X/Def2TZVPP)
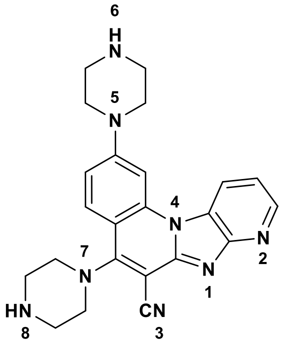<br>5b	Ca^{2+} bound to N1	-2005,5397315 a.u.

CARTESIAN COORDINATES

Center Number	Atomic Number	Atomic Type	Coordinates (Angstroms) X	Y	Z
1	6	0	-0.504717	4.708473	0.878277
2	6	0	-0.892300	3.381588	0.708545
3	6	0	0.098812	2.508257	0.289720
4	6	0	1.381119	3.036434	0.099184
5	7	0	1.775484	4.283525	0.268739
6	6	0	0.808257	5.121226	0.655134
7	7	0	0.244100	1.128850	0.024774
8	6	0	1.549273	0.920516	-0.283216
9	7	0	2.259930	2.047997	-0.261363
10	6	0	2.109371	-0.359977	-0.500128
11	6	0	1.283839	-1.510984	-0.338072
12	6	0	-0.129366	-1.250020	-0.214683
13	6	0	-0.661653	0.053003	-0.030255
14	6	0	-1.094398	-2.262370	-0.433669
15	6	0	-2.433806	-2.041465	-0.363958
16	6	0	-2.961831	-0.745429	-0.083185
17	6	0	-2.012872	0.297272	0.046064
18	6	0	3.497198	-0.193065	-0.586578
19	7	0	4.551578	0.293280	-0.630471
20	1	0	-1.232093	5.438067	1.201689
21	1	0	1.087816	6.155432	0.801407
22	7	0	1.791463	-2.744458	-0.318656
23	1	0	-0.761133	-3.246200	-0.726937
24	1	0	-3.094778	-2.859921	-0.593896
25	1	0	-2.341454	1.306066	0.199284
26	1	0	-1.908479	3.089941	0.921535
27	7	0	-4.285429	-0.521159	-0.006788
28	6	0	-5.243783	-1.585365	0.333186
29	6	0	-6.567515	-1.390450	-0.383803
30	7	0	-7.078326	-0.076532	-0.048067
31	6	0	-6.186140	0.947200	-0.545710
32	6	0	-4.836881	0.827064	0.151158
33	1	0	-4.831082	-2.556617	0.092529
34	1	0	-5.417790	-1.542379	1.412677
35	1	0	-6.415737	-1.524758	-1.464979
36	1	0	-7.262129	-2.154267	-0.038310
37	1	0	-8.020300	0.055418	-0.389242
38	1	0	-6.596631	1.932100	-0.327897
39	1	0	-6.022672	0.876157	-1.631580
40	1	0	-4.948793	1.052350	1.216847
41	1	0	-4.164456	1.548719	-0.303251
42	6	0	1.274851	-3.837164	0.529343
43	6	0	3.082386	-3.086773	-0.928254
44	6	0	4.111114	-3.464190	0.127039
45	7	0	3.568765	-4.577500	0.875263
46	6	0	2.342791	-4.215188	1.552843

47	1	0	4.246089	-4.979820	1.507502
48	1	0	1.038490	-4.696613	-0.100271
49	1	0	0.384570	-3.504401	1.050157
50	1	0	3.414759	-2.281882	-1.578268
51	1	0	2.903002	-3.959347	-1.559876
52	1	0	4.327580	-2.586591	0.759113
53	1	0	5.034469	-3.764478	-0.365923
54	1	0	1.977272	-5.065241	2.127085
55	1	0	2.471883	-3.366765	2.243600
56	20	0	4.412808	2.632273	-0.392546

Ligand	Description	Total Gibbs Free energy (M06-2X/Def2TZVPP)
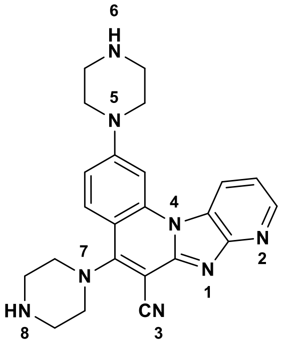 5b	Cu^{2+} bound to N1	-2968,3764659 a.u.

CARTESIAN COORDINATES

Center Number	Atomic Number	Atomic Type	Coordinates (Angstroms) X	Y	Z
1	6	0	0.331451	-4.742486	1.205246
2	6	0	0.774541	-3.434883	1.091500
3	6	0	-0.128259	-2.533030	0.546171
4	6	0	-1.410361	-2.989702	0.203439
5	7	0	-1.839810	-4.229965	0.313378
6	6	0	-0.960517	-5.095807	0.800851
7	7	0	-0.153730	-1.153662	0.278510
8	6	0	-1.421291	-0.866501	-0.170745
9	7	0	-2.186429	-1.934448	-0.234941
10	6	0	-1.831496	0.482434	-0.418763
11	6	0	-0.960796	1.544183	-0.174151
12	6	0	0.446425	1.168125	0.056675
13	6	0	0.827946	-0.172075	0.278936
14	6	0	1.474451	2.108356	-0.096179
15	6	0	2.800017	1.784004	0.056038
16	6	0	3.163245	0.456236	0.333043
17	6	0	2.168303	-0.513815	0.428440
18	6	0	-3.220637	0.549089	-0.698640
19	7	0	-4.339741	0.358038	-0.910593
20	1	0	0.979145	-5.497938	1.624716
21	1	0	-1.294980	-6.120844	0.893634
22	7	0	-1.306266	2.838583	-0.213730
23	1	0	1.223135	3.118646	-0.379744
24	1	0	3.541621	2.554792	-0.085166
25	1	0	2.424552	-1.537914	0.629387
26	1	0	1.755747	-3.170689	1.453492
27	7	0	4.506383	0.095104	0.503467
28	6	0	5.523745	1.076919	0.701811
29	6	0	6.190233	1.399926	-0.714127
30	7	0	6.625878	0.187468	-1.295016
31	6	0	5.665282	-0.835472	-1.431813
32	6	0	5.012671	-1.142012	0.001947
33	1	0	5.129603	1.982579	1.149548
34	1	0	6.303511	0.665737	1.340387
35	1	0	5.419508	1.863170	-1.333218
36	1	0	7.016772	2.089902	-0.567762
37	1	0	7.580650	-0.093894	-1.117851
38	1	0	6.108599	-1.750934	-1.814268
39	1	0	4.844201	-0.515547	-2.076648
40	1	0	5.792141	-1.529919	0.654442
41	1	0	4.234156	-1.881743	-0.144872
42	6	0	-0.824111	3.823253	0.776767
43	6	0	-2.468411	3.339897	-0.962190
44	6	0	-3.594297	3.771505	-0.032844
45	7	0	-3.054174	4.777116	0.860766
46	6	0	-1.986680	4.240495	1.674839

47	1	0	-3.773885	5.211280	1.421286
48	1	0	-0.436531	4.696061	0.247885
49	1	0	-0.043042	3.381304	1.386960
50	1	0	-2.781867	2.604342	-1.696890
51	1	0	-2.118692	4.220715	-1.504543
52	1	0	-3.992756	2.895818	0.500752
53	1	0	-4.398942	4.199616	-0.628348
54	1	0	-1.629272	5.005793	2.362635
55	1	0	-2.295954	3.364840	2.267768
56	29	0	-4.164860	-1.821885	-0.704620

Ligand	Description	Total Gibbs Free energy (M06-2X/Def2TZVPP)
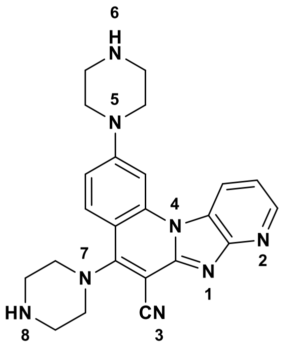 5b	Mg^{2+} bound to N1	-1527,9060811 a.u.

CARTESIAN COORDINATES

Center Number	Atomic Number	Atomic Type	Coordinates (Angstroms) X	Y	Z
1	6	0	-0.971200	4.989369	0.833905
2	6	0	-0.998070	3.604593	0.844091
3	6	0	0.113102	2.976202	0.302906
4	6	0	1.201191	3.748441	-0.149158
5	7	0	1.220941	5.065247	-0.160329
6	6	0	0.137232	5.666559	0.314201
7	7	0	0.530292	1.645139	0.139977
8	6	0	1.874480	1.734914	-0.355681
9	7	0	2.260044	2.941370	-0.543473
10	6	0	2.650609	0.533508	-0.481099
11	6	0	2.101835	-0.701158	-0.217175
12	6	0	0.635994	-0.731598	-0.010215
13	6	0	-0.113787	0.471813	0.192612
14	6	0	-0.063571	-1.913963	-0.109636
15	6	0	-1.464773	-1.977185	0.020746
16	6	0	-2.182736	-0.762337	0.282104
17	6	0	-1.527125	0.427929	0.346185
18	6	0	4.037942	0.741401	-0.736802
19	7	0	5.155969	0.906957	-0.931926
20	1	0	-1.795013	5.553486	1.245361
21	1	0	0.149212	6.749238	0.304026
22	7	0	2.761453	-1.888385	-0.224274
23	1	0	0.482933	-2.829820	-0.282951
24	1	0	-1.876620	-2.934908	0.359025
25	1	0	-2.074469	1.351192	0.439224
26	1	0	-1.829354	3.088877	1.301384
27	7	0	-3.619304	-0.881120	0.171618
28	6	0	-4.351668	-1.681628	1.184832
29	6	0	-5.565106	-2.259617	0.461595
30	7	0	-6.346578	-1.216429	-0.094605
31	6	0	-5.650393	-0.345043	-0.976576
32	6	0	-4.433279	0.263672	-0.281972
33	1	0	-3.710174	-2.473101	1.569505
34	1	0	-4.662710	-1.048341	2.016895
35	1	0	-5.150454	-2.940216	-0.363685
36	1	0	-6.160860	-2.905075	1.102754
37	1	0	-7.292066	-1.454922	-0.356063
38	1	0	-6.312373	0.418231	-1.379028
39	1	0	-5.250779	-0.918516	-1.875130
40	1	0	-4.740431	0.889346	0.557545
41	1	0	-3.857275	0.863226	-0.986830
42	6	0	2.617240	-2.846625	0.885348
43	6	0	3.922640	-2.179559	-1.082290
44	6	0	5.204707	-2.364515	-0.283177
45	7	0	4.961646	-3.386507	0.723410
46	6	0	3.932678	-2.962649	1.648007

47	1	0	5.813452	-3.634131	1.207415
48	1	0	2.362834	-3.831317	0.481445
49	1	0	1.831821	-2.510050	1.559398
50	1	0	4.022238	-1.407564	-1.838198
51	1	0	3.696622	-3.125005	-1.582845
52	1	0	5.512093	-1.406330	0.151001
53	1	0	5.989615	-2.698273	-0.959944
54	1	0	3.814590	-3.707591	2.434411
55	1	0	4.149392	-1.992199	2.121219
56	12	0	-3.185927	-2.166273	-1.382102

Ligand	Description	Total Gibbs Free energy (M06-2X/Def2TZVPP)
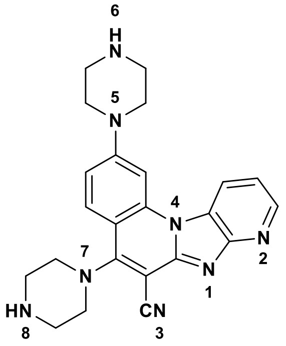 **5b**	Zn^{2+} bound to N1	-3107,2925439 a.u.

CARTESIAN COORDINATES

Center Number	Atomic Number	Atomic Type	Coordinates (Angstroms) X	Y	Z
1	6	0	-0.644157	-4.627656	-1.078783
2	6	0	-0.999375	-3.297087	-0.907174
3	6	0	-0.006174	-2.459260	-0.417885
4	6	0	1.251914	-3.018920	-0.187426
5	7	0	1.611957	-4.267915	-0.352119
6	6	0	0.646963	-5.075084	-0.788803
7	7	0	0.133359	-1.082958	-0.127917
8	6	0	1.420610	-0.868313	0.223465
9	7	0	2.121797	-2.018178	0.211547
10	6	0	1.939724	0.432184	0.476418
11	6	0	1.092441	1.575481	0.267087
12	6	0	-0.299865	1.292726	0.098703
13	6	0	-0.801151	-0.025729	-0.077877
14	6	0	-1.290819	2.289499	0.289139
15	6	0	-2.621565	2.039138	0.206810
16	6	0	-3.119880	0.724164	-0.060162
17	6	0	-2.141236	-0.303734	-0.149597
18	6	0	3.309922	0.339640	0.669175
19	7	0	4.384253	-0.105851	0.808070
20	1	0	-1.371685	-5.331351	-1.455368
21	1	0	0.911297	-6.114540	-0.928207
22	7	0	1.592716	2.809598	0.249409
23	1	0	-0.982402	3.283288	0.576097
24	1	0	-3.302705	2.844768	0.422294
25	1	0	-2.446575	-1.324159	-0.260577
26	1	0	-1.989428	-2.967296	-1.177151
27	7	0	-4.427390	0.475718	-0.169004
28	6	0	-5.446106	1.517237	-0.371838
29	6	0	-6.573555	1.372722	0.638060
30	7	0	-7.139999	0.047138	0.489487
31	6	0	-6.159232	-0.975805	0.778824
32	6	0	-5.011884	-0.868478	-0.222015
33	1	0	-5.002809	2.503197	-0.328581
34	1	0	-5.854053	1.372001	-1.375389
35	1	0	-6.178609	1.561565	1.647631
36	1	0	-7.334129	2.121238	0.422464
37	1	0	-7.976135	-0.069275	1.044643
38	1	0	-6.615266	-1.959361	0.675755
39	1	0	-5.741104	-0.895174	1.793819
40	1	0	-5.387476	-1.046958	-1.232542
41	1	0	-4.268011	-1.617853	0.021873
42	6	0	1.088410	3.890166	-0.624568
43	6	0	2.853912	3.180664	0.903594
44	6	0	3.915738	3.559504	-0.118236
45	7	0	3.383983	4.656830	-0.894323
46	6	0	2.188079	4.274232	-1.612308

47	1	0	4.077263	5.071492	-1.500854
48	1	0	0.817980	4.749478	-0.009025
49	1	0	0.221994	3.538498	-1.172676
50	1	0	3.172096	2.395366	1.585945
51	1	0	2.636612	4.058578	1.515626
52	1	0	4.165983	2.678480	-0.734038
53	1	0	4.817350	3.876066	0.403845
54	1	0	1.831910	5.115624	-2.204784
55	1	0	2.351779	3.423252	-2.292244
56	30	0	4.008837	-2.022378	0.514926

Ligand	Description	Total Gibbs Free energy (M06-2X/Def2TZVPP)
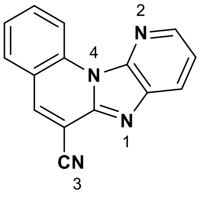 6a	H^+ bound to N1	-795.613072 a.u.

CARTESIAN COORDINATES

Center Number	Atomic Number	Atomic Type	Coordinates (Angstroms) X	Y	Z
1	7	0	0.113069	-0.143630	0.000003
2	6	0	-1.123045	-0.802682	0.000117
3	6	0	0.203812	1.203864	0.000021
4	7	0	1.491787	1.576045	0.000074
5	6	0	2.285742	0.444374	0.000072
6	6	0	1.428587	-0.655256	0.000033
7	6	0	-0.944531	2.032380	0.000049
8	7	0	1.793089	-1.916473	-0.000025
9	6	0	3.656510	0.235982	-0.000004
10	6	0	3.105990	-2.118576	-0.000051
11	6	0	4.054865	-1.086780	-0.000055
12	1	0	3.428654	-3.150975	-0.000121
13	1	0	5.105202	-1.338217	-0.000074
14	1	0	4.366645	1.050669	-0.000001
15	6	0	-2.280168	0.007912	0.000085
16	6	0	-2.164002	1.424216	0.000021
17	1	0	-3.062436	2.027600	-0.000254
18	6	0	-1.220465	-2.192313	0.000002
19	6	0	-3.541591	-0.615212	0.000030
20	6	0	-3.638534	-1.983440	-0.000134
21	6	0	-2.476331	-2.763972	0.000055
22	1	0	-4.427966	0.004968	-0.000043
23	1	0	-4.608038	-2.460224	-0.000088
24	1	0	-2.558763	-3.841957	-0.000294
25	1	0	-0.330032	-2.798933	0.000173
26	6	0	-0.728507	3.443825	-0.000119
27	7	0	-0.457403	4.558342	-0.000037
28	1	0	1.792948	2.541151	-0.000129

Ligand	Description	Total Gibbs Free energy (M06-2X/Def2TZVPP)
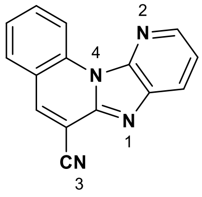 **6a**	Ca^{2+} bound to N1	-1472.371828 a.u.

CARTESIAN COORDINATES

Center Number	Atomic Number	Atomic Type	Coordinates (Angstroms) X	Y	Z
1	7	0	-0.564840	-0.368668	0.000436
2	6	0	-1.931396	-0.056605	0.000361
3	6	0	0.405058	0.575471	-0.000213
4	7	0	1.650506	0.068739	-0.000330
5	6	0	1.461312	-1.316314	-0.000251
6	6	0	0.096763	-1.610792	0.000291
7	6	0	0.033921	1.955661	-0.000515
8	7	0	-0.452243	-2.803179	0.000345
9	6	0	2.351680	-2.384551	-0.000580
10	6	0	0.406310	-3.815455	-0.000381
11	6	0	1.799609	-3.650225	-0.000964
12	1	0	-0.022690	-4.808557	-0.000467
13	1	0	2.433366	-4.524989	-0.001514
14	1	0	3.427883	-2.256556	-0.000915
15	6	0	-2.288123	1.318953	-0.000352
16	6	0	-1.288725	2.313944	-0.000802
17	1	0	-1.574432	3.358272	-0.001282
18	6	0	-2.910599	-1.041309	0.000941
19	6	0	-3.654460	1.670166	-0.000507
20	6	0	-4.617633	0.695228	0.000029
21	6	0	-4.238780	-0.653294	0.000753
22	1	0	-3.923969	2.718097	-0.001019
23	1	0	-5.664692	0.961602	-0.000086
24	1	0	-5.003008	-1.418737	0.001197
25	1	0	-2.635279	-2.083393	0.001499
26	6	0	1.170040	2.791823	-0.000559
27	7	0	2.249087	3.204119	-0.000430
28	20	0	3.600769	1.329549	0.000947

Ligand	Description	Total Gibbs Free energy (M06-2X/Def2TZVPP)
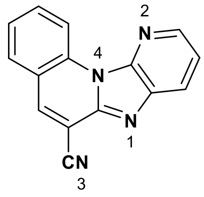<br>**6a**	Cu^{2+} bound to N1	-2435.124637 a.u.

CARTESIAN COORDINATES

Center Number	Atomic Number	Atomic Type	Coordinates (Angstroms) X	Y	Z
1	7	0	0.771211	0.413447	0.000010
2	6	0	2.099695	-0.020976	0.000007
3	6	0	-0.286264	-0.440871	0.000005
4	7	0	-1.492916	0.212691	0.000020
5	6	0	-1.201767	1.513225	0.000013
6	6	0	0.225332	1.702340	0.000017
7	6	0	-0.083749	-1.822549	0.000002
8	7	0	0.859201	2.835398	-0.000001
9	6	0	-2.019881	2.667313	-0.000011
10	6	0	0.060955	3.912441	-0.000017
11	6	0	-1.363035	3.863531	-0.000030
12	1	0	0.563109	4.871747	-0.000026
13	1	0	-1.907386	4.797375	-0.000049
14	1	0	-3.099328	2.597852	-0.000005
15	6	0	2.306787	-1.437930	0.000011
16	6	0	1.217966	-2.312743	0.000003
17	1	0	1.389366	-3.382403	-0.000002
18	6	0	3.173077	0.851859	-0.000002
19	6	0	3.636503	-1.934696	0.000002
20	6	0	4.692331	-1.067734	-0.000006
21	6	0	4.454446	0.318219	0.000004
22	1	0	3.791245	-3.005700	0.000001
23	1	0	5.707463	-1.438189	-0.000019
24	1	0	5.296983	0.997527	0.000013
25	1	0	3.020042	1.918973	-0.000021
26	6	0	-1.282448	-2.601531	-0.000002
27	7	0	-2.353864	-3.023733	-0.000098
28	29	0	-3.207814	-1.019351	0.000021

Ligand	Description	Total Gibbs Free energy (M06-2X/Def2TZVPP)
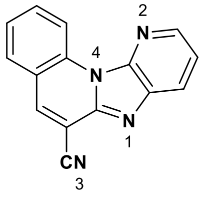 **6a**	Mg^{2+} bound to N1	-994.772366 a.u.

CARTESIAN COORDINATES

Center Number	Atomic Number	Atomic Type	Coordinates (Angstroms) X	Y	Z
1	7	0	-0.244823	0.347230	-0.000004
2	6	0	-1.646588	0.371820	-0.000009
3	6	0	0.465158	-0.799726	-0.000022
4	7	0	1.800196	-0.593692	-0.000077
5	6	0	1.952695	0.797628	-0.000021
6	6	0	0.696063	1.400223	-0.000009
7	6	0	-0.224913	-2.054089	-0.000006
8	7	0	0.442430	2.686851	0.000028
9	6	0	3.072128	1.620365	0.000001
10	6	0	1.515413	3.468942	0.000045
11	6	0	2.831025	2.980069	0.000031
12	1	0	1.331829	4.535033	0.000079
13	1	0	3.653020	3.681049	0.000043
14	1	0	4.084844	1.236711	-0.000001
15	6	0	-2.324997	-0.880453	0.000026
16	6	0	-1.598727	-2.084007	0.000030
17	1	0	-2.126106	-3.029744	0.000047
18	6	0	-2.360028	1.561924	-0.000041
19	6	0	-3.737554	-0.892942	0.000031
20	6	0	-4.436742	0.284748	0.000002
21	6	0	-3.743289	1.502888	-0.000040
22	1	0	-4.250765	-1.845560	0.000067
23	1	0	-5.517228	0.279335	0.000012
24	1	0	-4.301326	2.429797	-0.000032
25	1	0	-1.843749	2.508130	-0.000075
26	6	0	0.699984	-3.115438	-0.000020
27	7	0	1.705676	-3.690202	0.000070
28	12	0	3.007280	-2.168148	-0.000021

Ligand	Description	Total Gibbs Free energy (M06-2X/Def2TZVPP)
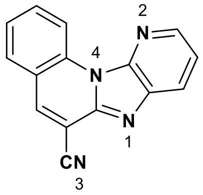<br>**6a**	Zn^{2+} bound to N1	-2574.017591 a.u.

CARTESIAN COORDINATES

Center Number	Atomic Number	Atomic Type	Coordinates (Angstroms) X	Y	Z
1	7	0	0.724689	0.389974	0.000006
2	6	0	2.068787	-0.009092	0.000000
3	6	0	-0.295300	-0.490546	0.000020
4	7	0	-1.499627	0.127769	0.000085
5	6	0	-1.233735	1.498223	0.000030
6	6	0	0.147260	1.682317	0.000012
7	6	0	-0.021381	-1.896747	0.000016
8	7	0	0.780034	2.829576	-0.000019
9	6	0	-2.051742	2.619597	0.000014
10	6	0	-0.002532	3.902588	-0.000032
11	6	0	-1.405071	3.839743	-0.000017
12	1	0	0.498515	4.861353	-0.000062
13	1	0	-1.973043	4.758732	-0.000027
14	1	0	-3.132534	2.556716	0.000026
15	6	0	2.336989	-1.408997	-0.000025
16	6	0	1.283205	-2.336094	-0.000035
17	1	0	1.501216	-3.396886	-0.000037
18	6	0	3.107933	0.909769	0.000028
19	6	0	3.680477	-1.848138	-0.000031
20	6	0	4.702115	-0.936567	-0.000010
21	6	0	4.408652	0.434553	0.000031
22	1	0	3.881939	-2.911295	-0.000061
23	1	0	5.730671	-1.267599	-0.000035
24	1	0	5.220840	1.149415	0.000008
25	1	0	2.902776	1.967854	0.000052
26	6	0	-1.217197	-2.641780	0.000057
27	7	0	-2.351556	-2.892655	0.000015
28	30	0	-3.041864	-1.027130	-0.000027

Ligand	Description	Total Gibbs Free energy (M06-2X/Def2TZVPP)
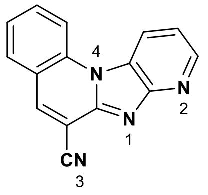 **6b**	H^+ bound to N1	-795.607971 a.u.

CARTESIAN COORDINATES

Center Number	Atomic Number	Atomic Type	Coordinates (Angstroms) X	Y	Z
1	7	0	-0.096833	-0.188944	0.000048
2	6	0	1.167345	-0.796121	0.000079
3	6	0	-0.220396	1.162446	0.000097
4	7	0	-1.510375	1.502055	0.000281
5	6	0	-2.276649	0.351547	0.000178
6	6	0	-1.396869	-0.735752	0.000010
7	6	0	0.902293	2.027619	-0.000041
8	6	0	-1.952451	-2.012032	-0.000400
9	7	0	-3.588087	0.315025	0.000185
10	6	0	-3.333350	-2.073081	-0.000417
11	6	0	-4.106514	-0.905465	-0.000031
12	1	0	-3.826847	-3.033567	-0.000766
13	1	0	-5.186801	-0.967125	-0.000106
14	6	0	2.297587	0.051084	-0.000254
15	6	0	2.139528	1.463682	-0.000323
16	6	0	0.645539	3.432610	0.000021
17	7	0	0.356423	4.542366	0.000088
18	1	0	3.018712	2.094485	-0.000514
19	6	0	1.332567	-2.178461	0.000529
20	6	0	3.581273	-0.524366	-0.000361
21	6	0	3.736646	-1.886939	-0.000075
22	6	0	2.605710	-2.709274	0.000436
23	1	0	4.441660	0.131489	-0.000623
24	1	0	4.724191	-2.324779	-0.000179
25	1	0	2.724515	-3.783842	0.000826
26	1	0	0.488389	-2.842383	0.001104
27	1	0	-1.380950	-2.923462	-0.000784
28	1	0	-1.864321	2.450700	0.000148

Ligand	Description	Total Gibbs Free energy (M06-2X/Def2TZVPP)
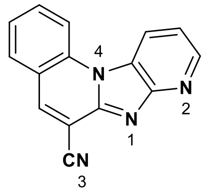 **6b**	Ca^{2+} bound to N1	-1472.385505 a.u.

CARTESIAN COORDINATES

Center Number	Atomic Number	Atomic Type	X	Y	Z
1	7	0	0.607620	-0.420378	-0.000391
2	6	0	1.998185	-0.243283	-0.000167
3	6	0	-0.221733	0.654731	-0.000335
4	7	0	-1.505298	0.324374	-0.000154
5	6	0	-1.526283	-1.047092	0.000001
6	6	0	-0.226083	-1.561376	-0.000225
7	6	0	0.268972	1.992471	-0.000174
8	6	0	-0.082748	-2.945440	-0.000189
9	7	0	-2.662912	-1.721348	0.000308
10	6	0	-1.262967	-3.672693	0.000090
11	6	0	-2.515981	-3.044512	0.000345
12	1	0	-1.223566	-4.752048	0.000113
13	1	0	-3.420348	-3.637793	0.000593
14	6	0	2.495169	1.089829	0.000062
15	6	0	1.616229	2.204814	0.000104
16	6	0	-0.849357	2.873101	0.000059
17	7	0	-1.945315	3.230988	-0.000084
18	1	0	2.027193	3.206222	0.000336
19	6	0	2.877130	-1.316840	-0.000213
20	6	0	3.890379	1.290741	0.000295
21	6	0	4.751660	0.223206	0.000298
22	6	0	4.238846	-1.078203	0.000039
23	1	0	4.268520	2.304618	0.000468
24	1	0	5.820128	0.382860	0.000481
25	1	0	4.918095	-1.919795	0.000020
26	1	0	2.514957	-2.330569	-0.000456
27	1	0	0.864273	-3.460300	-0.000447
28	20	0	-3.496822	1.389731	0.000060

Ligand	Description	Total Gibbs Free energy (M06-2X/Def2TZVPP)
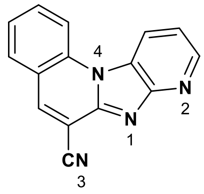 **6b**	Cu^{2+} bound to N1	-2435.118824 a.u.

CARTESIAN COORDINATES

Center Number	Atomic Number	Atomic Type	Coordinates (Angstroms) X	Y	Z
1	7	0	0.792821	0.468155	0.000076
2	6	0	2.144530	0.097467	0.000148
3	6	0	-0.203902	-0.465020	0.000049
4	7	0	-1.445441	0.091506	0.000165
5	6	0	-1.257266	1.404379	0.000093
6	6	0	0.153974	1.712978	-0.000061
7	6	0	0.075151	-1.834349	-0.000207
8	6	0	0.550655	3.033991	-0.000559
9	7	0	-2.252417	2.307812	0.000128
10	6	0	-0.487030	3.958832	-0.000653
11	6	0	-1.870390	3.550881	-0.000196
12	1	0	-0.262725	5.017159	-0.001129
13	1	0	-2.642249	4.311656	-0.000116
14	6	0	2.431541	-1.303730	-0.000265
15	6	0	1.397136	-2.247712	-0.000416
16	6	0	-1.092099	-2.661130	-0.000364
17	7	0	-2.156998	-3.096368	-0.000666
18	1	0	1.635825	-3.304301	-0.000755
19	6	0	3.181274	1.013379	0.000746
20	6	0	3.783586	-1.729388	-0.000363
21	6	0	4.798879	-0.813258	0.000034
22	6	0	4.490445	0.554662	0.000671
23	1	0	3.991143	-2.791513	-0.000693
24	1	0	5.830718	-1.133876	-0.000040
25	1	0	5.293827	1.279586	0.001156
26	1	0	3.003032	2.073690	0.001443
27	1	0	1.571555	3.376135	-0.000972
28	29	0	-3.157441	-1.133040	0.000387

Ligand	Description	Total Gibbs Free energy (M06-2X/Def2TZVPP)
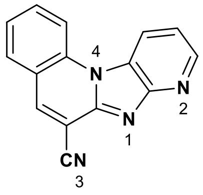 6b	Mg^{2+} bound to N1	-994.779757 a.u.

CARTESIAN COORDINATES

Center Number	Atomic Number	Atomic Type	Coordinates (Angstroms) X	Y	Z
1	7	0	0.294599	-0.390168	-0.000436
2	6	0	1.696153	-0.434125	-0.000229
3	6	0	-0.362100	0.795291	-0.000576
4	7	0	-1.688509	0.650741	-0.000288
5	6	0	-1.914642	-0.712721	-0.000257
6	6	0	-0.697879	-1.398547	-0.000188
7	6	0	0.343581	2.037434	-0.000373
8	6	0	-0.740093	-2.790512	0.000136
9	7	0	-3.124262	-1.230219	0.000021
10	6	0	-2.004181	-3.353908	0.000340
11	6	0	-3.159312	-2.558691	0.000292
12	1	0	-2.109697	-4.428930	0.000581
13	1	0	-4.138040	-3.019635	0.000487
14	6	0	2.401706	0.803591	0.000147
15	6	0	1.712082	2.035828	-0.000023
16	6	0	-0.604790	3.088301	0.000009
17	7	0	-1.636288	3.610439	-0.000029
18	1	0	2.270822	2.963141	0.000398
19	6	0	2.396632	-1.631044	-0.000468
20	6	0	3.813267	0.784333	0.000566
21	6	0	4.494486	-0.404574	0.000465
22	6	0	3.779703	-1.608022	-0.000115
23	1	0	4.344702	1.726971	0.000863
24	1	0	5.574799	-0.417414	0.000756
25	1	0	4.316957	-2.546837	-0.000317
26	1	0	1.884696	-2.576967	-0.001080
27	1	0	0.128478	-3.427754	0.000317
28	12	0	-3.009931	2.110506	0.000396

Ligand	Description	Total Gibbs Free energy (M06-2X/Def2TZVPP)
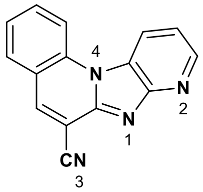 **6b**	Zn^{2+} bound to N1	-2574.021385 a.u.

CARTESIAN COORDINATES

Center Number	Atomic Number	Atomic Type	Coordinates (Angstroms) X	Y	Z
1	7	0	-0.756252	0.451830	0.000133
2	6	0	-2.123120	0.135713	0.000022
3	6	0	0.182339	-0.524020	0.000180
4	7	0	1.423392	-0.022472	0.000092
5	6	0	1.294074	1.354917	0.000049
6	6	0	-0.062150	1.687167	0.000076
7	6	0	-0.180836	-1.907189	0.000201
8	6	0	-0.392697	3.040726	0.000145
9	7	0	2.318595	2.174511	-0.000017
10	6	0	0.673479	3.921960	0.000077
11	6	0	1.999406	3.463671	-0.000015
12	1	0	0.487095	4.986005	0.000111
13	1	0	2.820983	4.167648	-0.000102
14	6	0	-2.488580	-1.241889	0.000104
15	6	0	-1.507430	-2.253081	0.000187
16	6	0	0.989011	-2.702816	0.000089
17	7	0	2.116699	-2.970246	-0.000013
18	1	0	-1.808296	-3.293177	0.000214
19	6	0	-3.107288	1.112389	-0.000180
20	6	0	-3.858417	-1.586710	0.000032
21	6	0	-4.821235	-0.612513	-0.000135
22	6	0	-4.438136	0.734175	-0.000262
23	1	0	-4.129647	-2.634307	0.000101
24	1	0	-5.868902	-0.876565	-0.000188
25	1	0	-5.198213	1.503915	-0.000436
26	1	0	-2.857794	2.158158	-0.000312
27	1	0	-1.399398	3.423413	0.000267
28	30	0	2.976220	-1.153515	-0.000148

Ligand	Description	Total Gibbs Free energy (M06-2X/Def2TZVPP)
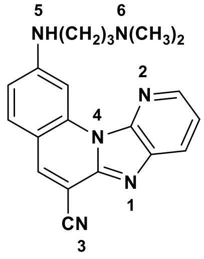 1a	2:1 complex with H^+ bound to N1	-2205.049333 a.u.

CARTESIAN COORDINATES

Center Number	Atomic Number	Atomic Type	Coordinates (Angstroms) X	Y	Z
1	7	0	-3.477569	0.723562	-0.108269
2	6	0	-4.508759	-0.143360	-0.477506
3	6	0	-2.156768	0.521153	-0.444432
4	7	0	-1.355258	1.465472	0.009108
5	6	0	-2.171087	2.337491	0.692962
6	6	0	-3.504805	1.903826	0.634845
7	6	0	-1.804707	-0.636654	-1.203543
8	7	0	-4.549312	2.499707	1.173669
9	6	0	-1.900400	3.507336	1.391788
10	6	0	-4.270014	3.618624	1.835698
11	6	0	-2.981918	4.147764	1.968525
12	1	0	-5.111816	4.125942	2.288502
13	1	0	-2.845175	5.061536	2.528207
14	1	0	-0.894376	3.894257	1.483606
15	6	0	-4.139226	-1.282817	-1.235676
16	6	0	-2.787788	-1.505108	-1.578811
17	1	0	-2.531281	-2.388468	-2.149134
18	6	0	-5.820159	0.092576	-0.124914
19	6	0	-5.161328	-2.170998	-1.619153
20	6	0	-6.466221	-1.954775	-1.280029
21	6	0	-6.820467	-0.810786	-0.519780
22	1	0	-4.893443	-3.045521	-2.198127
23	1	0	-7.227781	-2.654168	-1.589460
24	7	0	-8.103140	-0.582403	-0.170660
25	1	0	-6.073246	0.966632	0.454421
26	1	0	-8.303843	0.253434	0.352706
27	6	0	-9.225774	-1.425165	-0.533471
28	6	0	-10.513610	-0.858107	0.038533
29	1	0	-9.301765	-1.493782	-1.623360
30	1	0	-9.064548	-2.438442	-0.151752
31	6	0	-11.701524	-1.752524	-0.288509
32	1	0	-10.398692	-0.753411	1.119470
33	1	0	-10.699087	0.135956	-0.375637
34	7	0	-12.977830	-1.130553	0.007154
35	1	0	-11.686439	-1.979824	-1.357474
36	1	0	-11.593347	-2.715164	0.244832
37	6	0	-14.066370	-1.942614	-0.505872
38	6	0	-13.153292	-0.890792	1.427740
39	1	0	-15.015395	-1.440836	-0.323192
40	1	0	-13.952004	-2.079157	-1.580741
41	1	0	-14.105234	-2.936556	-0.031741
42	1	0	-14.139287	-0.463076	1.601893
43	1	0	-13.071022	-1.818255	2.018662
44	1	0	-12.414729	-0.180191	1.794460
45	6	0	-0.428811	-0.843947	-1.507374
46	7	0	0.692152	-0.980642	-1.721348
47	1	0	0.303944	1.398319	0.080075
48	7	0	1.374369	1.406632	0.056528
49	6	0	2.171470	0.447379	0.527996

50	7	0	3.462303	0.677082	0.155980
51	6	0	3.466251	1.845233	-0.624811
52	6	0	2.140052	2.278513	-0.683113
53	6	0	1.835440	3.415090	-1.414556
54	7	0	4.495936	2.428690	-1.197036
55	6	0	4.515132	-0.170287	0.518330
56	6	0	1.824653	-0.686657	1.298169
57	6	0	2.833083	-1.539284	1.665279
58	6	0	4.168883	-1.304551	1.298605
59	6	0	2.907096	4.037833	-2.029060
60	6	0	4.200172	3.522600	-1.894343
61	1	0	0.823417	3.783919	-1.502818
62	1	0	2.755208	4.928510	-2.620721
63	1	0	5.032540	4.018232	-2.375798
64	6	0	0.454951	-0.909989	1.621990
65	7	0	-0.656045	-1.070749	1.862581
66	1	0	2.594780	-2.419416	2.248216
67	6	0	5.210879	-2.177884	1.676105
68	6	0	5.814644	0.079018	0.138610
69	6	0	6.503168	-1.949549	1.310628
70	6	0	6.832671	-0.809364	0.527555
71	7	0	8.102815	-0.574723	0.155596
72	1	0	6.048825	0.946715	-0.457190
73	1	0	4.963198	-3.047571	2.270701
74	1	0	7.279260	-2.635318	1.614311
75	6	0	9.242777	-1.405555	0.501795
76	1	0	8.286812	0.252326	-0.388520
77	6	0	10.511957	-0.830834	-0.102901
78	1	0	9.340668	-1.461579	1.590132
79	1	0	9.081054	-2.422631	0.131790
80	6	0	11.715719	-1.709522	0.210269
81	1	0	10.374604	-0.739779	-1.182468
82	1	0	10.695561	0.169393	0.296925
83	7	0	12.977358	-1.077527	-0.121737
84	1	0	11.726099	-1.921823	1.282371
85	1	0	11.605341	-2.680435	-0.307342
86	6	0	14.086556	-1.865535	0.385934
87	6	0	13.122761	-0.862828	-1.549979
88	1	0	15.024785	-1.355035	0.174645
89	1	0	13.995074	-1.981787	1.465313
90	1	0	14.129328	-2.867854	-0.069382
91	1	0	14.099555	-0.426056	-1.750761
92	1	0	13.041570	-1.802272	-2.121516
93	1	0	12.368697	-0.168312	-1.916192

Ligand	Description	Total Gibbs Free energy (M06-2X/Def2TZVPP)
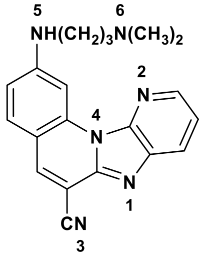 **1a**	2:1 complex with Ca^{2+} bound to N1	-2881.925717 a.u.

CARTESIAN COORDINATES

Center Number	Atomic Number	Atomic Type	Coordinates (Angstroms) X	Y	Z
1	7	0	4.372816	-0.233409	0.533369
2	6	0	5.570195	0.478810	0.386044
3	6	0	3.229352	0.321255	1.032886
4	7	0	2.207486	-0.531872	1.096652
5	6	0	2.718726	-1.730673	0.611107
6	6	0	4.061648	-1.570772	0.250502
7	6	0	3.234643	1.689888	1.430631
8	7	0	4.859665	-2.490663	-0.245002
9	6	0	2.149221	-2.986346	0.445196
10	6	0	4.298899	-3.686225	-0.404151
11	6	0	2.969458	-3.971165	-0.075015
12	1	0	4.934951	-4.461187	-0.810439
13	1	0	2.593782	-4.972051	-0.228810
14	1	0	1.122788	-3.191294	0.718657
15	6	0	5.551621	1.851403	0.791771
16	6	0	4.394853	2.429913	1.304792
17	1	0	4.409924	3.468523	1.608871
18	6	0	6.701139	-0.106109	-0.116394
19	6	0	6.755456	2.590056	0.653977
20	6	0	7.886205	2.027840	0.158383
21	6	0	7.886852	0.656604	-0.241182
22	1	0	6.757047	3.629088	0.957119
23	1	0	8.787912	2.613253	0.064893
24	7	0	8.988889	0.089913	-0.727707
25	1	0	6.690188	-1.142988	-0.414230
26	1	0	8.936693	-0.879404	-1.002180
27	6	0	10.273879	0.753305	-0.921059
28	6	0	11.283351	-0.225379	-1.494478
29	1	0	10.142798	1.600694	-1.599042
30	1	0	10.626759	1.141684	0.038128
31	6	0	12.644144	0.435897	-1.677515
32	1	0	11.361942	-1.082681	-0.822552
33	1	0	10.940091	-0.589429	-2.465888
34	7	0	13.559949	-0.379128	-2.447728
35	1	0	12.507050	1.377293	-2.215726
36	1	0	13.063918	0.691075	-0.686796
37	6	0	14.749206	0.382140	-2.793278
38	6	0	13.925864	-1.600013	-1.751445
39	1	0	15.403395	-0.226694	-3.414694
40	1	0	14.469401	1.270353	-3.358489
41	1	0	15.315067	0.698574	-1.903274
42	1	0	14.644751	-2.154451	-2.351688
43	1	0	14.381996	-1.396918	-0.768823
44	1	0	13.056710	-2.239483	-1.605521
45	6	0	1.992272	2.108331	1.936604
46	7	0	0.901358	2.212463	2.305573
47	20	0	0.000023	-0.000352	1.948189
48	7	0	-2.207465	0.531523	1.096790
49	6	0	-3.229369	-0.321544	1.032918

50	7	0	-4.372845	0.233270	0.533575
51	6	0	-4.061630	1.570667	0.250963
52	6	0	-2.718696	1.730446	0.611531
53	6	0	-2.149123	2.986125	0.445829
54	7	0	-4.859629	2.490704	-0.244328
55	6	0	-5.570257	-0.478872	0.386165
56	6	0	-3.234719	-1.690258	1.430407
57	6	0	-4.394975	-2.430194	1.304502
58	6	0	-5.551735	-1.851535	0.791630
59	6	0	-2.969336	3.971077	-0.074137
60	6	0	-4.298813	3.686257	-0.403262
61	1	0	-1.122656	3.190931	0.719278
62	1	0	-2.593637	4.971981	-0.227775
63	1	0	-4.934839	4.461347	-0.809356
64	6	0	-1.992363	-2.108826	1.936309
65	7	0	-0.901467	-2.213071	2.305291
66	1	0	-4.410093	-3.468858	1.608399
67	6	0	-6.755607	-2.590111	0.653730
68	6	0	-6.701194	0.106196	-0.116139
69	6	0	-7.886341	-2.027752	0.158269
70	6	0	-7.886940	-0.656428	-0.241023
71	7	0	-8.988993	-0.089591	-0.727353
72	1	0	-6.690186	1.143133	-0.413778
73	1	0	-6.757242	-3.629198	0.956691
74	1	0	-8.788083	-2.613102	0.064695
75	6	0	-10.273917	-0.752999	-0.921093
76	1	0	-8.936744	0.879744	-1.001749
77	6	0	-11.283371	0.225802	-1.494342
78	1	0	-10.142679	-1.600175	-1.599313
79	1	0	-10.626922	-1.141692	0.037924
80	6	0	-12.644227	-0.435371	-1.677298
81	1	0	-11.361835	1.083049	-0.822326
82	1	0	-10.940181	0.589900	-2.465760
83	7	0	-13.559782	0.379396	-2.448085
84	1	0	-12.507188	-1.377057	-2.215023
85	1	0	-13.064212	-0.689992	-0.686526
86	6	0	-14.749276	-0.381760	-2.793056
87	6	0	-13.925307	1.600886	-1.752662
88	1	0	-15.403295	0.226818	-3.414903
89	1	0	-14.469751	-1.270471	-3.357623
90	1	0	-15.315213	-0.697374	-1.902807
91	1	0	-14.644003	2.155141	-2.353304
92	1	0	-14.381519	1.398632	-0.769902
93	1	0	-13.055938	2.240167	-1.607178

Ligand	Description	Total Gibbs Free energy (M06-2X/Def2TZVPP)
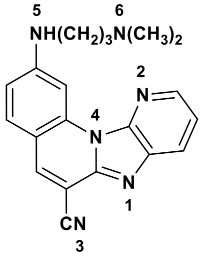 1a	2:1 complex with Cu^{2+} bound to N1	-3844.693378 a.u.

CARTESIAN COORDINATES

Center Number	Atomic Number	Atomic Type	X	Y	Z
1	7	0	4.160885	-0.136076	0.246424
2	6	0	5.320605	0.544790	-0.137577
3	6	0	2.903318	0.400488	0.137791
4	7	0	1.954528	-0.423941	0.557488
5	6	0	2.610753	-1.568549	0.965261
6	6	0	3.990632	-1.416055	0.784476
7	6	0	2.744579	1.716962	-0.385659
8	7	0	4.922356	-2.302547	1.068651
9	6	0	2.148854	-2.766005	1.493365
10	6	0	4.464869	-3.443273	1.575385
11	6	0	3.109674	-3.711047	1.800034
12	1	0	5.210634	-4.188935	1.817493
13	1	0	2.825830	-4.667861	2.212961
14	1	0	1.093843	-2.947882	1.642625
15	6	0	5.144382	1.854490	-0.660689
16	6	0	3.858508	2.412794	-0.772523
17	1	0	3.753227	3.412895	-1.172711
18	6	0	6.568544	-0.021756	-0.017247
19	6	0	6.300550	2.559411	-1.056734
20	6	0	7.545085	2.015543	-0.946578
21	6	0	7.704818	0.704548	-0.420078
22	1	0	6.182413	3.557819	-1.457826
23	1	0	8.410923	2.578743	-1.259362
24	7	0	8.924438	0.154530	-0.302408
25	1	0	6.673125	-1.018012	0.382979
26	1	0	8.982633	-0.783237	0.060224
27	6	0	10.170409	0.786851	-0.700971
28	6	0	11.341214	-0.136431	-0.412159
29	1	0	10.136886	1.024915	-1.768395
30	1	0	10.293969	1.727733	-0.156207
31	6	0	12.663569	0.518682	-0.788617
32	1	0	11.329563	-0.394162	0.648954
33	1	0	11.235286	-1.062298	-0.982799
34	7	0	13.777480	-0.408320	-0.767967
35	1	0	12.582790	0.913636	-1.804527
36	1	0	12.847626	1.382373	-0.122986
37	6	0	14.961923	0.210942	-1.336578
38	6	0	14.056949	-0.898958	0.569401
39	1	0	15.775766	-0.511827	-1.362250
40	1	0	14.757799	0.533484	-2.356961
41	1	0	15.295710	1.085271	-0.755476
42	1	0	14.924463	-1.555850	0.537854
43	1	0	14.270918	-0.079958	1.275894
44	1	0	13.218493	-1.477643	0.953202
45	6	0	1.415948	2.216247	-0.461838
46	7	0	0.308210	2.528638	-0.477856
47	29	0	0.001839	-0.077644	0.501081
48	7	0	-1.968630	0.242738	0.583948
49	6	0	-2.930380	-0.518146	0.100522

50	7	0	-4.178188	0.043741	0.236801
51	6	0	-3.977871	1.274915	0.867713
52	6	0	-2.597091	1.372896	1.069294
53	6	0	-2.103788	2.514488	1.688578
54	7	0	-4.889701	2.165169	1.207996
55	6	0	-5.345380	-0.576045	-0.206696
56	6	0	-2.805877	-1.803063	-0.519066
57	6	0	-3.922588	-2.434486	-0.964793
58	6	0	-5.207717	-1.841207	-0.821503
59	6	0	-3.040269	3.460384	2.056051
60	6	0	-4.401296	3.250066	1.800691
61	1	0	-1.045451	2.653450	1.856513
62	1	0	-2.733561	4.375663	2.540457
63	1	0	-5.125281	3.999762	2.091893
64	6	0	-1.491778	-2.348933	-0.623542
65	7	0	-0.407971	-2.727098	-0.667321
66	1	0	-3.841289	-3.404984	-1.436273
67	6	0	-6.370385	-2.476381	-1.279968
68	6	0	-6.587513	0.016071	-0.053084
69	6	0	-7.606629	-1.899367	-1.140207
70	6	0	-7.727232	-0.642848	-0.514705
71	7	0	-8.956525	-0.047512	-0.322556
72	1	0	-6.661926	0.978836	0.429821
73	1	0	-6.279039	-3.441346	-1.761132
74	1	0	-8.474998	-2.414688	-1.521576
75	6	0	-10.165322	-0.548368	-0.925997
76	1	0	-8.924739	0.943302	-0.141077
77	6	0	-11.362505	0.228752	-0.385952
78	1	0	-10.142380	-0.486071	-2.022040
79	1	0	-10.282264	-1.601600	-0.661848
80	6	0	-12.664438	-0.319946	-1.003674
81	1	0	-11.397593	0.135388	0.700359
82	1	0	-11.269444	1.288684	-0.633618
83	7	0	-13.803408	0.404864	-0.490323
84	1	0	-12.650453	-0.212470	-2.087448
85	1	0	-12.787179	-1.371664	-0.739495
86	6	0	-14.396080	1.455837	-1.278206
87	6	0	-14.332327	0.111997	0.816902
88	1	0	-13.607940	2.042307	-1.752261
89	1	0	-14.994662	0.992788	-2.071760
90	1	0	-15.032880	2.080477	-0.659050
91	1	0	-15.404115	-0.086880	0.725413
92	1	0	-13.821052	-0.740725	1.251418
93	1	0	-14.215601	0.995204	1.452598

Ligand	Description	Total Gibbs Free energy (M06-2X/Def2TZVPP)
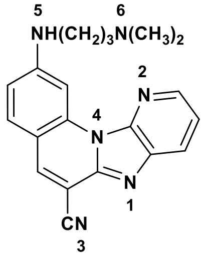 **1a**	2:1 complex with Mg^{2+} bound to N1	-2404.365318 a.u.

CARTESIAN COORDINATES

Center Number	Atomic Number	Atomic Type	Coordinates (Angstroms) X	Y	Z
1	7	0	4.001013	0.414706	0.126131
2	6	0	5.285049	0.622861	-0.396722
3	6	0	2.908075	1.101535	-0.305505
4	7	0	1.784620	0.759696	0.331726
5	6	0	2.169725	-0.217160	1.241830
6	6	0	3.544836	-0.451012	1.133662
7	6	0	3.043637	2.073553	-1.336454
8	7	0	4.253465	-1.306307	1.835244
9	6	0	1.456759	-0.949072	2.180127
10	6	0	3.558595	-2.002046	2.731621
11	6	0	2.181824	-1.855775	2.932433
12	1	0	4.119925	-2.712850	3.322960
13	1	0	1.693622	-2.459675	3.683053
14	1	0	0.391273	-0.820917	2.320406
15	6	0	5.401846	1.607213	-1.431234
16	6	0	4.290816	2.312151	-1.881917
17	1	0	4.409280	3.051521	-2.663249
18	6	0	6.370259	-0.074496	0.058976
19	6	0	6.695085	1.834166	-1.971820
20	6	0	7.782732	1.153575	-1.532918
21	6	0	7.647418	0.177005	-0.498704
22	1	0	6.800551	2.574368	-2.754401
23	1	0	8.753785	1.347206	-1.962020
24	7	0	8.706155	-0.495172	-0.055277
25	1	0	6.255527	-0.809742	0.840246
26	1	0	8.558199	-1.178633	0.672026
27	6	0	10.075217	-0.346597	-0.539347
28	6	0	11.000253	-1.278345	0.223367
29	1	0	10.105237	-0.575415	-1.607753
30	1	0	10.392091	0.691461	-0.408327
31	6	0	12.442354	-1.120641	-0.243872
32	1	0	10.917687	-1.059321	1.290054
33	1	0	10.696067	-2.316306	0.068127
34	7	0	13.311882	-2.154184	0.277153
35	1	0	12.466580	-1.187143	-1.334587
36	1	0	12.806092	-0.111325	0.024092
37	6	0	14.609203	-2.103428	-0.377160
38	6	0	13.468967	-2.063950	1.718244
39	1	0	15.233268	-2.916865	-0.011657
40	1	0	14.486627	-2.222843	-1.452965
41	1	0	15.134260	-1.154199	-0.188606
42	1	0	14.160987	-2.834292	2.053115
43	1	0	13.865756	-1.084821	2.032285
44	1	0	12.519627	-2.232673	2.224173
45	6	0	1.799400	2.662345	-1.623208
46	7	0	0.675101	2.932480	-1.613582
47	12	0	0.000014	1.742839	0.000012
48	7	0	-1.784583	0.759639	-0.331554
49	6	0	-2.908101	1.101715	0.305439

50	7	0	-4.001026	0.414842	-0.126162
51	6	0	-3.544772	-0.451169	-1.133408
52	6	0	-2.169636	-0.217404	-1.241472
53	6	0	-1.456594	-0.949540	-2.179531
54	7	0	-4.253358	-1.306589	-1.834875
55	6	0	-5.285119	0.623211	0.396475
56	6	0	-3.043747	2.074061	1.336063
57	6	0	-4.290998	2.312948	1.881239
58	6	0	-5.402009	1.607930	1.430625
59	6	0	-2.181613	-1.856375	-2.931726
60	6	0	-3.558415	-2.002547	-2.731031
61	1	0	-0.391090	-0.821424	-2.319712
62	1	0	-1.693355	-2.460458	-3.682163
63	1	0	-4.119708	-2.713471	-3.322263
64	6	0	-1.799511	2.662854	1.622836
65	7	0	-0.675192	2.932909	1.613289
66	1	0	-4.409530	3.052601	2.662291
67	6	0	-6.695312	1.835129	1.970955
68	6	0	-6.370290	-0.074289	-0.059095
69	6	0	-7.782924	1.154407	1.532168
70	6	0	-7.647506	0.177429	0.498355
71	7	0	-8.706199	-0.494930	0.055085
72	1	0	-6.255486	-0.809804	-0.840099
73	1	0	-6.800857	2.575634	2.753237
74	1	0	-8.754028	1.348251	1.961059
75	6	0	-10.075296	-0.346223	0.539012
76	1	0	-8.558171	-1.178637	-0.671970
77	6	0	-11.000192	-1.278545	-0.223169
78	1	0	-10.105327	-0.574409	1.607554
79	1	0	-10.392288	0.691719	0.407369
80	6	0	-12.442315	-1.120803	0.243982
81	1	0	-10.917659	-1.060130	-1.289983
82	1	0	-10.695845	-2.316369	-0.067335
83	7	0	-13.311721	-2.154612	-0.276710
84	1	0	-12.466556	-1.186923	1.334719
85	1	0	-12.806148	-0.111617	-0.024341
86	6	0	-14.608975	-2.103973	0.377748
87	6	0	-13.468999	-2.064698	-1.717800
88	1	0	-15.232939	-2.917578	0.012443
89	1	0	-14.486258	-2.223197	1.453558
90	1	0	-15.134222	-1.154870	0.189097
91	1	0	-14.160795	-2.835335	-2.052454
92	1	0	-13.866150	-1.085759	-2.031973
93	1	0	-12.519657	-2.233186	-2.223804

Ligand	Description	Total Gibbs Free energy (M06-2X/Def2TZVPP)
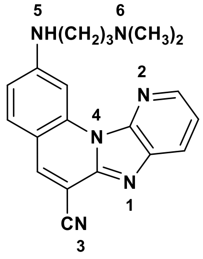<br>**1a**	2:1 complex with Zn^{2+} bound to N1	-3983.621700 a.u.

CARTESIAN COORDINATES

Center Number	Atomic Number	Atomic Type	Coordinates (Angstroms) X	Y	Z
1	7	0	4.029814	0.219978	0.122333
2	6	0	5.300959	0.520748	-0.387529
3	6	0	2.899220	0.842547	-0.306992
4	7	0	1.801017	0.407479	0.317310
5	6	0	2.237671	-0.556641	1.214353
6	6	0	3.625732	-0.693881	1.110678
7	6	0	2.972644	1.842627	-1.314514
8	7	0	4.384549	-1.512504	1.802869
9	6	0	1.567705	-1.348760	2.133903
10	6	0	3.732012	-2.268935	2.681844
11	6	0	2.347319	-2.218307	2.875526
12	1	0	4.335958	-2.950966	3.264970
13	1	0	1.896056	-2.866213	3.612421
14	1	0	0.495360	-1.291522	2.268242
15	6	0	5.358484	1.532269	-1.399919
16	6	0	4.205849	2.172782	-1.843464
17	1	0	4.280794	2.934505	-2.608516
18	6	0	6.426188	-0.116572	0.059471
19	6	0	6.636742	1.852781	-1.928150
20	6	0	7.763460	1.232311	-1.498523
21	6	0	7.686577	0.226013	-0.487133
22	1	0	6.698370	2.615282	-2.693779
23	1	0	8.721920	1.496455	-1.918206
24	7	0	8.784168	-0.389540	-0.055541
25	1	0	6.355237	-0.874393	0.824093
26	1	0	8.677681	-1.097220	0.655610
27	6	0	10.141573	-0.148391	-0.535124
28	6	0	11.120088	-1.047043	0.200020
29	1	0	10.182657	-0.344664	-1.609617
30	1	0	10.398570	0.902032	-0.374708
31	6	0	12.549378	-0.796413	-0.266063
32	1	0	11.029362	-0.862243	1.272504
33	1	0	10.873878	-2.095777	0.016895
34	7	0	13.477082	-1.793570	0.224639
35	1	0	12.572989	-0.832084	-1.358237
36	1	0	12.857511	0.224007	0.028189
37	6	0	14.766589	-1.654098	-0.432202
38	6	0	13.635380	-1.732922	1.667148
39	1	0	15.436292	-2.440920	-0.090007
40	1	0	14.645831	-1.752317	-1.510354
41	1	0	15.239347	-0.682461	-0.220769
42	1	0	14.370440	-2.472361	1.979246
43	1	0	13.978816	-0.741999	2.006032
44	1	0	12.699075	-1.967413	2.171153
45	6	0	1.695602	2.357508	-1.610214
46	7	0	0.556875	2.561415	-1.623007
47	30	0	-0.000010	1.244135	0.000111
48	7	0	-1.800975	0.407358	-0.316999
49	6	0	-2.899198	0.842328	0.307339

50	7	0	-4.029783	0.219869	-0.122140
51	6	0	-3.625692	-0.693784	-1.110694
52	6	0	-2.237613	-0.556531	-1.214300
53	6	0	-1.567654	-1.348427	-2.134031
54	7	0	-4.384499	-1.512220	-1.803086
55	6	0	-5.300946	0.520604	0.387695
56	6	0	-2.972639	1.842303	1.314972
57	6	0	-4.205874	2.172458	1.843862
58	6	0	-5.358498	1.532021	1.400189
59	6	0	-2.347268	-2.217770	-2.875905
60	6	0	-3.731956	-2.268435	-2.682270
61	1	0	-0.495300	-1.291189	-2.268304
62	1	0	-1.895976	-2.865493	-3.612942
63	1	0	-4.335910	-2.950311	-3.265560
64	6	0	-1.695623	2.357258	1.610653
65	7	0	-0.556921	2.561304	1.623408
66	1	0	-4.280845	2.934144	2.608949
67	6	0	-6.636786	1.852529	1.928372
68	6	0	-6.426167	-0.116643	-0.059429
69	6	0	-7.763495	1.232140	1.498620
70	6	0	-7.686580	0.225930	0.487131
71	7	0	-8.784160	-0.389567	0.055441
72	1	0	-6.355200	-0.874394	-0.824120
73	1	0	-6.698425	2.614964	2.694065
74	1	0	-8.721977	1.496262	1.918263
75	6	0	-10.141596	-0.148410	0.534947
76	1	0	-8.677656	-1.097169	-0.655786
77	6	0	-11.120029	-1.047210	-0.200124
78	1	0	-10.182692	-0.344556	1.609461
79	1	0	-10.398636	0.901981	0.374396
80	6	0	-12.549326	-0.796806	0.266041
81	1	0	-11.029390	-0.862417	-1.272616
82	1	0	-10.873649	-2.095903	-0.017013
83	7	0	-13.477063	-1.793535	-0.225455
84	1	0	-12.572985	-0.833287	1.358185
85	1	0	-12.857371	0.223858	-0.027463
86	6	0	-14.766402	-1.654943	0.431909
87	6	0	-13.635803	-1.731358	-1.667850
88	1	0	-15.436114	-2.441451	0.089007
89	1	0	-14.645337	-1.754368	1.509913
90	1	0	-15.239334	-0.683125	0.221699
91	1	0	-14.370747	-2.470662	-1.980541
92	1	0	-13.979619	-0.740160	-2.005538
93	1	0	-12.699589	-1.965028	-2.172399

Ligand	Description	Total Gibbs Free energy (M06-2X/Def2TZVPP)
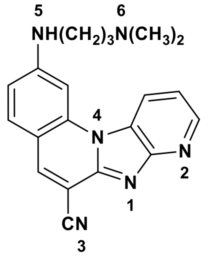 **1b**	2:1 complex with H^+ bound to N1	-2205.037557 a.u.

CARTESIAN COORDINATES

Center Number	Atomic Number	Atomic Type	Coordinates (Angstroms) X	Y	Z
1	7	0	3.495328	-0.600083	-0.011793
2	6	0	4.570067	0.196782	-0.411292
3	6	0	2.195410	-0.344148	-0.410901
4	7	0	1.331963	-1.212670	0.055280
5	6	0	2.060984	-2.088705	0.817638
6	6	0	3.426157	-1.735050	0.798804
7	6	0	1.916396	0.790695	-1.232301
8	6	0	4.322235	-2.501336	1.532497
9	7	0	1.544713	-3.109613	1.485902
10	6	0	3.784636	-3.570981	2.226508
11	6	0	2.412074	-3.833965	2.175278
12	1	0	4.425666	-4.207603	2.818174
13	1	0	2.005734	-4.672934	2.726683
14	6	0	4.273980	1.318717	-1.225357
15	6	0	2.943080	1.596215	-1.615836
16	1	0	2.747552	2.465268	-2.230165
17	6	0	5.872949	-0.079350	-0.046317
18	6	0	5.342528	2.137546	-1.632965
19	6	0	6.633508	1.877929	-1.272391
20	6	0	6.924415	0.750998	-0.465179
21	1	0	5.121185	2.996258	-2.253765
22	1	0	7.429908	2.525969	-1.604308
23	7	0	8.193397	0.472767	-0.097201
24	1	0	6.110190	-0.936678	0.557505
25	1	0	8.356932	-0.356293	0.448650
26	6	0	9.358569	1.237791	-0.498900
27	6	0	10.614913	0.635063	0.105621
28	1	0	9.438727	1.248029	-1.590601
29	1	0	9.248752	2.275218	-0.167931
30	6	0	11.847924	1.448321	-0.264231
31	1	0	10.494693	0.593064	1.190245
32	1	0	10.749264	-0.387384	-0.256140
33	7	0	13.089601	0.778851	0.071568
34	1	0	11.846588	1.616427	-1.344144
35	1	0	11.787806	2.443185	0.214711
36	6	0	14.219687	1.501746	-0.483889
37	6	0	13.251610	0.612979	1.504296
38	1	0	15.140826	0.962394	-0.268694
39	1	0	14.113360	1.581063	-1.565271
40	1	0	14.310265	2.518133	-0.068206
41	1	0	14.214114	0.145942	1.706342
42	1	0	13.216818	1.575928	2.040525
43	1	0	12.477333	-0.036493	1.908871
44	6	0	0.554930	1.033236	-1.579092
45	7	0	-0.555715	1.197238	-1.823248
46	1	0	-0.293817	-1.240147	0.002995
47	7	0	-1.370830	-1.248759	-0.068285
48	6	0	-2.193145	-0.303106	0.363946
49	7	0	-3.480054	-0.574651	-0.019810

50	6	0	-3.445206	-1.755416	-0.778344
51	6	0	-2.102741	-2.161243	-0.791942
52	7	0	-1.609787	-3.217453	-1.403936
53	6	0	-4.363106	-2.517572	-1.489613
54	6	0	-4.549260	0.251476	0.343311
55	6	0	-1.877450	0.864926	1.101750
56	6	0	-2.898938	1.714620	1.419220
57	6	0	-4.229803	1.431497	1.060309
58	6	0	-2.499157	-3.944892	-2.064543
59	6	0	-3.859968	-3.633459	-2.135116
60	1	0	-2.117057	-4.819993	-2.574370
61	1	0	-4.520806	-4.268032	-2.706590
62	6	0	-0.522180	1.133860	1.455112
63	7	0	0.568747	1.354093	1.735142
64	1	0	-2.683312	2.622948	1.966265
65	6	0	-5.287874	2.291241	1.420170
66	6	0	-5.859291	-0.056705	0.039960
67	6	0	-6.583644	2.007585	1.110184
68	6	0	-6.899229	0.810675	0.414935
69	7	0	-8.175602	0.503889	0.118273
70	1	0	-6.116144	-0.969849	-0.465141
71	1	0	-5.049116	3.196723	1.962709
72	1	0	-7.371338	2.685245	1.401238
73	6	0	-9.331912	1.303915	0.484758
74	1	0	-8.356617	-0.365787	-0.354908
75	6	0	-10.605018	0.637399	-0.006892
76	1	0	-9.368408	1.421839	1.571919
77	1	0	-9.240809	2.302253	0.046430
78	6	0	-11.830251	1.476491	0.331201
79	1	0	-10.528082	0.489635	-1.086223
80	1	0	-10.715421	-0.344837	0.459172
81	7	0	-13.076092	0.769438	0.109875
82	1	0	-11.788740	1.747165	1.389260
83	1	0	-11.797302	2.421160	-0.242584
84	6	0	-14.191479	1.529865	0.645793
85	6	0	-13.295580	0.473122	-1.294124
86	1	0	-15.113538	0.964807	0.519241
87	1	0	-14.041586	1.707196	1.710201
88	1	0	-14.312068	2.503352	0.144425
89	1	0	-14.259142	-0.019686	-1.411717
90	1	0	-13.296462	1.383789	-1.915688
91	1	0	-12.530642	-0.203208	-1.671755
92	1	0	5.378967	-2.306747	1.594511
93	1	0	-5.408847	-2.281234	-1.576641

Ligand	Description	Total Gibbs Free energy (M06-2X/Def2TZVPP)
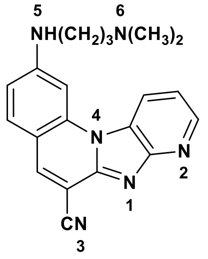 **1b**	2:1 complex with Ca^{2+} bound to N1	-2881.932868 a.u.

CARTESIAN COORDINATES

Center Number	Atomic Number	Atomic Type	Coordinates (Angstroms) X	Y	Z
1	7	0	4.486809	0.290214	0.120817
2	6	0	5.763542	0.551110	-0.393442
3	6	0	3.379721	0.951268	-0.344014
4	7	0	2.259227	0.584113	0.253731
5	6	0	2.634999	-0.371380	1.167331
6	6	0	4.022819	-0.585679	1.116257
7	6	0	3.487544	1.930304	-1.369329
8	6	0	4.573336	-1.523483	1.979254
9	7	0	1.778941	-0.980538	1.969263
10	6	0	3.683795	-2.168461	2.823981
11	6	0	2.317455	-1.874703	2.790743
12	1	0	4.046266	-2.909223	3.520954
13	1	0	1.638583	-2.387509	3.459322
14	6	0	5.856471	1.536722	-1.424382
15	6	0	4.723974	2.210609	-1.896022
16	1	0	4.834676	2.952265	-2.676170
17	6	0	6.882613	-0.100223	0.061937
18	6	0	7.141423	1.810955	-1.953879
19	6	0	8.255518	1.172947	-1.511796
20	6	0	8.153192	0.192919	-0.482563
21	1	0	7.222848	2.555448	-2.735298
22	1	0	9.219517	1.405299	-1.937091
23	7	0	9.238598	-0.441524	-0.034876
24	1	0	6.817909	-0.842697	0.837954
25	1	0	9.120874	-1.133699	0.688292
26	6	0	10.598922	-0.239177	-0.521919
27	6	0	11.560756	-1.137648	0.235246
28	1	0	10.636288	-0.463275	-1.591236
29	1	0	10.877895	0.809489	-0.388368
30	6	0	12.994486	-0.927353	-0.236799
31	1	0	11.474554	-0.923798	1.302772
32	1	0	11.294260	-2.185869	0.079023
33	7	0	13.903306	-1.928711	0.280582
34	1	0	13.017100	-0.992516	-1.327572
35	1	0	13.322348	0.094324	0.030340
36	6	0	15.195015	-1.831208	-0.379464
37	6	0	14.063559	-1.832129	1.720826
38	1	0	15.849896	-2.621187	-0.016401
39	1	0	15.072091	-1.955592	-1.454657
40	1	0	15.686019	-0.863371	-0.193723
41	1	0	14.784530	-2.576749	2.052899
42	1	0	14.426006	-0.839122	2.032836
43	1	0	13.123161	-2.034759	2.231016
44	6	0	2.219517	2.478743	-1.675929
45	7	0	1.096310	2.737171	-1.711643
46	20	0	0.000012	1.337804	0.000582
47	7	0	-2.259279	0.584615	-0.253510
48	6	0	-3.379670	0.950769	0.345042
49	7	0	-4.486820	0.290405	-0.120616

50	6	0	-4.022992	-0.583886	-1.117533
51	6	0	-2.635192	-0.369454	-1.168543
52	7	0	-1.779273	-0.977290	-1.971628
53	6	0	-4.573664	-1.520273	-1.981974
54	6	0	-5.763465	0.550445	0.394300
55	6	0	-3.487329	1.928178	1.371924
56	6	0	-4.723672	2.207635	1.899275
57	6	0	-5.856238	1.534469	1.426773
58	6	0	-2.317926	-1.870130	-2.794457
59	6	0	-3.684270	-2.163856	-2.827922
60	1	0	-1.639170	-2.381826	-3.464007
61	1	0	-4.046853	-2.903476	-3.526048
62	6	0	-2.219248	2.476091	1.679223
63	7	0	-1.096024	2.734408	1.715204
64	1	0	-4.834246	2.948061	2.680608
65	6	0	-7.141104	1.807852	1.956916
66	6	0	-6.882588	-0.100251	-0.061849
67	6	0	-8.255261	1.170492	1.514052
68	6	0	-8.153085	0.192034	0.483311
69	7	0	-9.238544	-0.441763	0.034842
70	1	0	-6.817996	-0.841587	-0.838965
71	1	0	-7.222407	2.551131	2.739503
72	1	0	-9.219195	1.402159	1.939871
73	6	0	-10.598830	-0.240053	0.522255
74	1	0	-9.120925	-1.132796	-0.689435
75	6	0	-11.560728	-1.137442	-0.236115
76	1	0	-10.636132	-0.465646	1.591259
77	1	0	-10.877783	0.808809	0.390195
78	6	0	-12.994425	-0.927773	0.236272
79	1	0	-11.474566	-0.922094	-1.303345
80	1	0	-11.294227	-2.185882	-0.081388
81	7	0	-13.903379	-1.928134	-0.282811
82	1	0	-13.017048	-0.994803	1.326929
83	1	0	-13.322150	0.094405	-0.029127
84	6	0	-15.195083	-1.831561	0.377401
85	6	0	-14.063686	-1.829073	-1.722884
86	1	0	-15.850007	-2.620966	0.013165
87	1	0	-15.072155	-1.957549	1.452404
88	1	0	-15.686029	-0.863419	0.193099
89	1	0	-14.784748	-2.573054	-2.056187
90	1	0	-14.426054	-0.835496	-2.033167
91	1	0	-13.123341	-2.030922	-2.233477
92	1	0	5.621435	-1.766338	2.024414
93	1	0	-5.621770	-1.763058	-2.027342

Ligand	Description	Total Gibbs Free energy (M06-2X/Def2TZVPP)
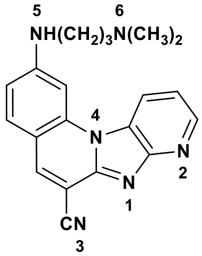 **1b**	2:1 complex with Cu^{2+} bound to N1	-3844.681599 a.u.

CARTESIAN COORDINATES

Center	Atomic	Atomic	Coordinates (Angstroms)		
Number	Number	Type	X	Y	Z
1	7	0	4.092563	0.611338	0.010103
2	6	0	5.107537	-0.289445	0.340947
3	6	0	2.773456	0.391580	0.355005
4	7	0	1.976776	1.358985	-0.042395
5	6	0	2.778503	2.269176	-0.688158
6	6	0	4.114677	1.831340	-0.674148
7	6	0	2.407243	-0.790393	1.069667
8	6	0	5.074478	2.622308	-1.294976
9	7	0	2.337459	3.383879	-1.245501
10	6	0	4.618928	3.791926	-1.875555
11	6	0	3.261612	4.130449	-1.828595
12	1	0	5.312637	4.452561	-2.374036
13	1	0	2.918644	5.048225	-2.289179
14	6	0	4.728331	-1.463326	1.035388
15	6	0	3.374873	-1.689338	1.389082
16	1	0	3.116959	-2.591655	1.927503
17	6	0	6.433437	-0.063811	0.018295
18	6	0	5.733767	-2.384630	1.373474
19	6	0	7.045990	-2.172950	1.054677
20	6	0	7.418941	-0.996227	0.366707
21	1	0	5.448793	-3.283380	1.904838
22	1	0	7.790918	-2.902394	1.333228
23	7	0	8.716410	-0.765592	0.033439
24	1	0	6.732956	0.828544	-0.501355
25	1	0	8.942724	0.124389	-0.376846
26	6	0	9.816197	-1.621165	0.421507
27	6	0	11.130170	-1.056227	-0.097158
28	1	0	9.859335	-1.716298	1.512095
29	1	0	9.663725	-2.624827	0.012211
30	6	0	12.283385	-1.985255	0.265106
31	1	0	11.060284	-0.937089	-1.179687
32	1	0	11.309960	-0.071752	0.341835
33	7	0	13.589445	-1.432815	-0.003014
34	1	0	12.244020	-2.231237	1.327452
35	1	0	12.173245	-2.935590	-0.284955
36	6	0	14.703353	-2.052516	0.666893
37	6	0	13.840151	-0.870983	-1.304336
38	1	0	15.578354	-1.409227	0.601713
39	1	0	14.457340	-2.230745	1.711606
40	1	0	14.953206	-3.017937	0.201624
41	1	0	14.838971	-0.443817	-1.330153
42	1	0	13.771818	-1.645217	-2.083600
43	1	0	13.114472	-0.093416	-1.535244
44	6	0	1.033979	-0.957758	1.421479
45	7	0	-0.081063	-1.043964	1.683561
46	29	0	-0.000006	1.426448	-0.000304
47	7	0	-1.976813	1.358786	0.042063
48	6	0	-2.773475	0.391220	-0.354923
49	7	0	-4.092527	0.610884	-0.009714

50	6	0	-4.114581	1.830947	0.674418
51	6	0	-2.778471	2.268972	0.687923
52	7	0	-2.337394	3.383783	1.245037
53	6	0	-5.074252	2.621705	1.295734
54	6	0	-5.107482	-0.290003	-0.340278
55	6	0	-2.407292	-0.790930	-1.069339
56	6	0	-3.374830	-1.690147	-1.388137
57	6	0	-4.728290	-1.464143	-1.034246
58	6	0	-3.261431	4.130220	1.828459
59	6	0	-4.618670	3.791421	1.876067
60	1	0	-2.918441	5.048087	2.288847
61	1	0	-5.312261	4.451908	2.374908
62	6	0	-1.034109	-0.958163	-1.421576
63	7	0	0.080840	-1.044313	-1.684046
64	1	0	-3.116918	-2.592629	-1.926280
65	6	0	-5.733692	-2.385587	-1.371927
66	6	0	-6.433434	-0.064194	-0.017844
67	6	0	-7.045949	-2.173752	-1.053236
68	6	0	-7.418888	-0.996750	-0.365839
69	7	0	-8.716433	-0.766035	-0.032212
70	1	0	-6.732958	0.828411	0.501385
71	1	0	-5.448734	-3.284516	-1.902998
72	1	0	-7.790856	-2.903269	-1.331654
73	6	0	-9.816354	-1.620298	-0.422785
74	1	0	-8.943013	0.126027	0.373433
75	6	0	-11.130273	-1.056414	0.097202
76	1	0	-9.859473	-1.713093	-1.513619
77	1	0	-9.664072	-2.624824	-0.015654
78	6	0	-12.283552	-1.984481	-0.267291
79	1	0	-11.060347	-0.939793	1.179991
80	1	0	-11.309986	-0.070889	-0.339507
81	7	0	-13.589608	-1.432919	0.002697
82	1	0	-12.244511	-2.227824	-1.330255
83	1	0	-12.173403	-2.936188	0.280370
84	6	0	-14.703930	-2.050075	-0.668818
85	6	0	-13.839631	-0.872187	1.304608
86	1	0	-15.578344	-1.406107	-0.602463
87	1	0	-14.457767	-2.226434	-1.713808
88	1	0	-14.954737	-3.016199	-0.205540
89	1	0	-14.839132	-0.446766	1.331960
90	1	0	-13.768824	-1.646783	2.083264
91	1	0	-13.114871	-0.093576	1.535025
92	1	0	6.120907	2.377214	-1.350517
93	1	0	-6.120577	2.376297	1.351914

Ligand	Description	Total Gibbs Free energy (M06-2X/Def2TZVPP)
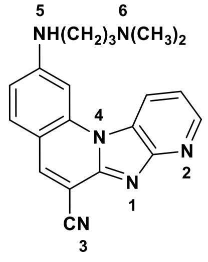 1b	2:1 complex with Mg^{2+} bound to N1	-2404.365219 a.u.

CARTESIAN COORDINATES

Center Number	Atomic Number	Atomic Type	Coordinates (Angstroms) X	Y	Z
1	7	0	4.025755	0.446746	0.179595
2	6	0	5.307157	0.619183	-0.361895
3	6	0	2.954368	1.158935	-0.282741
4	7	0	1.825317	0.873799	0.350665
5	6	0	2.160545	-0.081706	1.287390
6	6	0	3.531974	-0.374491	1.208463
7	6	0	3.102066	2.101464	-1.337615
8	6	0	4.053343	-1.320844	2.080396
9	7	0	1.296104	-0.622823	2.123935
10	6	0	3.153525	-1.894737	2.963109
11	6	0	1.805580	-1.524717	2.954344
12	1	0	3.492851	-2.637592	3.669498
13	1	0	1.115869	-1.981740	3.651832
14	6	0	5.439807	1.568384	-1.423945
15	6	0	4.343144	2.293630	-1.895398
16	1	0	4.481117	3.005978	-2.698068
17	6	0	6.395018	-0.079899	0.095880
18	6	0	6.730064	1.753496	-1.981644
19	6	0	7.812807	1.067039	-1.537347
20	6	0	7.671886	0.125635	-0.475901
21	1	0	6.839621	2.469283	-2.786077
22	1	0	8.781497	1.231352	-1.983233
23	7	0	8.726141	-0.553495	-0.023823
24	1	0	6.303874	-0.793312	0.895609
25	1	0	8.581430	-1.215591	0.722586
26	6	0	10.089308	-0.443659	-0.534348
27	6	0	11.010418	-1.366544	0.243773
28	1	0	10.098646	-0.707204	-1.595164
29	1	0	10.426190	0.592184	-0.441643
30	6	0	12.445399	-1.260825	-0.258766
31	1	0	10.955789	-1.105855	1.302904
32	1	0	10.680664	-2.402656	0.134334
33	7	0	13.304258	-2.287260	0.293334
34	1	0	12.444973	-1.379139	-1.345279
35	1	0	12.835216	-0.248089	-0.046516
36	6	0	14.584136	-2.304036	-0.396421
37	6	0	13.503355	-2.125339	1.722948
38	1	0	15.198218	-3.112697	-0.004366
39	1	0	14.429293	-2.476941	-1.460702
40	1	0	15.136969	-1.359959	-0.272679
41	1	0	14.183981	-2.895616	2.080539
42	1	0	13.933981	-1.142256	1.973412
43	1	0	12.564273	-2.240766	2.261959
44	6	0	1.856637	2.704905	-1.618580
45	7	0	0.737583	2.985547	-1.584037
46	12	0	-0.000053	1.741324	-0.000026
47	7	0	-1.825300	0.873570	-0.350670
48	6	0	-2.954275	1.158385	0.283028
49	7	0	-4.025661	0.446206	-0.179328

50	6	0	-3.531965	-0.374670	-1.208529
51	6	0	-2.160587	-0.081690	-1.287622
52	7	0	-1.296223	-0.622461	-2.124467
53	6	0	-4.053349	-1.320893	-2.080593
54	6	0	-5.307008	0.618406	0.362367
55	6	0	-3.101887	2.100549	1.338228
56	6	0	-4.342891	2.292407	1.896285
57	6	0	-5.439571	1.567237	1.424760
58	6	0	-1.805727	-1.524191	-2.955036
59	6	0	-3.153614	-1.894418	-2.963630
60	1	0	-1.116076	-1.980930	-3.652770
61	1	0	-3.492964	-2.637143	-3.670145
62	6	0	-1.856488	2.704062	1.619195
63	7	0	-0.737482	2.984909	1.584670
64	1	0	-4.480796	3.004476	2.699213
65	6	0	-6.729771	1.752095	1.982683
66	6	0	-6.394905	-0.080525	-0.095551
67	6	0	-7.812545	1.065777	1.538250
68	6	0	-7.671725	0.124809	0.476407
69	7	0	-8.726031	-0.554107	0.024136
70	1	0	-6.303846	-0.793605	-0.895585
71	1	0	-6.839257	2.467562	2.787408
72	1	0	-8.781178	1.229841	1.984357
73	6	0	-10.089182	-0.444361	0.534725
74	1	0	-8.581347	-1.216099	-0.722370
75	6	0	-11.010496	-1.366356	-0.244210
76	1	0	-10.098642	-0.708761	1.595331
77	1	0	-10.425781	0.591643	0.442844
78	6	0	-12.445436	-1.260879	0.258496
79	1	0	-10.955850	-1.104707	-1.303099
80	1	0	-10.680936	-2.402634	-0.135746
81	7	0	-13.304601	-2.286301	-0.295011
82	1	0	-12.445009	-1.380680	1.344843
83	1	0	-12.834960	-0.247739	0.047656
84	6	0	-14.584203	-2.304219	0.395231
85	6	0	-13.504291	-2.121880	-1.724258
86	1	0	-15.198507	-3.112116	0.001952
87	1	0	-14.428953	-2.479073	1.459134
88	1	0	-15.137014	-1.359878	0.273415
89	1	0	-14.184836	-2.891706	-2.082975
90	1	0	-13.935274	-1.138466	-1.972787
91	1	0	-12.565381	-2.236073	-2.263829
92	1	0	5.086789	-1.621180	2.104314
93	1	0	-5.086741	-1.621422	-2.104362

Ligand	Description	Total Gibbs Free energy (M06-2X/Def2TZVPP)
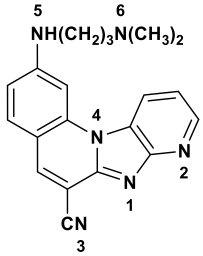 **1b**	2:1 complex with Zn^{2+} bound to N1	-3983.622007 a.u.

CARTESIAN COORDINATES

Center Number	Atomic Number	Atomic Type	Coordinates (Angstroms) X	Y	Z
1	7	0	4.038173	0.265286	0.168501
2	6	0	5.311216	0.520069	-0.361315
3	6	0	2.930978	0.916717	-0.296197
4	7	0	1.820769	0.546289	0.327618
5	6	0	2.202179	-0.397311	1.257420
6	6	0	3.589623	-0.598352	1.184438
7	6	0	3.025589	1.881705	-1.334497
8	6	0	4.164050	-1.516791	2.053334
9	7	0	1.367464	-0.999123	2.080056
10	6	0	3.296380	-2.156487	2.922863
11	6	0	1.927255	-1.874967	2.906146
12	1	0	3.678083	-2.881975	3.625656
13	1	0	1.262605	-2.382187	3.593089
14	6	0	5.394274	1.494101	-1.404924
15	6	0	4.258791	2.158430	-1.874319
16	1	0	4.359552	2.890810	-2.664402
17	6	0	6.434403	-0.125719	0.089719
18	6	0	6.674767	1.764346	-1.950042
19	6	0	7.792543	1.132482	-1.511583
20	6	0	7.700134	0.162548	-0.470641
21	1	0	6.747384	2.500319	-2.740291
22	1	0	8.752640	1.360708	-1.947832
23	7	0	8.789001	-0.466383	-0.027891
24	1	0	6.379700	-0.860843	0.872795
25	1	0	8.679062	-1.150689	0.704251
26	6	0	10.145577	-0.269795	-0.529961
27	6	0	11.113401	-1.162536	0.226257
28	1	0	10.171520	-0.503958	-1.597346
29	1	0	10.426159	0.779804	-0.408441
30	6	0	12.542614	-0.959346	-0.262742
31	1	0	11.038705	-0.938006	1.292457
32	1	0	10.843714	-2.211784	0.082943
33	7	0	13.454492	-1.956990	0.255921
34	1	0	12.553847	-1.036095	-1.352946
35	1	0	12.874623	0.064586	-0.009734
36	6	0	14.738989	-1.871017	-0.419943
37	6	0	13.631476	-1.844081	1.693113
38	1	0	15.395509	-2.658809	-0.055184
39	1	0	14.603470	-2.007478	-1.492140
40	1	0	15.234888	-0.902690	-0.250884
41	1	0	14.353900	-2.587066	2.025588
42	1	0	14.000492	-0.848696	1.989266
43	1	0	12.696432	-2.037878	2.216444
44	6	0	1.751253	2.416437	-1.632972
45	7	0	0.617950	2.638098	-1.628082
46	30	0	-0.000159	1.283774	-0.010113
47	7	0	-1.820301	0.537502	-0.332976
48	6	0	-2.931590	0.921983	0.280309
49	7	0	-4.037825	0.259262	-0.170542

50	6	0	-3.587314	-0.628270	-1.164792
51	6	0	-2.199876	-0.428210	-1.240558
52	7	0	-1.363570	-1.049345	-2.047071
53	6	0	-4.159774	-1.568410	-2.011471
54	6	0	-5.311854	0.526347	0.350752
55	6	0	-3.028097	1.910643	1.295934
56	6	0	-4.262130	2.198953	1.827732
57	6	0	-5.396720	1.523714	1.371923
58	6	0	-1.921556	-1.945576	-2.852224
59	6	0	-3.290424	-2.228602	-2.863788
60	1	0	-1.255617	-2.468982	-3.525626
61	1	0	-3.670553	-2.971684	-3.548828
62	6	0	-1.754312	2.452379	1.583906
63	7	0	-0.621018	2.673974	1.575338
64	1	0	-4.364259	2.949017	2.600868
65	6	0	-6.678157	1.806198	1.908559
66	6	0	-6.434381	-0.128962	-0.088070
67	6	0	-7.795220	1.164853	1.482261
68	6	0	-7.701106	0.172036	0.463279
69	7	0	-8.789369	-0.466168	0.032473
70	1	0	-6.378443	-0.880952	-0.854867
71	1	0	-6.752119	2.559718	2.681965
72	1	0	-8.756064	1.402922	1.911533
73	6	0	-10.146830	-0.257698	0.527307
74	1	0	-8.678315	-1.166659	-0.684013
75	6	0	-11.113613	-1.167436	-0.209725
76	1	0	-10.174819	-0.467094	1.599768
77	1	0	-10.426844	0.788892	0.381050
78	6	0	-12.543724	-0.951496	0.271162
79	1	0	-11.036366	-0.968383	-1.280794
80	1	0	-10.845130	-2.213179	-0.040857
81	7	0	-13.455154	-1.961200	-0.224416
82	1	0	-12.557226	-1.001102	1.362917
83	1	0	-12.874654	0.066002	-0.007848
84	6	0	-14.741145	-1.857237	0.446049
85	6	0	-13.628546	-1.884348	-1.664422
86	1	0	-15.397610	-2.653206	0.099412
87	1	0	-14.608252	-1.967175	1.521618
88	1	0	-15.235673	-0.892924	0.251859
89	1	0	-14.351161	-2.634559	-1.979828
90	1	0	-13.995525	-0.896290	-1.986526
91	1	0	-12.692493	-2.092508	-2.180392
92	1	0	5.214831	-1.747851	2.085442
93	1	0	-5.210246	-1.801465	-2.038917

Ligand	Description	Total Gibbs Free energy (M06-2X/Def2TZVPP)
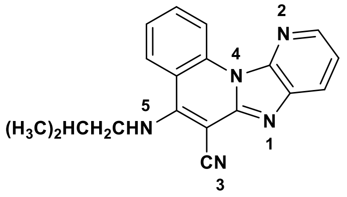 2a	2:1 complex with H^+ bound to N1	-2015.856073 a.u.

CARTESIAN COORDINATES

Center Number	Atomic Number	Atomic Type	Coordinates (Angstroms) X	Y	Z
1	7	0	-3.380165	-1.234990	-0.706479
2	6	0	-4.533547	-0.450284	-0.639766
3	6	0	-2.258002	-0.908141	-0.014871
4	7	0	-1.302703	-1.813522	-0.232017
5	6	0	-1.784405	-2.745152	-1.126263
6	6	0	-3.103850	-2.409484	-1.432415
7	6	0	-2.177577	0.248288	0.788980
8	7	0	-3.901221	-3.070168	-2.238394
9	6	0	-1.219870	-3.860528	-1.718419
10	6	0	-3.357623	-4.148065	-2.803433
11	6	0	-2.045579	-4.567761	-2.577857
12	1	0	-3.998813	-4.705795	-3.472649
13	1	0	-1.681086	-5.450492	-3.082118
14	1	0	-0.197603	-4.153276	-1.524692
15	6	0	-4.511577	0.701165	0.165695
16	6	0	-3.296256	1.078132	0.898104
17	6	0	-0.898220	0.503537	1.360211
18	7	0	0.163007	0.644349	1.777511
19	7	0	-3.321362	2.187552	1.630860
20	6	0	-2.302385	2.709002	2.538785
21	1	0	-4.151489	2.750272	1.570936
22	6	0	-5.677850	-0.814898	-1.350977
23	6	0	-5.690931	1.457178	0.243845
24	6	0	-6.821232	1.102067	-0.454758
25	6	0	-6.809861	-0.036312	-1.257893
26	1	0	-5.750063	2.338596	0.865793
27	1	0	-7.714478	1.704425	-0.374114
28	1	0	-7.695182	-0.319529	-1.809616
29	1	0	-5.657973	-1.702164	-1.962205
30	6	0	-2.875505	3.857512	3.362656
31	1	0	-1.974881	1.909676	3.206422
32	1	0	-1.435380	3.046994	1.969810
33	6	0	-1.755885	4.494397	4.178200
34	1	0	-3.262315	4.608146	2.663761
35	6	0	-4.008466	3.387557	4.270824
36	1	0	-2.137305	5.327887	4.766021
37	1	0	-1.326788	3.766760	4.869718
38	1	0	-0.956002	4.866993	3.538723
39	1	0	-4.417846	4.224236	4.835133
40	1	0	-4.831351	2.927739	3.720859
41	1	0	-3.636113	2.650812	4.985394
42	1	0	-0.269159	-1.737441	0.034068
43	7	0	1.361379	-1.845073	0.302422
44	6	0	1.920119	-2.716621	1.212068
45	6	0	3.238798	-2.337176	1.503708
46	7	0	3.463985	-1.189634	0.735963
47	6	0	2.299891	-0.952937	0.049498
48	6	0	2.202113	0.165061	-0.832889
49	6	0	4.575114	-0.355942	0.635101
50	6	0	3.294977	1.002560	-1.021252

51	6	0	4.508601	0.748398	-0.232223
52	6	0	1.416377	-3.841944	1.847657
53	7	0	4.068135	-2.945038	2.323187
54	6	0	2.271185	-4.498041	2.718727
55	6	0	3.568440	-4.024601	2.923220
56	6	0	0.916677	0.343304	-1.415411
57	7	0	-0.156471	0.430712	-1.820853
58	6	0	5.630511	1.587142	-0.300043
59	6	0	5.725035	-0.615005	1.383841
60	6	0	6.763657	1.338065	0.440121
61	6	0	6.808877	0.228582	1.280823
62	1	0	5.634241	2.466516	-0.927954
63	1	0	7.610759	2.004788	0.368055
64	1	0	7.696602	0.025393	1.863335
65	1	0	5.743387	-1.473967	2.034776
66	1	0	4.234015	-4.540144	3.602854
67	1	0	1.944139	-5.380958	3.248250
68	1	0	0.405944	-4.187722	1.674195
69	7	0	3.310271	2.029101	-1.878020
70	1	0	4.174598	2.532474	-1.961223
71	6	0	2.305239	2.377598	-2.874129
72	6	0	2.824108	3.484247	-3.786093
73	1	0	2.060141	1.490811	-3.462703
74	1	0	1.389353	2.712683	-2.382792
75	6	0	1.827533	3.709974	-4.917504
76	6	0	3.067363	4.779751	-3.016164
77	1	0	3.769571	3.142318	-4.221065
78	1	0	2.182838	4.487496	-5.592231
79	1	0	1.669220	2.801870	-5.498805
80	1	0	0.863109	4.029544	-4.517667
81	1	0	3.448572	5.553611	-3.681197
82	1	0	2.132188	5.139582	-2.582277
83	1	0	3.785203	4.665091	-2.202298

Ligand	Description	Total Gibbs Free energy (M06-2X/Def2TZVPP)
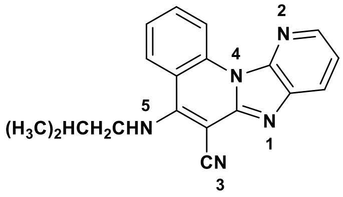<br>2a	2:1 complex with Ca^{2+} bound to N1	-2692.734575 a.u.

CARTESIAN COORDINATES

Center Number	Atomic Number	Atomic Type	Coordinates (Angstroms) X	Y	Z
1	7	0	-3.741272	-1.330916	-1.095615
2	6	0	-5.143323	-1.360531	-1.084820
3	6	0	-3.033563	-0.293725	-0.579352
4	7	0	-1.711788	-0.444538	-0.694727
5	6	0	-1.550018	-1.675120	-1.323431
6	6	0	-2.797930	-2.248937	-1.587032
7	6	0	-3.709302	0.812237	0.021114
8	7	0	-3.022444	-3.404710	-2.167606
9	6	0	-0.417922	-2.383626	-1.698967
10	6	0	-1.928715	-4.072268	-2.528973
11	6	0	-0.628847	-3.605608	-2.315388
12	1	0	-2.091805	-5.026695	-3.011253
13	1	0	0.206380	-4.210301	-2.636583
14	1	0	0.580993	-2.006720	-1.516372
15	6	0	-5.838753	-0.279527	-0.510090
16	6	0	-5.111175	0.851756	0.070614
17	6	0	-2.785297	1.752036	0.509735
18	7	0	-1.841094	2.346563	0.813476
19	7	0	-5.780281	1.853569	0.613267
20	6	0	-5.221212	3.056101	1.221539
21	1	0	-6.785520	1.803512	0.603240
22	6	0	-5.827379	-2.441661	-1.637215
23	6	0	-7.240185	-0.329483	-0.513111
24	6	0	-7.918312	-1.395262	-1.057931
25	6	0	-7.206398	-2.452072	-1.620487
26	1	0	-7.829647	0.470517	-0.088421
27	1	0	-8.998474	-1.408185	-1.048289
28	1	0	-7.734415	-3.291391	-2.050947
29	1	0	-5.269384	-3.255076	-2.071577
30	6	0	-6.331057	3.968872	1.735233
31	1	0	-4.572428	2.770080	2.055656
32	1	0	-4.618044	3.583489	0.477788
33	6	0	-5.715259	5.276522	2.220487
34	1	0	-6.992493	4.189561	0.890809
35	6	0	-7.135907	3.300845	2.846727
36	1	0	-6.491644	5.953268	2.572320
37	1	0	-5.032746	5.093301	3.052737
38	1	0	-5.163037	5.780759	1.427628
39	1	0	-7.929102	3.963602	3.188235
40	1	0	-7.608219	2.366602	2.536831
41	1	0	-6.492660	3.080569	3.700927
42	20	0	0.014368	1.122069	-0.088018
43	7	0	1.696765	-0.448400	0.623189
44	6	0	1.501245	-1.636192	1.321002
45	6	0	2.732944	-2.228129	1.618570
46	7	0	3.701113	-1.366067	1.076774
47	6	0	3.022179	-0.340806	0.501401
48	6	0	3.728072	0.707480	-0.164414
49	6	0	5.101881	-1.433837	1.071698
50	6	0	5.130249	0.701658	-0.220032

51	6	0	5.826704	-0.408393	0.434960
52	6	0	0.350231	-2.290989	1.734610
53	7	0	2.925836	-3.354767	2.264152
54	6	0	0.527690	-3.481334	2.419659
55	6	0	1.814300	-3.970578	2.661228
56	6	0	2.830366	1.637766	-0.716201
57	7	0	1.903065	2.236830	-1.060430
58	6	0	7.226543	-0.489308	0.454795
59	6	0	5.756197	-2.496063	1.692555
60	6	0	7.875394	-1.536041	1.067946
61	6	0	7.134623	-2.540904	1.685706
62	1	0	7.837589	0.272869	-0.007329
63	1	0	8.955015	-1.573614	1.069919
64	1	0	7.639520	-3.365410	2.169259
65	1	0	5.176184	-3.267629	2.172089
66	1	0	1.951129	-4.900243	3.197079
67	1	0	-0.323747	-4.043821	2.773351
68	1	0	-0.637971	-1.897927	1.529472
69	7	0	5.826444	1.643762	-0.831293
70	1	0	6.828687	1.550906	-0.841957
71	6	0	5.300707	2.814593	-1.525288
72	6	0	6.437019	3.684104	-2.055782
73	1	0	4.664843	2.484921	-2.351303
74	1	0	4.691027	3.400089	-0.829776
75	6	0	5.850772	4.806024	-2.905549
76	6	0	7.286971	4.247659	-0.920399
77	1	0	7.061322	3.053529	-2.697776
78	1	0	6.645856	5.428146	-3.312203
79	1	0	5.267115	4.417125	-3.739787
80	1	0	5.204459	5.446342	-2.301971
81	1	0	8.099710	4.850922	-1.321134
82	1	0	6.681654	4.887562	-0.275385
83	1	0	7.736877	3.475102	-0.293942

Ligand	Description	Total Gibbs Free energy (M06-2X/Def2TZVPP)
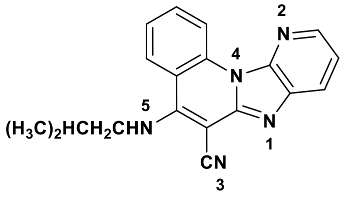 **2a**	2:1 complex with Cu^{2+} bound to N1	-3655.474179 a.u.

CARTESIAN COORDINATES

Center Number	Atomic Number	Atomic Type	Coordinates (Angstroms) X	Y	Z
1	7	0	-3.848874	-1.751363	0.596140
2	6	0	-5.174938	-1.618193	0.172552
3	6	0	-2.924047	-0.768408	0.412826
4	7	0	-1.726712	-1.094837	0.889136
5	6	0	-1.870608	-2.364564	1.418545
6	6	0	-3.188554	-2.802522	1.250814
7	6	0	-3.279267	0.451105	-0.237685
8	7	0	-3.684510	-3.956574	1.634651
9	6	0	-0.977326	-3.213394	2.052633
10	6	0	-2.816814	-4.763323	2.242018
11	6	0	-1.477106	-4.435539	2.468081
12	1	0	-3.205033	-5.718903	2.568249
13	1	0	-0.840410	-5.147616	2.972116
14	1	0	0.055038	-2.932098	2.211153
15	6	0	-5.557603	-0.431589	-0.478816
16	6	0	-4.582779	0.640042	-0.709988
17	6	0	-2.199196	1.362045	-0.316792
18	7	0	-1.215332	1.965942	-0.277143
19	7	0	-4.976546	1.733951	-1.350224
20	6	0	-4.175610	2.898290	-1.707995
21	1	0	-5.930153	1.761063	-1.667332
22	6	0	-6.092601	-2.644486	0.396639
23	6	0	-6.895343	-0.315725	-0.885462
24	6	0	-7.803399	-1.325107	-0.665046
25	6	0	-7.396371	-2.492676	-0.023263
26	1	0	-7.256270	0.574552	-1.380230
27	1	0	-8.827555	-1.207978	-0.988176
28	1	0	-8.105642	-3.289321	0.152250
29	1	0	-5.768126	-3.542300	0.896923
30	6	0	-5.026125	3.922189	-2.453855
31	1	0	-3.770746	3.355708	-0.800905
32	1	0	-3.337415	2.580442	-2.332340
33	6	0	-4.128357	5.047663	-2.955228
34	1	0	-5.465267	3.418108	-3.321384
35	6	0	-6.144043	4.472487	-1.572434
36	1	0	-4.710916	5.782095	-3.508602
37	1	0	-3.656288	5.562416	-2.115859
38	1	0	-3.343811	4.673578	-3.612861
39	1	0	-6.746675	5.189491	-2.127546
40	1	0	-6.819717	3.699618	-1.201912
41	1	0	-5.723715	4.987245	-0.706124
42	29	0	-0.073931	0.001887	0.741768
43	7	0	1.673521	1.023815	0.908148
44	6	0	1.761126	2.274168	1.381240
45	6	0	3.100444	2.756619	1.262656
46	7	0	3.814919	1.724617	0.685744
47	6	0	2.925763	0.701266	0.485191
48	6	0	3.313233	-0.500377	-0.092419
49	6	0	5.171566	1.618790	0.333753
50	6	0	4.677614	-0.680128	-0.493943

51	6	0	5.613512	0.419044	-0.254014
52	6	0	0.794246	3.126151	1.951159
53	7	0	3.535761	3.937219	1.636354
54	6	0	1.241944	4.356317	2.343266
55	6	0	2.603686	4.720398	2.169914
56	6	0	2.282037	-1.471151	-0.231838
57	7	0	1.377846	-2.178311	-0.293810
58	6	0	6.970505	0.323117	-0.597768
59	6	0	6.047530	2.674820	0.563577
60	6	0	7.841136	1.364092	-0.371478
61	6	0	7.375326	2.540786	0.209552
62	1	0	7.377012	-0.572011	-1.047482
63	1	0	8.881687	1.264657	-0.644124
64	1	0	8.055713	3.361426	0.389271
65	1	0	5.684194	3.583665	1.014167
66	1	0	2.936327	5.700454	2.487694
67	1	0	0.571089	5.074539	2.791434
68	1	0	-0.234673	2.812837	2.053731
69	7	0	5.099160	-1.783304	-1.061628
70	1	0	6.078477	-1.810116	-1.308536
71	6	0	4.379547	-3.017647	-1.416123
72	6	0	5.330475	-3.988062	-2.110015
73	1	0	3.973397	-3.460018	-0.506115
74	1	0	3.551446	-2.766105	-2.081412
75	6	0	4.604808	-5.311561	-2.328638
76	6	0	5.843589	-3.421237	-3.431066
77	1	0	6.175461	-4.164629	-1.435275
78	1	0	5.268134	-6.029814	-2.806460
79	1	0	4.260940	-5.742740	-1.388898
80	1	0	3.740308	-5.171827	-2.980149
81	1	0	6.530216	-4.123218	-3.900517
82	1	0	5.013651	-3.252514	-4.119703
83	1	0	6.380674	-2.475690	-3.321212

Ligand	Description	Total Gibbs Free energy (M06-2X/Def2TZVPP)
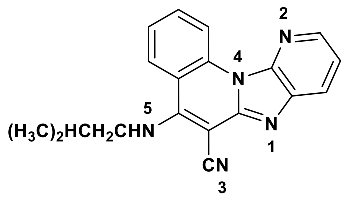<br>**2a**	2:1 complex with Mg^{2+} bound to N1	-2215.177879 a.u.

CARTESIAN COORDINATES

Center Number	Atomic Number	Atomic Type	Coordinates (Angstroms) X	Y	Z
1	7	0	-3.706987	-1.174893	-1.344452
2	6	0	-5.098426	-1.005487	-1.280438
3	6	0	-2.854401	-0.383258	-0.652736
4	7	0	-1.569983	-0.695803	-0.848134
5	6	0	-1.590270	-1.772797	-1.729015
6	6	0	-2.910995	-2.092128	-2.055758
7	6	0	-3.349927	0.662346	0.184097
8	7	0	-3.308617	-3.050646	-2.858571
9	6	0	-0.576942	-2.530057	-2.295298
10	6	0	-2.328055	-3.771224	-3.398698
11	6	0	-0.971344	-3.547769	-3.147077
12	1	0	-2.633862	-4.566603	-4.064907
13	1	0	-0.236946	-4.178905	-3.625271
14	1	0	0.467925	-2.340490	-2.084981
15	6	0	-5.620172	0.022747	-0.470712
16	6	0	-4.728377	0.898138	0.291716
17	6	0	-2.269024	1.319235	0.793560
18	7	0	-1.199221	1.623681	1.114124
19	7	0	-5.224511	1.862689	1.044586
20	6	0	-4.462440	2.815098	1.844622
21	1	0	-6.224637	1.976967	1.066119
22	6	0	-5.940921	-1.842467	-2.008680
23	6	0	-7.013524	0.172092	-0.421406
24	6	0	-7.847834	-0.653135	-1.139297
25	6	0	-7.306255	-1.660992	-1.934101
26	1	0	-7.471472	0.941108	0.184294
27	1	0	-8.918102	-0.517055	-1.084453
28	1	0	-7.957817	-2.311233	-2.500862
29	1	0	-5.514383	-2.620154	-2.621312
30	6	0	-5.392419	3.775139	2.581119
31	1	0	-3.856890	2.267151	2.574702
32	1	0	-3.792187	3.375049	1.186109
33	6	0	-4.555921	4.843849	3.275955
34	1	0	-6.022730	4.262454	1.829911
35	6	0	-6.277048	3.038148	3.582822
36	1	0	-5.200707	5.552763	3.791885
37	1	0	-3.897284	4.392013	4.020327
38	1	0	-3.943162	5.400363	2.566989
39	1	0	-6.942342	3.738571	4.084584
40	1	0	-6.903891	2.271603	3.123216
41	1	0	-5.664401	2.553502	4.345509
42	12	0	0.008320	0.363073	-0.059677
43	7	0	1.552163	-0.669648	0.827773
44	6	0	1.539142	-1.666739	1.798223
45	6	0	2.849429	-2.000215	2.151864
46	7	0	3.673546	-1.174515	1.364380
47	6	0	2.845670	-0.417663	0.606807
48	6	0	3.373376	0.531705	-0.320325
49	6	0	5.069614	-1.055586	1.287975
50	6	0	4.758684	0.707462	-0.451983

51	6	0	5.622840	-0.120285	0.391078
52	6	0	0.502776	-2.338907	2.426784
53	7	0	3.217181	-2.898962	3.034120
54	6	0	0.865516	-3.292344	3.362947
55	6	0	2.214731	-3.537949	3.633486
56	6	0	2.313564	1.162830	-0.990722
57	7	0	1.253939	1.470645	-1.340586
58	6	0	7.019914	-0.012761	0.338435
59	6	0	5.886024	-1.849179	2.090798
60	6	0	7.828398	-0.794458	1.130710
61	6	0	7.256185	-1.714285	2.006751
62	1	0	7.501086	0.690496	-0.326342
63	1	0	8.902176	-0.691979	1.071715
64	1	0	7.887496	-2.330758	2.631263
65	1	0	5.435630	-2.556745	2.767967
66	1	0	2.495828	-4.283122	4.365431
67	1	0	0.111815	-3.855532	3.893152
68	1	0	-0.535742	-2.133822	2.200254
69	7	0	5.284581	1.576390	-1.295628
70	1	0	6.288379	1.638305	-1.344086
71	6	0	4.552518	2.463538	-2.192762
72	6	0	5.510484	3.342741	-2.991730
73	1	0	3.945256	1.859111	-2.872854
74	1	0	3.885343	3.099830	-1.601348
75	6	0	4.715210	4.137147	-4.021808
76	6	0	6.305887	4.273677	-2.080788
77	1	0	6.199965	2.678362	-3.523176
78	1	0	5.381834	4.756868	-4.618508
79	1	0	4.166598	3.483317	-4.699604
80	1	0	4.000964	4.798827	-3.527792
81	1	0	6.992752	4.879689	-2.668956
82	1	0	5.633607	4.950048	-1.549361
83	1	0	6.901425	3.743320	-1.335274

Ligand	Description	Total Gibbs Free energy (M06-2X/Def2TZVPP)
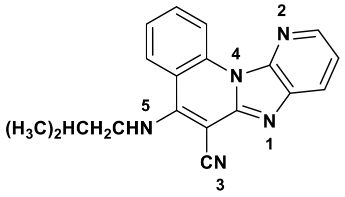 2a	2:1 complex with Zn^{2+} bound to N1	-3794.434344 a.u.

CARTESIAN COORDINATES

Center Number	Atomic Number	Atomic Type	Coordinates (Angstroms) X	Y	Z
1	7	0	-3.784740	-1.173721	-1.287218
2	6	0	-5.159633	-0.905043	-1.204381
3	6	0	-2.872036	-0.457849	-0.590618
4	7	0	-1.619356	-0.868158	-0.810276
5	6	0	-1.721899	-1.921818	-1.709936
6	6	0	-3.066162	-2.134458	-2.024650
7	6	0	-3.276931	0.607331	0.266966
8	7	0	-3.541173	-3.045879	-2.839695
9	6	0	-0.771922	-2.737886	-2.301734
10	6	0	-2.622212	-3.826164	-3.404794
11	6	0	-1.249836	-3.707578	-3.166705
12	1	0	-2.993139	-4.584512	-4.080995
13	1	0	-0.569117	-4.381345	-3.665684
14	1	0	0.286011	-2.626789	-2.100997
15	6	0	-5.595544	0.145392	-0.373250
16	6	0	-4.633781	0.941179	0.391634
17	6	0	-2.148988	1.178737	0.882246
18	7	0	-1.061743	1.408587	1.207910
19	7	0	-5.051948	1.927700	1.163430
20	6	0	-4.218329	2.812438	1.970341
21	1	0	-6.041073	2.112626	1.196054
22	6	0	-6.068444	-1.667650	-1.934665
23	6	0	-6.973817	0.394340	-0.304600
24	6	0	-7.873726	-0.357051	-1.024301
25	6	0	-7.415966	-1.389092	-1.840542
26	1	0	-7.368247	1.184660	0.318092
27	1	0	-8.930617	-0.144821	-0.953972
28	1	0	-8.119484	-1.981710	-2.408356
29	1	0	-5.706518	-2.464609	-2.563773
30	6	0	-5.072909	3.826408	2.725735
31	1	0	-3.648861	2.213293	2.688877
32	1	0	-3.514162	3.331980	1.314452
33	6	0	-4.157316	4.821671	3.429895
34	1	0	-5.670402	4.368652	1.985399
35	6	0	-6.002904	3.141388	3.723206
36	1	0	-4.745966	5.568842	3.958801
37	1	0	-3.530240	4.312531	4.164429
38	1	0	-3.508510	5.341178	2.724917
39	1	0	-6.610876	3.881355	4.240704
40	1	0	-6.688270	2.430664	3.257373
41	1	0	-5.422698	2.600892	4.473641
42	30	0	0.004689	0.031316	-0.043901
43	7	0	1.599845	-0.838474	0.812091
44	6	0	1.669629	-1.803787	1.808764
45	6	0	3.006822	-2.028724	2.144428
46	7	0	3.754957	-1.167183	1.319157
47	6	0	2.864774	-0.491776	0.556134
48	6	0	3.302735	0.470304	-0.401227
49	6	0	5.137702	-0.951982	1.213373
50	6	0	4.669779	0.742455	-0.559269

51	6	0	5.606109	-0.002045	0.284739
52	6	0	0.694645	-2.528414	2.474375
53	7	0	3.453377	-2.872562	3.043952
54	6	0	1.142306	-3.425361	3.429340
55	6	0	2.510482	-3.564720	3.680013
56	6	0	2.193330	1.011302	-1.074864
57	7	0	1.113740	1.240476	-1.425499
58	6	0	6.991036	0.201380	0.201441
59	6	0	6.022284	-1.666906	2.017853
60	6	0	7.867032	-0.502478	0.994928
61	6	0	7.377647	-1.438453	1.903330
62	1	0	7.409594	0.919399	-0.489353
63	1	0	8.929678	-0.326892	0.911468
64	1	0	8.062270	-1.993998	2.528787
65	1	0	5.635876	-2.387899	2.719804
66	1	0	2.857630	-4.266020	4.426743
67	1	0	0.440953	-4.025684	3.989725
68	1	0	-0.359393	-2.403101	2.261871
69	7	0	5.119113	1.628861	-1.428764
70	1	0	6.115129	1.760646	-1.494073
71	6	0	4.314891	2.446394	-2.330871
72	6	0	5.199362	3.379947	-3.152650
73	1	0	3.746256	1.789692	-2.995414
74	1	0	3.609554	3.041761	-1.741523
75	6	0	4.338887	4.099956	-4.184976
76	6	0	5.935204	4.378879	-2.263924
77	1	0	5.929388	2.758942	-3.682439
78	1	0	4.953229	4.757268	-4.797377
79	1	0	3.832207	3.398545	-4.847652
80	1	0	3.583602	4.715461	-3.692362
81	1	0	6.570209	5.024238	-2.868319
82	1	0	5.221607	5.012601	-1.733796
83	1	0	6.575395	3.903520	-1.518350

Ligand	Description	Total Gibbs Free energy (M06-2X/Def2TZVPP)
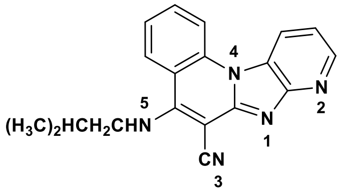 2b	2:1 complex with H^+ bound to N1	-2015.843624 a.u.

CARTESIAN COORDINATES

Center Number	Atomic Number	Atomic Type	Coordinates (Angstroms) X	Y	Z
1	7	0	-3.408433	1.184100	0.845324
2	6	0	-4.582857	0.442331	0.763151
3	6	0	-2.292444	0.844706	0.110947
4	7	0	-1.298157	1.682229	0.275590
5	6	0	-1.741416	2.617477	1.177950
6	6	0	-3.068305	2.336947	1.563358
7	6	0	-2.274398	-0.339815	-0.686403
8	6	0	-3.677077	3.159077	2.498921
9	7	0	-1.010217	3.621434	1.632819
10	6	0	-2.914781	4.217029	2.970919
11	6	0	-1.609531	4.408226	2.516910
12	1	0	-3.329870	4.895697	3.701207
13	1	0	-1.023961	5.236689	2.895904
14	6	0	-4.601707	-0.717540	-0.035154
15	6	0	-3.398033	-1.147095	-0.760912
16	6	0	-1.007135	-0.631106	-1.267046
17	7	0	0.049709	-0.803284	-1.686583
18	7	0	-3.461819	-2.285502	-1.461688
19	6	0	-2.472472	-2.835912	-2.378227
20	1	0	-4.277785	-2.855272	-1.331142
21	6	0	-5.737262	0.848302	1.433593
22	6	0	-5.809690	-1.421959	-0.134393
23	6	0	-6.942459	-1.024592	0.538679
24	6	0	-6.901101	0.119215	1.329173
25	1	0	-5.891446	-2.293377	-0.767650
26	1	0	-7.857366	-1.590148	0.439118
27	1	0	-7.785590	0.451195	1.854491
28	1	0	-5.729878	1.746480	2.022461
29	6	0	-3.081222	-3.979274	-3.183954
30	1	0	-2.140440	-2.049417	-3.059420
31	1	0	-1.598665	-3.187813	-1.826694
32	6	0	-1.989345	-4.649511	-4.010052
33	1	0	-3.474736	-4.715095	-2.472954
34	6	0	-4.217455	-3.493451	-4.079235
35	1	0	-2.397597	-5.479243	-4.585370
36	1	0	-1.553873	-3.937457	-4.713910
37	1	0	-1.188657	-5.034528	-3.378759
38	1	0	-4.656392	-4.325924	-4.627702
39	1	0	-5.016781	-3.006914	-3.518520
40	1	0	-3.839414	-2.773231	-4.807666
41	1	0	0.261184	1.669951	-0.163155
42	7	0	1.282811	1.713559	-0.507847
43	6	0	1.737145	2.613833	-1.448127
44	6	0	3.072243	2.303607	-1.741581
45	7	0	3.370663	1.167022	-0.971136
46	6	0	2.263627	0.863830	-0.232097
47	6	0	2.253715	-0.188806	0.711556
48	6	0	4.499162	0.348032	-0.933425
49	6	0	3.423549	-0.917760	0.926621
50	6	0	4.551754	-0.685776	0.017104
51	7	0	1.037433	3.604329	-1.954903

52	6	0	3.765433	3.142017	-2.600961
53	6	0	1.699384	4.378172	-2.807506
54	6	0	3.041911	4.194904	-3.139921
55	6	0	1.014911	-0.433405	1.370533
56	7	0	-0.009603	-0.612166	1.858075
57	6	0	5.688961	-1.506278	0.026651
58	6	0	5.541083	0.524204	-1.843341
59	6	0	6.727470	-1.314363	-0.854785
60	6	0	6.646982	-0.294960	-1.799276
61	1	0	5.767254	-2.337355	0.712640
62	1	0	7.588154	-1.966428	-0.825666
63	1	0	7.444143	-0.151653	-2.514962
64	1	0	5.469513	1.275624	-2.608274
65	1	0	3.521297	4.884719	-3.818382
66	1	0	1.140295	5.197053	-3.241493
67	7	0	3.570499	-1.818921	1.894974
68	1	0	4.473688	-2.251950	1.972243
69	6	0	2.665705	-2.113934	3.004707
70	6	0	3.356998	-3.018344	4.019288
71	1	0	2.367027	-1.176254	3.476342
72	1	0	1.763805	-2.602920	2.632949
73	6	0	2.458713	-3.172181	5.241709
74	6	0	3.691168	-4.382776	3.421506
75	1	0	4.284058	-2.524774	4.330819
76	1	0	2.935973	-3.803001	5.990087
77	1	0	2.237797	-2.208593	5.700112
78	1	0	1.512800	-3.640606	4.963196
79	1	0	4.190168	-5.009929	4.158948
80	1	0	2.776496	-4.891238	3.110515
81	1	0	4.346549	-4.322502	2.550365
82	1	0	-4.674411	3.017340	2.879122
83	1	0	4.809119	3.032841	-2.842471

Ligand	Description	Total Gibbs Free energy (M06-2X/Def2TZVPP)
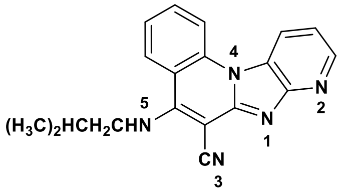 **2b**	2:1 complex with Ca^{2+} bound to N1	-2692.747485 a.u.

CARTESIAN COORDINATES

Center Number	Atomic Number	Atomic Type	Coordinates (Angstroms) X	Y	Z
1	7	0	-4.192167	-1.177834	-1.400591
2	6	0	-5.576925	-0.988170	-1.310423
3	6	0	-3.337924	-0.403557	-0.672481
4	7	0	-2.066439	-0.706795	-0.865685
5	6	0	-2.073926	-1.739904	-1.773241
6	6	0	-3.389083	-2.069028	-2.137919
7	6	0	-3.804466	0.624935	0.195081
8	6	0	-3.574254	-3.093812	-3.054047
9	7	0	-0.969358	-2.311960	-2.215964
10	6	0	-2.420372	-3.704140	-3.526363
11	6	0	-1.158664	-3.294493	-3.092921
12	1	0	-2.496393	-4.509027	-4.242111
13	1	0	-0.270801	-3.781970	-3.472981
14	6	0	-6.078280	0.021563	-0.461921
15	6	0	-5.172922	0.871669	0.321814
16	6	0	-2.697611	1.257706	0.803938
17	7	0	-1.634383	1.567436	1.130834
18	7	0	-5.662883	1.818986	1.104233
19	6	0	-4.890216	2.737095	1.933704
20	1	0	-6.660505	1.946736	1.127562
21	6	0	-6.450579	-1.785194	-2.044744
22	6	0	-7.469000	0.181678	-0.389291
23	6	0	-8.328778	-0.607824	-1.117703
24	6	0	-7.812821	-1.596201	-1.950317
25	1	0	-7.908090	0.934706	0.249103
26	1	0	-9.395868	-0.459724	-1.040188
27	1	0	-8.478025	-2.223222	-2.527105
28	1	0	-6.067827	-2.555399	-2.689496
29	6	0	-5.811636	3.664153	2.720593
30	1	0	-4.275331	2.158932	2.631076
31	1	0	-4.225985	3.323360	1.292784
32	6	0	-4.968249	4.705812	3.447392
33	1	0	-6.453431	4.180789	1.999034
34	6	0	-6.681668	2.885677	3.703522
35	1	0	-5.606786	5.390945	4.001946
36	1	0	-4.297509	4.224874	4.162135
37	1	0	-4.366685	5.292501	2.753365
38	1	0	-7.342235	3.563046	4.241988
39	1	0	-7.311233	2.135835	3.220857
40	1	0	-6.057443	2.372403	4.437536
41	20	0	0.001579	0.124491	-0.023083
42	7	0	2.057870	-0.657624	0.892997
43	6	0	2.050417	-1.593488	1.900538
44	6	0	3.361300	-1.922889	2.280021
45	7	0	4.177596	-1.134071	1.446735
46	6	0	3.334219	-0.413602	0.653605
47	6	0	3.816144	0.498676	-0.328029
48	6	0	5.565482	-0.990480	1.324969
49	6	0	5.189058	0.681668	-0.504940
50	6	0	6.081929	-0.095294	0.364313
51	7	0	0.937265	-2.087566	2.410500

52	6	0	3.531879	-2.859694	3.288508
53	6	0	1.112464	-2.984374	3.377631
54	6	0	2.368757	-3.386803	3.832983
55	6	0	2.718523	1.086659	-0.995607
56	7	0	1.659711	1.385909	-1.345736
57	6	0	7.475032	0.030267	0.271956
58	6	0	6.427389	-1.717295	2.141425
59	6	0	8.323189	-0.690740	1.080573
60	6	0	7.792583	-1.569658	2.019901
61	1	0	7.925203	0.705247	-0.441612
62	1	0	9.392470	-0.571614	0.985383
63	1	0	8.448557	-2.140978	2.661482
64	1	0	6.032978	-2.397535	2.874328
65	1	0	2.433327	-4.120814	4.622267
66	1	0	0.217264	-3.406410	3.814578
67	7	0	5.693570	1.510312	-1.404104
68	1	0	6.694548	1.575250	-1.480951
69	6	0	4.934361	2.341459	-2.331804
70	6	0	5.867948	3.188398	-3.191288
71	1	0	4.320584	1.696979	-2.967099
72	1	0	4.269317	2.998722	-1.762161
73	6	0	5.046633	3.919007	-4.247802
74	6	0	6.666664	4.175244	-2.344300
75	1	0	6.557454	2.505972	-3.699587
76	1	0	5.695166	4.515580	-4.886698
77	1	0	4.495663	3.224092	-4.881299
78	1	0	4.331253	4.595834	-3.776455
79	1	0	7.335514	4.758997	-2.974387
80	1	0	5.994174	4.869241	-1.836338
81	1	0	7.280798	3.690893	-1.582831
82	1	0	-4.533694	-3.430678	-3.409107
83	1	0	4.486927	-3.191110	3.660143

Ligand	Description	Total Gibbs Free energy (M06-2X/Def2TZVPP)
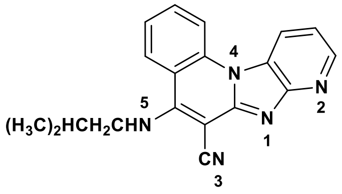 2b	2:1 complex with Cu^{2+} bound to N1	-3655.500239 a.u.

CARTESIAN COORDINATES

Center Number	Atomic Number	Atomic Type	Coordinates (Angstroms) X	Y	Z
1	7	0	-3.795565	-1.636741	0.826692
2	6	0	-5.148176	-1.536987	0.470789
3	6	0	-2.896208	-0.727174	0.369025
4	7	0	-1.664383	-0.970656	0.800077
5	6	0	-1.751623	-2.100929	1.589866
6	6	0	-3.080114	-2.546533	1.631905
7	6	0	-3.272406	0.355079	-0.469359
8	6	0	-3.367152	-3.664024	2.402700
9	7	0	-0.723470	-2.636006	2.212805
10	6	0	-2.291583	-4.243865	3.058880
11	6	0	-1.006882	-3.711337	2.941813
12	1	0	-2.448647	-5.116448	3.675272
13	1	0	-0.179075	-4.171465	3.464810
14	6	0	-5.567363	-0.465708	-0.345985
15	6	0	-4.608213	0.526974	-0.840373
16	6	0	-2.120876	1.105806	-0.791549
17	7	0	-1.025508	1.468024	-0.858146
18	7	0	-5.012492	1.526611	-1.604528
19	6	0	-4.168618	2.578495	-2.162530
20	1	0	-5.988630	1.578179	-1.843586
21	6	0	-6.068974	-2.482772	0.912872
22	6	0	-6.928000	-0.386720	-0.674561
23	6	0	-7.835465	-1.319098	-0.228368
24	6	0	-7.398904	-2.374569	0.566995
25	1	0	-7.302993	0.415566	-1.293540
26	1	0	-8.877796	-1.231902	-0.497813
27	1	0	-8.101328	-3.117412	0.917872
28	1	0	-5.748978	-3.307881	1.522182
29	6	0	-5.002055	3.581153	-2.955050
30	1	0	-3.656732	3.097200	-1.345270
31	1	0	-3.414233	2.123255	-2.810279
32	6	0	-4.068152	4.568689	-3.645763
33	1	0	-5.545500	3.022395	-3.724397
34	6	0	-6.000212	4.310235	-2.059857
35	1	0	-4.640606	5.283601	-4.233817
36	1	0	-3.492168	5.130996	-2.908179
37	1	0	-3.370736	4.063926	-4.314036
38	1	0	-6.593269	5.009221	-2.647029
39	1	0	-6.698049	3.638767	-1.556022
40	1	0	-5.474309	4.880144	-1.291386
41	29	0	-0.000301	-0.016650	0.428991
42	7	0	1.651208	0.957306	0.804339
43	6	0	1.713539	2.124051	1.541684
44	6	0	3.041169	2.568890	1.610773
45	7	0	3.782027	1.621010	0.875551
46	6	0	2.896906	0.691725	0.430000
47	6	0	3.300393	-0.433408	-0.336151
48	6	0	5.145547	1.504955	0.569905
49	6	0	4.649139	-0.628771	-0.643116
50	6	0	5.591171	0.392386	-0.174328
51	7	0	0.665491	2.689315	2.101533

52	6	0	3.304540	3.719278	2.340441
53	6	0	0.926259	3.796871	2.789684
54	6	0	2.207950	4.331193	2.929409
55	6	0	2.160244	-1.203064	-0.654147
56	7	0	1.067561	-1.569856	-0.737646
57	6	0	6.960202	0.303984	-0.463072
58	6	0	6.050599	2.476941	0.986966
59	6	0	7.852028	1.262379	-0.041031
60	6	0	7.390335	2.355650	0.686120
61	1	0	7.353710	-0.524405	-1.034345
62	1	0	8.901386	1.167150	-0.278595
63	1	0	8.080411	3.119110	1.016680
64	1	0	5.710512	3.333493	1.539434
65	1	0	2.345832	5.230447	3.511079
66	1	0	0.081687	4.282468	3.260256
67	7	0	5.079981	-1.673162	-1.328785
68	1	0	6.067936	-1.753673	-1.502361
69	6	0	4.256539	-2.760554	-1.847855
70	6	0	5.110539	-3.777932	-2.598674
71	1	0	3.741495	-3.244908	-1.013643
72	1	0	3.503745	-2.347103	-2.527223
73	6	0	4.240453	-4.968152	-2.987084
74	6	0	5.766352	-3.158034	-3.829482
75	1	0	5.887711	-4.126869	-1.910514
76	1	0	4.834031	-5.717708	-3.507116
77	1	0	3.790757	-5.440352	-2.113822
78	1	0	3.439465	-4.654035	-3.659168
79	1	0	6.381621	-3.896000	-4.341260
80	1	0	5.004855	-2.812165	-4.531156
81	1	0	6.410589	-2.308856	-3.593550
82	1	0	-4.349341	-4.088836	2.520909
83	1	0	4.283850	4.145411	2.476667

Ligand	Description	Total Gibbs Free energy (M06-2X/Def2TZVPP)
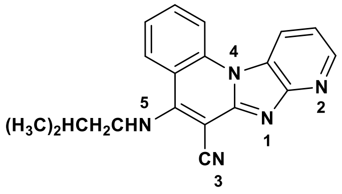<br>**2b**	2:1 complex with Mg^{2+} bound to N1	-2215.182394 a.u.

CARTESIAN COORDINATES

Center Number	Atomic Number	Atomic Type	Coordinates (Angstroms) X	Y	Z
1	7	0	-3.653758	-1.709446	0.830253
2	6	0	-5.050335	-1.616621	0.746934
3	6	0	-2.860026	-0.822837	0.171776
4	7	0	-1.566779	-1.048672	0.354584
5	6	0	-1.498071	-2.150075	1.185558
6	6	0	-2.790089	-2.594652	1.505220
7	6	0	-3.406608	0.223090	-0.627212
8	6	0	-2.915803	-3.701523	2.332372
9	7	0	-0.365225	-2.682040	1.598486
10	6	0	-1.730271	-4.269908	2.772731
11	6	0	-0.496421	-3.740746	2.391505
12	1	0	-1.758696	-5.134343	3.419212
13	1	0	0.419677	-4.196059	2.743913
14	6	0	-5.628046	-0.595075	-0.036385
15	6	0	-4.789883	0.365267	-0.760862
16	6	0	-2.348571	0.970008	-1.182203
17	7	0	-1.290164	1.344869	-1.456501
18	7	0	-5.340530	1.313333	-1.498454
19	6	0	-4.625934	2.322261	-2.273030
20	1	0	-6.344492	1.346686	-1.559269
21	6	0	-5.862733	-2.517988	1.428959
22	6	0	-7.026908	-0.525919	-0.095665
23	6	0	-7.825034	-1.419878	0.579626
24	6	0	-7.235403	-2.421121	1.345333
25	1	0	-7.519946	0.239992	-0.676842
26	1	0	-8.900343	-1.341729	0.514889
27	1	0	-7.851399	-3.129504	1.881157
28	1	0	-5.426187	-3.296139	2.027418
29	6	0	-5.601716	3.259648	-2.978388
30	1	0	-3.986731	2.903754	-1.600020
31	1	0	-3.990400	1.820960	-3.008668
32	6	0	-4.819914	4.194797	-3.894315
33	1	0	-6.262278	2.642172	-3.596191
34	6	0	-6.438924	4.051178	-1.977583
35	1	0	-5.498663	4.860930	-4.423585
36	1	0	-4.132697	4.813672	-3.314073
37	1	0	-4.242719	3.643039	-4.636043
38	1	0	-7.136742	4.702321	-2.501140
39	1	0	-7.027152	3.417708	-1.310903
40	1	0	-5.795694	4.678872	-1.357787
41	12	0	0.005729	0.019187	-0.402729
42	7	0	1.550133	0.827383	0.669411
43	6	0	1.449554	1.658665	1.767848
44	6	0	2.728236	1.988436	2.242319
45	7	0	3.617253	1.315208	1.381202
46	6	0	2.849406	0.650645	0.476580
47	6	0	3.426413	-0.140650	-0.558977
48	6	0	5.016146	1.237530	1.324306
49	6	0	4.813638	-0.255474	-0.675326
50	6	0	5.623373	0.466370	0.310774
51	7	0	0.301340	2.066019	2.270608

52	6	0	2.821760	2.826596	3.344107
53	6	0	0.401684	2.866316	3.327205
54	6	0	1.619872	3.261852	3.881342
55	6	0	2.390091	-0.699872	-1.332593
56	7	0	1.341810	-0.974686	-1.734367
57	6	0	7.023645	0.413122	0.277072
58	6	0	5.802134	1.911174	2.254926
59	6	0	7.795704	1.083090	1.197650
60	6	0	7.177226	1.834717	2.192206
61	1	0	7.538596	-0.160488	-0.480197
62	1	0	8.872875	1.023559	1.146234
63	1	0	7.772376	2.364286	2.922671
64	1	0	5.342633	2.495387	3.030667
65	1	0	1.623459	3.916789	4.739789
66	1	0	-0.527368	3.214773	3.758526
67	7	0	5.392177	-0.974317	-1.621048
68	1	0	6.397462	-1.017876	-1.638787
69	6	0	4.707102	-1.741672	-2.655555
70	6	0	5.709136	-2.421634	-3.583947
71	1	0	4.070278	-2.491921	-2.178076
72	1	0	4.071716	-1.067359	-3.239766
73	6	0	4.956029	-3.332195	-4.547497
74	6	0	6.549788	-1.399856	-4.344434
75	1	0	6.364786	-3.041067	-2.962863
76	1	0	5.653464	-3.844438	-5.207625
77	1	0	4.376099	-4.087866	-4.017904
78	1	0	4.274891	-2.750381	-5.171585
79	1	0	7.265855	-1.906048	-4.989477
80	1	0	5.911742	-0.778150	-4.975474
81	1	0	7.118202	-0.735936	-3.690320
82	1	0	-3.853470	-4.131309	2.640949
83	1	0	3.746833	3.149508	3.790426

Ligand	Description	Total Gibbs Free energy (M06-2X/Def2TZVPP)
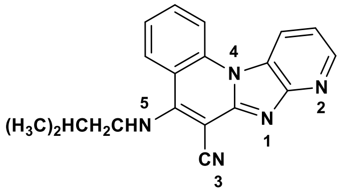 2b	2:1 complex with Zn^{2+} bound to N1	-3794.437833 a.u.

CARTESIAN COORDINATES

Center Number	Atomic Number	Atomic Type	Coordinates (Angstroms) X	Y	Z
1	7	0	-3.728869	-1.500425	-1.072620
2	6	0	-5.114236	-1.283425	-1.045662
3	6	0	-2.874270	-0.574880	-0.561312
4	7	0	-1.604807	-0.943559	-0.666644
5	6	0	-1.614413	-2.179912	-1.280776
6	6	0	-2.935886	-2.562464	-1.552905
7	6	0	-3.337603	0.644564	0.008933
8	6	0	-3.145074	-3.783372	-2.178736
9	7	0	-0.526581	-2.867554	-1.557264
10	6	0	-2.007447	-4.518656	-2.475065
11	6	0	-0.737634	-4.036618	-2.153879
12	1	0	-2.102242	-5.477678	-2.962195
13	1	0	0.140435	-4.622361	-2.391621
14	6	0	-5.612812	-0.085668	-0.492164
15	6	0	-4.705206	0.927823	0.054029
16	6	0	-2.233562	1.403552	0.450708
17	7	0	-1.156247	1.749713	0.688085
18	7	0	-5.184129	2.049581	0.562611
19	6	0	-4.401329	3.141405	1.132606
20	1	0	-6.180412	2.190464	0.548115
21	6	0	-5.991407	-2.236946	-1.554673
22	6	0	-7.002279	0.100235	-0.475790
23	6	0	-7.864715	-0.844173	-0.981994
24	6	0	-7.352239	-2.018957	-1.524064
25	1	0	-7.436899	0.997252	-0.058845
26	1	0	-8.930859	-0.673110	-0.955559
27	1	0	-8.019496	-2.769625	-1.923575
28	1	0	-5.615227	-3.151896	-1.973372
29	6	0	-5.312044	4.255920	1.639388
30	1	0	-3.802265	2.756321	1.964478
31	1	0	-3.723698	3.530144	0.367354
32	6	0	-4.454878	5.436854	2.081346
33	1	0	-5.937467	4.576963	0.799660
34	6	0	-6.204666	3.773631	2.779462
35	1	0	-5.085285	6.253241	2.428738
36	1	0	-3.800735	5.148212	2.906415
37	1	0	-3.835460	5.812226	1.267004
38	1	0	-6.855561	4.578950	3.115394
39	1	0	-6.846162	2.935555	2.500273
40	1	0	-5.596591	3.457726	3.629347
41	30	0	0.003949	0.067511	-0.063922
42	7	0	1.585715	-0.410326	1.051068
43	6	0	1.562459	-1.016278	2.291320
44	6	0	2.873995	-1.198980	2.753000
45	7	0	3.695286	-0.675764	1.733558
46	6	0	2.865216	-0.225566	0.755478
47	6	0	3.361282	0.370858	-0.438270
48	6	0	5.086391	-0.561693	1.596407
49	6	0	4.737074	0.513546	-0.636998
50	6	0	5.617268	0.026455	0.429444
51	7	0	0.456235	-1.356975	2.918077

52	6	0	3.050971	-1.796806	3.992876
53	6	0	0.636296	-1.929219	4.104391
54	6	0	1.893658	-2.161135	4.664125
55	6	0	2.277620	0.730249	-1.266972
56	7	0	1.209629	0.890542	-1.679862
57	6	0	7.011280	0.131597	0.324406
58	6	0	5.937638	-1.020325	2.597772
59	6	0	7.848109	-0.322568	1.317090
60	6	0	7.304102	-0.902432	2.459195
61	1	0	7.469770	0.577470	-0.546419
62	1	0	8.918512	-0.227880	1.208022
63	1	0	7.950897	-1.263568	3.246329
64	1	0	5.536664	-1.469673	3.487288
65	1	0	1.963091	-2.630847	5.633943
66	1	0	-0.257515	-2.219326	4.640640
67	7	0	5.246664	1.059720	-1.727124
68	1	0	6.248196	1.118545	-1.805367
69	6	0	4.493700	1.584239	-2.861885
70	6	0	5.432418	2.165709	-3.915006
71	1	0	3.896973	0.777306	-3.296298
72	1	0	3.814808	2.367876	-2.509502
73	6	0	4.619193	2.559682	-5.143071
74	6	0	6.211506	3.360140	-3.371373
75	1	0	6.133811	1.375423	-4.202830
76	1	0	5.271747	2.958891	-5.917263
77	1	0	4.081968	1.708407	-5.560782
78	1	0	3.892737	3.334146	-4.889303
79	1	0	6.883660	3.750457	-4.133548
80	1	0	5.526969	4.161367	-3.086283
81	1	0	6.820299	3.117146	-2.498372
82	1	0	-4.111443	-4.178795	-2.440726
83	1	0	4.007194	-1.987646	4.449028

Ligand	Description	Total Gibbs Free energy (M06-2X/Def2TZVPP)
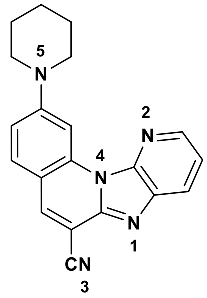 3a	2:1 complex with H^+ bound to N1	-2092.034262 a.u.

CARTESIAN COORDINATES

Center Number	Atomic Number	Atomic Type	Coordinates (Angstroms) X	Y	Z
1	7	0	-3.458427	0.193117	-0.062683
2	6	0	-4.528158	-0.660613	0.227449
3	6	0	-2.191157	-0.039918	0.383710
4	7	0	-1.368415	0.925485	-0.029969
5	6	0	-2.089123	1.802449	-0.806773
6	6	0	-3.416492	1.368464	-0.832382
7	6	0	-1.889880	-1.180822	1.160065
8	7	0	-4.409415	1.959048	-1.461071
9	6	0	-1.740732	2.944970	-1.508712
10	6	0	-4.071443	3.058493	-2.130242
11	6	0	-2.772737	3.573704	-2.182186
12	1	0	-4.872952	3.558906	-2.656967
13	1	0	-2.584908	4.468994	-2.756314
14	1	0	-0.725054	3.313592	-1.531984
15	6	0	-4.228579	-1.795696	1.018414
16	6	0	-2.919560	-2.036909	1.461098
17	1	0	-2.716370	-2.921392	2.050739
18	6	0	-5.802995	-0.396629	-0.226469
19	6	0	-5.304197	-2.658007	1.327626
20	6	0	-6.567167	-2.416816	0.887153
21	6	0	-6.862214	-1.277312	0.075158
22	1	0	-5.104102	-3.536296	1.927539
23	1	0	-7.341193	-3.121591	1.140166
24	7	0	-8.108226	-1.066101	-0.410201
25	1	0	-5.962854	0.498065	-0.797483
26	6	0	-0.541325	-1.408018	1.560202
27	7	0	0.553974	-1.571433	1.863242
28	6	0	-9.307770	-1.612592	0.225889
29	6	0	-9.877227	-0.588081	1.207566
30	1	0	-10.030970	-1.818554	-0.566003
31	1	0	-9.096424	-2.555560	0.715156
32	6	0	-10.164215	0.737413	0.502015
33	1	0	-10.785276	-0.985288	1.662021
34	1	0	-9.150028	-0.435721	2.010226
35	6	0	-8.943898	1.231736	-0.276239
36	1	0	-10.994261	0.595174	-0.196485
37	1	0	-10.485025	1.487468	1.224248
38	6	0	-8.435982	0.123010	-1.194502
39	1	0	-9.194551	2.114441	-0.864975
40	1	0	-8.144219	1.514071	0.414540
41	1	0	-9.225350	-0.167304	-1.891650
42	1	0	-7.585386	0.428724	-1.795258
43	1	0	-0.301820	0.916681	0.055769
44	7	0	1.360740	0.981249	0.084534
45	6	0	2.134664	1.834518	0.836090
46	6	0	3.466797	1.391704	0.860942
47	7	0	3.481647	0.223987	0.097255
48	6	0	2.184592	0.037427	-0.330123

49	6	0	1.876543	-1.105958	-1.127553
50	6	0	4.526604	-0.648672	-0.214224
51	6	0	2.878014	-1.975579	-1.452406
52	6	0	4.202732	-1.766003	-1.015855
53	6	0	1.824262	2.994515	1.535096
54	7	0	4.475793	1.972667	1.479671
55	6	0	2.867401	3.617416	2.195407
56	6	0	4.158435	3.081831	2.140977
57	6	0	0.521705	-1.299888	-1.521227
58	7	0	-0.584289	-1.426152	-1.807534
59	6	0	5.255700	-2.642858	-1.345175
60	6	0	5.813692	-0.417620	0.231104
61	6	0	6.530310	-2.432886	-0.912505
62	6	0	6.851263	-1.311633	-0.091915
63	7	0	8.111515	-1.130384	0.389725
64	1	0	0.816581	3.386897	1.564399
65	1	0	2.698129	4.522350	2.760551
66	1	0	4.970413	3.575678	2.658501
67	1	0	2.655063	-2.847319	-2.053895
68	1	0	5.033815	-3.509881	-1.954176
69	1	0	7.288203	-3.150508	-1.178823
70	1	0	5.993929	0.466905	0.812798
71	6	0	9.288518	-1.647434	-0.307424
72	6	0	9.806262	-0.600145	-1.293873
73	1	0	10.048196	-1.859597	0.448175
74	1	0	9.067101	-2.584271	-0.804787
75	6	0	10.108398	0.715668	-0.576088
76	1	0	10.698608	-0.977783	-1.794331
77	1	0	9.043655	-0.440814	-2.061704
78	6	0	8.915255	1.182299	0.259702
79	1	0	10.967317	0.568944	0.085994
80	1	0	10.392995	1.482359	-1.296342
81	6	0	8.456298	0.050945	1.176477
82	1	0	9.180009	2.057875	0.853207
83	1	0	8.085921	1.467909	-0.393627
84	1	0	9.275743	-0.239173	1.838450
85	1	0	7.625236	0.336826	1.813952

Ligand	Description	Total Gibbs Free energy (M06-2X/Def2TZVPP)
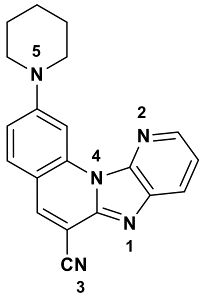 3a	2:1 complex with Ca^{2+} bound to N1	-2768.914719 a.u.

CARTESIAN COORDINATES

Center Number	Atomic Number	Atomic Type	Coordinates (Angstroms) X	Y	Z
1	7	0	-4.362510	0.082959	0.121725
2	6	0	-5.529434	-0.675202	-0.046784
3	6	0	-3.205329	-0.431868	0.634876
4	7	0	-2.215766	0.458452	0.712158
5	6	0	-2.763813	1.638308	0.222262
6	6	0	-4.095961	1.431266	-0.155808
7	6	0	-3.164814	-1.799432	1.029282
8	7	0	-4.918314	2.325530	-0.660325
9	6	0	-2.236284	2.913057	0.066133
10	6	0	-4.396610	3.540292	-0.810408
11	6	0	-3.082956	3.870488	-0.463457
12	1	0	-5.053622	4.293553	-1.224022
13	1	0	-2.739939	4.884022	-0.610556
14	1	0	-1.221170	3.151976	0.353945
15	6	0	-5.464026	-2.043467	0.359582
16	6	0	-4.298962	-2.581821	0.887246
17	1	0	-4.281374	-3.620295	1.191551
18	6	0	-6.668972	-0.121642	-0.564549
19	6	0	-6.650768	-2.808705	0.206198
20	6	0	-7.787028	-2.280157	-0.304843
21	6	0	-7.843187	-0.907728	-0.732285
22	1	0	-6.634863	-3.846835	0.512079
23	1	0	-8.650578	-2.916261	-0.393124
24	7	0	-8.949749	-0.390786	-1.273661
25	1	0	-6.649073	0.919325	-0.825300
26	6	0	-1.915790	-2.172821	1.549775
27	7	0	-0.826221	-2.240042	1.932858
28	6	0	-10.262858	-1.045319	-1.248734
29	6	0	-11.118991	-0.416483	-0.150169
30	1	0	-10.721129	-0.879592	-2.225646
31	1	0	-10.165956	-2.115432	-1.120655
32	6	0	-11.249452	1.090508	-0.364674
33	1	0	-12.099999	-0.891191	-0.146117
34	1	0	-10.652502	-0.622162	0.817157
35	6	0	-9.881416	1.747610	-0.543570
36	1	0	-11.849992	1.271915	-1.260490
37	1	0	-11.782342	1.545228	0.469196
38	6	0	-9.089396	1.024727	-1.630675
39	1	0	-9.990603	2.797572	-0.814636
40	1	0	-9.314614	1.706961	0.390737
41	1	0	-9.637073	1.058324	-2.574756
42	1	0	-8.121538	1.477270	-1.810160
43	20	0	0.000108	0.000540	1.581690
44	7	0	2.215878	-0.457531	0.712052
45	6	0	2.763719	-1.637565	0.222356
46	6	0	4.095874	-1.430790	-0.155818
47	7	0	4.362642	-0.082476	0.121434
48	6	0	3.205560	0.432634	0.634537

49	6	0	3.165242	1.800280	1.028643
50	6	0	5.529659	0.675476	-0.047249
51	6	0	4.299495	2.582476	0.886400
52	6	0	5.464460	2.043839	0.358820
53	6	0	2.236002	-2.912272	0.066480
54	7	0	4.918078	-2.325271	-0.660218
55	6	0	3.082497	-3.869919	-0.462974
56	6	0	4.396196	-3.539974	-0.810057
57	6	0	1.916283	2.173908	1.549125
58	7	0	0.826730	2.241199	1.932247
59	6	0	6.651303	2.808879	0.205235
60	6	0	6.669136	0.121634	-0.564865
61	6	0	7.787507	2.280028	-0.305611
62	6	0	7.843498	0.907470	-0.732623
63	7	0	8.950046	0.390212	-1.273734
64	1	0	1.220875	-3.150992	0.354402
65	1	0	2.739347	-4.883433	-0.609875
66	1	0	5.053067	-4.293415	-1.223560
67	1	0	4.282070	3.621017	1.190474
68	1	0	6.635491	3.847129	0.510704
69	1	0	8.651078	2.916050	-0.394294
70	1	0	6.649124	-0.919406	-0.825322
71	6	0	10.263522	1.043945	-1.247646
72	6	0	11.118605	0.413902	-0.148933
73	1	0	10.722303	0.878619	-2.224385
74	1	0	10.167198	2.114022	-1.118821
75	6	0	11.248213	-1.093063	-0.364101
76	1	0	12.099921	0.887966	-0.144061
77	1	0	10.651696	0.619398	0.818229
78	6	0	9.879857	-1.749202	-0.544111
79	1	0	11.849151	-1.274439	-1.259657
80	1	0	11.780332	-1.548516	0.469861
81	6	0	9.089011	-1.025213	-1.631316
82	1	0	9.988536	-2.799090	-0.815671
83	1	0	9.312534	-1.708682	0.389884
84	1	0	9.637297	-1.058689	-2.575053
85	1	0	8.120953	-1.476971	-1.811736

Ligand	Description	Total Gibbs Free energy (M06-2X/Def2TZVPP)
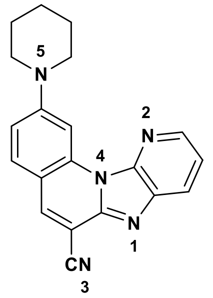 **3a**	2:1 complex with Cu^{2+} bound to N1	-3731.673964 a.u.

CARTESIAN COORDINATES

Center Number	Atomic Number	Atomic Type	X	Y	Z
1	7	0	4.190966	-0.161652	-0.069080
2	6	0	5.337521	0.500528	-0.521494
3	6	0	2.932380	0.373110	-0.162635
4	7	0	1.993691	-0.433158	0.319426
5	6	0	2.660567	-1.561313	0.754035
6	6	0	4.035121	-1.419482	0.525279
7	6	0	2.756895	1.662987	-0.736712
8	7	0	4.972770	-2.296891	0.817037
9	6	0	2.213848	-2.734938	1.345216
10	6	0	4.529411	-3.416006	1.382678
11	6	0	3.181456	-3.670519	1.659545
12	1	0	5.280966	-4.153228	1.632030
13	1	0	2.909215	-4.608361	2.120906
14	1	0	1.164328	-2.906606	1.540087
15	6	0	5.142829	1.788005	-1.090531
16	6	0	3.862377	2.344306	-1.188566
17	1	0	3.745287	3.326925	-1.626851
18	6	0	6.583519	-0.068484	-0.409618
19	6	0	6.300212	2.468275	-1.538372
20	6	0	7.539223	1.923192	-1.439261
21	6	0	7.730624	0.616254	-0.881266
22	1	0	6.182647	3.451759	-1.974755
23	1	0	8.377241	2.489435	-1.807798
24	7	0	8.949836	0.051951	-0.821617
25	1	0	6.656448	-1.032708	0.056733
26	6	0	1.423266	2.144161	-0.790411
27	7	0	0.304471	2.419292	-0.773011
28	6	0	10.200255	0.803846	-0.951981
29	6	0	10.757512	1.104177	0.439988
30	1	0	10.898290	0.176241	-1.509666
31	1	0	10.056108	1.708981	-1.527893
32	6	0	10.956356	-0.186772	1.232500
33	1	0	11.699305	1.644048	0.341154
34	1	0	10.056299	1.762280	0.960611
35	6	0	9.686655	-1.037648	1.239676
36	1	0	11.766242	-0.763195	0.776160
37	1	0	11.267096	0.040625	2.251488
38	6	0	9.192372	-1.246267	-0.190169
39	1	0	9.873641	-2.005210	1.705470
40	1	0	8.900173	-0.542006	1.815752
41	1	0	9.965979	-1.743325	-0.779606
42	1	0	8.307737	-1.870881	-0.240741
43	29	0	0.050532	-0.040881	0.289646
44	7	0	-1.930535	0.292593	0.377059
45	6	0	-2.530525	1.451758	0.817700
46	6	0	-3.913978	1.382488	0.611555
47	7	0	-4.142503	0.139375	0.023760
48	6	0	-2.908248	-0.460781	-0.084929

49	6	0	-2.818881	-1.767176	-0.655530
50	6	0	-5.320226	-0.465257	-0.406452
51	6	0	-3.953063	-2.395929	-1.088019
52	6	0	-5.211801	-1.763414	-0.975870
53	6	0	-2.010938	2.605007	1.403011
54	7	0	-4.808217	2.303554	0.915363
55	6	0	-2.926399	3.581979	1.728937
56	6	0	-4.294842	3.395880	1.472141
57	6	0	-1.520730	-2.353467	-0.737885
58	7	0	-0.450233	-2.767557	-0.770906
59	6	0	-6.401410	-2.390070	-1.422664
60	6	0	-6.533180	0.170656	-0.295297
61	6	0	-7.608994	-1.778221	-1.330986
62	6	0	-7.717702	-0.463801	-0.761553
63	7	0	-8.905138	0.154138	-0.669828
64	1	0	-0.951104	2.723682	1.575988
65	1	0	-2.603365	4.506502	2.184172
66	1	0	-5.000305	4.173449	1.733713
67	1	0	-3.890862	-3.384480	-1.522432
68	1	0	-6.334452	-3.385623	-1.840626
69	1	0	-8.485243	-2.310455	-1.660338
70	1	0	-6.540474	1.162989	0.119619
71	6	0	-10.158392	-0.397061	-1.198833
72	6	0	-11.020345	-0.901761	-0.037716
73	1	0	-10.664237	0.428429	-1.703256
74	1	0	-9.960636	-1.158937	-1.941113
75	6	0	-11.291363	0.234042	0.943385
76	1	0	-11.948479	-1.298739	-0.446948
77	1	0	-10.500365	-1.725328	0.458249
78	6	0	-9.992579	0.904038	1.377934
79	1	0	-11.940023	0.973870	0.467494
80	1	0	-11.826215	-0.141531	1.814544
81	6	0	-9.158549	1.336052	0.163422
82	1	0	-10.190064	1.789489	1.981174
83	1	0	-9.393002	0.221492	1.985073
84	1	0	-9.717397	2.045612	-0.448493
85	1	0	-8.233503	1.804605	0.471446

Ligand	Description	Total Gibbs Free energy (M06-2X/Def2TZVPP)
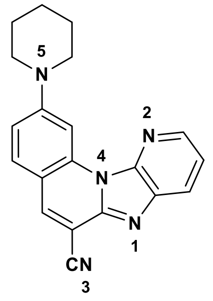 3a	2:1 complex with Mg^{2+} bound to N1	-2291.354344 a.u.

CARTESIAN COORDINATES

Center Number	Atomic Number	Atomic Type	X	Y	Z
1	7	0	-3.990532	-0.046716	0.138501
2	6	0	-5.248367	-0.640656	0.320357
3	6	0	-2.885646	-0.433184	0.835283
4	7	0	-1.790617	0.263141	0.514902
5	6	0	-2.207766	1.164786	-0.455452
6	6	0	-3.573333	0.992483	-0.709554
7	6	0	-2.980814	-1.480382	1.792328
8	7	0	-4.302339	1.671453	-1.567129
9	6	0	-1.531223	2.145765	-1.164832
10	6	0	-3.641159	2.609140	-2.241905
11	6	0	-2.278708	2.874286	-2.073213
12	1	0	-4.220254	3.181937	-2.953618
13	1	0	-1.818978	3.655577	-2.660312
14	1	0	-0.475847	2.335472	-1.018173
15	6	0	-5.322983	-1.687615	1.292071
16	6	0	-4.205646	-2.090541	2.008430
17	1	0	-4.295791	-2.883760	2.739018
18	6	0	-6.339041	-0.226074	-0.393258
19	6	0	-6.600349	-2.278793	1.488161
20	6	0	-7.690755	-1.884356	0.791324
21	6	0	-7.607221	-0.844408	-0.200177
22	1	0	-6.692674	-3.067591	2.223526
23	1	0	-8.628393	-2.372023	0.993937
24	7	0	-8.672265	-0.477470	-0.915862
25	1	0	-6.207818	0.579341	-1.090509
26	6	0	-1.732204	-1.710948	2.392846
27	7	0	-0.612691	-1.649774	2.678116
28	6	0	-10.046667	-0.906951	-0.630206
29	6	0	-10.790159	0.220506	0.084039
30	1	0	-10.519080	-1.120303	-1.591004
31	1	0	-10.059984	-1.826132	-0.059424
32	6	0	-10.769375	1.494853	-0.758111
33	1	0	-11.814606	-0.095505	0.279599
34	1	0	-10.312391	0.397372	1.051518
35	6	0	-9.347353	1.855987	-1.184869
36	1	0	-11.380159	1.340662	-1.652044
37	1	0	-11.220074	2.318858	-0.206871
38	6	0	-8.673422	0.656032	-1.847168
39	1	0	-9.355187	2.693666	-1.882028
40	1	0	-8.754642	2.157198	-0.316698
41	1	0	-9.245158	0.343290	-2.723279
42	1	0	-7.670062	0.881501	-2.187342
43	12	0	0.000078	-0.000184	1.508437
44	7	0	1.790625	-0.263152	0.514519
45	6	0	2.207781	-1.164595	-0.456020
46	6	0	3.573346	-0.992283	-0.710049
47	7	0	3.990561	0.046724	0.138238
48	6	0	2.885680	0.433072	0.835072

49	6	0	2.980857	1.480079	1.792338
50	6	0	5.248411	0.640632	0.320192
51	6	0	4.205678	2.090232	2.008494
52	6	0	5.323010	1.687458	1.292043
53	6	0	1.531178	-2.145311	-1.165728
54	7	0	4.302344	-1.671057	-1.567795
55	6	0	2.278643	-2.873618	-2.074282
56	6	0	3.641134	-2.608516	-2.242846
57	6	0	1.732288	1.710453	2.393004
58	7	0	0.612824	1.649118	2.678426
59	6	0	6.600370	2.278635	1.488212
60	6	0	6.339102	0.226085	-0.393417
61	6	0	7.690788	1.884285	0.791343
62	6	0	7.607267	0.844422	-0.200253
63	7	0	8.672319	0.477574	-0.915965
64	1	0	0.475787	-2.334965	-1.019141
65	1	0	1.818883	-3.654701	-2.661635
66	1	0	4.220216	-3.181161	-2.954692
67	1	0	4.295840	2.883295	2.739249
68	1	0	6.692683	3.067357	2.223656
69	1	0	8.628427	2.371940	0.993970
70	1	0	6.207903	-0.579269	-1.090744
71	6	0	10.046716	0.906935	-0.630081
72	6	0	10.789899	-0.220555	0.084442
73	1	0	10.519358	1.120116	-1.590801
74	1	0	10.060023	1.826190	-0.059410
75	6	0	10.769112	-1.494985	-0.757589
76	1	0	11.814352	0.095318	0.280193
77	1	0	10.311896	-0.397262	1.051835
78	6	0	9.347137	-1.855978	-1.184622
79	1	0	11.380119	-1.340976	-1.651402
80	1	0	11.219579	-2.318999	-0.206168
81	6	0	8.673542	-0.655987	-1.847201
82	1	0	9.355003	-2.693766	-1.881649
83	1	0	8.754185	-2.156972	-0.316534
84	1	0	9.245561	-0.343374	-2.723169
85	1	0	7.670233	-0.881307	-2.187641

Ligand	Description	Total Gibbs Free energy (M06-2X/Def2TZVPP)
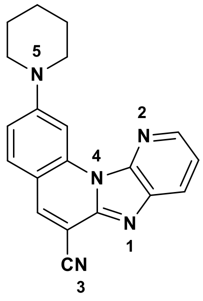 **3a**	2:1 complex with Zn^{2+} bound to N1	-3870.611194 a.u.

CARTESIAN COORDINATES

Center Number	Atomic Number	Atomic Type	Coordinates (Angstroms) X	Y	Z
1	7	0	-4.033935	-0.083694	-0.018499
2	6	0	-5.274895	-0.664329	0.284318
3	6	0	-2.878762	-0.457075	0.597383
4	7	0	-1.816520	0.226309	0.159596
5	6	0	-2.303921	1.103457	-0.797581
6	6	0	-3.685955	0.928384	-0.929580
7	6	0	-2.893744	-1.470172	1.592020
8	7	0	-4.481725	1.585910	-1.742696
9	6	0	-1.685422	2.060091	-1.587022
10	6	0	-3.877352	2.501025	-2.497213
11	6	0	-2.505078	2.765358	-2.450427
12	1	0	-4.512832	3.055695	-3.174266
13	1	0	-2.094496	3.527818	-3.095797
14	1	0	-0.621178	2.248781	-1.532147
15	6	0	-5.271630	-1.678460	1.292250
16	6	0	-4.099589	-2.064156	1.926793
17	1	0	-4.131955	-2.832969	2.687643
18	6	0	-6.419337	-0.266592	-0.350829
19	6	0	-6.529869	-2.256227	1.611751
20	6	0	-7.672834	-1.878232	0.994449
21	6	0	-7.668698	-0.871292	-0.033845
22	1	0	-6.563403	-3.021406	2.376465
23	1	0	-8.591466	-2.356091	1.287415
24	7	0	-8.788248	-0.522320	-0.671530
25	1	0	-6.344479	0.516512	-1.081020
26	6	0	-1.602214	-1.698994	2.100930
27	7	0	-0.463592	-1.653402	2.303792
28	6	0	-10.136795	-0.921351	-0.250493
29	6	0	-10.804664	0.245449	0.475437
30	1	0	-10.692876	-1.169797	-1.156503
31	1	0	-10.109437	-1.814265	0.360004
32	6	0	-10.844000	1.483372	-0.418704
33	1	0	-11.811503	-0.048094	0.771635
34	1	0	-10.243410	0.456109	1.389876
35	6	0	-9.460352	1.807585	-0.980111
36	1	0	-11.530475	1.300456	-1.250141
37	1	0	-11.238003	2.335255	0.133604
38	6	0	-8.858902	0.571796	-1.646070
39	1	0	-9.519658	2.615886	-1.708747
40	1	0	-8.792253	2.136051	-0.179127
41	1	0	-9.508318	0.230940	-2.455014
42	1	0	-7.886703	0.767724	-2.081901
43	30	0	0.000165	0.002611	0.993121
44	7	0	1.816740	-0.224210	0.160111
45	6	0	2.304068	-1.104742	-0.793982
46	6	0	3.686174	-0.930603	-0.926271
47	7	0	4.034310	0.084447	-0.018563
48	6	0	2.879139	0.460329	0.595786

49	6	0	2.894256	1.476863	1.586897
50	6	0	5.275389	0.665784	0.282435
51	6	0	4.100224	2.071631	1.919848
52	6	0	5.272264	1.683360	1.286880
53	6	0	1.685396	-2.063761	-1.580426
54	7	0	4.481914	-1.591152	-1.736983
55	6	0	2.504979	-2.772212	-2.441252
56	6	0	3.877404	-2.508563	-2.488566
57	6	0	1.602726	1.707821	2.094821
58	7	0	0.464067	1.663186	2.297684
59	6	0	6.530630	2.261811	1.604684
60	6	0	6.419851	0.265467	-0.351067
61	6	0	7.673621	1.881243	0.989022
62	6	0	7.669346	0.870763	-0.035780
63	7	0	8.788895	0.519158	-0.672011
64	1	0	0.621070	-2.251833	-1.525169
65	1	0	2.094261	-3.536627	-3.084220
66	1	0	4.512836	-3.065777	-3.163573
67	1	0	4.132678	2.843059	2.678041
68	1	0	6.564186	3.029823	2.366549
69	1	0	8.592284	2.360164	1.280149
70	1	0	6.345051	-0.520432	-1.078267
71	6	0	10.137788	0.916297	-0.250252
72	6	0	10.803238	-0.251283	0.476716
73	1	0	10.694954	1.163621	-1.155897
74	1	0	10.111410	1.809273	0.360182
75	6	0	10.841010	-1.489957	-0.416424
76	1	0	11.810395	0.040775	0.773308
77	1	0	10.241101	-0.460335	1.390979
78	6	0	9.457091	-1.812302	-0.978241
79	1	0	11.528175	-1.308848	-1.247686
80	1	0	11.233359	-2.342023	0.136779
81	6	0	8.858373	-0.575937	-1.645517
82	1	0	9.515356	-2.621319	-1.706163
83	1	0	8.788002	-2.138899	-0.177317
84	1	0	9.509138	-0.236868	-2.454138
85	1	0	7.886158	-0.770196	-2.082099

Ligand	Description	Total Gibbs Free energy (M06-2X/Def2TZVPP)
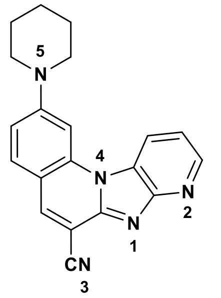 **3b**	2:1 complex with H^+ bound to N1	-2092.021947 a.u.

CARTESIAN COORDINATES

Center Number	Atomic Number	Atomic Type	Coordinates (Angstroms) X	Y	Z
1	7	0	-3.483215	0.427960	-0.322571
2	6	0	-4.508210	-0.394325	-0.793262
3	6	0	-2.148868	0.164794	-0.578712
4	7	0	-1.338581	1.056299	-0.061863
5	6	0	-2.142936	1.957651	0.585697
6	6	0	-3.499431	1.597095	0.441375
7	6	0	-1.788152	-0.995243	-1.328918
8	6	0	-4.466754	2.396197	1.038228
9	7	0	-1.698619	3.009103	1.258815
10	6	0	-4.003295	3.497551	1.736010
11	6	0	-2.632308	3.760495	1.820264
12	1	0	-4.702252	4.160322	2.224345
13	1	0	-2.284843	4.624009	2.374076
14	6	0	-4.131778	-1.538583	-1.529739
15	6	0	-2.770327	-1.820727	-1.783374
16	1	0	-2.512943	-2.709124	-2.344951
17	6	0	-5.841379	-0.117392	-0.542758
18	6	0	-5.163554	-2.380085	-1.990289
19	6	0	-6.477851	-2.115293	-1.749452
20	6	0	-6.863321	-0.955510	-1.020180
21	1	0	-4.892010	-3.258390	-2.561823
22	1	0	-7.223366	-2.780513	-2.152315
23	7	0	-8.179920	-0.653733	-0.826904
24	1	0	-6.093348	0.738235	0.047780
25	6	0	-0.398978	-1.240407	-1.535709
26	7	0	0.730035	-1.406763	-1.668864
27	6	0	-9.196992	-1.700805	-0.719868
28	6	0	-9.348816	-2.129511	0.739921
29	1	0	-10.136159	-1.283992	-1.090349
30	1	0	-8.952061	-2.545257	-1.353206
31	6	0	-9.678733	-0.929754	1.628051
32	1	0	-10.127018	-2.889403	0.818539
33	1	0	-8.410797	-2.588270	1.065730
34	6	0	-8.677857	0.208327	1.419125
35	1	0	-10.681040	-0.567767	1.379561
36	1	0	-9.701955	-1.229148	2.675447
37	6	0	-8.577598	0.528854	-0.070853
38	1	0	-8.984337	1.096460	1.972983
39	1	0	-7.693098	-0.089546	1.791410
40	1	0	-9.560311	0.826571	-0.444389
41	1	0	-7.904916	1.355046	-0.286665
42	1	0	0.286498	1.094035	0.040770
43	7	0	1.364931	1.107122	0.070051
44	6	0	2.158461	2.014447	-0.590875
45	6	0	3.497300	1.624788	-0.432510
46	7	0	3.463952	0.448325	0.334583
47	6	0	2.147162	0.177581	0.601924
48	6	0	1.768808	-0.963600	1.349941

49	6	0	4.493509	-0.380904	0.792556
50	6	0	2.760063	-1.791781	1.797966
51	6	0	4.113875	-1.521868	1.533910
52	7	0	1.719793	3.061683	-1.257419
53	6	0	4.476816	2.415500	-1.021642
54	6	0	2.664739	3.804033	-1.815899
55	6	0	4.030372	3.522886	-1.721409
56	6	0	0.387712	-1.227585	1.588024
57	7	0	-0.723486	-1.445521	1.774787
58	6	0	5.145614	-2.374077	1.984655
59	6	0	5.822897	-0.113863	0.523827
60	6	0	6.455813	-2.119986	1.727677
61	6	0	6.844940	-0.961832	0.990978
62	7	0	8.154896	-0.675357	0.778498
63	1	0	2.327262	4.670494	-2.370108
64	1	0	4.741785	4.177187	-2.202822
65	1	0	2.498429	-2.676394	2.363502
66	1	0	4.872218	-3.249504	2.559257
67	1	0	7.200206	-2.792305	2.120222
68	1	0	6.077917	0.733001	-0.076400
69	6	0	9.178833	-1.719848	0.700874
70	6	0	9.356354	-2.156390	-0.753719
71	1	0	10.108531	-1.295622	1.085721
72	1	0	8.925459	-2.560935	1.334842
73	6	0	9.695011	-0.961919	-1.645505
74	1	0	10.139801	-2.912344	-0.813004
75	1	0	8.426704	-2.623349	-1.091387
76	6	0	8.688343	0.175112	-1.460295
77	1	0	10.692478	-0.594816	-1.386127
78	1	0	9.733687	-1.269391	-2.689905
79	6	0	8.565418	0.505839	0.025414
80	1	0	9.001768	1.060155	-2.014864
81	1	0	7.709468	-0.126772	-1.845010
82	1	0	9.542142	0.803360	0.413854
83	1	0	7.891021	1.333736	0.227776
84	1	0	5.533539	2.219077	-0.967543
85	1	0	-5.526365	2.208170	0.992551

Ligand	Description	Total Gibbs Free energy (M06-2X/Def2TZVPP)
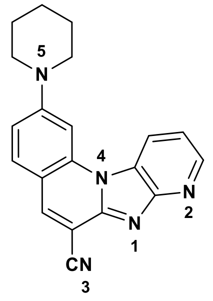 3b	2:1 complex with Ca^{2+} bound to N1	-2768.920212 a.u.

CARTESIAN COORDINATES

Center Number	Atomic Number	Atomic Type	X	Y	Z
1	7	0	4.480478	0.102110	0.039058
2	6	0	5.728368	0.690059	0.283420
3	6	0	3.353513	0.514379	0.702960
4	7	0	2.264309	-0.147598	0.348931
5	6	0	2.682931	-1.048521	-0.600133
6	6	0	4.065134	-0.924705	-0.825109
7	6	0	3.411850	1.556179	1.666666
8	6	0	4.653317	-1.763561	-1.762253
9	7	0	1.867342	-1.900210	-1.197161
10	6	0	3.805836	-2.660181	-2.394309
11	6	0	2.442504	-2.698633	-2.089442
12	1	0	4.199710	-3.341544	-3.133539
13	1	0	1.796559	-3.406689	-2.591744
14	6	0	5.770179	1.732503	1.255468
15	6	0	4.622489	2.152755	1.931427
16	1	0	4.696835	2.945683	2.663985
17	6	0	6.862780	0.274518	-0.370432
18	6	0	7.037267	2.314485	1.513410
19	6	0	8.162695	1.914740	0.874200
20	6	0	8.121708	0.878756	-0.116513
21	1	0	7.094559	3.108891	2.246314
22	1	0	9.088888	2.410490	1.108341
23	7	0	9.223692	0.509232	-0.785287
24	1	0	6.796700	-0.541335	-1.060333
25	6	0	2.133908	1.801782	2.220203
26	7	0	1.012290	1.782282	2.488556
27	6	0	10.589264	0.787832	-0.324754
28	6	0	11.150719	-0.457141	0.362372
29	1	0	11.183664	1.037477	-1.205608
30	1	0	10.613756	1.647257	0.332513
31	6	0	11.102420	-1.666062	-0.570762
32	1	0	12.174158	-0.258796	0.680165
33	1	0	10.561992	-0.652419	1.262919
34	6	0	9.704806	-1.861006	-1.159789
35	1	0	11.811887	-1.514520	-1.389105
36	1	0	11.419314	-2.563392	-0.041231
37	6	0	9.228584	-0.554700	-1.789982
38	1	0	9.711156	-2.650375	-1.911501
39	1	0	9.001928	-2.156656	-0.375222
40	1	0	9.928028	-0.246265	-2.569877
41	1	0	8.256404	-0.636981	-2.264900
42	20	0	-0.000088	0.003080	1.114722
43	7	0	-2.264606	0.150548	0.348897
44	6	0	-2.683960	1.049717	-0.601497
45	6	0	-4.066072	0.924435	-0.826281
46	7	0	-4.480569	-0.101409	0.039453
47	6	0	-3.353256	-0.511795	0.703929
48	6	0	-3.410706	-1.552248	1.669135

49	6	0	-5.727972	-0.689991	0.284722
50	6	0	-4.620841	-2.149473	1.934734
51	6	0	-5.768907	-1.731122	1.258213
52	7	0	-1.869055	1.901154	-1.199796
53	6	0	-4.654912	1.761356	-1.764718
54	6	0	-2.444857	2.697747	-2.093320
55	6	0	-3.808138	2.657702	-2.398145
56	6	0	-2.132549	-1.795961	2.223067
57	7	0	-1.010956	-1.775035	2.491416
58	6	0	-7.035515	-2.313775	1.517019
59	6	0	-6.862773	-0.276182	-0.369567
60	6	0	-8.161306	-1.915757	0.877369
61	6	0	-8.121200	-0.881021	-0.114671
62	7	0	-9.223540	-0.513192	-0.783809
63	1	0	-1.799478	3.405569	-2.596675
64	1	0	-4.202569	3.337588	-3.138437
65	1	0	-4.694521	-2.941422	2.668418
66	1	0	-7.092108	-3.107312	2.250918
67	1	0	-9.087078	-2.412051	1.112050
68	1	0	-6.797469	0.538949	-1.060402
69	6	0	-10.588871	-0.791064	-0.322129
70	6	0	-11.150426	0.455434	0.362175
71	1	0	-11.183539	-1.043132	-1.202112
72	1	0	-10.612826	-1.648761	0.337418
73	6	0	-11.102582	1.662273	-0.573662
74	1	0	-12.173757	0.257602	0.680644
75	1	0	-10.561555	0.652885	1.262154
76	6	0	-9.705118	1.856185	-1.163403
77	1	0	-11.812164	1.508785	-1.391541
78	1	0	-11.419569	2.560694	-0.046039
79	6	0	-9.228941	0.548538	-1.790813
80	1	0	-9.711757	2.643908	-1.916836
81	1	0	-9.002131	2.153649	-0.379617
82	1	0	-9.928670	0.238303	-2.569744
83	1	0	-8.256935	0.629752	-2.266299
84	1	0	-5.701926	1.750370	-2.018315
85	1	0	5.700352	-1.753827	-2.015812

Ligand	Description	Total Gibbs Free energy (M06-2X/Def2TZVPP)
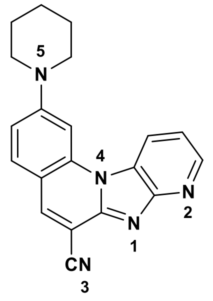 3b	2:1 complex with Cu^{2+} bound to N1	-3731.659244 a.u.

CARTESIAN COORDINATES

Center Number	Atomic Number	Atomic Type	Coordinates (Angstroms) X	Y	Z
1	7	0	4.000531	0.519541	0.282283
2	6	0	4.909146	-0.485745	0.619541
3	6	0	2.646479	0.397878	0.528662
4	7	0	1.967588	1.459207	0.149089
5	6	0	2.886489	2.329901	-0.382014
6	6	0	4.174821	1.770741	-0.318599
7	6	0	2.131899	-0.789120	1.128711
8	6	0	5.236335	2.501005	-0.843963
9	7	0	2.577318	3.509219	-0.894718
10	6	0	4.919798	3.737052	-1.374854
11	6	0	3.595507	4.199459	-1.378750
12	1	0	5.696560	4.356443	-1.798282
13	1	0	3.364826	5.169243	-1.800668
14	6	0	4.374399	-1.674532	1.191259
15	6	0	2.999122	-1.804290	1.436773
16	1	0	2.625553	-2.712910	1.889563
17	6	0	6.262738	-0.340883	0.431414
18	6	0	5.284192	-2.706062	1.526549
19	6	0	6.620813	-2.579458	1.338194
20	6	0	7.170395	-1.373444	0.789554
21	1	0	4.887125	-3.623788	1.940043
22	1	0	7.259026	-3.410839	1.584027
23	7	0	8.494561	-1.227731	0.629172
24	1	0	6.637823	0.587756	0.055496
25	6	0	0.736727	-0.859102	1.407540
26	7	0	-0.394086	-0.878880	1.614149
27	6	0	9.479613	-2.235707	1.033932
28	6	0	9.989602	-2.984245	-0.196800
29	1	0	10.303293	-1.692071	1.502071
30	1	0	9.067496	-2.894565	1.787610
31	6	0	10.596952	-1.996752	-1.190259
32	1	0	10.726533	-3.722768	0.117243
33	1	0	9.156549	-3.524320	-0.654632
34	6	0	9.625154	-0.862283	-1.503113
35	1	0	11.512555	-1.578492	-0.763420
36	1	0	10.882008	-2.509129	-2.108007
37	6	0	9.116121	-0.205302	-0.216098
38	1	0	10.103937	-0.100096	-2.117632
39	1	0	8.765965	-1.242485	-2.061721
40	1	0	9.948066	0.222945	0.346250
41	1	0	8.422986	0.596111	-0.437386
42	29	0	0.009925	1.656994	-0.004149
43	7	0	-1.952155	1.481396	-0.154673
44	6	0	-2.860400	2.354688	0.389897
45	6	0	-4.154131	1.807724	0.328270
46	7	0	-3.993630	0.558938	-0.281208
47	6	0	-2.642083	0.430488	-0.540515
48	6	0	-2.141875	-0.749805	-1.166283

49	6	0	-4.909891	-0.440873	-0.612734
50	6	0	-3.018706	-1.750853	-1.489554
51	6	0	-4.390754	-1.617878	-1.223715
52	7	0	-2.538380	3.528712	0.907234
53	6	0	-5.208618	2.550132	0.851560
54	6	0	-3.548388	4.227497	1.394898
55	6	0	-4.878507	3.780527	1.386500
56	6	0	-0.749868	-0.825018	-1.461628
57	7	0	0.377630	-0.851759	-1.683518
58	6	0	-5.308688	-2.637492	-1.573632
59	6	0	-6.258047	-0.304501	-0.383738
60	6	0	-6.641355	-2.512982	-1.358136
61	6	0	-7.172635	-1.327397	-0.751381
62	7	0	-8.491547	-1.191507	-0.545249
63	1	0	-3.307229	5.192853	1.821081
64	1	0	-5.649127	4.408927	1.807921
65	1	0	-2.656592	-2.650938	-1.967809
66	1	0	-4.920499	-3.544427	-2.018075
67	1	0	-7.289130	-3.335402	-1.610971
68	1	0	-6.629141	0.612964	0.023614
69	6	0	-9.494151	-2.128951	-1.061371
70	6	0	-10.051019	-2.983475	0.075377
71	1	0	-10.294684	-1.517180	-1.484800
72	1	0	-9.082248	-2.716643	-1.872267
73	6	0	-10.654914	-2.081361	1.147632
74	1	0	-10.799280	-3.664511	-0.328610
75	1	0	-9.244531	-3.590741	0.494813
76	6	0	-9.654077	-1.020868	1.595911
77	1	0	-11.544962	-1.591631	0.743462
78	1	0	-10.980089	-2.672115	2.002743
79	6	0	-9.088274	-0.245154	0.400497
80	1	0	-10.120178	-0.309403	2.277335
81	1	0	-8.822321	-1.488173	2.128931
82	1	0	-9.885872	0.288144	-0.120856
83	1	0	-8.358789	0.481855	0.734533
84	1	0	-6.236161	2.228501	0.866903
85	1	0	6.258370	2.162760	-0.869329

Ligand	Description	Total Gibbs Free energy (M06-2X/Def2TZVPP)
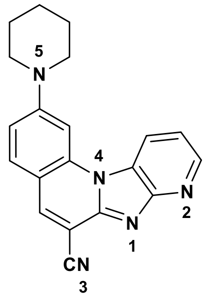 **3b**	2:1 complex with Mg^{2+} bound to N1	-2291.353398 a.u.

CARTESIAN COORDINATES

Center Number	Atomic Number	Atomic Type	Coordinates (Angstroms) X	Y	Z
1	7	0	4.017249	0.032698	0.150940
2	6	0	5.266771	0.649333	0.305193
3	6	0	2.929152	0.435790	0.875226
4	7	0	1.833782	-0.260669	0.602379
5	6	0	2.209994	-1.176893	-0.356935
6	6	0	3.572126	-1.021766	-0.665811
7	6	0	3.027467	1.499425	1.812123
8	6	0	4.128368	-1.862487	-1.620762
9	7	0	1.387162	-2.059637	-0.889509
10	6	0	3.271907	-2.793336	-2.185612
11	6	0	1.930711	-2.859788	-1.798969
12	1	0	3.640682	-3.478603	-2.934166
13	1	0	1.274935	-3.593929	-2.248482
14	6	0	5.349048	1.713597	1.252764
15	6	0	4.240420	2.126044	1.989728
16	1	0	4.342767	2.934893	2.701063
17	6	0	6.366205	0.241934	-0.408613
18	6	0	6.618897	2.324690	1.420287
19	6	0	7.709712	1.932530	0.721249
20	6	0	7.627570	0.874690	-0.244891
21	1	0	6.704871	3.135294	2.132318
22	1	0	8.638614	2.450268	0.886857
23	7	0	8.691788	0.510358	-0.971357
24	1	0	6.276037	-0.590177	-1.075373
25	6	0	1.775586	1.713114	2.427180
26	7	0	0.662845	1.619603	2.720690
27	6	0	10.078787	0.830193	-0.611098
28	6	0	10.723178	-0.394303	0.039200
29	1	0	10.601192	1.091829	-1.533187
30	1	0	10.125520	1.692667	0.040743
31	6	0	10.642261	-1.611014	-0.881077
32	1	0	11.760745	-0.164092	0.280408
33	1	0	10.207631	-0.599409	0.981374
34	6	0	9.211549	-1.849311	-1.364616
35	1	0	11.285405	-1.445883	-1.749991
36	1	0	11.022586	-2.494759	-0.370952
37	6	0	8.654400	-0.562192	-1.966918
38	1	0	9.184718	-2.643898	-2.110278
39	1	0	8.576230	-2.157747	-0.529072
40	1	0	9.287088	-0.240659	-2.797004
41	1	0	7.653412	-0.675553	-2.369784
42	12	0	0.000036	-0.000403	1.484936
43	7	0	-1.833667	0.260351	0.602429
44	6	0	-2.209811	1.176798	-0.356702
45	6	0	-3.571990	1.021920	-0.665505
46	7	0	-4.017199	-0.032651	0.151059
47	6	0	-2.929115	-0.436008	0.875215
48	6	0	-3.027521	-1.499769	1.811959

49	6	0	-5.266768	-0.649226	0.305190
50	6	0	-4.240546	-2.126269	1.989513
51	6	0	-5.349139	-1.713607	1.252622
52	7	0	-1.386904	2.059538	-0.889167
53	6	0	-4.128206	1.862938	-1.620209
54	6	0	-1.930425	2.859960	-1.798406
55	6	0	-3.271667	2.793785	-2.184941
56	6	0	-1.775659	-1.713735	2.426948
57	7	0	-0.662905	-1.620503	2.720510
58	6	0	-6.619032	-2.324633	1.420029
59	6	0	-6.366143	-0.241688	-0.408622
60	6	0	-7.709791	-1.932343	0.720977
61	6	0	-7.627552	-0.874412	-0.245063
62	7	0	-8.691714	-0.509986	-0.971565
63	1	0	-1.274581	3.594087	-2.247844
64	1	0	-3.640411	3.479276	-2.933305
65	1	0	-4.342972	-2.935198	2.700746
66	1	0	-6.705097	-3.135285	2.131995
67	1	0	-8.638734	-2.450019	0.886535
68	1	0	-6.275867	0.590455	-1.075323
69	6	0	-10.078720	-0.830187	-0.611646
70	6	0	-10.723445	0.393957	0.038973
71	1	0	-10.600912	-1.091580	-1.533924
72	1	0	-10.125416	-1.692910	0.039864
73	6	0	-10.642633	1.610980	-0.880895
74	1	0	-11.761000	0.163463	0.279961
75	1	0	-10.208069	0.598855	0.981287
76	6	0	-9.211896	1.849704	-1.364141
77	1	0	-11.285618	1.446001	-1.749957
78	1	0	-11.023207	2.494490	-0.370548
79	6	0	-8.654388	0.562896	-1.966779
80	1	0	-9.185101	2.644529	-2.109550
81	1	0	-8.576762	2.157998	-0.528405
82	1	0	-9.286876	0.241503	-2.797073
83	1	0	-7.653370	0.676629	-2.369451
84	1	0	-5.157673	1.830045	-1.934937
85	1	0	5.157785	-1.829340	-1.935621

Ligand	Description	Total Gibbs Free energy (M06-2X/Def2TZVPP)
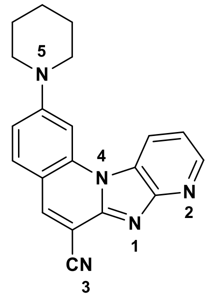 3b	2:1 complex with Zn^{2+} bound to N1	-3870.609311 a.u.

CARTESIAN COORDINATES

Center Number	Atomic Number	Atomic Type	Coordinates (Angstroms) X	Y	Z
1	7	0	4.043605	0.069732	-0.001491
2	6	0	5.282153	0.672050	0.262452
3	6	0	2.908535	0.462226	0.651172
4	7	0	1.838186	-0.224338	0.271529
5	6	0	2.276282	-1.118684	-0.681004
6	6	0	3.657687	-0.959813	-0.879244
7	6	0	2.937960	1.498639	1.620149
8	6	0	4.281183	-1.775561	-1.814375
9	7	0	1.492906	-1.981099	-1.296005
10	6	0	3.467153	-2.686088	-2.468071
11	6	0	2.100756	-2.758283	-2.184633
12	1	0	3.889552	-3.350861	-3.206699
13	1	0	1.478351	-3.476494	-2.702057
14	6	0	5.297368	1.712587	1.238495
15	6	0	4.138127	2.112412	1.900960
16	1	0	4.190422	2.903026	2.637770
17	6	0	6.430494	0.271987	-0.374575
18	6	0	6.553628	2.311524	1.515535
19	6	0	7.692145	1.928106	0.891776
20	6	0	7.678920	0.892057	-0.101160
21	1	0	6.589621	3.104600	2.251156
22	1	0	8.608484	2.434806	1.140551
23	7	0	8.794018	0.536117	-0.751609
24	1	0	6.387041	-0.544565	-1.064581
25	6	0	1.648051	1.716209	2.154011
26	7	0	0.516881	1.642154	2.376049
27	6	0	10.150347	0.844677	-0.281039
28	6	0	10.733401	-0.388214	0.409742
29	1	0	10.744905	1.107235	-1.157955
30	1	0	10.151581	1.704194	0.376364
31	6	0	10.719404	-1.597078	-0.524319
32	1	0	11.749591	-0.166836	0.735370
33	1	0	10.142431	-0.596932	1.305748
34	6	0	9.331244	-1.822284	-1.124348
35	1	0	11.431991	-1.429606	-1.336766
36	1	0	11.051472	-2.487539	0.007397
37	6	0	8.830754	-0.526594	-1.757831
38	1	0	9.360802	-2.610168	-1.876988
39	1	0	8.628683	-2.134345	-0.345937
40	1	0	9.529368	-0.201261	-2.531502
41	1	0	7.864832	-0.630793	-2.240827
42	30	0	0.000012	0.000003	1.009807
43	7	0	-1.838146	0.224270	0.271425
44	6	0	-2.276175	1.118428	-0.681311
45	6	0	-3.657622	0.959734	-0.879394
46	7	0	-4.043622	-0.069570	-0.001392
47	6	0	-2.908573	-0.462027	0.651328
48	6	0	-2.938102	-1.498075	1.620697

49	6	0	-5.282202	-0.671785	0.262656
50	6	0	-4.138350	-2.111537	1.901828
51	6	0	-5.297545	-1.711859	1.239187
52	7	0	-1.492721	1.980620	-1.296531
53	6	0	-4.281111	1.775546	-1.814479
54	6	0	-2.100539	2.757770	-2.185207
55	6	0	-3.466999	2.685822	-2.468416
56	6	0	-1.648201	-1.715618	2.154590
57	7	0	-0.517015	-1.641630	2.376574
58	6	0	-6.553857	-2.310593	1.516411
59	6	0	-6.430443	-0.272088	-0.374782
60	6	0	-7.692288	-1.927480	0.892304
61	6	0	-7.678909	-0.892019	-0.101243
62	7	0	-8.793903	-0.536528	-0.752136
63	1	0	-1.478069	3.475784	-2.702826
64	1	0	-3.889384	3.350618	-3.207031
65	1	0	-4.190743	-2.901820	2.638985
66	1	0	-6.589976	-3.103237	2.252492
67	1	0	-8.608688	-2.433932	1.141352
68	1	0	-6.386812	0.543911	-1.065420
69	6	0	-10.150264	-0.845299	-0.281777
70	6	0	-10.733343	0.387305	0.409475
71	1	0	-10.744736	-1.107424	-1.158877
72	1	0	-10.151566	-1.705194	0.375135
73	6	0	-10.719393	1.596457	-0.524231
74	1	0	-11.749522	0.165817	0.735060
75	1	0	-10.142348	0.595746	1.305533
76	6	0	-9.331276	1.821884	-1.124285
77	1	0	-11.432028	1.429206	-1.336680
78	1	0	-11.051449	2.486758	0.007762
79	6	0	-8.830702	0.526397	-1.758139
80	1	0	-9.360919	2.609975	-1.876706
81	1	0	-8.628688	2.133776	-0.345829
82	1	0	-9.529279	0.201199	-2.531891
83	1	0	-7.864798	0.630815	-2.241120
84	1	0	-5.330866	1.737573	-2.051668
85	1	0	5.330864	-1.737282	-2.051845

Ligand	Description	Total Gibbs Free energy (M06-2X/Def2TZVPP)
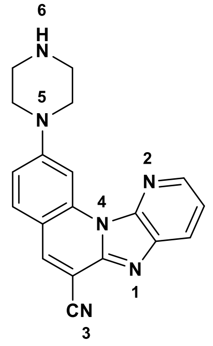 **4a**	2:1 complex with H^{+} bound to N1	-2124.123413 a.u.

CARTESIAN COORDINATES

Center Number	Atomic Number	Atomic Type	Coordinates (Angstroms) X	Y	Z
1	7	0	-3.465576	0.500701	-0.368163
2	6	0	-4.458230	-0.368217	-0.826236
3	6	0	-2.122187	0.316183	-0.616096
4	7	0	-1.363232	1.259578	-0.091390
5	6	0	-2.232962	2.111228	0.549069
6	6	0	-3.555701	1.667522	0.391878
7	6	0	-1.708147	-0.823404	-1.369524
8	7	0	-4.639925	2.246467	0.869241
9	6	0	-2.021537	3.270755	1.284828
10	6	0	-4.416475	3.355953	1.567416
11	6	0	-3.145339	3.892813	1.796798
12	1	0	-5.291641	3.848757	1.970214
13	1	0	-3.055244	4.797840	2.379684
14	1	0	-1.027563	3.663664	1.451743
15	6	0	-4.026758	-1.489362	-1.569487
16	6	0	-2.655248	-1.695383	-1.825233
17	1	0	-2.351673	-2.567324	-2.390004
18	6	0	-5.793239	-0.145738	-0.548499
19	6	0	-5.022765	-2.377578	-2.023748
20	6	0	-6.343606	-2.172633	-1.762270
21	6	0	-6.775916	-1.037107	-1.015832
22	1	0	-4.719796	-3.238848	-2.605333
23	1	0	-7.063117	-2.867300	-2.163310
24	7	0	-8.099771	-0.806104	-0.799203
25	1	0	-6.047042	0.706155	0.054131
26	6	0	-0.312939	-1.014232	-1.581668
27	7	0	0.821153	-1.139753	-1.720536
28	6	0	-9.080278	-1.887100	-0.709378
29	6	0	-9.303794	-2.243133	0.757824
30	1	0	-10.015950	-1.533712	-1.145218
31	1	0	-8.754972	-2.759065	-1.263738
32	7	0	-9.748175	-1.056633	1.471950
33	1	0	-10.070762	-3.013501	0.833338
34	1	0	-8.362606	-2.652805	1.157459
35	6	0	-8.774895	0.023977	1.399099
36	1	0	-9.973568	-1.277442	2.431747
37	6	0	-8.568800	0.368528	-0.070295
38	1	0	-9.162918	0.891056	1.932550
39	1	0	-7.802502	-0.246505	1.839188
40	1	0	-9.531577	0.650154	-0.500446
41	1	0	-7.887761	1.201999	-0.207188
42	1	0	0.289466	1.194004	0.103208
43	7	0	1.357732	1.199475	0.160558
44	6	0	2.178260	2.080650	-0.504510
45	6	0	3.495608	1.639558	-0.359605
46	7	0	3.430834	0.455689	0.395429

47	6	0	2.114827	0.225880	0.669635
48	6	0	1.709801	-0.918460	1.391457
49	6	0	4.449994	-0.404253	0.818756
50	6	0	2.687196	-1.785688	1.812025
51	6	0	4.043319	-1.550163	1.543626
52	6	0	1.930341	3.232813	-1.232773
53	7	0	4.565175	2.231178	-0.845419
54	6	0	3.044842	3.863951	-1.755678
55	6	0	4.323131	3.340729	-1.539164
56	6	0	0.320184	-1.141597	1.613469
57	7	0	-0.805200	-1.304252	1.772684
58	6	0	5.063270	-2.433746	1.963178
59	6	0	5.773276	-0.150813	0.526242
60	6	0	6.372992	-2.200737	1.686391
61	6	0	6.779642	-1.038874	0.958332
62	7	0	8.087822	-0.786537	0.721973
63	1	0	0.928170	3.607251	-1.386041
64	1	0	2.938160	4.767203	-2.338094
65	1	0	5.189203	3.842689	-1.949294
66	1	0	2.404200	-2.676407	2.357732
67	1	0	4.782739	-3.312711	2.528889
68	1	0	7.108027	-2.894761	2.058420
69	1	0	6.005273	0.716269	-0.062308
70	6	0	9.113489	-1.828739	0.673734
71	6	0	9.424928	-2.155408	-0.784477
72	1	0	10.010617	-1.445460	1.162091
73	1	0	8.793703	-2.720684	1.197704
74	7	0	9.856927	-0.944035	-1.461044
75	1	0	10.225135	-2.893547	-0.826256
76	1	0	8.523318	-2.598145	-1.236188
77	6	0	8.839748	0.095185	-1.428268
78	1	0	10.147327	-1.141688	-2.408290
79	6	0	8.547635	0.416110	0.031774
80	1	0	9.218669	0.983517	-1.932177
81	1	0	7.901097	-0.207513	-1.918217
82	1	0	9.476381	0.727249	0.513404
83	1	0	7.830857	1.222366	0.141282

Ligand	Description	Total Gibbs Free energy (M06-2X/Def2TZVPP)
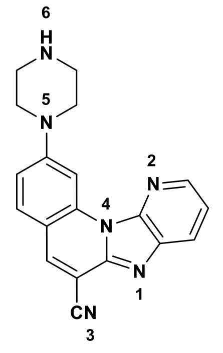 **4a**	2:1 complex with Ca^{2+} bound to N1	-2801.004675 a.u.

CARTESIAN COORDINATES

Center Number	Atomic Number	Atomic Type	Coordinates (Angstroms) X	Y	Z
1	7	0	-4.337387	0.131247	0.245179
2	6	0	-5.507355	-0.625196	0.091150
3	6	0	-3.181825	-0.378950	0.766525
4	7	0	-2.190146	0.509798	0.831964
5	6	0	-2.735581	1.684562	0.326959
6	6	0	-4.068237	1.475756	-0.048056
7	6	0	-3.145933	-1.740070	1.183561
8	7	0	-4.889424	2.365766	-0.561804
9	6	0	-2.205579	2.956401	0.155440
10	6	0	-4.365697	3.577746	-0.726010
11	6	0	-3.050912	3.909283	-0.384240
12	1	0	-5.021602	4.327429	-1.147800
13	1	0	-2.706042	4.920358	-0.543441
14	1	0	-1.189693	3.196557	0.439465
15	6	0	-5.443548	-1.989135	0.510936
16	6	0	-4.280522	-2.522384	1.049268
17	1	0	-4.264355	-3.557778	1.363953
18	6	0	-6.642570	-0.079158	-0.444772
19	6	0	-6.624551	-2.760079	0.345610
20	6	0	-7.754997	-2.239446	-0.186286
21	6	0	-7.817345	-0.864793	-0.604420
22	1	0	-6.612228	-3.793408	0.667447
23	1	0	-8.622674	-2.872248	-0.257863
24	7	0	-8.935742	-0.342749	-1.119124
25	1	0	-6.603662	0.942922	-0.770438
26	6	0	-1.898597	-2.108587	1.712076
27	7	0	-0.809706	-2.172864	2.097338
28	6	0	-10.090205	-1.130074	-1.569294
29	6	0	-11.371164	-0.621521	-0.925970
30	1	0	-10.165874	-0.999094	-2.651812
31	1	0	-9.945776	-2.182535	-1.367154
32	7	0	-11.526818	0.777232	-1.273632
33	1	0	-12.211464	-1.191169	-1.319756
34	1	0	-11.316531	-0.791169	0.159951
35	6	0	-10.425990	1.566730	-0.761350
36	1	0	-12.417958	1.140401	-0.965670
37	6	0	-9.122638	1.086662	-1.391536
38	1	0	-10.572655	2.612613	-1.027253
39	1	0	-10.325580	1.500966	0.332650
40	1	0	-9.162736	1.231658	-2.473730
41	1	0	-8.300626	1.663209	-0.986136
42	20	0	0.025961	0.057901	1.704362
43	7	0	2.228702	-0.428662	0.817225
44	6	0	2.764172	-1.620287	0.342151
45	6	0	4.091341	-1.426715	-0.059895
46	7	0	4.367928	-0.074575	0.187472

47	6	0	3.220765	0.455451	0.707847
48	6	0	3.191509	1.830759	1.075293
49	6	0	5.535447	0.675030	-0.013007
50	6	0	4.326038	2.605707	0.899142
51	6	0	5.480828	2.051496	0.365707
52	6	0	2.228651	-2.895204	0.218367
53	7	0	4.901784	-2.334370	-0.559911
54	6	0	3.062806	-3.866566	-0.305703
55	6	0	4.372342	-3.549300	-0.678876
56	6	0	1.951765	2.220018	1.606136
57	7	0	0.868053	2.299814	2.003196
58	6	0	6.665607	2.809589	0.169760
59	6	0	6.664494	0.105936	-0.536403
60	6	0	7.791903	2.265304	-0.346499
61	6	0	7.843343	0.879411	-0.730241
62	7	0	8.947333	0.341968	-1.257123
63	1	0	1.216994	-3.123683	0.526089
64	1	0	2.713082	-4.881060	-0.428350
65	1	0	5.019418	-4.313462	-1.088133
66	1	0	4.316701	3.650650	1.180853
67	1	0	6.654668	3.857803	0.439342
68	1	0	8.646870	2.903920	-0.483780
69	1	0	6.632399	-0.939738	-0.774839
70	6	0	10.252234	1.009102	-1.333101
71	6	0	11.271254	0.228292	-0.509142
72	1	0	10.562816	1.020462	-2.379387
73	1	0	10.194290	2.028723	-0.978328
74	7	0	11.342246	-1.129431	-1.011610
75	1	0	12.244671	0.705252	-0.613401
76	1	0	10.973730	0.280024	0.549370
77	6	0	10.065184	-1.807445	-0.905669
78	1	0	12.081201	-1.652759	-0.563575
79	6	0	9.047223	-1.039822	-1.739478
80	1	0	10.157361	-2.818070	-1.300763
81	1	0	9.694954	-1.872725	0.129107
82	1	0	9.398732	-0.995384	-2.772319
83	1	0	8.082632	-1.529925	-1.731613

Ligand	Description	Total Gibbs Free energy (M06-2X/Def2TZVPP)
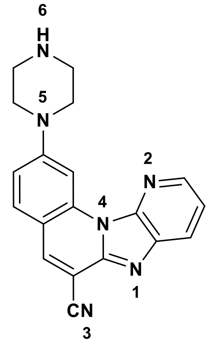<br>**4a**	2:1 complex with Cu^{2+} bound to N1	-3763.762625 a.u.

CARTESIAN COORDINATES

Center Number	Atomic Number	Atomic Type	Coordinates (Angstroms) X	Y	Z
1	7	0	-4.158339	-0.043829	-0.351912
2	6	0	-5.307374	-0.697775	0.084331
3	6	0	-2.897111	-0.580923	-0.227910
4	7	0	-1.955217	0.209449	-0.703047
5	6	0	-2.609244	1.329798	-1.167848
6	6	0	-3.988003	1.198809	-0.961103
7	6	0	-2.745464	-1.867591	0.373654
8	7	0	-4.923853	2.072065	-1.279815
9	6	0	-2.144742	2.493736	-1.776617
10	6	0	-4.462676	3.176191	-1.858652
11	6	0	-3.104983	3.420250	-2.121786
12	1	0	-5.203960	3.914623	-2.134250
13	1	0	-2.825410	4.348981	-2.596805
14	1	0	-1.091819	2.658214	-1.954041
15	6	0	-5.138178	-1.976577	0.679825
16	6	0	-3.849056	-2.540757	0.816389
17	1	0	-3.740830	-3.515928	1.271588
18	6	0	-6.552971	-0.135341	-0.067052
19	6	0	-6.298711	-2.659741	1.116220
20	6	0	-7.539554	-2.128975	0.966199
21	6	0	-7.708744	-0.836201	0.368200
22	1	0	-6.182166	-3.626949	1.586601
23	1	0	-8.386184	-2.678391	1.341868
24	7	0	-8.931669	-0.296881	0.211908
25	1	0	-6.616388	0.817056	-0.563518
26	6	0	-1.419650	-2.385222	0.472010
27	7	0	-0.328211	-2.739872	0.514531
28	6	0	-10.181138	-1.010471	0.482539
29	6	0	-10.829736	-0.422331	1.741282
30	1	0	-10.837957	-0.826175	-0.368105
31	1	0	-10.012826	-2.075869	0.562255
32	7	0	-11.048872	0.983366	1.502403
33	1	0	-11.781362	-0.922252	1.910775
34	1	0	-10.169984	-0.626937	2.598184
35	6	0	-9.829140	1.707130	1.245944
36	1	0	-11.632554	1.421266	2.200565
37	6	0	-9.171829	1.129795	-0.019292
38	1	0	-10.052788	2.757031	1.066189
39	1	0	-9.096828	1.642459	2.064630
40	1	0	-9.856948	1.224627	-0.861140
41	1	0	-8.250985	1.654923	-0.235205
42	29	0	0.035998	-0.036840	-0.610615
43	7	0	1.996941	-0.333763	-0.638654
44	6	0	2.712896	-1.437488	-1.056698
45	6	0	4.081537	-1.223953	-0.848544
46	7	0	4.182513	0.052266	-0.282989

47	6	0	2.900080	0.527625	-0.186372
48	6	0	2.668843	1.822061	0.357150
49	6	0	5.301183	0.785006	0.127713
50	6	0	3.744432	2.567412	0.777786
51	6	0	5.049903	2.072547	0.674142
52	6	0	2.317022	-2.644062	-1.617823
53	7	0	5.057837	-2.060574	-1.133701
54	6	0	3.325697	-3.537051	-1.925803
55	6	0	4.662924	-3.211202	-1.671476
56	6	0	1.314535	2.242438	0.416991
57	7	0	0.185200	2.469686	0.410040
58	6	0	6.176687	2.815647	1.099661
59	6	0	6.571992	0.274475	0.014596
60	6	0	7.439779	2.330701	0.995531
61	6	0	7.691317	1.033180	0.438485
62	7	0	8.942739	0.561350	0.304862
63	1	0	1.274939	-2.870834	-1.795246
64	1	0	3.094702	-4.496424	-2.364825
65	1	0	5.446529	-3.915925	-1.916060
66	1	0	3.584267	3.552501	1.196390
67	1	0	6.016505	3.804607	1.509199
68	1	0	8.255311	2.958490	1.311164
69	1	0	6.680976	-0.719032	-0.377355
70	6	0	10.115250	1.113751	0.985582
71	6	0	10.619109	0.103103	2.014093
72	1	0	10.891093	1.290887	0.238805
73	1	0	9.882213	2.050903	1.473078
74	7	0	10.927327	-1.143498	1.340795
75	1	0	11.521742	0.494434	2.481274
76	1	0	9.847923	-0.008038	2.791905
77	6	0	9.764039	-1.709508	0.684062
78	1	0	11.362155	-1.805862	1.967440
79	6	0	9.273019	-0.704273	-0.350986
80	1	0	10.046187	-2.634183	0.182648
81	1	0	8.939003	-1.929103	1.379425
82	1	0	10.084839	-0.500327	-1.052116
83	1	0	8.430256	-1.084076	-0.914971

Ligand	Description	Total Gibbs Free energy (M06-2X/Def2TZVPP)
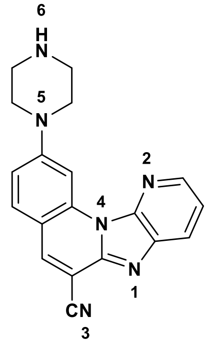 4a	2:1 complex with Mg^{2+} bound to N1	-2323.443127 a.u.

CARTESIAN COORDINATES

Center Number	Atomic Number	Atomic Type	Coordinates (Angstroms) X	Y	Z
1	7	0	-3.991806	0.019315	0.211791
2	6	0	-5.261997	-0.543446	0.406153
3	6	0	-2.893606	-0.381904	0.910688
4	7	0	-1.785533	0.289304	0.581641
5	6	0	-2.186685	1.190081	-0.396369
6	6	0	-3.555992	1.043050	-0.645728
7	6	0	-3.008431	-1.415758	1.880181
8	7	0	-4.273658	1.729613	-1.506750
9	6	0	-1.492382	2.150949	-1.116190
10	6	0	-3.595794	2.648214	-2.191037
11	6	0	-2.227618	2.886833	-2.028551
12	1	0	-4.165238	3.226482	-2.906105
13	1	0	-1.753737	3.653338	-2.623832
14	1	0	-0.432878	2.320285	-0.974385
15	6	0	-5.354953	-1.581319	1.385397
16	6	0	-4.244247	-1.999602	2.103996
17	1	0	-4.347958	-2.786615	2.839487
18	6	0	-6.344021	-0.120059	-0.315931
19	6	0	-6.639725	-2.157336	1.575988
20	6	0	-7.721117	-1.754575	0.869656
21	6	0	-7.624438	-0.707854	-0.113046
22	1	0	-6.747225	-2.936558	2.319442
23	1	0	-8.669018	-2.217010	1.083683
24	7	0	-8.691822	-0.301159	-0.805771
25	1	0	-6.187335	0.640954	-1.056484
26	6	0	-1.763027	-1.666788	2.479720
27	7	0	-0.641550	-1.626981	2.760666
28	6	0	-9.982520	-1.001415	-0.836744
29	6	0	-11.114622	-0.054596	-0.467050
30	1	0	-10.134787	-1.347501	-1.862049
31	1	0	-9.972496	-1.866158	-0.187671
32	7	0	-11.101037	1.053504	-1.401330
33	1	0	-12.059361	-0.589725	-0.549203
34	1	0	-10.986681	0.262650	0.578886
35	6	0	-9.857216	1.790373	-1.319003
36	1	0	-11.899970	1.658636	-1.273348
37	6	0	-8.699910	0.869226	-1.691233
38	1	0	-9.877684	2.620290	-2.023924
39	1	0	-9.666104	2.197300	-0.314396
40	1	0	-8.823585	0.522293	-2.719666
41	1	0	-7.769834	1.417348	-1.611366
42	12	0	0.000056	0.002287	1.577309
43	7	0	1.785624	-0.286987	0.582169
44	6	0	2.186409	-1.189857	-0.394062
45	6	0	3.555728	-1.043695	-0.643892
46	7	0	3.991922	-0.018350	0.211504

47	6	0	2.893910	0.384592	0.909707
48	6	0	3.009118	1.420425	1.877056
49	6	0	5.262267	0.544535	0.404505
50	6	0	4.245098	2.004457	2.099470
51	6	0	5.355594	1.584434	1.381570
52	6	0	1.491761	-2.151978	-1.111874
53	7	0	4.273099	-1.732169	-1.503629
54	6	0	2.226680	-2.889874	-2.022867
55	6	0	3.594896	-2.651949	-2.186004
56	6	0	1.763815	1.672879	2.476192
57	7	0	0.642340	1.633638	2.757198
58	6	0	6.640527	2.160559	1.570735
59	6	0	6.344087	0.119362	-0.316819
60	6	0	7.721730	1.756064	0.865100
61	6	0	7.624690	0.707273	-0.115370
62	7	0	8.691853	0.298883	-0.807407
63	1	0	0.432225	-2.320742	-0.969611
64	1	0	1.752513	-3.657436	-2.616556
65	1	0	4.164097	-3.231794	-2.899986
66	1	0	4.349101	2.792962	2.833318
67	1	0	6.748320	2.941348	2.312502
68	1	0	8.669735	2.218805	1.077989
69	1	0	6.187088	-0.643232	-1.055673
70	6	0	9.983120	0.998025	-0.839167
71	6	0	11.114126	0.051174	-0.466151
72	1	0	10.136464	1.341354	-1.865229
73	1	0	9.973315	1.864449	-0.192336
74	7	0	11.100395	-1.059260	-1.397660
75	1	0	12.059390	0.585293	-0.548847
76	1	0	10.985023	-0.263323	0.580475
77	6	0	9.855830	-1.794821	-1.314732
78	1	0	11.898671	-1.664781	-1.267431
79	6	0	8.699735	-0.873478	-1.690253
80	1	0	9.876222	-2.626436	-2.017651
81	1	0	9.663392	-2.199167	-0.309335
82	1	0	8.824901	-0.528887	-2.719285
83	1	0	7.769050	-1.420535	-1.610307

Ligand	Description	Total Gibbs Free energy (M06-2X/Def2TZVPP)
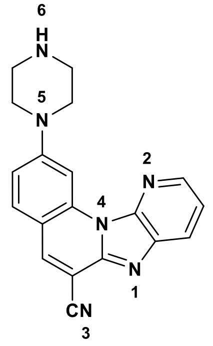 **4a**	2:1 complex with Zn^{2+} bound to N1	-3902.701201 a.u.

CARTESIAN COORDINATES

Center Number	Atomic Number	Atomic Type	Coordinates (Angstroms) X	Y	Z
1	7	0	-4.034819	-0.008579	0.034165
2	6	0	-5.286224	-0.567419	0.334924
3	6	0	-2.886610	-0.406176	0.647968
4	7	0	-1.812616	0.262289	0.216256
5	6	0	-2.284519	1.156278	-0.733092
6	6	0	-3.669280	1.006535	-0.866702
7	6	0	-2.920002	-1.424457	1.637086
8	7	0	-4.453582	1.686329	-1.672600
9	6	0	-1.649327	2.109545	-1.513319
10	6	0	-3.833423	2.598316	-2.417965
11	6	0	-2.456574	2.837735	-2.369285
12	1	0	-4.459155	3.170765	-3.089280
13	1	0	-2.032748	3.598985	-3.007477
14	1	0	-0.581891	2.278780	-1.457069
15	6	0	-5.299706	-1.594652	1.329195
16	6	0	-4.135051	-2.003507	1.963379
17	1	0	-4.180361	-2.781224	2.714460
18	6	0	-6.422558	-0.150247	-0.302653
19	6	0	-6.564272	-2.169574	1.626842
20	6	0	-7.698569	-1.774717	1.003923
21	6	0	-7.681459	-0.737639	0.006576
22	1	0	-6.611676	-2.940422	2.385101
23	1	0	-8.626591	-2.233913	1.297746
24	7	0	-8.801157	-0.340166	-0.603657
25	1	0	-6.327058	0.603278	-1.061135
26	6	0	-1.633296	-1.676533	2.147492
27	7	0	-0.494550	-1.649891	2.352801
28	6	0	-10.091679	-1.034856	-0.515973
29	6	0	-11.170404	-0.090538	-0.006411
30	1	0	-10.352849	-1.352355	-1.528270
31	1	0	-10.018355	-1.917851	0.103993
32	7	0	-11.247167	1.037543	-0.913867
33	1	0	-12.123609	-0.616763	0.002112
34	1	0	-10.927804	0.202742	1.026256
35	6	0	-9.995286	1.764509	-0.957427
36	1	0	-12.021793	1.645579	-0.687911
37	6	0	-8.894007	0.840917	-1.469445
38	1	0	-10.086900	2.606410	-1.642182
39	1	0	-9.690362	2.152683	0.026338
40	1	0	-9.135542	0.508045	-2.481185
41	1	0	-7.955022	1.379304	-1.488421
42	30	0	-0.000021	0.001947	1.048406
43	7	0	1.812509	-0.260517	0.216688
44	6	0	2.284093	-1.156567	-0.730867
45	6	0	3.668918	-1.007635	-0.864734
46	7	0	4.034825	0.009122	0.034128

47	6	0	2.886747	0.408381	0.647104
48	6	0	2.920512	1.428601	1.634219
49	6	0	5.286436	0.568079	0.333820
50	6	0	4.135776	2.007815	1.959414
51	6	0	5.300284	1.597256	1.326073
52	6	0	1.648543	-2.111123	-1.509226
53	7	0	4.452974	-1.689342	-1.669255
54	6	0	2.455528	-2.841331	-2.363722
55	6	0	3.832474	-2.602561	-2.412825
56	6	0	1.633884	1.682094	2.144100
57	7	0	0.495116	1.656135	2.349360
58	6	0	6.565071	2.172250	1.622637
59	6	0	6.422623	0.149220	-0.302906
60	6	0	7.699237	1.775712	1.000547
61	6	0	7.681762	0.736690	0.005225
62	7	0	8.801330	0.337544	-0.604146
63	1	0	0.581036	-2.279806	-1.452683
64	1	0	2.031425	-3.603673	-3.000426
65	1	0	4.458002	-3.176597	-3.082977
66	1	0	4.181364	2.786990	2.708967
67	1	0	6.612755	2.944581	2.379368
68	1	0	8.627416	2.235124	1.293523
69	1	0	6.326828	-0.605753	-1.059905
70	6	0	10.092133	1.031894	-0.517784
71	6	0	11.170608	0.088015	-0.006886
72	1	0	10.353258	1.347704	-1.530623
73	1	0	10.019257	1.915880	0.100820
74	7	0	11.246850	-1.041553	-0.912527
75	1	0	12.123988	0.613942	0.000641
76	1	0	10.928078	-0.203518	1.026292
77	6	0	9.994709	-1.768151	-0.954633
78	1	0	12.021306	-1.649481	-0.685697
79	6	0	8.893639	-0.845041	-1.467949
80	1	0	10.085872	-2.611214	-1.638017
81	1	0	9.689855	-2.154597	0.029834
82	1	0	9.135052	-0.513950	-2.480306
83	1	0	7.954491	-1.383177	-1.485801

Ligand	Description	Total Gibbs Free energy (M06-2X/Def2TZVPP)
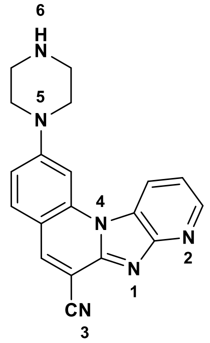<br>4b	2:1 complex with H^+ bound to N1	-2124.109825 a.u.

CARTESIAN COORDINATES

Center Number	Atomic Number	Atomic Type	Coordinates (Angstroms) X	Y	Z
1	7	0	3.483178	0.412008	0.341209
2	6	0	4.504159	-0.424907	0.794294
3	6	0	2.146631	0.143056	0.580442
4	7	0	1.340323	1.044721	0.075132
5	6	0	2.149812	1.961666	-0.543393
6	6	0	3.505373	1.600733	-0.391788
7	6	0	1.780338	-1.028565	1.309292
8	6	0	4.478091	2.423752	-0.945716
9	7	0	1.710927	3.029321	-1.194162
10	6	0	4.020367	3.542125	-1.619761
11	6	0	2.649456	3.799472	-1.721002
12	1	0	4.723752	4.223759	-2.074630
13	1	0	2.306447	4.675878	-2.257044
14	6	0	4.122348	-1.577048	1.515694
15	6	0	2.759410	-1.859573	1.760396
16	1	0	2.498172	-2.754656	2.309430
17	6	0	5.838391	-0.157570	0.536732
18	6	0	5.149803	-2.429565	1.966455
19	6	0	6.464757	-2.170215	1.725273
20	6	0	6.854763	-1.009642	0.999745
21	1	0	4.873948	-3.309880	2.532792
22	1	0	7.209712	-2.836842	2.127784
23	7	0	8.173874	-0.722271	0.803416
24	1	0	6.094094	0.692962	-0.059969
25	6	0	0.389767	-1.275684	1.503966
26	7	0	-0.740103	-1.443054	1.628157
27	6	0	9.182037	-1.771849	0.652395
28	6	0	9.411121	-2.041723	-0.832015
29	1	0	10.109249	-1.420854	1.107699
30	1	0	8.876903	-2.680850	1.156819
31	7	0	9.819254	-0.806431	-1.483153
32	1	0	10.199848	-2.784080	-0.950112
33	1	0	8.481421	-2.456423	-1.252589
34	6	0	8.815835	0.239555	-1.354625
35	1	0	10.052724	-0.969053	-2.452562
36	6	0	8.605764	0.498039	0.132370
37	1	0	9.177937	1.143994	-1.842748
38	1	0	7.851693	-0.038365	-1.809976
39	1	0	9.563014	0.784941	0.571307
40	1	0	7.905212	1.305841	0.323243
41	1	0	-0.284187	1.089613	-0.034668
42	7	0	-1.362257	1.108388	-0.070862
43	6	0	-2.157208	2.025267	0.575301
44	6	0	-3.497063	1.652138	0.390050
45	7	0	-3.463045	0.468520	-0.365029
46	6	0	-2.145448	0.189017	-0.619201

47	6	0	-1.769682	-0.940393	-1.385842
48	6	0	-4.493257	-0.363493	-0.815353
49	6	0	-2.764568	-1.745749	-1.867067
50	6	0	-4.117555	-1.479888	-1.595182
51	7	0	-1.720738	3.074158	1.240678
52	6	0	-4.480660	2.477282	0.921676
53	6	0	-2.669221	3.839876	1.760497
54	6	0	-4.036522	3.587215	1.618857
55	6	0	-0.388904	-1.209668	-1.619610
56	7	0	0.722067	-1.431447	-1.803199
57	6	0	-5.151077	-2.323941	-2.058136
58	6	0	-5.817286	-0.128431	-0.493560
59	6	0	-6.457323	-2.091295	-1.765042
60	6	0	-6.839615	-0.974315	-0.963430
61	7	0	-8.146564	-0.725493	-0.697592
62	1	0	-2.333491	4.706675	2.315232
63	1	0	-4.750507	4.268271	2.057457
64	1	0	-2.505751	-2.614759	-2.457550
65	1	0	-4.882361	-3.173527	-2.672359
66	1	0	-7.206857	-2.747235	-2.175986
67	1	0	-6.061866	0.671213	0.173534
68	6	0	-9.157483	-1.781677	-0.622657
69	6	0	-9.412058	-2.126607	0.842372
70	1	0	-10.076069	-1.407621	-1.076903
71	1	0	-8.840973	-2.664476	-1.164472
72	7	0	-9.829135	-0.926955	1.549451
73	1	0	-10.203877	-2.872073	0.905905
74	1	0	-8.490891	-2.566019	1.256195
75	6	0	-8.822507	0.120645	1.495875
76	1	0	-10.088093	-1.138088	2.502833
77	6	0	-8.589304	0.459640	0.028861
78	1	0	-9.190590	0.998560	2.025757
79	1	0	-7.865431	-0.183271	1.949657
80	1	0	-9.539717	0.768993	-0.409762
81	1	0	-7.886661	1.275998	-0.107688
82	1	0	-5.538184	2.310672	0.811614
83	1	0	5.538000	2.244363	-0.879527

Ligand	Description	Total Gibbs Free energy (M06-2X/Def2TZVPP)
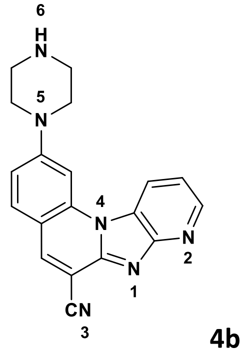<br>4b	2:1 complex with Ca^{2+} bound to N1	-2801.007224 a.u.

CARTESIAN COORDINATES

Center Number	Atomic Number	Atomic Type	Coordinates (Angstroms) X	Y	Z
1	7	0	-4.461457	0.032371	0.195627
2	6	0	-5.720861	-0.497680	0.502929
3	6	0	-3.332711	-0.378625	0.856587
4	7	0	-2.231631	0.224732	0.440903
5	6	0	-2.644778	1.087597	-0.545319
6	6	0	-4.035581	1.000006	-0.729482
7	6	0	-3.403170	-1.354253	1.887305
8	6	0	-4.622617	1.818840	-1.685118
9	7	0	-1.817929	1.881800	-1.203301
10	6	0	-3.763588	2.654941	-2.380684
11	6	0	-2.390879	2.657316	-2.116691
12	1	0	-4.155323	3.317495	-3.137940
13	1	0	-1.735984	3.317701	-2.669748
14	6	0	-5.774200	-1.478386	1.534566
15	6	0	-4.624363	-1.891625	2.215206
16	1	0	-4.706785	-2.637406	2.994907
17	6	0	-6.853904	-0.105571	-0.171090
18	6	0	-7.047145	-2.026569	1.831043
19	6	0	-8.169823	-1.647925	1.172936
20	6	0	-8.122200	-0.652551	0.144066
21	1	0	-7.114286	-2.766481	2.618104
22	1	0	-9.108265	-2.085861	1.466671
23	7	0	-9.238141	-0.243282	-0.488073
24	1	0	-6.764681	0.587034	-0.982759
25	6	0	-2.120832	-1.606180	2.429938
26	7	0	-0.993577	-1.606535	2.673084
27	6	0	-10.439795	-1.085545	-0.594392
28	6	0	-11.707426	-0.250898	-0.585434
29	1	0	-10.381900	-1.629303	-1.542325
30	1	0	-10.468923	-1.806790	0.211655
31	7	0	-11.628184	0.713800	-1.664209
32	1	0	-12.556404	-0.913319	-0.746262
33	1	0	-11.820360	0.222712	0.400891
34	6	0	-10.531256	1.628014	-1.431516
35	1	0	-12.501030	1.208652	-1.784117
36	6	0	-9.215496	0.860970	-1.450136
37	1	0	-10.500642	2.376582	-2.221922
38	1	0	-10.613547	2.150937	-0.466890
39	1	0	-9.029386	0.467900	-2.455256
40	1	0	-8.423769	1.553896	-1.179609
41	20	0	0.042170	0.060990	1.176653
42	7	0	2.299313	-0.147150	0.403582
43	6	0	2.700852	-1.089945	-0.511564
44	6	0	4.081397	-0.986403	-0.755957
45	7	0	4.514409	0.070300	0.062368
46	6	0	3.397204	0.520334	0.718393
47	6	0	3.471789	1.605152	1.632013

48	6	0	5.770519	0.654202	0.273634
49	6	0	4.688857	2.205705	1.855816
50	6	0	5.827364	1.743317	1.192738
51	7	0	1.871827	-1.956832	-1.066679
52	6	0	4.651122	-1.861677	-1.670710
53	6	0	2.430241	-2.792591	-1.935238
54	6	0	3.789602	-2.774735	-2.258908
55	6	0	2.200397	1.887626	2.182547
56	7	0	1.080557	1.890928	2.459043
57	6	0	7.100285	2.329005	1.409732
58	6	0	6.897744	0.192000	-0.360523
59	6	0	8.218539	1.884868	0.787829
60	6	0	8.166179	0.788886	-0.136077
61	7	0	9.267170	0.360285	-0.768755
62	1	0	1.773074	-3.513924	-2.402888
63	1	0	4.168971	-3.484424	-2.978820
64	1	0	4.775316	3.033331	2.547496
65	1	0	7.167089	3.167706	2.090634
66	1	0	9.146664	2.397018	0.974705
67	1	0	6.820048	-0.663518	-0.999387
68	6	0	10.639115	0.694799	-0.370805
69	6	0	11.361191	-0.579040	0.059828
70	1	0	11.148243	1.133881	-1.230527
71	1	0	10.646328	1.408392	0.442231
72	7	0	11.334705	-1.535024	-1.030228
73	1	0	12.394573	-0.332666	0.299163
74	1	0	10.877052	-0.957583	0.973041
75	6	0	9.980323	-1.896410	-1.399472
76	1	0	11.896544	-2.348856	-0.823933
77	6	0	9.268072	-0.627321	-1.848922
78	1	0	10.007636	-2.606161	-2.225193
79	1	0	9.412283	-2.348295	-0.570695
80	1	0	9.825719	-0.191921	-2.680916
81	1	0	8.261208	-0.825656	-2.196116
82	1	0	5.693907	-1.865572	-1.940926
83	1	0	-5.677207	1.839068	-1.904288

Ligand	Description	Total Gibbs Free energy (M06-2X/Def2TZVPP)
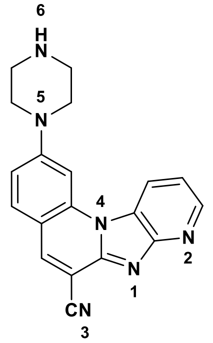<br>**4b**	2:1 complex with Cu^{2+} bound to N1	-3763.748040 a.u.

CARTESIAN COORDINATES

Center Number	Atomic Number	Atomic Type	X	Y	Z
1	7	0	-3.963954	0.286790	-0.045098
2	6	0	-4.877617	-0.734884	-0.320971
3	6	0	-2.601737	0.094561	-0.126885
4	7	0	-1.919352	1.187725	0.158146
5	6	0	-2.852187	2.152814	0.453910
6	6	0	-4.147912	1.620841	0.341056
7	6	0	-2.079424	-1.183314	-0.478966
8	6	0	-5.226680	2.447898	0.633462
9	7	0	-2.550149	3.390368	0.805549
10	6	0	-4.917827	3.745619	1.000570
11	6	0	-3.586927	4.171448	1.070231
12	1	0	-5.709172	4.440137	1.240253
13	1	0	-3.361049	5.190110	1.358484
14	6	0	-4.343298	-2.001700	-0.660634
15	6	0	-2.955470	-2.204840	-0.731230
16	1	0	-2.579647	-3.183502	-0.999639
17	6	0	-6.241322	-0.522142	-0.290467
18	6	0	-5.262679	-3.035914	-0.949140
19	6	0	-6.606101	-2.839787	-0.911367
20	6	0	-7.150158	-1.564709	-0.567688
21	1	0	-4.872160	-4.013519	-1.200604
22	1	0	-7.254949	-3.674627	-1.115035
23	7	0	-8.489186	-1.367777	-0.492887
24	1	0	-6.613592	0.464250	-0.108682
25	6	0	-0.664358	-1.322522	-0.549192
26	7	0	0.484957	-1.367395	-0.583595
27	6	0	-9.425266	-2.150283	-1.312232
28	6	0	-10.789520	-2.252461	-0.655739
29	1	0	-9.534616	-1.649885	-2.280136
30	1	0	-9.032461	-3.144679	-1.483655
31	7	0	-11.279721	-0.912833	-0.397106
32	1	0	-11.463799	-2.764690	-1.340298
33	1	0	-10.703064	-2.855086	0.260498
34	6	0	-10.415967	-0.250121	0.556889
35	1	0	-12.237896	-0.926396	-0.076332
36	6	0	-9.027126	-0.084446	-0.042440
37	1	0	-10.810084	0.738842	0.786521
38	1	0	-10.322293	-0.809657	1.499645
39	1	0	-9.067735	0.619507	-0.881996
40	1	0	-8.376803	0.316655	0.731525
41	29	0	0.025572	1.425540	0.287064
42	7	0	2.020054	1.388477	0.295811
43	6	0	2.800164	2.140350	-0.547410
44	6	0	4.128200	1.678607	-0.521697
45	7	0	4.119338	0.597036	0.362350
46	6	0	2.817251	0.491966	0.821345

47	6	0	2.479133	-0.501889	1.790883
48	6	0	5.115455	-0.285496	0.771875
49	6	0	3.442403	-1.358903	2.237201
50	6	0	4.758035	-1.276874	1.733557
51	7	0	2.346353	3.151754	-1.270522
52	6	0	5.077319	2.349186	-1.289684
53	6	0	3.249188	3.770181	-2.006933
54	6	0	4.608847	3.410040	-2.037572
55	6	0	1.140773	-0.528101	2.286588
56	7	0	0.062056	-0.515614	2.678035
57	6	0	5.758599	-2.179370	2.168268
58	6	0	6.392022	-0.238169	0.266305
59	6	0	7.023004	-2.140334	1.679840
60	6	0	7.385521	-1.161313	0.697258
61	7	0	8.635697	-1.107962	0.208433
62	1	0	2.899157	4.600833	-2.606339
63	1	0	5.289728	3.978089	-2.654301
64	1	0	3.200382	-2.110085	2.976336
65	1	0	5.496385	-2.910164	2.921510
66	1	0	7.756778	-2.820525	2.077759
67	1	0	6.620207	0.463758	-0.510595
68	6	0	9.589439	-2.221500	0.276009
69	6	0	9.802225	-2.747875	-1.152862
70	1	0	10.529758	-1.836968	0.669529
71	1	0	9.221455	-3.014832	0.911496
72	7	0	10.283123	-1.666961	-1.976570
73	1	0	10.544536	-3.542852	-1.114431
74	1	0	8.850725	-3.177403	-1.500077
75	6	0	9.350710	-0.571034	-2.064961
76	1	0	10.627565	-1.973677	-2.875022
77	6	0	9.139975	-0.030601	-0.645983
78	1	0	9.768596	0.221649	-2.682735
79	1	0	8.370531	-0.856698	-2.477770
80	1	0	10.106818	0.264710	-0.237248
81	1	0	8.482202	0.828539	-0.616830
82	1	0	6.125939	2.103327	-1.320822
83	1	0	-6.257693	2.139333	0.599185

Ligand	Description	Total Gibbs Free energy (M06-2X/Def2TZVPP)
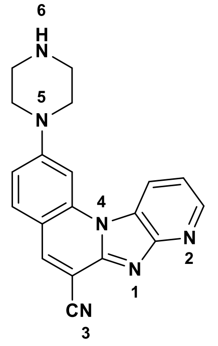<br>**4b**	2:1 complex with Mg^{2+} bound to N1	-2323.442677 a.u.

CARTESIAN COORDINATES

Center Number	Atomic Number	Atomic Type	Coordinates (Angstroms) X	Y	Z
1	7	0	-3.999983	0.101604	0.288473
2	6	0	-5.263344	-0.460840	0.516411
3	6	0	-2.906930	-0.284474	1.014110
4	7	0	-1.797943	0.354998	0.667879
5	6	0	-2.171317	1.218016	-0.340701
6	6	0	-3.545480	1.088479	-0.603961
7	6	0	-3.017071	-1.268369	2.033960
8	6	0	-4.104977	1.895309	-1.586142
9	7	0	-1.337257	2.040763	-0.947065
10	6	0	-3.236926	2.762945	-2.228091
11	6	0	-1.882303	2.804254	-1.886418
12	1	0	-3.606933	3.418779	-3.001986
13	1	0	-1.217370	3.488306	-2.397333
14	6	0	-5.356137	-1.449402	1.539945
15	6	0	-4.243126	-1.838571	2.286084
16	1	0	-4.352808	-2.590274	3.056565
17	6	0	-6.362171	-0.092729	-0.222077
18	6	0	-6.632303	-2.029137	1.757584
19	6	0	-7.721461	-1.673519	1.035504
20	6	0	-7.635463	-0.670987	0.014522
21	1	0	-6.728532	-2.773950	2.536946
22	1	0	-8.665587	-2.133375	1.271808
23	7	0	-8.718488	-0.285612	-0.681122
24	1	0	-6.241975	0.604193	-1.025489
25	6	0	-1.758143	-1.473187	2.639267
26	7	0	-0.637046	-1.391605	2.901948
27	6	0	-9.907486	-1.136710	-0.844826
28	6	0	-11.183576	-0.314362	-0.842142
29	1	0	-9.817975	-1.644687	-1.809738
30	1	0	-9.947351	-1.887717	-0.067130
31	7	0	-11.087566	0.679608	-1.892527
32	1	0	-12.022249	-0.979501	-1.041235
33	1	0	-11.324008	0.130451	0.154029
34	6	0	-10.002098	1.594895	-1.615351
35	1	0	-11.961190	1.171140	-2.020136
36	6	0	-8.680698	0.836161	-1.623099
37	1	0	-9.959444	2.361234	-2.387977
38	1	0	-10.107656	2.095520	-0.641188
39	1	0	-8.474809	0.459548	-2.630153
40	1	0	-7.898197	1.528420	-1.327123
41	12	0	0.044369	0.117469	1.540458
42	7	0	1.868295	-0.229675	0.669564
43	6	0	2.226744	-1.218180	-0.222580
44	6	0	3.585192	-1.094842	-0.560241
45	7	0	4.047803	0.013126	0.171363
46	6	0	2.970816	0.478953	0.874466

47	6	0	3.084563	1.616184	1.718486
48	6	0	5.304679	0.627022	0.267722
49	6	0	4.300288	2.254337	1.822216
50	6	0	5.398880	1.774032	1.112333
51	7	0	1.390982	-2.130101	-0.680555
52	6	0	4.120228	-1.998980	-1.468027
53	6	0	1.915902	-2.994568	-1.540812
54	6	0	3.250261	-2.961135	-1.954316
55	6	0	1.841441	1.886028	2.329014
56	7	0	0.732195	1.822107	2.643112
57	6	0	6.671454	2.394358	1.207724
58	6	0	6.398927	0.141134	-0.403280
59	6	0	7.756033	1.928856	0.545195
60	6	0	7.668488	0.770744	-0.298467
61	7	0	8.736002	0.315327	-0.965445
62	1	0	1.249490	-3.754173	-1.928026
63	1	0	3.602419	-3.695851	-2.662904
64	1	0	4.412951	3.123489	2.456695
65	1	0	6.763896	3.276626	1.827820
66	1	0	8.683274	2.467168	0.638957
67	1	0	6.299537	-0.757091	-0.976248
68	6	0	10.121845	0.715755	-0.693952
69	6	0	10.915524	-0.502727	-0.230520
70	1	0	10.552247	1.105725	-1.617928
71	1	0	10.164122	1.485791	0.064439
72	7	0	10.844129	-1.533702	-1.247307
73	1	0	11.954003	-0.208962	-0.085496
74	1	0	10.511364	-0.829156	0.739865
75	6	0	9.480037	-1.959764	-1.488316
76	1	0	11.447717	-2.313773	-1.028801
77	6	0	8.693437	-0.748125	-1.971304
78	1	0	9.471193	-2.725555	-2.262720
79	1	0	8.989061	-2.367446	-0.590422
80	1	0	9.173468	-0.357196	-2.870990
81	1	0	7.672160	-1.002702	-2.226918
82	1	0	5.142190	-1.987699	-1.807408
83	1	0	-5.145697	1.884090	-1.863023

Ligand	Description	Total Gibbs Free energy (M06-2X/Def2TZVPP)
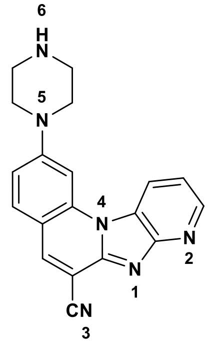 **4b**	2:1 complex with Zn^{2+} bound to N1	-3902.698440 a.u.

CARTESIAN COORDINATES

Center Number	Atomic Number	Atomic Type	Coordinates (Angstroms) X	Y	Z
1	7	0	-4.021835	0.071177	0.144756
2	6	0	-5.268512	-0.472370	0.484596
3	6	0	-2.882642	-0.296999	0.803592
4	7	0	-1.803494	0.323221	0.343875
5	6	0	-2.241217	1.151304	-0.667179
6	6	0	-3.631231	1.019731	-0.818027
7	6	0	-2.919293	-1.237589	1.866393
8	6	0	-4.259862	1.792898	-1.785568
9	7	0	-1.451287	1.942673	-1.364161
10	6	0	-3.439244	2.628603	-2.524838
11	6	0	-2.062326	2.673350	-2.289083
12	1	0	-3.864347	3.256918	-3.293237
13	1	0	-1.434652	3.331728	-2.875192
14	6	0	-5.291140	-1.418589	1.550271
15	6	0	-4.127874	-1.785962	2.228089
16	1	0	-4.184515	-2.506438	3.033335
17	6	0	-6.415818	-0.126225	-0.188200
18	6	0	-6.550287	-1.981830	1.881005
19	6	0	-7.686835	-1.648293	1.224313
20	6	0	-7.670536	-0.686670	0.161391
21	1	0	-6.592986	-2.695000	2.693974
22	1	0	-8.613559	-2.092877	1.544329
23	7	0	-8.799217	-0.320971	-0.469778
24	1	0	-6.351597	0.535958	-1.026552
25	6	0	-1.623438	-1.442840	2.392690
26	7	0	-0.486022	-1.381964	2.583278
27	6	0	-9.995310	-1.176257	-0.525219
28	6	0	-11.269918	-0.353275	-0.473787
29	1	0	-9.966204	-1.721392	-1.473453
30	1	0	-9.984770	-1.896364	0.282173
31	7	0	-11.240768	0.600968	-1.564323
32	1	0	-12.118345	-1.024624	-0.595473
33	1	0	-11.348702	0.128625	0.511844
34	6	0	-10.142266	1.525488	-1.388025
35	1	0	-12.121650	1.087538	-1.655988
36	6	0	-8.822436	0.766911	-1.451157
37	1	0	-10.149491	2.263902	-2.188467
38	1	0	-10.188157	2.060258	-0.427470
39	1	0	-8.678014	0.355938	-2.455540
40	1	0	-8.024511	1.468907	-1.227536
41	30	0	0.042742	0.124573	1.069032
42	7	0	1.867701	-0.191479	0.333862
43	6	0	2.279280	-1.162715	-0.553034
44	6	0	3.659368	-1.039331	-0.782471
45	7	0	4.073169	0.046673	0.010494
46	6	0	2.952841	0.506758	0.644375

47	6	0	3.008806	1.619326	1.523274
48	6	0	5.325161	0.644550	0.215869
49	6	0	4.220152	2.239493	1.733961
50	6	0	5.365289	1.766723	1.096313
51	7	0	1.474513	-2.055769	-1.091790
52	6	0	4.255242	-1.925373	-1.670388
53	6	0	2.057036	-2.903235	-1.931888
54	6	0	3.418666	-2.868866	-2.244005
55	6	0	1.729111	1.900815	2.051443
56	7	0	0.599788	1.864273	2.291718
57	6	0	6.632267	2.370785	1.304258
58	6	0	6.462156	0.165575	-0.385785
59	6	0	7.759256	1.912185	0.711469
60	6	0	7.725224	0.779313	-0.169414
61	7	0	8.835456	0.333183	-0.769725
62	1	0	1.416712	-3.648107	-2.385833
63	1	0	3.819066	-3.588279	-2.942608
64	1	0	4.292034	3.090600	2.398127
65	1	0	6.685346	3.234309	1.954581
66	1	0	8.681673	2.437211	0.890019
67	1	0	6.398993	-0.715184	-0.990087
68	6	0	10.200927	0.700560	-0.375827
69	6	0	10.923254	-0.540432	0.141822
70	1	0	10.717312	1.086666	-1.256125
71	1	0	10.195350	1.465134	0.389183
72	7	0	10.917996	-1.559640	-0.889476
73	1	0	11.951557	-0.273512	0.380841
74	1	0	10.427637	-0.866142	1.069157
75	6	0	9.571897	-1.950456	-1.258235
76	1	0	11.481324	-2.356282	-0.627621
77	6	0	8.859608	-0.714327	-1.792532
78	1	0	9.616273	-2.706790	-2.040690
79	1	0	8.992569	-2.356966	-0.413954
80	1	0	9.428189	-0.322499	-2.638378
81	1	0	7.860049	-0.939472	-2.144175
82	1	0	5.299628	-1.913435	-1.932374
83	1	0	-5.318622	1.779634	-1.981659

Ligand	Description	Total Gibbs Free energy (M06-2X/Def2TZVPP)
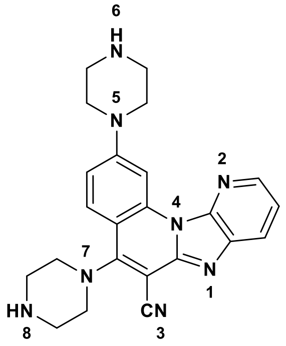<br>**5a**	2:1 complex with H^+ bound to N1	-2657.338342 a.u.

CARTESIAN COORDINATES

Center Number	Atomic Number	Atomic Type	Coordinates (Angstroms) X	Y	Z
1	7	0	-3.564619	-1.142578	0.204802
2	6	0	-4.721894	-0.404747	0.475814
3	6	0	-2.326691	-0.649547	0.487089
4	7	0	-1.390291	-1.531538	0.123394
5	6	0	-2.012938	-2.621792	-0.438775
6	6	0	-3.393047	-2.403462	-0.393958
7	6	0	-2.140849	0.584824	1.129520
8	7	0	-4.315542	-3.228831	-0.834037
9	6	0	-1.533542	-3.791556	-0.999573
10	6	0	-3.850296	-4.360106	-1.365934
11	6	0	-2.495162	-4.672983	-1.469470
12	1	0	-4.596221	-5.052591	-1.732252
13	1	0	-2.203348	-5.608507	-1.923435
14	1	0	-0.474444	-3.996172	-1.072954
15	6	0	-4.561088	0.864783	1.072985
16	6	0	-3.263710	1.358675	1.469368
17	7	0	-3.139899	2.531824	2.135150
18	6	0	-5.955199	-0.898577	0.095496
19	6	0	-5.727812	1.655541	1.125257
20	6	0	-6.952791	1.194896	0.741206
21	6	0	-7.122467	-0.134273	0.263881
22	1	0	-5.653509	2.679399	1.455116
23	1	0	-7.791146	1.867026	0.814684
24	7	0	-8.350726	-0.642812	-0.018966
25	1	0	-5.981503	-1.864949	-0.370657
26	6	0	-0.814072	0.876272	1.544387
27	7	0	0.271194	1.071129	1.870528
28	6	0	-9.499181	0.201378	-0.343276
29	6	0	-9.615632	0.329841	-1.858780
30	1	0	-10.392998	-0.286569	0.047829
31	1	0	-9.418744	1.174866	0.124586
32	7	0	-9.753339	-1.000326	-2.431534
33	1	0	-10.496555	0.919380	-2.110882
34	1	0	-8.725614	0.863481	-2.228436
35	6	0	-8.620702	-1.859361	-2.116951
36	1	0	-9.905223	-0.950324	-3.429080
37	6	0	-8.521945	-1.971791	-0.600572
38	1	0	-8.788429	-2.844080	-2.551875
39	1	0	-7.666633	-1.468380	-2.503986
40	1	0	-9.460686	-2.373949	-0.216410
41	1	0	-7.724241	-2.637176	-0.287997
42	6	0	-4.035933	2.943792	3.219344
43	6	0	-3.213492	3.223004	4.471927
44	1	0	-4.569496	3.856629	2.938750
45	1	0	-4.751100	2.150839	3.417585
46	7	0	-2.232946	4.252222	4.179722

47	1	0	-3.884029	3.572930	5.256349
48	1	0	-2.754345	2.279570	4.804524
49	6	0	-1.282294	3.791394	3.177645
50	1	0	-1.755964	4.544920	5.021035
51	6	0	-2.053147	3.488506	1.902838
52	1	0	-0.560942	4.582143	2.974114
53	1	0	-0.733405	2.895385	3.492381
54	1	0	-2.525068	4.414935	1.560681
55	1	0	-1.406071	3.128479	1.109409
56	1	0	-0.330610	-1.411325	0.172319
57	7	0	1.329711	-1.543580	0.171305
58	6	0	2.049835	-2.563743	0.748200
59	6	0	3.426961	-2.294742	0.671319
60	7	0	3.519728	-1.060044	0.023549
61	6	0	2.229000	-0.666420	-0.237735
62	6	0	1.983317	0.548411	-0.936481
63	6	0	4.619962	-0.259946	-0.283686
64	6	0	3.041869	1.369832	-1.317217
65	6	0	4.379329	0.971965	-0.922654
66	6	0	1.654987	-3.741360	1.366925
67	7	0	4.400144	-3.056525	1.124203
68	6	0	2.663906	-4.559147	1.849974
69	6	0	4.001336	-4.186063	1.707959
70	6	0	0.636205	0.751707	-1.333490
71	7	0	-0.468447	0.867728	-1.635694
72	6	0	5.493034	1.826862	-1.042285
73	6	0	5.896038	-0.669152	0.078798
74	6	0	6.750868	1.452413	-0.665217
75	6	0	6.997791	0.168300	-0.117687
76	7	0	8.262957	-0.203468	0.267997
77	7	0	2.850080	2.521574	-2.020119
78	1	0	0.610366	-4.001809	1.476847
79	1	0	2.427148	-5.489903	2.344647
80	1	0	4.786457	-4.827652	2.085835
81	1	0	6.001419	-1.645275	0.514839
82	1	0	7.548529	2.173792	-0.742981
83	1	0	5.346499	2.832114	-1.404376
84	6	0	3.684444	2.912177	-3.157373
85	6	0	2.810360	3.087154	-4.393106
86	1	0	4.185136	3.862451	-2.947564
87	1	0	4.430995	2.144280	-3.338588
88	7	0	1.792331	4.085890	-4.117841
89	1	0	3.435190	3.426099	-5.219162
90	1	0	2.383114	2.108460	-4.660216
91	6	0	0.899210	3.634002	-3.059458
92	1	0	1.272018	4.307655	-4.955177
93	6	0	1.725357	3.429707	-1.798444
94	1	0	0.148686	4.401510	-2.870881
95	1	0	0.383424	2.699466	-3.311930
96	1	0	2.151633	4.396912	-1.512062
97	1	0	1.120532	3.070477	-0.971013
98	6	0	9.410624	0.224272	-0.535658
99	6	0	10.696058	0.149968	0.266770
100	1	0	9.501709	-0.429903	-1.411603
101	1	0	9.262657	1.240458	-0.884109
102	7	0	10.862553	-1.205945	0.757848
103	1	0	11.531753	0.407050	-0.383104
104	1	0	10.648581	0.889095	1.080527
105	6	0	9.773926	-1.544411	1.655902
106	1	0	11.756149	-1.317507	1.216473
107	6	0	8.455498	-1.504109	0.901092
108	1	0	9.922215	-2.550966	2.045141
109	1	0	9.705917	-0.852006	2.508347
110	1	0	8.434409	-2.303995	0.149671
111	1	0	7.649413	-1.674100	1.611388

Ligand	Description	Total Gibbs Free energy (M06-2X/Def2TZVPP)
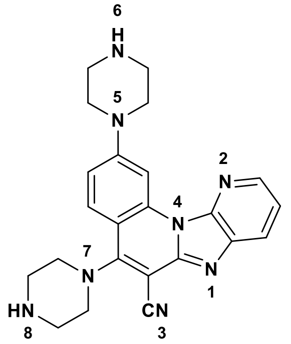 5a	2:1 complex with Ca^{2+} bound to N1	-3334.233785 a.u.

CARTESIAN COORDINATES

Center Number	Atomic Number	Atomic Type	X	Y	Z
1	7	0	4.247726	0.699591	-0.128418
2	6	0	5.516893	0.133248	-0.322585
3	6	0	3.212838	-0.009681	0.398204
4	7	0	2.076713	0.691077	0.466904
5	6	0	2.395866	1.938393	-0.055912
6	6	0	3.741380	1.969954	-0.441399
7	6	0	3.416635	-1.337778	0.873637
8	7	0	4.378211	2.987747	-0.975302
9	6	0	1.642038	3.087607	-0.235532
10	6	0	3.640237	4.083572	-1.147703
11	6	0	2.292701	4.174794	-0.796100
12	1	0	4.146275	4.933736	-1.585009
13	1	0	1.766634	5.102870	-0.964415
14	1	0	0.600353	3.141365	0.054661
15	6	0	5.725923	-1.189842	0.137386
16	6	0	4.707296	-1.924651	0.834997
17	7	0	4.968380	-3.112131	1.411984
18	6	0	6.492879	0.855428	-0.973512
19	6	0	6.951666	-1.777065	-0.252882
20	6	0	7.920632	-1.090816	-0.917104
21	6	0	7.747106	0.280269	-1.269694
22	1	0	7.113852	-2.826906	-0.062856
23	1	0	8.806186	-1.625684	-1.216359
24	7	0	8.712332	0.976867	-1.908420
25	1	0	6.266042	1.870765	-1.237765
26	6	0	2.275478	-1.803803	1.532860
27	7	0	1.231428	-1.984976	2.004217
28	6	0	10.130230	0.609281	-1.807846
29	6	0	10.888818	0.977480	-3.070245
30	1	0	10.564702	1.153954	-0.963152
31	1	0	10.233150	-0.451891	-1.619122
32	7	0	10.717360	2.395944	-3.314928
33	1	0	11.944023	0.756496	-2.916856
34	1	0	10.522669	0.356625	-3.901564
35	6	0	9.321482	2.690605	-3.564437
36	1	0	11.300971	2.711208	-4.077228
37	6	0	8.501143	2.365163	-2.324976
38	1	0	9.203536	3.750581	-3.784888
39	1	0	8.914903	2.116512	-4.410683
40	1	0	8.781133	3.038425	-1.507889
41	1	0	7.453398	2.516852	-2.564614
42	6	0	6.215169	-3.407182	2.138146
43	6	0	5.889427	-3.687809	3.601884
44	1	0	6.694928	-4.282511	1.694785
45	1	0	6.881797	-2.554185	2.076351

46	7	0	4.937879	-4.777142	3.692426
47	1	0	6.811098	-3.966471	4.111025
48	1	0	5.514687	-2.757457	4.056848
49	6	0	3.679729	-4.427425	3.062488
50	1	0	4.807496	-5.077632	4.648059
51	6	0	3.965105	-4.164372	1.591367
52	1	0	2.982561	-5.259989	3.148014
53	1	0	3.211092	-3.538209	3.511591
54	1	0	4.391146	-5.074606	1.161815
55	1	0	3.069617	-3.926598	1.024878
56	20	0	-0.000036	0.000774	1.467671
57	7	0	-2.076399	-0.690009	0.466444
58	6	0	-2.395268	-1.937584	-0.055923
59	6	0	-3.740833	-1.969657	-0.441201
60	7	0	-4.247496	-0.699337	-0.128562
61	6	0	-3.212741	0.010400	0.397692
62	6	0	-3.416847	1.338596	0.872701
63	6	0	-5.516847	-0.133408	-0.322770
64	6	0	-4.707665	1.925126	0.833981
65	6	0	-5.726172	1.189793	0.136737
66	6	0	-1.641113	-3.086605	-0.235385
67	7	0	-4.377439	-2.987795	-0.974710
68	6	0	-2.291540	-4.174152	-0.795534
69	6	0	-3.639157	-4.083450	-1.146916
70	6	0	-2.275802	1.805084	1.531796
71	7	0	-1.231808	1.986626	2.003135
72	6	0	-6.952115	1.776549	-0.253614
73	6	0	-6.492710	-0.856084	-0.973321
74	6	0	-7.920973	1.089805	-0.917487
75	6	0	-7.747106	-0.281353	-1.269622
76	7	0	-8.712187	-0.978439	-1.908084
77	7	0	-4.969010	3.112749	1.410551
78	1	0	-0.599351	-3.139960	0.054612
79	1	0	-1.765204	-5.102102	-0.963707
80	1	0	-4.145012	-4.933893	-1.583892
81	1	0	-6.265647	-1.871454	-1.237228
82	1	0	-8.806718	1.624319	-1.216806
83	1	0	-7.114561	2.826412	-0.063941
84	6	0	-6.215831	3.407743	2.136688
85	6	0	-5.890067	3.689108	3.600277
86	1	0	-6.695883	4.282744	1.692997
87	1	0	-6.882209	2.554522	2.075304
88	7	0	-4.938863	4.778785	3.690260
89	1	0	-6.811791	3.967700	4.109362
90	1	0	-5.514996	2.759078	4.055628
91	6	0	-3.680643	4.429176	3.060402
92	1	0	-4.808517	5.079767	4.645743
93	6	0	-3.966018	4.165350	1.589418
94	1	0	-2.983741	5.262008	3.145483
95	1	0	-3.211691	3.540326	3.509897
96	1	0	-4.392325	5.075271	1.159463
97	1	0	-3.070489	3.927566	1.022997
98	6	0	-10.130159	-0.611042	-1.807759
99	6	0	-10.888621	-0.980140	-3.069967
100	1	0	-10.564571	-1.155282	-0.962752
101	1	0	-10.233282	0.450223	-1.619681
102	7	0	-10.716979	-2.398741	-3.313723
103	1	0	-11.943859	-0.759161	-2.916813
104	1	0	-10.522461	-0.359794	-3.901661
105	6	0	-9.321065	-2.693378	-3.562986
106	1	0	-11.300547	-2.714604	-4.075806
107	6	0	-8.500791	-2.366982	-2.323730
108	1	0	-9.202948	-3.753484	-3.782722
109	1	0	-8.914524	-2.119798	-4.409602
110	1	0	-8.780661	-3.039737	-1.506182
111	1	0	-7.453030	-2.518683	-2.563278

Ligand	Description	Total Gibbs Free energy (M06-2X/Def2TZVPP)
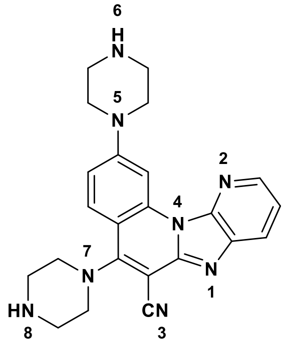 5a	2:1 complex with Cu^{2+} bound to N1	-4296.986085 a.u.

CARTESIAN COORDINATES

Center Number	Atomic Number	Atomic Type	Coordinates (Angstroms) X	Y	Z
1	7	0	4.155630	0.307394	0.594583
2	6	0	5.370642	-0.214190	0.175435
3	6	0	2.962739	-0.322540	0.335410
4	7	0	1.924113	0.360597	0.779975
5	6	0	2.443729	1.500961	1.358145
6	6	0	3.839630	1.495692	1.259285
7	6	0	2.956585	-1.607347	-0.280552
8	7	0	4.668515	2.417952	1.702536
9	6	0	1.836337	2.581377	1.987755
10	6	0	4.070750	3.445046	2.306166
11	6	0	2.684894	3.561308	2.466293
12	1	0	4.724202	4.219548	2.684961
13	1	0	2.288814	4.431699	2.968135
14	1	0	0.761662	2.649247	2.079606
15	6	0	5.366591	-1.457486	-0.508260
16	6	0	4.147968	-2.232257	-0.672116
17	7	0	4.194311	-3.472880	-1.206325
18	6	0	6.532475	0.495770	0.382032
19	6	0	6.587642	-1.848174	-1.108448
20	6	0	7.743096	-1.149066	-0.947185
21	6	0	7.765534	0.032823	-0.141766
22	1	0	6.601202	-2.724499	-1.736478
23	1	0	8.632989	-1.515700	-1.428898
24	7	0	8.906953	0.705053	0.099009
25	1	0	6.450023	1.430834	0.907854
26	6	0	1.685624	-2.242457	-0.307340
27	7	0	0.629349	-2.696349	-0.277032
28	6	0	10.154283	0.498437	-0.639797
29	6	0	10.373849	1.720978	-1.545039
30	1	0	10.964037	0.435157	0.086210
31	1	0	10.124337	-0.411213	-1.222517
32	7	0	10.422698	2.899209	-0.714525
33	1	0	11.320687	1.597618	-2.067431
34	1	0	9.564449	1.739104	-2.290353
35	6	0	9.204648	3.118993	0.027595
36	1	0	10.741190	3.719806	-1.209533
37	6	0	8.984260	1.903708	0.937276
38	1	0	9.305978	4.006819	0.648752
39	1	0	8.313814	3.237137	-0.608018
40	1	0	9.854251	1.783419	1.582686
41	1	0	8.103056	2.008785	1.556207
42	6	0	5.201941	-4.472306	-0.818336
43	6	0	4.502154	-5.700855	-0.246920
44	1	0	5.779781	-4.772815	-1.696359
45	1	0	5.865346	-4.047801	-0.069205

46	7	0	3.589780	-6.225969	-1.240852
47	1	0	5.257561	-6.449566	-0.011382
48	1	0	3.998716	-5.412729	0.689183
49	6	0	2.525341	-5.279394	-1.525332
50	1	0	3.222695	-7.126875	-0.968572
51	6	0	3.161888	-4.022447	-2.098128
52	1	0	1.851929	-5.700813	-2.270170
53	1	0	1.936453	-5.020971	-0.635489
54	1	0	3.673051	-4.291839	-3.026433
55	1	0	2.426537	-3.258928	-2.330302
56	29	0	-0.046373	-0.018876	0.644807
57	7	0	-1.969552	-0.490442	0.759984
58	6	0	-2.537325	-1.606697	1.342474
59	6	0	-3.932889	-1.540909	1.238804
60	7	0	-4.198983	-0.338865	0.570099
61	6	0	-2.988082	0.238008	0.308954
62	6	0	-2.914212	1.511332	-0.316662
63	6	0	-5.403730	0.241206	0.158206
64	6	0	-4.091279	2.185305	-0.691681
65	6	0	-5.337549	1.485038	-0.513244
66	6	0	-1.980358	-2.704582	1.977584
67	7	0	-4.791655	-2.430229	1.687974
68	6	0	-2.866549	-3.653156	2.460651
69	6	0	-4.242027	-3.480189	2.297427
70	6	0	-1.614835	2.065511	-0.313997
71	7	0	-0.511075	2.399280	-0.249685
72	6	0	-6.546240	1.916026	-1.102069
73	6	0	-6.590703	-0.432079	0.355755
74	6	0	-7.727823	1.257865	-0.936585
75	6	0	-7.808235	0.082106	-0.135299
76	7	0	-8.987658	-0.520500	0.135092
77	7	0	-4.065759	3.437194	-1.208571
78	1	0	-0.909291	-2.813057	2.080073
79	1	0	-2.504703	-4.535463	2.967818
80	1	0	-4.931023	-4.222893	2.677040
81	1	0	-6.545962	-1.379605	0.858322
82	1	0	-8.600906	1.656307	-1.424825
83	1	0	-6.538642	2.787908	-1.737168
84	6	0	-5.040530	4.476073	-0.856572
85	6	0	-4.308210	5.677049	-0.269058
86	1	0	-5.584345	4.794081	-1.750226
87	1	0	-5.740652	4.082506	-0.126353
88	7	0	-3.333475	6.162480	-1.227184
89	1	0	-5.036362	6.460763	-0.063249
90	1	0	-3.850828	5.372151	0.685159
91	6	0	-2.313547	5.160731	-1.486197
92	1	0	-2.925042	7.035412	-0.924505
93	6	0	-2.999347	3.938921	-2.077926
94	1	0	-1.597324	5.549978	-2.209031
95	1	0	-1.765685	4.866738	-0.579834
96	1	0	-3.475755	4.239081	-3.015993
97	1	0	-2.295737	3.143825	-2.302312
98	6	0	-10.204326	-0.310024	-0.647877
99	6	0	-10.396033	-1.489077	-1.597658
100	1	0	-11.045599	-0.263639	0.045184
101	1	0	-10.166300	0.623663	-1.194509
102	7	0	-10.466089	-2.712205	-0.816569
103	1	0	-11.326420	-1.363563	-2.150205
104	1	0	-9.563159	-1.488732	-2.318560
105	6	0	-9.263623	-2.937799	-0.031249
106	1	0	-10.679151	-3.507970	-1.401168
107	6	0	-9.088313	-1.753848	0.912224
108	1	0	-9.379600	-3.854303	0.545941
109	1	0	-8.357017	-3.030190	-0.650022
110	1	0	-9.977907	-1.669721	1.538705
111	1	0	-8.231248	-1.873632	1.565222

Ligand	Description	Total Gibbs Free energy (M06-2X/Def2TZVPP)
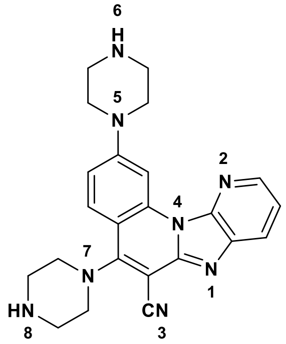 5a	2:1 complex with Mg^{2+} bound to N1	-2856.676492 a.u.

CARTESIAN COORDINATES

Center Number	Atomic Number	Atomic Type	Coordinates (Angstroms) X	Y	Z
1	7	0	3.843719	0.518632	-0.300748
2	6	0	5.197101	0.350444	0.039379
3	6	0	2.897861	-0.379978	0.071968
4	7	0	1.675948	-0.067645	-0.380319
5	6	0	1.841692	1.113757	-1.091221
6	6	0	3.186909	1.500486	-1.061733
7	6	0	3.222027	-1.473694	0.920533
8	7	0	3.702768	2.567862	-1.626862
9	6	0	0.943159	1.920525	-1.768813
10	6	0	2.829155	3.335106	-2.279379
11	6	0	1.465520	3.053915	-2.372169
12	1	0	3.234286	4.219752	-2.751919
13	1	0	0.824661	3.728981	-2.919854
14	1	0	-0.111123	1.680692	-1.824459
15	6	0	5.537465	-0.755329	0.861766
16	6	0	4.545813	-1.639489	1.403912
17	7	0	4.851678	-2.580999	2.312248
18	6	0	6.136039	1.213353	-0.472001
19	6	0	6.924151	-1.003211	0.983775
20	6	0	7.873668	-0.174921	0.470926
21	6	0	7.516537	1.023078	-0.221003
22	1	0	7.252635	-1.911883	1.465020
23	1	0	8.905176	-0.461102	0.583532
24	7	0	8.437055	1.908665	-0.635742
25	1	0	5.783853	2.018912	-1.087243
26	6	0	2.040625	-2.112696	1.308175
27	7	0	0.931384	-2.430337	1.426126
28	6	0	9.882923	1.688191	-0.571502
29	6	0	10.416267	1.432295	-1.976671
30	1	0	10.336921	2.598743	-0.177158
31	1	0	10.129410	0.875108	0.098725
32	7	0	10.091308	2.578490	-2.806757
33	1	0	11.498041	1.312777	-1.936684
34	1	0	9.978361	0.493202	-2.349820
35	6	0	8.660990	2.814243	-2.886457
36	1	0	10.505216	2.498755	-3.724789
37	6	0	8.136428	3.067924	-1.477409
38	1	0	8.475743	3.691246	-3.505271
39	1	0	8.107460	1.964421	-3.316072
40	1	0	8.659306	3.926197	-1.052129
41	1	0	7.076281	3.289213	-1.481493
42	6	0	5.813305	-2.375350	3.408494
43	6	0	5.076873	-2.455473	4.742610
44	1	0	6.582632	-3.149107	3.364083
45	1	0	6.271464	-1.397509	3.311397

46	7	0	4.389666	-3.726241	4.849745
47	1	0	5.809043	-2.368455	5.544287
48	1	0	4.392001	-1.595266	4.809495
49	6	0	3.376637	-3.864930	3.822405
50	1	0	4.008779	-3.867035	5.774683
51	6	0	4.085316	-3.819479	2.477061
52	1	0	2.872869	-4.824563	3.929237
53	1	0	2.614948	-3.070101	3.865044
54	1	0	4.808433	-4.638008	2.446582
55	1	0	3.408512	-3.955818	1.638207
56	12	0	0.000099	-1.196980	-0.000086
57	7	0	-1.675749	-0.067692	0.380298
58	6	0	-1.841514	1.113654	1.091293
59	6	0	-3.186744	1.500343	1.061861
60	7	0	-3.843536	0.518533	0.300799
61	6	0	-2.897663	-0.380031	-0.071981
62	6	0	-3.221815	-1.473720	-0.920598
63	6	0	-5.196933	0.350288	-0.039247
64	6	0	-4.545630	-1.639552	-1.403914
65	6	0	-5.537279	-0.755448	-0.861688
66	6	0	-0.942990	1.920425	1.768894
67	7	0	-3.702625	2.567676	1.627051
68	6	0	-1.465373	3.053770	2.372318
69	6	0	-2.829019	3.334915	2.279587
70	6	0	-2.040415	-2.112684	-1.308292
71	7	0	-0.931179	-2.430301	-1.426345
72	6	0	-6.923965	-1.003337	-0.983709
73	6	0	-6.135869	1.213148	0.472208
74	6	0	-7.873485	-0.175102	-0.470784
75	6	0	-7.516379	1.022828	0.221301
76	7	0	-8.436936	1.908238	0.636309
77	7	0	-4.851541	-2.581050	-2.312257
78	1	0	0.111309	1.680641	1.824481
79	1	0	-0.824531	3.728841	2.920016
80	1	0	-3.234166	4.219525	2.752180
81	1	0	-5.783639	2.018749	1.087368
82	1	0	-8.904986	-0.461334	-0.583312
83	1	0	-7.252436	-1.912000	-1.464979
84	6	0	-5.813132	-2.375331	-3.408521
85	6	0	-5.076667	-2.455301	-4.742628
86	1	0	-6.582438	-3.149114	-3.364199
87	1	0	-6.271326	-1.397512	-3.311350
88	7	0	-4.389399	-3.726029	-4.849865
89	1	0	-5.808828	-2.368247	-5.544310
90	1	0	-4.391835	-1.595056	-4.809428
91	6	0	-3.376391	-3.864764	-3.822512
92	1	0	-4.008493	-3.866731	-5.774809
93	6	0	-4.085110	-3.819477	-2.477186
94	1	0	-2.872583	-4.824369	-3.929416
95	1	0	-2.614728	-3.069908	-3.865067
96	1	0	-4.808198	-4.638036	-2.446832
97	1	0	-3.408339	-3.955875	-1.638319
98	6	0	-9.882833	1.688287	0.570916
99	6	0	-10.417471	1.432546	1.975610
100	1	0	-10.336176	2.599032	0.176245
101	1	0	-10.129065	0.875340	-0.099566
102	7	0	-10.092806	2.578623	2.805965
103	1	0	-11.499252	1.313419	1.934683
104	1	0	-9.980232	0.493297	2.349140
105	6	0	-8.662471	2.813767	2.886900
106	1	0	-10.507553	2.499080	3.723635
107	6	0	-8.136504	3.067287	1.478342
108	1	0	-8.477375	3.690672	3.505896
109	1	0	-8.109675	1.963698	3.316965
110	1	0	-8.658486	3.925904	1.052647
111	1	0	-7.076240	3.287953	1.483557

Ligand	Description	Total Gibbs Free energy (M06-2X/Def2TZVPP)
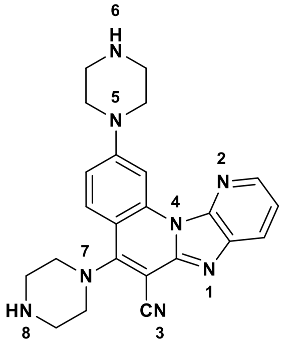 **5a**	2:1 complex with Zn^{2+} bound to N1	-4435.932824 a.u.

CARTESIAN COORDINATES

Center Number	Atomic Number	Atomic Type	Coordinates (Angstroms) X	Y	Z
1	7	0	3.976414	0.254649	0.666414
2	6	0	5.305420	-0.107286	0.385955
3	6	0	2.949672	-0.135062	-0.129909
4	7	0	1.770188	0.343574	0.289259
5	6	0	2.047458	1.092757	1.422723
6	6	0	3.422540	1.056029	1.680008
7	6	0	3.161084	-1.010900	-1.226717
8	7	0	4.041791	1.649554	2.674072
9	6	0	1.235905	1.812805	2.281593
10	6	0	3.251905	2.341617	3.495547
11	6	0	1.869344	2.447217	3.338696
12	1	0	3.743668	2.837548	4.321460
13	1	0	1.301281	3.028804	4.049600
14	1	0	0.165051	1.878461	2.136922
15	6	0	5.534786	-0.959258	-0.725559
16	6	0	4.460988	-1.509695	-1.501909
17	7	0	4.672140	-2.440713	-2.447741
18	6	0	6.324604	0.413775	1.146115
19	6	0	6.891904	-1.103744	-1.096049
20	6	0	7.918417	-0.581840	-0.371967
21	6	0	7.679203	0.142994	0.835722
22	1	0	7.131848	-1.615022	-2.015770
23	1	0	8.918787	-0.723291	-0.743232
24	7	0	8.679928	0.568703	1.623769
25	1	0	6.056353	1.060135	1.959668
26	6	0	1.927176	-1.403617	-1.759656
27	7	0	0.797160	-1.527758	-1.992022
28	6	0	10.095095	0.548533	1.248940
29	6	0	10.562494	1.975164	0.979820
30	1	0	10.655749	0.138944	2.090708
31	1	0	10.267895	-0.085452	0.389267
32	7	0	10.339449	2.769242	2.174575
33	1	0	11.626338	1.967661	0.746703
34	1	0	10.019140	2.358274	0.101868
35	6	0	8.939674	2.805977	2.558404
36	1	0	10.721675	3.699123	2.076300
37	6	0	8.482533	1.377178	2.827722
38	1	0	8.831526	3.398708	3.465766
39	1	0	8.290853	3.240284	1.781441
40	1	0	9.107129	0.948386	3.613344
41	1	0	7.452810	1.340296	3.161875
42	6	0	5.637713	-3.543278	-2.305413
43	6	0	4.885471	-4.870870	-2.304023
44	1	0	6.340918	-3.518827	-3.140628
45	1	0	6.176011	-3.436282	-1.370223

46	7	0	4.095842	-4.989664	-3.512836
47	1	0	5.615202	-5.678509	-2.264879
48	1	0	4.272796	-4.918355	-1.389866
49	6	0	3.083465	-3.954627	-3.584054
50	1	0	3.696365	-5.912685	-3.607056
51	6	0	3.804027	-2.615335	-3.615956
52	1	0	2.503120	-4.069967	-4.498406
53	1	0	2.387943	-3.978699	-2.730236
54	1	0	4.457281	-2.601917	-4.491822
55	1	0	3.124532	-1.772728	-3.707500
56	30	0	0.038561	-0.028114	-0.653777
57	7	0	-1.722421	-0.426583	0.221977
58	6	0	-2.034687	-1.216909	1.317856
59	6	0	-3.417266	-1.191402	1.531479
60	7	0	-3.939781	-0.352242	0.531350
61	6	0	-2.888690	0.064961	-0.217599
62	6	0	-3.067514	0.974569	-1.292917
63	6	0	-5.259819	0.008763	0.212952
64	6	0	-4.357233	1.482141	-1.591764
65	6	0	-5.456256	0.901570	-0.869690
66	6	0	-1.249877	-1.968356	2.174946
67	7	0	-4.067455	-1.823475	2.480764
68	6	0	-1.916165	-2.643490	3.185433
69	6	0	-3.303680	-2.545845	3.300419
70	6	0	-1.817528	1.384407	-1.774859
71	7	0	-0.681146	1.516212	-1.966982
72	6	0	-6.801473	1.072848	-1.270564
73	6	0	-6.304103	-0.537709	0.925643
74	6	0	-7.848200	0.524312	-0.596538
75	6	0	-7.643923	-0.275385	0.566825
76	7	0	-8.678196	-0.804584	1.254846
77	7	0	-4.540745	2.446216	-2.509297
78	1	0	-0.174772	-2.027787	2.062775
79	1	0	-1.370372	-3.251333	3.891800
80	1	0	-3.821463	-3.073893	4.089721
81	1	0	-6.061536	-1.162375	1.764305
82	1	0	-8.838183	0.666461	-0.996018
83	1	0	-7.012661	1.618648	-2.177261
84	6	0	-5.507465	3.546755	-2.354951
85	6	0	-4.751957	4.870479	-2.281497
86	1	0	-6.185095	3.555720	-3.211266
87	1	0	-6.073876	3.407568	-1.440919
88	7	0	-3.926436	5.031483	-3.460930
89	1	0	-5.480646	5.678595	-2.233703
90	1	0	-4.166751	4.881509	-1.348321
91	6	0	-2.914431	3.996800	-3.539978
92	1	0	-3.523110	5.956307	-3.510020
93	6	0	-3.636463	2.661870	-3.642978
94	1	0	-2.306857	4.143613	-4.431842
95	1	0	-2.244533	3.987634	-2.665669
96	1	0	-4.263050	2.683082	-4.537901
97	1	0	-2.956434	1.820886	-3.745116
98	6	0	-10.017048	-0.201478	1.230098
99	6	0	-11.101088	-1.251184	1.396538
100	1	0	-10.084798	0.510496	2.059156
101	1	0	-10.171883	0.337302	0.303846
102	7	0	-10.858346	-1.972811	2.629988
103	1	0	-12.065128	-0.747590	1.449426
104	1	0	-11.102059	-1.908219	0.514055
105	6	0	-9.598787	-2.682817	2.548985
106	1	0	-11.620355	-2.600354	2.845985
107	6	0	-8.458920	-1.685277	2.404700
108	1	0	-9.440049	-3.254777	3.462060
109	1	0	-9.561684	-3.378592	1.697260
110	1	0	-8.376098	-1.082965	3.315535
111	1	0	-7.538267	-2.242961	2.264216

Ligand	Description	Total Gibbs Free energy (M06-2X/Def2TZVPP)
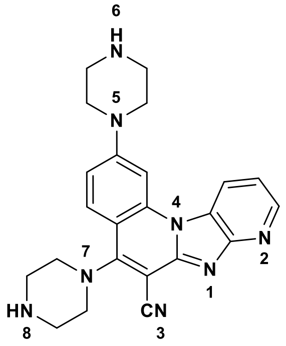 **5b**	2:1 complex with H^{+} bound to N1	-2657.326426 a.u.

CARTESIAN COORDINATES

Center	Atomic	Atomic	Coordinates (Angstroms)		
Number	Number	Type	X	Y	Z
1	7	0	3.548633	1.053169	-0.006223
2	6	0	4.672560	0.277873	0.269112
3	6	0	2.277471	0.594225	0.271157
4	7	0	1.337521	1.418875	-0.128235
5	6	0	1.989968	2.476736	-0.708749
6	6	0	3.387317	2.289009	-0.640438
7	6	0	2.086275	-0.621949	0.988570
8	6	0	4.217057	3.281967	-1.139110
9	7	0	1.387397	3.522031	-1.252764
10	6	0	3.586346	4.381790	-1.700789
11	6	0	2.193385	4.453365	-1.743811
12	1	0	4.174460	5.191422	-2.106909
13	1	0	1.713337	5.313248	-2.194818
14	6	0	4.491861	-0.939852	0.951930
15	6	0	3.178308	-1.379138	1.391779
16	7	0	3.061274	-2.513464	2.138245
17	6	0	5.929513	0.680014	-0.168910
18	6	0	5.627070	-1.767064	1.057738
19	6	0	6.862335	-1.386748	0.618032
20	6	0	7.056015	-0.125738	0.005375
21	1	0	5.511059	-2.761418	1.459446
22	1	0	7.678557	-2.088148	0.687596
23	7	0	8.297417	0.246593	-0.460520
24	1	0	6.024568	1.625384	-0.660807
25	6	0	0.743270	-0.875911	1.375813
26	7	0	-0.359383	-1.036568	1.663110
27	6	0	9.475525	-0.074808	0.351390
28	6	0	10.743093	-0.008761	-0.478942
29	1	0	9.553804	0.644727	1.175830
30	1	0	9.371928	-1.067807	0.775209
31	7	0	10.844164	1.313377	-1.070612
32	1	0	11.599269	-0.182717	0.171673
33	1	0	10.713693	-0.806791	-1.235692
34	6	0	9.729580	1.539836	-1.970984
35	1	0	11.725471	1.429362	-1.551325
36	6	0	8.426523	1.502300	-1.188888
37	1	0	9.829940	2.519453	-2.436765
38	1	0	9.674975	0.783348	-2.767958
39	1	0	8.386396	2.357125	-0.499448
40	1	0	7.603897	1.582050	-1.897917
41	6	0	3.923650	-2.797102	3.287112
42	6	0	3.075996	-2.907015	4.548751
43	1	0	4.447830	-3.745476	3.134286
44	1	0	4.651263	-1.998513	3.400077
45	7	0	2.081567	-3.949507	4.364314
46	1	0	3.724064	-3.168884	5.384907

47	1	0	2.626302	-1.923339	4.753827
48	6	0	1.159075	-3.598257	3.293104
49	1	0	1.581402	-4.124012	5.224799
50	6	0	1.959155	-3.465377	2.005797
51	1	0	0.425620	-4.395492	3.173908
52	1	0	0.622889	-2.661346	3.487385
53	1	0	2.409175	-4.438814	1.785433
54	1	0	1.331252	-3.186879	1.164983
55	1	0	-0.287922	1.395400	-0.034350
56	7	0	-1.357719	1.491036	0.050088
57	6	0	-1.992413	2.566516	0.629656
58	6	0	-3.377091	2.344145	0.568032
59	7	0	-3.529386	1.087477	-0.042623
60	6	0	-2.280569	0.618649	-0.343112
61	6	0	-2.079590	-0.587547	-1.042936
62	6	0	-4.660077	0.303839	-0.284925
63	6	0	-3.185306	-1.357358	-1.419411
64	6	0	-4.488165	-0.917357	-0.963308
65	7	0	-1.392021	3.616879	1.144188
66	6	0	-4.216407	3.340803	1.044125
67	6	0	-2.206386	4.550602	1.621222
68	6	0	-3.597241	4.460203	1.579729
69	6	0	-0.754272	-0.847646	-1.486326
70	7	0	0.320465	-1.035671	-1.847199
71	6	0	-5.625221	-1.748887	-1.034324
72	6	0	-5.902403	0.703945	0.188767
73	6	0	-6.848636	-1.369028	-0.567557
74	6	0	-7.033757	-0.105085	0.046888
75	7	0	-8.257624	0.266118	0.541865
76	7	0	-3.061190	-2.488204	-2.155961
77	1	0	-1.731945	5.422523	2.053023
78	1	0	-4.195746	5.271612	1.966288
79	1	0	-5.985964	1.645632	0.687961
80	1	0	-7.662069	-2.075012	-0.614346
81	1	0	-5.518208	-2.746557	-1.428707
82	6	0	-3.962198	-2.829962	-3.260538
83	6	0	-3.148630	-3.006845	-4.537218
84	1	0	-4.480892	-3.768416	-3.043866
85	1	0	-4.690175	-2.034614	-3.392959
86	7	0	-2.157633	-4.046483	-4.328191
87	1	0	-3.822616	-3.304051	-5.340266
88	1	0	-2.699980	-2.037425	-4.802428
89	6	0	-1.200943	-3.650043	-3.304044
90	1	0	-1.685598	-4.272547	-5.192561
91	6	0	-1.960938	-3.445783	-2.003173
92	1	0	-0.471493	-4.447143	-3.164072
93	1	0	-0.662605	-2.729584	-3.559634
94	1	0	-2.419404	-4.399129	-1.722212
95	1	0	-1.308801	-3.134337	-1.193583
96	6	0	-9.469072	-0.106488	-0.197941
97	6	0	-10.695561	-0.043778	0.692187
98	1	0	-9.603621	0.586881	-1.036654
99	1	0	-9.364810	-1.108944	-0.597804
100	7	0	-10.794695	1.290940	1.253708
101	1	0	-11.577302	-0.252017	0.087685
102	1	0	-10.613964	-0.820808	1.466674
103	6	0	-9.647541	1.556273	2.099529
104	1	0	-11.657486	1.407209	1.766807
105	6	0	-8.379286	1.529053	1.261411
106	1	0	-9.745361	2.542878	2.550606
107	1	0	-9.543144	0.817142	2.907705
108	1	0	-8.383121	2.373382	0.558778
109	1	0	-7.530070	1.632166	1.934918
110	1	0	-5.291649	3.303074	1.001567
111	1	0	5.293403	3.254340	-1.099270

Ligand	Description	Total Gibbs Free energy (M06-2X/Def2TZVPP)
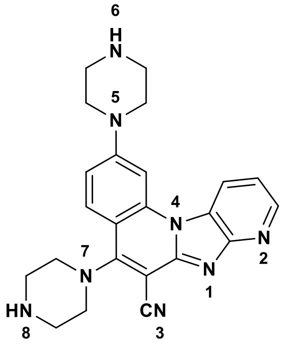 5b	2:1 complex with Ca^{2+} bound to N1	-3334.240758 a.u.

CARTESIAN COORDINATES

Center Number	Atomic Number	Atomic Type	Coordinates (Angstroms) X	Y	Z
1	7	0	4.411325	-0.512869	-0.553044
2	6	0	5.729072	-0.387262	-0.093976
3	6	0	3.390446	0.116442	0.099497
4	7	0	2.199556	-0.168834	-0.407895
5	6	0	2.445588	-1.022016	-1.456973
6	6	0	3.824262	-1.261470	-1.586521
7	6	0	3.627599	1.049660	1.144220
8	6	0	4.253912	-2.060393	-2.634090
9	7	0	1.492503	-1.502422	-2.234162
10	6	0	3.258849	-2.578583	-3.453669
11	6	0	1.914725	-2.286551	-3.223733
12	1	0	3.526090	-3.209403	-4.288221
13	1	0	1.152769	-2.696814	-3.873132
14	6	0	5.989278	0.551027	0.930750
15	6	0	4.949301	1.377019	1.506983
16	7	0	5.242521	2.371623	2.361429
17	6	0	6.718161	-1.203837	-0.607840
18	6	0	7.294234	0.515462	1.469616
19	6	0	8.279110	-0.285347	0.976187
20	6	0	8.034988	-1.167664	-0.112494
21	1	0	7.514248	1.108442	2.344197
22	1	0	9.235042	-0.282982	1.472646
23	7	0	9.008328	-1.977780	-0.601552
24	1	0	6.460583	-1.886886	-1.391374
25	6	0	2.413522	1.675471	1.485195
26	7	0	1.310453	2.010088	1.577665
27	6	0	10.418083	-1.563148	-0.552742
28	6	0	11.352572	-2.757197	-0.604046
29	1	0	10.619699	-0.917314	-1.414066
30	1	0	10.608920	-0.997877	0.351538
31	7	0	11.049540	-3.531813	-1.791545
32	1	0	12.376815	-2.391260	-0.655205
33	1	0	11.239911	-3.341658	0.321021
34	6	0	9.709381	-4.070383	-1.694789
35	1	0	11.729538	-4.265230	-1.936108
36	6	0	8.703503	-2.929658	-1.668990
37	1	0	9.498815	-4.694329	-2.562239
38	1	0	9.565750	-4.680398	-0.790417
39	1	0	8.700377	-2.416810	-2.638552
40	1	0	7.720951	-3.359863	-1.488319
41	6	0	6.385467	3.283405	2.181948
42	6	0	5.866113	4.688000	1.888954
43	1	0	6.983390	3.294027	3.095572
44	1	0	6.994115	2.947039	1.350113
45	7	0	4.988072	5.119437	2.957731

46	1	0	6.718762	5.362624	1.823275
47	1	0	5.365473	4.672048	0.907866
48	6	0	3.817172	4.268873	3.051478
49	1	0	4.736920	6.093106	2.861218
50	6	0	4.301027	2.862350	3.371479
51	1	0	3.173239	4.617329	3.857768
52	1	0	3.226669	4.252480	2.122323
53	1	0	4.851313	2.898885	4.314543
54	1	0	3.486567	2.154802	3.496468
55	20	0	0.000006	0.646389	0.000298
56	7	0	-2.199640	-0.168567	0.408431
57	6	0	-2.445859	-1.021346	1.457789
58	6	0	-3.824550	-1.260751	1.587182
59	7	0	-4.411430	-0.512529	0.553330
60	6	0	-3.390438	0.116540	-0.099270
61	6	0	-3.627410	1.049409	-1.144348
62	6	0	-5.729089	-0.387126	0.093972
63	6	0	-4.949056	1.376674	-1.507394
64	6	0	-5.989121	0.550800	-0.931109
65	7	0	-1.492906	-1.501459	2.235325
66	6	0	-4.254388	-2.059256	2.634996
67	6	0	-1.915300	-2.285230	3.225104
68	6	0	-3.259471	-2.577147	3.454935
69	6	0	-2.413248	1.675047	-1.485348
70	7	0	-1.310146	2.009576	-1.577762
71	6	0	-7.293960	0.515001	-1.470261
72	6	0	-6.718251	-1.203579	0.607910
73	6	0	-8.278898	-0.285680	-0.976765
74	6	0	-8.034968	-1.167604	0.112285
75	7	0	-9.008404	-1.977566	0.601420
76	7	0	-5.242201	2.371043	-2.362145
77	1	0	-1.153461	-2.695244	3.874794
78	1	0	-3.526856	-3.207625	4.289698
79	1	0	-6.460816	-1.886385	1.391704
80	1	0	-9.234722	-0.283579	-1.473430
81	1	0	-7.513789	1.107670	-2.345099
82	6	0	-6.385195	3.282851	-2.182985
83	6	0	-5.865891	4.687462	-1.889995
84	1	0	-6.982901	3.293385	-3.096750
85	1	0	-6.994036	2.946558	-1.351266
86	7	0	-4.987642	5.118817	-2.958627
87	1	0	-6.718546	5.362098	-1.824518
88	1	0	-5.365442	4.671569	-0.908805
89	6	0	-3.816728	4.268244	-3.052093
90	1	0	-4.736507	6.092494	-2.862150
91	6	0	-4.300523	2.861691	-3.372058
92	1	0	-3.172653	4.616622	-3.858302
93	1	0	-3.226385	4.251937	-2.122834
94	1	0	-4.850633	2.898138	-4.315226
95	1	0	-3.486040	2.154131	-3.496838
96	6	0	-10.418127	-1.562848	0.552395
97	6	0	-11.352681	-2.756832	0.603873
98	1	0	-10.619778	-0.916833	1.413575
99	1	0	-10.608842	-0.997729	-0.352008
100	7	0	-11.049817	-3.531184	1.791600
101	1	0	-12.376909	-2.390827	0.654840
102	1	0	-11.239959	-3.341531	-0.321032
103	6	0	-9.709664	-4.069836	1.695133
104	1	0	-11.729858	-4.264550	1.936237
105	6	0	-8.703737	-2.929160	1.669161
106	1	0	-9.499223	-4.693570	2.562766
107	1	0	-9.565956	-4.680085	0.790933
108	1	0	-8.700726	-2.416041	2.638581
109	1	0	-7.721166	-3.359427	1.488734
110	1	0	-5.288783	-2.272843	2.850882
111	1	0	5.288261	-2.274119	-2.850055

Ligand	Description	Total Gibbs Free energy (M06-2X/Def2TZVPP)
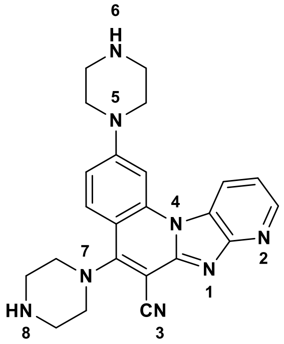 5b	2:1 complex with Cu^{2+} bound to N1	-4296.971180 a.u.

CARTESIAN COORDINATES

Center Number	Atomic Number	Atomic Type	X	Y	Z
1	7	0	4.188991	-0.608917	-0.624547
2	6	0	5.439405	-0.159279	-0.190479
3	6	0	3.045776	-0.279556	0.055456
4	7	0	1.972276	-0.868312	-0.447275
5	6	0	2.411536	-1.606454	-1.521716
6	6	0	3.803622	-1.458176	-1.668932
7	6	0	3.060181	0.676832	1.106747
8	6	0	4.426244	-2.070587	-2.744650
9	7	0	1.628571	-2.319752	-2.309943
10	6	0	3.609185	-2.824272	-3.575125
11	6	0	2.241963	-2.927930	-3.319877
12	1	0	4.029773	-3.329381	-4.431857
13	1	0	1.616122	-3.523309	-3.972462
14	6	0	5.472285	0.787485	0.856437
15	6	0	4.263297	1.301113	1.464553
16	7	0	4.326965	2.312228	2.360886
17	6	0	6.594940	-0.699873	-0.723371
18	6	0	6.749884	1.065158	1.385819
19	6	0	7.899497	0.547994	0.867266
20	6	0	7.868887	-0.327783	-0.252815
21	1	0	6.827551	1.691631	2.260900
22	1	0	8.832978	0.820337	1.329948
23	7	0	9.005882	-0.786195	-0.831298
24	1	0	6.505371	-1.482469	-1.449543
25	6	0	1.758969	1.044413	1.529204
26	7	0	0.647175	1.236335	1.773638
27	6	0	10.287701	-0.864275	-0.129439
28	6	0	10.507257	-2.295294	0.352276
29	1	0	11.074341	-0.593105	-0.834867
30	1	0	10.325193	-0.170525	0.700963
31	7	0	10.465110	-3.189152	-0.793202
32	1	0	11.483188	-2.372882	0.829821
33	1	0	9.736003	-2.527927	1.103592
34	6	0	9.194908	-3.128837	-1.497048
35	1	0	10.694259	-4.136298	-0.527144
36	6	0	8.997123	-1.694649	-1.973311
37	1	0	9.227341	-3.803054	-2.352225
38	1	0	8.339929	-3.413664	-0.862579
39	1	0	9.839268	-1.420871	-2.611122
40	1	0	8.090997	-1.569008	-2.557410
41	6	0	5.190870	3.486101	2.182680
42	6	0	4.326817	4.737523	2.070736
43	1	0	5.856762	3.585851	3.043775
44	1	0	5.780628	3.369991	1.279055
45	7	0	3.495380	4.858201	3.253000

46	1	0	4.981136	5.605478	1.996881
47	1	0	3.738205	4.669378	1.142394
48	6	0	2.572959	3.740335	3.359351
49	1	0	3.007010	5.742319	3.268733
50	6	0	3.398244	2.468369	3.481739
51	1	0	1.963064	3.855822	4.254624
52	1	0	1.902402	3.657251	2.492394
53	1	0	4.010980	2.547770	4.384096
54	1	0	2.778234	1.583097	3.578277
55	29	0	0.089467	-0.632431	0.133087
56	7	0	-1.874791	-0.805071	0.539288
57	6	0	-2.303308	-1.492081	1.631261
58	6	0	-3.705512	-1.372850	1.768416
59	7	0	-4.108242	-0.603396	0.686304
60	6	0	-2.962470	-0.267799	-0.001578
61	6	0	-2.993312	0.641519	-1.075578
62	6	0	-5.367117	-0.232469	0.202655
63	6	0	-4.231604	1.230783	-1.478181
64	6	0	-5.413517	0.689486	-0.880079
65	7	0	-1.500903	-2.154705	2.459047
66	6	0	-4.318747	-1.937968	2.883689
67	6	0	-2.095922	-2.714395	3.489319
68	6	0	-3.483112	-2.619173	3.743543
69	6	0	-1.717073	1.062404	-1.536117
70	7	0	-0.654640	1.374978	-1.846232
71	6	0	-6.698446	0.921548	-1.436551
72	6	0	-6.502619	-0.803142	0.717381
73	6	0	-7.835348	0.387782	-0.926581
74	6	0	-7.793804	-0.479508	0.212399
75	7	0	-8.907792	-0.985517	0.750464
76	7	0	-4.279183	2.241061	-2.362430
77	1	0	-1.464618	-3.263926	4.176055
78	1	0	-3.884697	-3.081429	4.633563
79	1	0	-6.398134	-1.527447	1.499528
80	1	0	-8.762694	0.574324	-1.440325
81	1	0	-6.773034	1.508141	-2.338844
82	6	0	-5.210018	3.374903	-2.239341
83	6	0	-4.407008	4.672308	-2.226829
84	1	0	-5.893232	3.386486	-3.091962
85	1	0	-5.774644	3.278017	-1.316303
86	7	0	-3.624496	4.758656	-3.441459
87	1	0	-5.107349	5.505444	-2.182095
88	1	0	-3.790758	4.691055	-1.314849
89	6	0	-2.638994	3.694658	-3.508641
90	1	0	-3.198275	5.668700	-3.545111
91	6	0	-3.386932	2.371384	-3.526771
92	1	0	-2.065709	3.784088	-4.430013
93	1	0	-1.937271	3.704231	-2.663641
94	1	0	-4.037106	2.353874	-4.405758
95	1	0	-2.720580	1.518685	-3.589559
96	6	0	-10.265712	-0.603033	0.343370
97	6	0	-10.970118	-1.799848	-0.287397
98	1	0	-10.798852	-0.319567	1.252569
99	1	0	-10.246796	0.253403	-0.316912
100	7	0	-10.994839	-2.867840	0.690656
101	1	0	-11.989315	-1.514917	-0.542580
102	1	0	-10.443484	-2.071365	-1.215012
103	6	0	-9.664725	-3.279411	1.082240
104	1	0	-11.559158	-3.648969	0.387359
105	6	0	-8.947655	-2.087040	1.717485
106	1	0	-9.732247	-4.077565	1.819874
107	1	0	-9.054494	-3.638565	0.239422
108	1	0	-9.506943	-1.747990	2.590618
109	1	0	-7.952038	-2.375700	2.027740
110	1	0	-5.367675	-1.841913	3.113056
111	1	0	5.474715	-1.969750	-2.972570

Ligand	Description	Total Gibbs Free energy (M06-2X/Def2TZVPP)
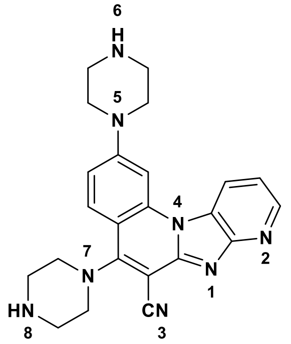 5b	2:1 complex with Mg^{2+} bound to N1	-2856.677280 a.u.

CARTESIAN COORDINATES

Center Number	Atomic Number	Atomic Type	Coordinates (Angstroms) X	Y	Z
1	7	0	3.917889	-0.371468	-0.655267
2	6	0	5.231763	-0.375881	-0.164710
3	6	0	2.941420	0.319857	-0.004902
4	7	0	1.740846	0.148854	-0.550103
5	6	0	1.937602	-0.696627	-1.623653
6	6	0	3.296502	-1.037043	-1.725542
7	6	0	3.237583	1.193758	1.076871
8	6	0	3.697767	-1.831656	-2.787984
9	7	0	0.976465	-1.087457	-2.436142
10	6	0	2.692514	-2.252382	-3.647444
11	6	0	1.367370	-1.871990	-3.436720
12	1	0	2.936281	-2.874177	-4.495900
13	1	0	0.593809	-2.209095	-4.114135
14	6	0	5.552314	0.510323	0.889307
15	6	0	4.575899	1.404109	1.469900
16	7	0	4.928525	2.350326	2.353758
17	6	0	6.155060	-1.265185	-0.678400
18	6	0	6.837778	0.347358	1.451754
19	6	0	7.758909	-0.524058	0.956274
20	6	0	7.460999	-1.355977	-0.159058
21	1	0	7.089888	0.896094	2.346318
22	1	0	8.700570	-0.617324	1.470751
23	7	0	8.368976	-2.234632	-0.651515
24	1	0	5.854397	-1.907659	-1.480133
25	6	0	2.067517	1.890398	1.418151
26	7	0	0.974512	2.268207	1.470569
27	6	0	9.809512	-1.955299	-0.555670
28	6	0	10.629824	-3.230183	-0.610249
29	1	0	10.093902	-1.313977	-1.396602
30	1	0	10.026799	-1.428845	0.365763
31	7	0	10.290977	-3.946816	-1.824108
32	1	0	11.684678	-2.960673	-0.624555
33	1	0	10.436194	-3.821401	0.296961
34	6	0	8.904439	-4.359563	-1.777388
35	1	0	10.904171	-4.736830	-1.969124
36	6	0	8.008101	-3.130566	-1.750010
37	1	0	8.662008	-4.940787	-2.665902
38	1	0	8.678011	-4.974059	-0.893178
39	1	0	8.081246	-2.597396	-2.705584
40	1	0	6.985393	-3.471789	-1.607294
41	6	0	6.148106	3.167683	2.222704
42	6	0	5.753168	4.612643	1.930935
43	1	0	6.713658	3.118122	3.155232
44	1	0	6.754151	2.792166	1.406073
45	7	0	4.876372	5.103213	2.974698

46	1	0	6.659015	5.216772	1.902289
47	1	0	5.287293	4.646522	0.933361
48	6	0	3.639047	4.349081	3.021233
49	1	0	4.708213	6.095106	2.883206
50	6	0	3.999963	2.906129	3.342034
51	1	0	2.998148	4.741131	3.809651
52	1	0	3.080041	4.387940	2.072954
53	1	0	4.524007	2.891814	4.300301
54	1	0	3.129723	2.262974	3.439940
55	12	0	-0.000026	1.072112	-0.000390
56	7	0	-1.740674	0.148324	0.549141
57	6	0	-1.937231	-0.697291	1.622628
58	6	0	-3.296126	-1.037618	1.724803
59	7	0	-3.917736	-0.371824	0.654798
60	6	0	-2.941385	0.319526	0.004290
61	6	0	-3.237800	1.193687	-1.077204
62	6	0	-5.231715	-0.376181	0.164509
63	6	0	-4.576200	1.404197	-1.469818
64	6	0	-5.552512	0.510242	-0.889227
65	7	0	-0.975943	-1.088283	2.434861
66	6	0	-3.697234	-1.832216	2.787314
67	6	0	-1.366689	-1.872872	3.435456
68	6	0	-2.691817	-2.253129	3.646488
69	6	0	-2.067849	1.890521	-1.418503
70	7	0	-0.974894	2.268447	-1.471083
71	6	0	-6.838065	0.347355	-1.451483
72	6	0	-6.154866	-1.265655	0.678186
73	6	0	-7.759057	-0.524218	-0.956022
74	6	0	-7.460900	-1.356358	0.159082
75	7	0	-8.368788	-2.235150	0.651543
76	7	0	-4.929040	2.350678	-2.353306
77	1	0	-0.592996	-2.210124	4.112645
78	1	0	-2.935440	-2.874946	4.494965
79	1	0	-5.854002	-1.908352	1.479665
80	1	0	-8.700811	-0.617437	-1.470330
81	1	0	-7.090355	0.896295	-2.345868
82	6	0	-6.148585	3.167998	-2.221683
83	6	0	-5.753565	4.612845	-1.929471
84	1	0	-6.714328	3.118792	-3.154112
85	1	0	-6.754466	2.792181	-1.405068
86	7	0	-4.877012	5.103783	-2.973267
87	1	0	-6.659397	5.216974	-1.900388
88	1	0	-5.287448	4.646356	-0.931998
89	6	0	-3.639699	4.349663	-3.020348
90	1	0	-4.708824	6.095642	-2.881457
91	6	0	-4.000698	2.906830	-3.341591
92	1	0	-2.998974	4.741990	-3.808770
93	1	0	-3.080481	4.388179	-2.072181
94	1	0	-4.524957	2.892867	-4.299746
95	1	0	-3.130486	2.263702	-3.439929
96	6	0	-9.809326	-1.955596	0.556254
97	6	0	-10.629779	-3.230367	0.611077
98	1	0	-10.093331	-1.314278	1.397322
99	1	0	-10.026874	-1.429048	-0.365061
100	7	0	-10.290596	-3.947076	1.824819
101	1	0	-11.684593	-2.960725	0.625778
102	1	0	-10.436553	-3.821598	-0.296206
103	6	0	-8.904116	-4.360006	1.777618
104	1	0	-10.903807	-4.737066	1.969940
105	6	0	-8.007612	-3.131151	1.749900
106	1	0	-8.661477	-4.941260	2.666056
107	1	0	-8.678079	-4.974550	0.893342
108	1	0	-8.080269	-2.597940	2.705487
109	1	0	-6.985002	-3.472510	1.606765
110	1	0	-4.719821	-2.109100	2.985406
111	1	0	4.720352	-2.108705	-2.985838

Ligand	Description	Total Gibbs Free energy (M06-2X/Def2TZVPP)
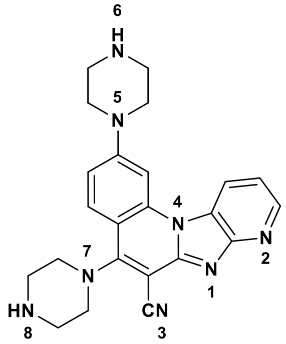 5b	2:1 complex with Zn^{2+} bound to N1	-4435.932133 a.u.

CARTESIAN COORDINATES

Center Number	Atomic Number	Atomic Type	Coordinates (Angstroms) X	Y	Z
1	7	0	3.960701	0.363543	0.494479
2	6	0	5.287782	-0.001418	0.223856
3	6	0	2.949923	-0.075744	-0.304491
4	7	0	1.775068	0.444670	0.040659
5	6	0	2.018421	1.256839	1.129081
6	6	0	3.385282	1.222120	1.447026
7	6	0	3.161181	-1.050167	-1.315230
8	6	0	3.817694	1.926867	2.559467
9	7	0	1.086187	1.928513	1.771176
10	6	0	2.844530	2.642227	3.243466
11	6	0	1.515726	2.624366	2.820633
12	1	0	3.114034	3.213764	4.119054
13	1	0	0.767845	3.188867	3.361839
14	6	0	5.517057	-0.996752	-0.752786
15	6	0	4.437996	-1.628880	-1.477705
16	7	0	4.656588	-2.680575	-2.281818
17	6	0	6.324339	0.645587	0.867517
18	6	0	6.869863	-1.208725	-1.099766
19	6	0	7.902801	-0.580964	-0.473445
20	6	0	7.670683	0.364634	0.564378
21	1	0	7.100090	-1.854684	-1.933215
22	1	0	8.902439	-0.766511	-0.829018
23	7	0	8.693405	1.006897	1.180880
24	1	0	6.086882	1.391096	1.598163
25	6	0	1.919196	-1.435469	-1.849466
26	7	0	0.790975	-1.521860	-2.095522
27	6	0	10.007012	0.361514	1.323455
28	6	0	11.116600	1.383844	1.482081
29	1	0	9.983296	-0.277296	2.212667
30	1	0	10.212731	-0.259984	0.460405
31	7	0	10.800652	2.237979	2.610108
32	1	0	12.048683	0.853924	1.671661
33	1	0	11.223750	1.946904	0.543214
34	6	0	9.595312	2.989482	2.331745
35	1	0	11.571448	2.851567	2.835011
36	6	0	8.421280	2.032731	2.187467
37	1	0	9.384835	3.668623	3.156687
38	1	0	9.673900	3.584136	1.409351
39	1	0	8.207971	1.559928	3.153756
40	1	0	7.557784	2.615257	1.874850
41	6	0	5.573173	-3.783155	-1.940676
42	6	0	4.761514	-5.055672	-1.715141
43	1	0	6.275797	-3.932320	-2.762879
44	1	0	6.115803	-3.540252	-1.034086
45	7	0	3.970475	-5.349022	-2.892752

46	1	0	5.453982	-5.875356	-1.527945
47	1	0	4.145521	-4.914757	-0.812872
48	6	0	3.006419	-4.298268	-3.155634
49	1	0	3.529208	-6.255381	-2.827813
50	6	0	3.789006	-3.019269	-3.413773
51	1	0	2.424283	-4.546088	-4.042015
52	1	0	2.308088	-4.140578	-2.318792
53	1	0	4.449005	-3.190125	-4.267239
54	1	0	3.151387	-2.175434	-3.661972
55	30	0	-0.000006	-0.000014	-0.745571
56	7	0	-1.775068	-0.444578	0.040733
57	6	0	-2.018389	-1.256541	1.129314
58	6	0	-3.385223	-1.221749	1.447313
59	7	0	-3.960672	-0.363318	0.494657
60	6	0	-2.949932	0.075798	-0.304452
61	6	0	-3.161222	1.050019	-1.315389
62	6	0	-5.287778	0.001498	0.223944
63	6	0	-4.438058	1.628681	-1.477923
64	6	0	-5.517091	0.996621	-0.752888
65	7	0	-1.086123	-1.928103	1.771497
66	6	0	-3.817588	-1.926255	2.559933
67	6	0	-1.515616	-2.623752	2.821106
68	6	0	-2.844407	-2.641498	3.244018
69	6	0	-1.919253	1.435120	-1.849791
70	7	0	-0.791033	1.521335	-2.095920
71	6	0	-6.869902	1.208443	-1.099965
72	6	0	-6.324310	-0.645435	0.867724
73	6	0	-7.902812	0.580750	-0.473547
74	6	0	-7.670656	-0.364623	0.564481
75	7	0	-8.693361	-1.006808	1.181085
76	7	0	-4.656732	2.680235	-2.282198
77	1	0	-0.767722	-3.188154	3.362391
78	1	0	-3.113870	-3.212850	4.119738
79	1	0	-6.086851	-1.390766	1.598552
80	1	0	-8.902462	0.766118	-0.829175
81	1	0	-7.100118	1.854218	-1.933560
82	6	0	-5.573333	3.782847	-1.941163
83	6	0	-4.761671	5.055338	-1.715492
84	1	0	-6.275828	3.932024	-2.763471
85	1	0	-6.116111	3.539959	-1.034660
86	7	0	-3.970445	5.348692	-2.892976
87	1	0	-5.454147	5.875036	-1.528381
88	1	0	-4.145818	4.914391	-0.813132
89	6	0	-3.006404	4.297899	-3.155756
90	1	0	-3.529145	6.255027	-2.827942
91	6	0	-3.789026	3.018951	-3.414054
92	1	0	-2.424114	4.545722	-4.042036
93	1	0	-2.308210	4.140141	-2.318812
94	1	0	-4.448941	3.189897	-4.267563
95	1	0	-3.151439	2.175090	-3.662248
96	6	0	-10.007039	-0.361510	1.323449
97	6	0	-11.116555	-1.383926	1.482034
98	1	0	-9.983475	0.277366	2.212615
99	1	0	-10.212699	0.259903	0.460325
100	7	0	-10.800654	-2.237926	2.610172
101	1	0	-12.048709	-0.854068	1.671444
102	1	0	-11.223537	-1.947084	0.543205
103	6	0	-9.595219	-2.989349	2.332029
104	1	0	-11.571419	-2.851542	2.835093
105	6	0	-8.421246	-2.032505	2.187817
106	1	0	-9.384794	-3.668426	3.157036
107	1	0	-9.673623	-3.584058	1.409654
108	1	0	-8.208103	-1.559581	3.154080
109	1	0	-7.557654	-2.614974	1.875347
110	1	0	-4.833128	-1.924785	2.920851
111	1	0	4.833251	1.925510	2.920332

Ligand	Description	Total Gibbs Free energy (M06-2X/Def2TZVPP)
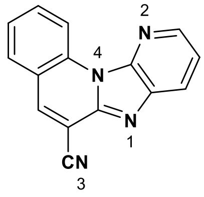 **6a**	2:1 complex with H$^+$ bound to N1	-1590.892470 a.u.

CARTESIAN COORDINATES

Center Number	Atomic Number	Atomic Type	Coordinates (Angstroms) X	Y	Z
1	7	0	3.471538	-0.159429	0.010306
2	6	0	4.549160	0.675682	-0.303245
3	6	0	2.204060	0.096779	-0.404602
4	7	0	1.369049	-0.849204	0.018586
5	6	0	2.090270	-1.749794	0.769882
6	6	0	3.424174	-1.341019	0.770110
7	6	0	1.910503	1.256192	-1.174898
8	7	0	4.422230	-1.951645	1.370566
9	6	0	1.737667	-2.895708	1.468746
10	6	0	4.082336	-3.050216	2.034751
11	6	0	2.773477	-3.546257	2.110355
12	1	0	4.886804	-3.569081	2.538703
13	1	0	2.585140	-4.444797	2.679211
14	1	0	0.717327	-3.249472	1.511493
15	6	0	4.268552	1.822345	-1.073788
16	6	0	2.934910	2.091146	-1.492993
17	1	0	2.736703	2.982998	-2.072735
18	6	0	5.847662	0.399756	0.121189
19	6	0	5.323985	2.687887	-1.408989
20	6	0	6.603168	2.417409	-0.991954
21	6	0	6.856981	1.273107	-0.228009
22	1	0	5.106910	3.567994	-1.999526
23	1	0	7.412619	3.084658	-1.250507
24	1	0	7.865675	1.063442	0.099879
25	1	0	6.047626	-0.479166	0.711477
26	6	0	0.555530	1.503065	-1.554022
27	7	0	-0.542466	1.679671	-1.835076
28	1	0	0.299458	-0.824658	-0.056415
29	7	0	-1.359203	-0.871330	-0.071277
30	6	0	-2.184780	0.061146	0.358640
31	7	0	-3.489069	-0.145109	-0.022535
32	6	0	-3.481942	-1.320473	-0.774169
33	6	0	-2.145436	-1.744881	-0.788982
34	6	0	-1.842951	-2.909181	-1.487541
35	7	0	-4.504581	-1.917309	-1.353394
36	6	0	-4.534081	0.714780	0.321462
37	6	0	-1.869071	1.221065	1.138784
38	6	0	-2.858538	2.079174	1.489124
39	6	0	-4.211134	1.847431	1.095017
40	6	0	-2.899215	-3.549574	-2.105085
41	6	0	-4.196729	-3.027503	-2.012833
42	1	0	-0.832410	-3.290437	-1.547994
43	1	0	-2.738812	-4.458081	-2.666934
44	1	0	-5.019200	-3.536032	-2.498552
45	6	0	-0.500818	1.429112	1.491359
46	7	0	0.612468	1.563020	1.738290
47	1	0	-2.627766	2.959648	2.074122
48	6	0	-5.236157	2.734597	1.460278
49	6	0	-5.849344	0.477842	-0.075491
50	6	0	-6.532443	2.501597	1.070144
51	6	0	-6.830572	1.371862	0.302746
52	1	0	-7.850925	1.189259	-0.004896

53	1	0	-6.081690	-0.391764	-0.668351
54	1	0	-4.984277	3.603736	2.053628
55	1	0	-7.318283	3.186888	1.353220

<table>
<tr><th>Ligand</th><th>Description</th><th>Total Gibbs Free energy (M06-2X/Def2TZVPP)</th></tr>
<tr><td>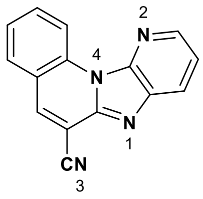

6a</td><td>2:1 complex with Ca^{2+} bound to N1</td><td>-2267.745226 a.u.</td></tr>
</table>

CARTESIAN COORDINATES

Center Number	Atomic Number	Atomic Type	Coordinates (Angstroms) X	Y	Z
1	7	0	-4.401165	0.408552	-0.161266
2	6	0	-5.628933	-0.254232	-0.271378
3	6	0	-3.275592	-0.190381	0.315075
4	7	0	-2.210624	0.608165	0.348323
5	6	0	-2.676392	1.825390	-0.138387
6	6	0	-4.032723	1.726828	-0.465378
7	6	0	-3.342590	-1.559565	0.727575
8	7	0	-4.799855	2.678035	-0.951859
9	6	0	-2.052568	3.053842	-0.332885
10	6	0	-4.190550	3.842063	-1.137454
11	6	0	-2.837690	4.068248	-0.842956
12	1	0	-4.799628	4.642133	-1.536674
13	1	0	-2.420882	5.049291	-1.017490
14	1	0	-1.011338	3.216293	-0.088362
15	6	0	-5.679861	-1.608936	0.140660
16	6	0	-4.517785	-2.246339	0.642294
17	1	0	-4.574483	-3.280177	0.957698
18	6	0	-6.765657	0.379383	-0.762280
19	6	0	-6.900617	-2.304942	0.045988
20	6	0	-8.019839	-1.677643	-0.438168
21	6	0	-7.944082	-0.338640	-0.839294
22	1	0	-6.937186	-3.339083	0.362159
23	1	0	-8.956502	-2.211046	-0.510469
24	1	0	-8.828900	0.152726	-1.220291
25	1	0	-6.718093	1.410512	-1.072839
26	6	0	-2.098264	-2.034403	1.208726
27	7	0	-1.005405	-2.177204	1.546272
28	20	0	0.000016	-0.000012	1.154613
29	7	0	2.210596	-0.608130	0.348158
30	6	0	3.275580	0.190404	0.314975
31	7	0	4.401174	-0.408556	-0.161284
32	6	0	4.032730	-1.726824	-0.465405
33	6	0	2.676389	-1.825359	-0.138510
34	6	0	2.052536	-3.053802	-0.333032
35	7	0	4.799877	-2.678063	-0.951828
36	6	0	5.628955	0.254214	-0.271348
37	6	0	3.342558	1.559602	0.727407
38	6	0	4.517759	2.246372	0.642157
39	6	0	5.679862	1.608937	0.140627
40	6	0	2.837659	-4.068226	-0.843046
41	6	0	4.190559	-3.842068	-1.137449
42	1	0	1.011281	-3.216197	-0.088570
43	1	0	2.420854	-5.049264	-1.017613
44	1	0	4.799631	-4.642164	-1.536626
45	6	0	2.098211	2.034423	1.208506
46	7	0	1.005350	2.177224	1.546056
47	1	0	4.574443	3.280229	0.957503
48	6	0	6.900630	2.304931	0.046002
49	6	0	6.765701	-0.379434	-0.762147
50	6	0	8.019876	1.677600	-0.438054
51	6	0	7.944136	0.338578	-0.839122
52	1	0	8.828977	-0.152812	-1.220036

53	1	0	6.718150	-1.410577	-1.072662
54	1	0	6.937189	3.339085	0.362130
55	1	0	8.956549	2.210991	-0.510319

Ligand	Description	Total Gibbs Free energy (M06-2X/Def2TZVPP)
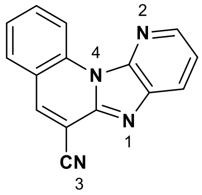<br>6a	2:1 complex with Cu^{2+} bound to N1	-3230.477514 a.u.

CARTESIAN COORDINATES

Center Number	Atomic Number	Atomic Type	Coordinates (Angstroms) X	Y	Z
1	7	0	4.159123	0.261918	0.005888
2	6	0	5.314902	-0.380545	-0.445312
3	6	0	2.918805	-0.299862	-0.070015
4	7	0	1.959174	0.486506	0.408472
5	6	0	2.600896	1.635181	0.826752
6	6	0	3.976050	1.523733	0.589179
7	6	0	2.773653	-1.604957	-0.631033
8	7	0	4.896724	2.421565	0.862354
9	6	0	2.132435	2.806076	1.413767
10	6	0	4.435708	3.535014	1.421126
11	6	0	3.081489	3.762812	1.709563
12	1	0	5.173610	4.290314	1.656782
13	1	0	2.794586	4.698385	2.166586
14	1	0	1.082265	2.957346	1.620925
15	6	0	5.163084	-1.672223	-1.003254
16	6	0	3.876141	-2.265436	-1.085494
17	1	0	3.778756	-3.254124	-1.514698
18	6	0	6.571747	0.212057	-0.358332
19	6	0	6.305063	-2.350427	-1.469815
20	6	0	7.542262	-1.765425	-1.383237
21	6	0	7.667077	-0.486101	-0.827097
22	1	0	6.186644	-3.337837	-1.895996
23	1	0	8.418474	-2.286143	-1.741301
24	1	0	8.644511	-0.028350	-0.760614
25	1	0	6.676259	1.196391	0.068163
26	6	0	1.444290	-2.114346	-0.662845
27	7	0	0.328727	-2.394194	-0.621312
28	29	0	0.024642	0.048956	0.378615
29	7	0	-1.945889	-0.390676	0.469607
30	6	0	-2.967180	0.357662	-0.038540
31	7	0	-4.167779	-0.279246	0.040341
32	6	0	-3.907505	-1.522199	0.639594
33	6	0	-2.502345	-1.536826	0.886219
34	6	0	-1.952602	-2.680521	1.501046
35	7	0	-4.757786	-2.465294	0.917847
36	6	0	-5.358423	0.277676	-0.415241
37	6	0	-2.878399	1.635558	-0.599182
38	6	0	-4.037102	2.235820	-1.068495
39	6	0	-5.273404	1.583379	-0.987147
40	6	0	-2.837538	-3.682796	1.799496
41	6	0	-4.211023	-3.544616	1.496714
42	1	0	-0.895555	-2.751044	1.712210
43	1	0	-2.502946	-4.595156	2.271093
44	1	0	-4.898170	-4.345093	1.738565
45	6	0	-1.589603	2.246262	-0.655950
46	7	0	-0.518856	2.660220	-0.664029
47	1	0	-3.984209	3.225099	-1.505619
48	6	0	-6.456866	2.197802	-1.467277
49	6	0	-6.577423	-0.382108	-0.329936
50	6	0	-7.652492	1.543958	-1.380201
51	6	0	-7.704734	0.257278	-0.811732
52	1	0	-8.657048	-0.252081	-0.747457

53	1	0	-6.639326	-1.367688	0.101725
54	1	0	-6.389753	3.187042	-1.900057
55	1	0	-8.558583	2.006331	-1.744247

Ligand	Description	Total Gibbs Free energy (M06-2X/Def2TZVPP)
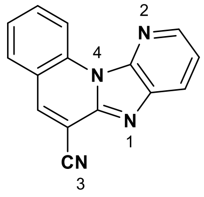 **6a**	2:1 complex with Mg^{2+} bound to N1	-1790.181722 a.u.

CARTESIAN COORDINATES

Center Number	Atomic Number	Atomic Type	Coordinates (Angstroms) X	Y	Z
1	7	0	-4.017158	-0.353609	0.170426
2	6	0	-5.323599	0.083994	-0.080504
3	6	0	-2.942143	0.128984	-0.500647
4	7	0	-1.784818	-0.418990	-0.122899
5	6	0	-2.130986	-1.328429	0.870305
6	6	0	-3.513922	-1.306763	1.072024
7	6	0	-3.131757	1.129908	-1.505591
8	7	0	-4.196110	-2.032118	1.929729
9	6	0	-1.373881	-2.200390	1.644201
10	6	0	-3.463349	-2.861484	2.662518
11	6	0	-2.069088	-2.974443	2.551757
12	1	0	-4.001308	-3.471177	3.376086
13	1	0	-1.548398	-3.676130	3.186692
14	1	0	-0.298483	-2.273562	1.545781
15	6	0	-5.500862	1.073600	-1.080567
16	6	0	-4.384977	1.587435	-1.786934
17	1	0	-4.538354	2.341785	-2.547781
18	6	0	-6.414499	-0.425294	0.614938
19	6	0	-6.802980	1.533274	-1.358894
20	6	0	-7.877262	1.028755	-0.671911
21	6	0	-7.675409	0.052324	0.310808
22	1	0	-6.937308	2.287876	-2.122604
23	1	0	-8.875928	1.380764	-0.886124
24	1	0	-8.525671	-0.343083	0.849544
25	1	0	-6.269795	-1.178129	1.372865
26	6	0	-1.887620	1.490999	-2.079292
27	7	0	-0.761056	1.546209	-2.318121
28	12	0	-0.000041	0.001662	-1.068172
29	7	0	1.784831	0.419012	-0.121585
30	6	0	2.942198	-0.127720	-0.500926
31	7	0	4.017170	0.353037	0.171523
32	6	0	3.513867	1.303582	1.075836
33	6	0	2.130924	1.325655	0.874211
34	6	0	1.373741	2.195279	1.650638
35	7	0	4.195994	2.026606	1.935547
36	6	0	5.323640	-0.083747	-0.080680
37	6	0	3.131863	-1.125668	-1.508812
38	6	0	4.385110	-1.582301	-1.791475
39	6	0	5.500966	-1.070460	-1.083590
40	6	0	2.068877	2.966923	2.560310
41	6	0	3.463158	2.853841	2.670675
42	1	0	0.298321	2.268469	1.552565
43	1	0	1.548117	3.666713	3.197279
44	1	0	4.001076	3.461575	3.385941
45	6	0	1.887703	-1.484871	-2.083691
46	7	0	0.761135	-1.539084	-2.322728
47	1	0	4.538541	-2.334399	-2.554535
48	6	0	6.803105	-1.529214	-1.363260
49	6	0	6.414493	0.423590	0.616236
50	6	0	7.877371	-1.026590	-0.674833
51	6	0	7.675450	-0.053046	0.310714
52	1	0	8.525678	0.340859	0.850606

53	1	0	6.269741	1.174192	1.376366
54	1	0	6.937487	-2.281596	-2.129148
55	1	0	8.876056	-1.377893	-0.890097

Ligand	Description	Total Gibbs Free energy (M06-2X/Def2TZVPP)
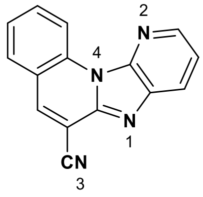<br>6a	2:1 complex with Zn^{2+} bound to N1	-3369.438978 a.u.

CARTESIAN COORDINATES

Center Number	Atomic Number	Atomic Type	X	Y	Z
1	7	0	-4.041309	0.205311	0.311051
2	6	0	-5.326453	-0.140012	-0.126487
3	6	0	-2.924054	-0.399131	-0.160187
4	7	0	-1.801947	0.072084	0.388524
5	6	0	-2.211947	1.050905	1.283671
6	6	0	-3.605074	1.154106	1.252888
7	6	0	-3.035773	-1.430220	-1.144175
8	7	0	-4.346769	1.972891	1.964122
9	6	0	-1.511783	1.884821	2.146322
10	6	0	-3.669603	2.765865	2.785417
11	6	0	-2.271409	2.753011	2.904941
12	1	0	-4.258101	3.448918	3.383224
13	1	0	-1.798642	3.431633	3.599628
14	1	0	-0.432575	1.858985	2.224084
15	6	0	-5.430351	-1.165099	-1.100166
16	6	0	-4.266291	-1.801625	-1.599096
17	1	0	-4.365011	-2.582409	-2.342128
18	6	0	-6.463876	0.488576	0.367789
19	6	0	-6.708346	-1.538118	-1.559969
20	6	0	-7.828911	-0.916975	-1.070819
21	6	0	-7.698940	0.092707	-0.109777
22	1	0	-6.787345	-2.321016	-2.302618
23	1	0	-8.809089	-1.203809	-1.423146
24	1	0	-8.585585	0.579104	0.273319
25	1	0	-6.374334	1.266440	1.108703
26	6	0	-1.756682	-1.927048	-1.503220
27	7	0	-0.617922	-2.099585	-1.569669
28	30	0	0.000128	-0.719770	0.000469
29	7	0	1.801820	0.072963	-0.387452
30	6	0	2.924185	-0.398758	0.160322
31	7	0	4.041250	0.205806	-0.311214
32	6	0	3.604603	1.155192	-1.252266
33	6	0	2.211443	1.052243	-1.282258
34	6	0	1.510884	1.886732	-2.144016
35	7	0	4.345986	1.974288	-1.963460
36	6	0	5.326592	-0.139878	0.125445
37	6	0	3.036308	-1.430306	1.143774
38	6	0	4.267040	-1.802046	1.597862
39	6	0	5.430901	-1.165429	1.098591
40	6	0	2.270168	2.755282	-2.902565
41	6	0	3.668437	2.767867	-2.783862
42	1	0	0.431640	1.860922	-2.221212
43	1	0	1.797075	3.434382	-3.596565
44	1	0	4.256675	3.451227	-3.381572
45	6	0	1.757370	-1.927185	1.503305
46	7	0	0.618617	-2.099486	1.570579
47	1	0	4.366073	-2.583174	2.340489
48	6	0	6.709102	-1.538814	1.557515
49	6	0	6.463814	0.488806	-0.369160
50	6	0	7.829473	-0.917581	1.068037
51	6	0	7.699094	0.092564	0.107539
52	1	0	8.585583	0.579031	-0.275828

53	1	0	6.373962	1.267039	-1.109648
54	1	0	6.788413	-2.322069	2.299756
55	1	0	8.809809	-1.204698	1.419693

Ligand	Description	Total Gibbs Free energy (M06-2X/Def2TZVPP)
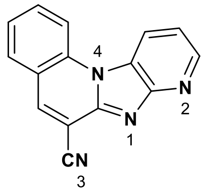 **6b**	2:1 complex with H^+ bound to N1	-1590.882632 a.u.

CARTESIAN COORDINATES

Center Number	Atomic Number	Atomic Type	Coordinates (Angstroms) X	Y	Z
1	7	0	-3.503036	0.157642	0.083036
2	6	0	-4.587924	-0.641734	-0.282463
3	6	0	-2.214258	-0.124503	-0.322745
4	7	0	-1.332106	0.742299	0.105868
5	6	0	-2.041268	1.650538	0.851058
6	6	0	-3.409711	1.315067	0.858211
7	6	0	-1.961906	-1.289294	-1.117658
8	6	0	-4.289879	2.116419	1.577298
9	7	0	-1.502126	2.683728	1.482520
10	6	0	-3.729116	3.196720	2.231134
11	6	0	-2.351273	3.439342	2.158259
12	1	0	-4.354323	3.860743	2.809433
13	1	0	-1.928572	4.288742	2.680388
14	6	0	-4.328000	-1.785132	-1.066618
15	6	0	-2.992116	-2.092389	-1.469900
16	1	0	-2.814512	-2.979257	-2.063268
17	6	0	-5.897287	-0.346540	0.092501
18	6	0	-5.397125	-2.611745	-1.445433
19	6	0	-6.684273	-2.318008	-1.066264
20	6	0	-6.926884	-1.178470	-0.297207
21	1	0	-5.184999	-3.486868	-2.045548
22	1	0	-7.502088	-2.958893	-1.361723
23	1	0	-7.937635	-0.936391	0.000584
24	1	0	-6.120149	0.526549	0.677836
25	6	0	-0.603015	-1.550689	-1.475392
26	7	0	0.504333	-1.723991	-1.723136
27	1	0	0.288223	0.775519	0.011945
28	7	0	1.364352	0.794086	-0.090273
29	6	0	2.212997	-0.125711	0.338810
30	7	0	3.483210	0.154040	-0.077478
31	6	0	3.415560	1.325764	-0.848047
32	6	0	2.067288	1.708177	-0.841683
33	7	0	1.543199	2.749471	-1.455198
34	6	0	4.311839	2.105023	-1.572848
35	6	0	4.565461	-0.663475	0.268212
36	6	0	1.932940	-1.286583	1.116036
37	6	0	2.961583	-2.110255	1.437121
38	6	0	4.294179	-1.819088	1.028426
39	6	0	2.407645	3.487165	-2.133928
40	6	0	3.776577	3.203492	-2.217507
41	1	0	2.002165	4.349848	-2.646783
42	1	0	4.417107	3.850121	-2.798572
43	6	0	0.585709	-1.556431	1.509643
44	7	0	-0.497856	-1.773194	1.816132
45	1	0	2.772908	-3.003881	2.016889
46	6	0	5.355003	-2.668971	1.384058
47	6	0	5.876039	-0.369113	-0.101508
48	6	0	6.641964	-2.379873	1.005661
49	6	0	6.895704	-1.222321	0.265513
50	1	0	7.908858	-0.983081	-0.026078
51	1	0	6.111272	0.518068	-0.658991
52	1	0	5.134488	-3.554434	1.965297

53	1	0	7.454279	-3.036386	1.281341
54	1	0	-5.349027	1.939501	1.653695
55	1	0	5.363495	1.897235	-1.663181

Ligand	Description	Total Gibbs Free energy (M06-2X/Def2TZVPP)
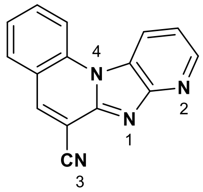 6b	2:1 complex with Ca^{2+} bound to N1	-2267.758061 a.u.

CARTESIAN COORDINATES

Center Number	Atomic Number	Atomic Type	Coordinates (Angstroms) X	Y	Z
1	7	0	4.507357	0.352697	0.198506
2	6	0	5.802968	-0.088421	-0.098806
3	6	0	3.419892	-0.170891	-0.440393
4	7	0	2.272323	0.349749	-0.049454
5	6	0	2.611274	1.278107	0.905512
6	6	0	4.002056	1.311772	1.091604
7	6	0	3.572250	-1.184347	-1.436118
8	6	0	4.519021	2.203405	2.025769
9	7	0	1.716110	2.020859	1.536141
10	6	0	3.590964	2.984060	2.691378
11	6	0	2.220101	2.867490	2.424520
12	1	0	3.922620	3.698640	3.429969
13	1	0	1.512997	3.490006	2.956684
14	6	0	5.949527	-1.094038	-1.086918
15	6	0	4.813101	-1.635475	-1.752718
16	1	0	4.954310	-2.400610	-2.504674
17	6	0	6.925417	0.426680	0.539078
18	6	0	7.241434	-1.552355	-1.404050
19	6	0	8.344099	-1.037748	-0.769675
20	6	0	8.178312	-0.047268	0.202239
21	1	0	7.347790	-2.319749	-2.159450
22	1	0	9.333520	-1.393316	-1.017498
23	1	0	9.044924	0.360398	0.704160
24	1	0	6.833311	1.189250	1.291974
25	6	0	2.300838	-1.558581	-1.958089
26	7	0	1.174380	-1.642911	-2.176214
27	20	0	-0.000248	0.003478	-0.717893
28	7	0	-2.272029	-0.346706	-0.048529
29	6	0	-3.420553	0.171599	-0.439800
30	7	0	-4.507052	-0.353319	0.199671
31	6	0	-3.999984	-1.310723	1.093554
32	6	0	-2.609277	-1.274774	0.907328
33	7	0	-1.712789	-2.015375	1.538598
34	6	0	-4.515336	-2.202390	2.028588
35	6	0	-5.803469	0.085249	-0.097895
36	6	0	-3.574837	1.183978	-1.436364
37	6	0	-4.816513	1.632698	-1.753147
38	6	0	-5.951902	1.089829	-1.086776
39	6	0	-2.215242	-2.862094	2.427753
40	6	0	-3.585881	-2.980822	2.694843
41	1	0	-1.506990	-3.482808	2.960502
42	1	0	-3.916240	-3.695297	3.434115
43	6	0	-2.304190	1.560102	-1.958825
44	7	0	-1.177935	1.646449	-2.177196
45	1	0	-4.959141	2.396959	-2.505723
46	6	0	-7.244646	1.545592	-1.404184
47	6	0	-6.924966	-0.431326	0.540479
48	6	0	-8.346354	1.029535	-0.769329
49	6	0	-8.178726	0.040147	0.203379
50	1	0	-9.044569	-0.368657	0.705703
51	1	0	-6.831468	-1.193156	1.293948
52	1	0	-7.352408	2.312183	-2.160200

53	1	0	-9.336425	1.383124	-1.017384
54	1	0	5.567551	2.312763	2.247225
55	1	0	-5.563656	-2.313350	2.250232

Ligand	Description	Total Gibbs Free energy (M06-2X/Def2TZVPP)
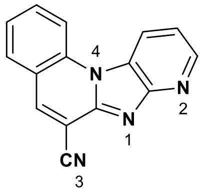 **6b**	2:1 complex with Cu^{2+} bound to N1	-3230.506830 a.u.

CARTESIAN COORDINATES

Center Number	Atomic Number	Atomic Type	Coordinates (Angstroms) X	Y	Z
1	7	0	4.121150	0.346227	0.116233
2	6	0	5.359982	-0.182337	-0.271473
3	6	0	2.958670	-0.235110	-0.282593
4	7	0	1.884614	0.387181	0.180959
5	6	0	2.356498	1.447961	0.926071
6	6	0	3.757904	1.452083	0.907120
7	6	0	2.956456	-1.390288	-1.118627
8	6	0	4.419262	2.453321	1.611597
9	7	0	1.574761	2.302986	1.555105
10	6	0	3.610820	3.360350	2.271444
11	6	0	2.214199	3.254605	2.221972
12	1	0	4.057702	4.164054	2.837641
13	1	0	1.599385	3.972721	2.748349
14	6	0	5.358946	-1.334475	-1.097786
15	6	0	4.137679	-1.929587	-1.517788
16	1	0	4.164192	-2.804747	-2.153512
17	6	0	6.564654	0.385041	0.127210
18	6	0	6.590593	-1.883327	-1.501746
19	6	0	7.774540	-1.316356	-1.103731
20	6	0	7.753879	-0.181122	-0.288019
21	1	0	6.583838	-2.762152	-2.132919
22	1	0	8.717140	-1.741396	-1.416335
23	1	0	8.685541	0.267718	0.027759
24	1	0	6.586671	1.257605	0.755043
25	6	0	1.615827	-1.772663	-1.404483
26	7	0	0.464473	-1.789245	-1.436170
27	29	0	-0.000022	0.000337	-0.146762
28	7	0	-1.884565	-0.386752	0.181052
29	6	0	-2.958759	0.235249	-0.282553
30	7	0	-4.121110	-0.346293	0.116364
31	6	0	-3.757614	-1.451972	0.907374
32	6	0	-2.356206	-1.447545	0.926293
33	7	0	-1.574264	-2.302321	1.555409
34	6	0	-4.418734	-2.453278	1.611973
35	6	0	-5.360068	0.181960	-0.271360
36	6	0	-2.956838	1.390326	-1.118745
37	6	0	-4.138188	1.929316	-1.517937
38	6	0	-5.359311	1.333998	-1.097814
39	6	0	-2.213477	-3.254003	2.222404
40	6	0	-3.610072	-3.360051	2.271912
41	1	0	-1.598494	-3.971916	2.748861
42	1	0	-4.056767	-4.163795	2.838200
43	6	0	-1.616274	1.772887	-1.404643
44	7	0	-0.464919	1.789446	-1.436187
45	1	0	-4.164910	2.804385	-2.153778
46	6	0	-6.591093	1.882536	-1.501789
47	6	0	-6.564600	-0.385629	0.127445
48	6	0	-7.774900	1.315357	-1.103656
49	6	0	-7.753963	0.180228	-0.287806
50	1	0	-8.685518	-0.268773	0.028062
51	1	0	-6.586389	-1.258114	0.755395
52	1	0	-6.584555	2.761287	-2.133067

53	1	0	-8.717604	1.740157	-1.416271
54	1	0	5.490094	2.553296	1.665655
55	1	0	-5.489541	-2.553520	1.666077

Ligand	Description	Total Gibbs Free energy (M06-2X/Def2TZVPP)
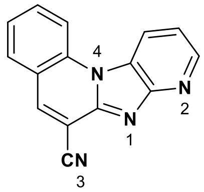 **6b**	2:1 complex with Mg^{2+} bound to N1	-1790.187739 a.u.

CARTESIAN COORDINATES

Center Number	Atomic Number	Atomic Type	Coordinates (Angstroms) X	Y	Z
1	7	0	-4.020016	0.436419	-0.189256
2	6	0	-5.329678	-0.030505	-0.014815
3	6	0	-2.983050	-0.095218	0.515725
4	7	0	-1.814468	0.459153	0.234782
5	6	0	-2.089075	1.421310	-0.715313
6	6	0	-3.461670	1.435685	-1.005261
7	6	0	-3.204157	-1.145701	1.460367
8	6	0	-3.928333	2.350832	-1.943651
9	7	0	-1.168848	2.201864	-1.251161
10	6	0	-2.972223	3.172716	-2.510222
11	6	0	-1.623271	3.070113	-2.144182
12	1	0	-3.263891	3.907799	-3.245623
13	1	0	-0.890849	3.724150	-2.598907
14	6	0	-5.543796	-1.072820	0.922579
15	6	0	-4.460106	-1.623377	1.659588
16	1	0	-4.649384	-2.415913	2.371545
17	6	0	-6.403813	0.493539	-0.724311
18	6	0	-6.851041	-1.557284	1.117292
19	6	0	-7.904865	-1.032634	0.413103
20	6	0	-7.673097	-0.006474	-0.507316
21	1	0	-7.007240	-2.352113	1.834681
22	1	0	-8.906428	-1.407106	0.566372
23	1	0	-8.501284	0.409139	-1.064636
24	1	0	-6.263704	1.282752	-1.440834
25	6	0	-1.968628	-1.508162	2.063227
26	7	0	-0.848880	-1.534444	2.330339
27	12	0	-0.000041	-0.000461	1.068853
28	7	0	1.814499	-0.459694	0.234865
29	6	0	2.982924	0.095011	0.515822
30	7	0	4.020054	-0.436363	-0.189114
31	6	0	3.462006	-1.435807	-1.005106
32	6	0	2.089400	-1.421806	-0.715194
33	7	0	1.169401	-2.202623	-1.251044
34	6	0	3.928940	-2.350845	-1.943467
35	6	0	5.329584	0.030918	-0.014645
36	6	0	3.203712	1.145592	1.460421
37	6	0	4.459526	1.623623	1.659659
38	6	0	5.543390	1.073330	0.922712
39	6	0	1.624082	-3.070767	-2.144037
40	6	0	2.973070	-3.173006	-2.510041
41	1	0	0.891848	-3.725016	-2.598761
42	1	0	3.264961	-3.908024	-3.245419
43	6	0	1.968084	1.507768	2.063244
44	7	0	0.848339	1.533807	2.330399
45	1	0	4.648560	2.416246	2.371584
46	6	0	6.850501	1.558145	1.117456
47	6	0	6.403885	-0.492877	-0.724076
48	6	0	7.904490	1.033740	0.413332
49	6	0	7.673028	0.007478	-0.507053
50	1	0	8.501349	-0.407943	-1.064317
51	1	0	6.264010	-1.282163	-1.440564
52	1	0	7.006465	2.353045	1.834817

53	1	0	8.905949	1.408478	0.566629
54	1	0	-4.959487	2.448192	-2.238353
55	1	0	4.960128	-2.447926	-2.238143

Ligand	Description	Total Gibbs Free energy (M06-2X/Def2TZVPP)
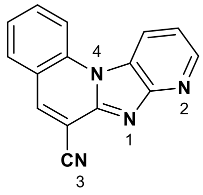 6b	2:1 complex with Zn^{2+} bound to N1	-3369.443729 a.u.

CARTESIAN COORDINATES

Center Number	Atomic Number	Atomic Type	Coordinates (Angstroms) X	Y	Z
1	7	0	4.037650	-0.386509	-0.224045
2	6	0	5.333185	0.070460	0.055068
3	6	0	2.956382	0.129333	0.421319
4	7	0	1.814282	-0.418804	0.035285
5	6	0	2.150078	-1.355860	-0.919317
6	6	0	3.539636	-1.361800	-1.106903
7	6	0	3.107884	1.150968	1.408658
8	6	0	4.073323	-2.251541	-2.034260
9	7	0	1.271254	-2.118900	-1.539182
10	6	0	3.161423	-3.056057	-2.691313
11	6	0	1.789405	-2.962589	-2.421145
12	1	0	3.506333	-3.770569	-3.423937
13	1	0	1.091771	-3.602603	-2.945122
14	6	0	5.481679	1.082865	1.036080
15	6	0	4.348791	1.615410	1.709192
16	1	0	4.488054	2.386585	2.455332
17	6	0	6.453559	-0.435115	-0.593842
18	6	0	6.772371	1.557177	1.336920
19	6	0	7.872332	1.050894	0.692486
20	6	0	7.704807	0.054164	-0.273255
21	1	0	6.878516	2.329526	2.087245
22	1	0	8.861076	1.417491	0.926678
23	1	0	8.569599	-0.346533	-0.783913
24	1	0	6.363667	-1.200922	-1.342890
25	6	0	1.838853	1.515082	1.938463
26	7	0	0.705371	1.561496	2.139881
27	30	0	0.000003	0.000092	0.736429
28	7	0	-1.814291	0.418923	0.035314
29	6	0	-2.956347	-0.129323	0.421325
30	7	0	-4.037660	0.386493	-0.223984
31	6	0	-3.539729	1.361891	-1.106769
32	6	0	-2.150166	1.356026	-0.919214
33	7	0	-1.271403	2.119175	-1.539032
34	6	0	-4.073493	2.251697	-2.034022
35	6	0	-5.333159	-0.070597	0.055106
36	6	0	-3.107761	-1.151032	1.408598
37	6	0	-4.348633	-1.615571	1.709130
38	6	0	-5.481568	-1.083062	1.036069
39	6	0	-1.789627	2.962913	-2.420903
40	6	0	-3.161659	3.056328	-2.691024
41	1	0	-1.092046	3.603016	-2.944842
42	1	0	-3.506630	3.770896	-3.423564
43	6	0	-1.838704	-1.515105	1.938371
44	7	0	-0.705223	-1.561526	2.139793
45	1	0	-4.487831	-2.386798	2.455227
46	6	0	-6.772223	-1.557484	1.336896
47	6	0	-6.453573	0.434909	-0.593788
48	6	0	-7.872226	-1.051254	0.692490
49	6	0	-7.704782	-0.054479	-0.273218
50	1	0	-8.569607	0.346162	-0.783863
51	1	0	-6.363735	1.200737	-1.342822
52	1	0	-6.878309	-2.329876	2.087185

53	1	0	-8.860941	-1.417937	0.926673
54	1	0	5.123040	-2.342595	-2.255984
55	1	0	-5.123223	2.342723	-2.255695
